# Virtual Care, What Are We Measuring and What Should We Measure? Scoping Review of Reviews

**DOI:** 10.2196/65312

**Published:** 2025-12-01

**Authors:** Melanie Powis, Anum Ali, Jackelyn Salmini, Saidah Hack, Rouhi Fazelzad, Lisa Barbara, Alejandro Berlin, Matthew Cheung, Mary DeVera, Annemarie Edwards, Helen McTaggart-Cowan, Robert Olson, Stuart Peacock, Ambreen Sayani, Simron Singh, Monika K Krzyzanowska

**Affiliations:** 1 Cancer Quality Lab (CQuaL), Princess Margaret Cancer Centre, University Health Network, 610 University Ave, Toronto, ON, M5G2M9, Canada, 1 905-713-5450; 2 Dalla Lana School of Public Health, University of Toronto, Toronto, ON, Canada; 3 Library and Information Services, University Health Network, Toronto, ON, Canada; 4 Department of Radiation Oncology, Arthur JE Child Comprehensive Cancer Centre, University of Calgary, Calgary, AB, Canada; 5 Cancer Digital Intelligence, Princess Margaret Cancer Centre, University Health Network, Toronto, ON, Canada; 6 Odette Cancer Centre, Sunnybrook Health Sciences Centre, Toronto, ON, Canada; 7 Faculty of Pharmaceutical Sciences, University of British Columbia, Vancouver, BC, Canada; 8 Cancer Strategy and Innovation, Canadian Cancer Society, Toronto, ON, Canada; 9 Faculty of Health Sciences, Simon Fraser University, Burnaby, BC, Canada; 10 Cancer Control Research, BC Cancer Agency, Vancouver, BC, Canada; 11 Department of Surgery, University of British Columbia, Vancouver, BC, Canada; 12 Institute for Health System Solutions and Virtual Care, Women’s College Hospital, Toronto, ON, Canada; 13 Patient Centred Care, Cancer Care Ontario, Ontario Health, Toronto, ON, Canada

**Keywords:** virtual care, telemedicine, quality measure, telehealth, evaluation, systematic review, clinical outcomes, health equity, remote monitoring

## Abstract

**Background:**

Virtual care is here to stay; however, there remains no comprehensive measurement framework to guide evaluation of its impacts, to inform policy decisions, and to support optimization of practice.

**Objective:**

This study aimed to conduct a scoping review of reviews to synthesize measures related to virtual care evaluation across clinical conditions and contexts to identify gaps in current evaluation measures and to inform the development of recommendations for future work.

**Methods:**

Citations published from 2015 to 2023 were retrieved from MEDLINE, Cochrane Database of Systematic Reviews, Embase, Emcare, Scopus, CINAHL, and Web of Science using search terms grouped by key concepts (virtual care and evaluation or quality measurement). Measures were defined as any quantitative or qualitative evaluation of performance or impact of virtual care on processes, outcomes, or systems. Articles were excluded if they were not a literature review (eg, primary results, commentaries, letters, protocols), dealt exclusively with pediatric populations, were published in a language other than English, or were abstracts only. Measures from retained articles (1233) were thematically grouped against the Proctor Implementation Research Outcomes framework. The study was reported according to the PRISMA (Preferred Reporting Items for Systematic Reviews and Meta-Analyses) guideline extension for scoping reviews.

**Results:**

There has been substantial growth in the virtual care literature, particularly since the start of the COVID-19 pandemic. The majority of articles (900/1233, 73.0%) evaluated client outcomes, including satisfaction with virtual care, usability or functionality of platforms, or clinical outcomes. Relative to the other domains of the Proctor framework, implementation measures were poorly defined, and many of the measures were proxy rather than direct measures. Despite the potential impacts of virtual care on health equity, most studies examining health equity were purely qualitative. Measures of safety, privacy, and security of virtual care were sparse and poorly defined. Caregivers play an important role in facilitating virtual visits and providing informal technical support; however, few studies examined implementation or satisfaction with virtual care from the perspective of caregivers. Additionally, clinician experience and acceptance of virtual care have implications for availability and adoption; however, relative to patients, few articles examined this perspective.

**Conclusions:**

Our study highlights gaps in current evaluations of virtual care. Work is needed to improve the quality and standardization of virtual care evaluation to ensure reproducibility, generalizability, and comparability of findings. Additionally, compliance with existing measure definitions and conventions should extend to virtual care. Finally, additional theoretical work is needed to standardize and conceptually frame future virtual care evaluations. Future studies should include both the caregiver and clinician as unique perspectives in evaluations and should embed systematic evaluations of the impact of social determinants of health on virtual care access, adoption, and perceptions of care.

## Introduction

Until recently, usage of virtual care (VC; synchronous video or telephone visits) was largely confined to remote monitoring of chronic health conditions [1,2] and to the delivery of subspecialty care in remote or rural populations [3]. However, the COVID-19 pandemic forced rapid and unplanned uptake as VC advanced from an opportunity to a necessity [4]. Findings of emerging quantitative and qualitative studies indicate that both patients [5,6] and clinicians [6,7] see benefits to VC, particularly with respect to the lessened impact of attending appointments on patients’ finances and time, and the convenience of accessing care. However, both patients [8] and clinicians [7] have expressed concerns regarding the potential negative impacts of VC on informational and relational continuity of care, particularly when interactions involve difficult conversations or physical examinations [6-8].

Patients expect VC to remain an option moving forward [9], and as such, it is unlikely that usage will recede back to prepandemic levels. To date, many evaluations of VC have been specific to a clinical scenario or patient population or have only focused on a small number of measures. As we transition to more long-term adoption, there is a need for higher quality evaluation [10] to understand the broader impacts of VC on care delivery, stakeholder experiences, and clinical outcomes and to inform policy decisions and optimize delivery of virtual visits [11-13]. However, there has been no synthesis of existing measures of VC to identify gaps in evaluation across clinical conditions and contexts, which is needed to inform future work.

To this end, we undertook a scoping review of reviews to understand what is currently measured when evaluating VC. We used a review of reviews methodology, given the volume of literature evaluating VC to date, and the substantial number of review articles published on the topic. Existing measures from the literature were thematically grouped against the Proctor implementation research outcomes framework [14] to examine gaps in current measurement. The Proctor framework was chosen as it is comprehensive yet simple, commonly used within the implementation and evaluation literature, and focused on outcomes. This informed the development of a set of recommendations for future work, particularly with respect to future evaluations, research needs, and theoretical or conceptual needs [15].

## Methods

### Data Sources

The study was reported according to the PRISMA (Preferred Reporting Items for Systematic Reviews and Meta-Analyses) guideline extension for reporting scoping reviews [16]; the review protocol was not registered with a database. Citations were retrieved from MEDLINE, Cochrane database, Embase, Emcare, Scopus, CINAHL, and Web of Science, using search terms grouped by key concepts (VC and evaluation or quality measurement; Multimedia Appendix 1); syntax was translated as appropriate for included databases. Review articles published from January 2015 to May 2023 were retained; earlier articles were excluded for multiple reasons: (1) technological changes, (2) the need to balance comprehensiveness of the search with manageability of the volume of articles, and (3) the inclusion of reviews that incorporated studies published before 2015, which allowed us to capture relevant earlier work.

### Study Selection

Titles and abstracts were independently screened for relevance by 2 members of the research team trained in literature review methods (MP and AA, JS, or SH), whereas full-text articles were independently screened for inclusion by 2 members of the research team (MP and AA or JS). Screening was undertaken in Covidence (Veritas Health Innovation, Melbourne, Australia). Articles were retained if they reported on any type of systematic review with the exclusion of narrative reviews and reviews of reviews, were published since January 2015, examined VC delivered in the outpatient setting, were published in English, and reported on some evaluation measure. For the purposes of this work, VC was defined as any synchronous communication between patients and clinicians (people providing direct patient care) that takes place remotely.

### Exclusions

Eligibility issues were addressed through consensus discussions with a third reviewer (MKK) as required. Articles that reported on virtual reality interventions or reported on *eHealth* or *mobile health* interventions that were not delivered synchronously or were undefined were excluded. Measures were defined as any quantitative or qualitative evaluation of the performance or impact of VC on processes, outcomes, or systems. Articles were excluded if they were not a literature review (eg, primary results, commentaries, letters, and protocols). As there are different legal and ethical requirements for the use of VC in pediatric populations, and the role of the caregiver in care is different from that of adult populations, articles that dealt exclusively with pediatric populations were excluded. Conference abstracts in the absence of an accompanying full-text article were excluded as they provided insufficient details for analysis.

### Synthesis

Details of retained articles (dates of publication, clinical condition or population, description of the modality of VC delivery, details of measures, data collection methods, barriers to VC, and key findings) were abstracted by 1 member of the study team (AA) into a standardized abstraction template in Covidence. To ensure abstraction reliability, a second reviewer (MP) extracted data from a subset of 15 randomly selected manuscripts; no discrepancies were found between reviewers.

The abstracted data were exported as a CSV file to Microsoft Excel for analysis. Measures were grouped using deductive thematic analysis against the 3 domains and their constructs of the Proctor outcomes framework for implementation research (Table 1) [14]. Measures were captured under the construct specified within the original review article. As such, similar measures may be listed under more than one construct of the Proctor framework. The proportion of retained articles reporting on a measure was calculated out of those review articles reporting on the corresponding domain or construct, as appropriate.

**Table 1.** Proctor [14] domains and constructs.

Domain	Constructs
Implementation outcomes	Acceptability, adoption, appropriateness, costs, feasibility, fidelity, penetration, and sustainability
Service outcomes	Efficiency, safety, effectiveness, equity, patient-centeredness, and timeliness
Client outcomes	Satisfaction, function, and symptomatology

## Results

### Characteristics of Retained Articles

**Figure 1.**
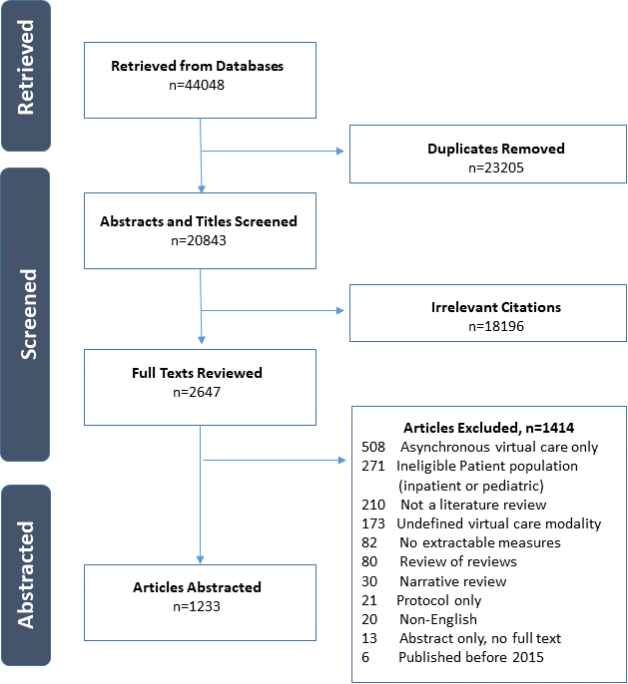
PRISMA (Preferred Reporting Items for Systematic Reviews and Meta-Analyses) diagram.

The search returned 44,048 articles, of which 1233 were retained for analysis. A PRISMA diagram is included in Figure 1, and details of the retained articles are described in Multimedia Appendix 2. A substantial proportion of the review articles (885/1233, 71.8%) were published following the shift to the adoption of virtual visits as a result of the COVID-19 pandemic (Table 2). Articles were frequently not specific to a clinical context or condition (197/1233, 16.0%), or dealt specifically with mental health conditions (181/1233, 14.7%) or cardiovascular conditions ( 121/1233, 9.8%). Most review articles evaluated telephone and video visits collectively (691/1233, 56.0%), or focused solely on telephone-based visits (256/1233, 20.8%). Client outcomes were the most commonly evaluated domain (900/1233, 73.0%), followed by service outcomes ( 507/1233, 41.1%) and implementation outcomes (476/1233, 38.6%).

**Table 2.** Characteristics of retained review articles (N=1233).

Characteristics	Values, n (%)
Year of publication	
2015	46 (3.7)
2016	60 (4.9)
2017	64 (5.2)
2018	84 (6.8)
2019	94 (7.6)
2020	164 (13.3)
2021	244 (19.8)
2022	349 (28.3)
2023	128 (10.4)
Clinical context	
Acute or urgent care	10 (0.8)
Sexual assault or intimate partner violence	6 (0.5)
Autoimmune conditions	7 (0.6)
Cancer	99 (8.0)
Cardiovascular	121 (9.8)
Central nervous system	72 (5.8)
Dentistry	9 (0.7)
Dermatology	17 (1.4)
End of life	18 (1.5)
Ear, nose, throat	12 (1.0)
Geriatric	23 (1.9)
Gastrointestinal	13 (1.1)
Genitourinary	9 (0.7)
Infectious diseases	47 (3.8)
Maternal health	50 (4.1)
Mental health	181 (14.7)
Metabolic	79 (6.4)
Musculoskeletal	46 (3.7)
Pain	18 (1.5)
Rehabilitation /occupational therapy	11 (0.9)
Respiratory	36 (2.9)
Surgery	35 (2.8)
Multiple conditions	197 (16.0)
All health conditions	83 (6.7)
Not specified	34 (2.8)
VC^a^ type	
Telephone and video	691 (56.0)
Internet-based NOS^b^	21 (1.7)
Mobile app	8 (0.6)
Multiple	25 (2.0)
Telemedicine NOS	13 (1.1)
Telemonitoring	6 (0.5)
Telephone	256 (20.8)
Telerehabilitation NOS	2 (0.2)
Video	208 (16.9)
Virtual NOS	3 (0.2)
Implementation outcomes (476/1233, 38.6%)	
Acceptability	109 (8.8)
Adoption	135 (10.9)
Appropriateness	9 (0.7)
Costs	221 (17.9)
Feasibility	108 (8.8)
Fidelity	45 (3.6)
Penetration	24 (1.9)
Sustainability	10 (0.8)
Service outcomes (507/1233, 41.1%)	
Efficiency	79 (6.4)
Safety	25 (2.0)
Effectiveness	365 (29.6)
Equity	28 (2.3)
Patient centeredness	36 (2.9)
Timeliness	44 (3.6)
Client outcomes (900/1233, 73.0%)	
Satisfaction	403 (32.7)
Function	62 (5.0)
Clinical outcomes	734 (59.5)

a VC: virtual care.

b NOS: not otherwise specified.

### Summary of Overall Findings

For each of the domains and constructs, Multimedia Appendix 3 provides details on the evaluation measures used to date, whereas Table 3 summarizes the high-level key findings. Across each of the domains, we found that articles frequently chose measures that did not match the construct that they indicated they were evaluating. For example, measures of acceptability were used interchangeably to evaluate acceptability, satisfaction, and usability of VC, despite these constructs being conceptually different. Additionally, we observed high heterogeneity in the measures that have been used to evaluate VC, particularly for acceptability, adoption, feasibility, and fidelity, which suggests a lack of consensus about what should be measured and how.

**Table 3.** Summary of key findings.

Domain	Key findings
Implementation outcomes	Relative to other domains of the Proctor framework, measures were less well defined in the implementation domain. There were inconsistent definitions across subdomains, particularly for acceptability, adoption, and feasibility, and many studies reported on proxy measures or used the concepts interchangeably. Few studies examining adoption or acceptability reported on either the clinician and system perspectives, which are likely to have implications for long-term adoption and sustainability of VC^a^. Few reviews examined cost from the patient perspective and even fewer from the societal perspective. Despite many studies highlighting the substantial role of caregivers in providing navigation and technical support in VC, relative to patients, implementation outcomes were not examined from the caregiver perspective. Concerns regarding the impact of VC on medical education were raised by clinicians, but few studies evaluated VC from the perspective of learners or trainees. Despite their importance for implementation, intervention, and implementation fidelity measures were rarely reported and rarely made use of standardized tools. There was limited exploration of factors influencing the penetration of VC among populations. Few reviews examined the sustainability of VC, either issues impacting long-term adoption or the impact of VC on environmental sustainability.
Service outcomes	Despite the potential for VC to impact health equity, there are very few studies examining disparities in VC delivery and uptake, and no studies reported objective or validated measures. There was limited exploration of specific inequities, such as socioeconomic status, race, gender (often used interchangeably with sex), and accessibility (including language and literacy barriers), and limited exploration of intersecting identities. Existing validated measures of patient-centeredness, such as the Picker survey, were not cited in the literature. Despite patients, clinicians, and health systems highlighting issues with the delivery of culturally appropriate care, none of the reviews objectively evaluated this. There is a lack of specific assessments on the safety of VC, and existing studies only cover a narrow range of topics and do not include patient perspectives. There are no objective measures of the quality of interactions, relationships, and trust. Additionally, relative to the patient perspective, few studies examined the quality of the interaction from the clinician perspective.
Client outcomes	Satisfaction was often measured through proxy measures such as acceptability, perceived usefulness, and adoption despite the availability of multiple widespread, validated survey measures. While there were many validated measures of satisfaction, patient experience, and clinician experience, there was a lack of consensus on the most appropriate measures of these concepts as they related to VC. Relative to patient experience, few reviews examined experience from the clinician perspective, which has implications for VC availability and long-term adoption. Despite the integral role of caregivers in facilitating virtual visits, caregiver satisfaction was rarely evaluated, and, compared to patients, few reviews examined caregiver experience. There were no objective measures of privacy or security of VC delivery. Clinical outcomes (psychosocial and physical) are well defined and include widely used objective physical measurements and validated survey instruments; however, appropriate comparators are not always used, limiting interpretability.

a VC; virtual care.

### Implementation Outcomes

Of those review articles reporting on implementation outcomes (n=476), the most commonly reported implementation measures were related to costs of VC (221/476, 46.4%), adoption into practice (135/476, 28.4%), and acceptability (109/476, 22.9%; Multimedia Appendix 3). However, there appeared to be a lack of consensus on definitions of outcomes within the implementation domain, particularly for acceptability, adoption, and feasibility, where many review articles reported on proxy rather than direct measures or used the concepts interchangeably. Of articles reporting on adoption or acceptability, evaluations of the patient perspective were far more common than those of clinicians or the health system (adoption: patient 92.6% vs clinician 5.2% vs system 1.5%; acceptability: patient 96.3% vs clinician 17.4%).

Few review articles reported on caregiver or trainee perspectives on implementation despite reports that these groups may have unique experiences with VC beyond those of patients and clinicians. There were few articles examining penetration of VC (24/476, 5.0%), and those that did most frequently described availability of virtual visits (5/24, 20.8%), satisfaction of key stakeholders with access to VC (4/24, 16.6%), or sociodemographic factors associated with access to VC (4/24, 16.6%), most often race or ethnicity (3/24, 12.5%). Measures of appropriateness of virtual visits for different clinical scenarios, patient populations, or visit types were sparsely reported (9/476, 1.9%), as were measures of long-term adoption or sustainability (10/476, 2.1%). Additionally, few articles examined fidelity of virtual visits (45/476, 9.5%), and those that did focused mostly on fidelity of the intervention (43/45, 95.6%), examining diagnostic accuracy (32/43, 74.4%) or concordance with an intervention protocol (10/43, 23.2%).

### Service Outcomes

Of those review articles reporting on service outcomes (n=507), the most commonly reported outcome was effectiveness (365/507, 72.0%); while there was a high heterogeneity of measures, effectiveness was most commonly reported as uptake of health behaviors as a result of exposure to VC (201/365, 55.1%), adherence to treatment (123/365, 30.1%), or as a change in self-efficacy on a validated scale (111/365, 30.4%; Multimedia Appendix 3). Few review articles evaluated the safety of VC (25/507, 4.9%), and those that did only covered a narrow range of topics, including adverse event rates (9/25, 36.0%) and acute care utilization (6/25, 24.0%), generally from the clinician perspective. Additionally, few review articles evaluated the impact of VC on health equity (28/507, 5.5%), with most (25/28, 89.3%) focusing on marginalized or vulnerable populations, especially Indigenous populations (8/25, 32.0%) and racialized populations (7/25, 28.0%). There was limited evaluation of equity issues around VC as they related to gender (4/28, 14.3%) or language/literacy barriers (1/28, 3.6%). Of note, none of the articles described the use of objective or validated measures for assessing disparities in care. Of articles examining the quality of clinical interactions delivered virtually (36/507, 7.1%), most focused on capturing the patient perspective (34/36, 94.4%), whereas few captured the clinician perspective (4/36, 11.1%), and usage of existing validated measures was limited.

### Client Outcomes

The majority of the retained review articles included client outcomes (n=900). Approximately half of the review articles reporting on client outcomes evaluated satisfaction with virtual visits (403/900, 44.7%), although proxy measures such as acceptability, perceived usefulness, and adoption were prevalent and heterogeneity of validated measures was high (Table 3 and Multimedia Appendix 3). Both satisfaction and experience with VC were more often evaluated from the perspective of patients rather than clinicians (satisfaction: 93.3% vs 28.5%; experience: 59.6% vs 31.8%). Additionally, caregiver satisfaction was rarely evaluated, whereas few reviews examined caregivers’ experiences with VC (40/151, 26.4%). Usability was more frequently evaluated from the patient rather than clinician perspective (89.7% vs 12.1%), and patient-focused measures of usability were more comprehensive, as they captured aspects of functionality, such as ease of use, perceptions of technical features, and factors associated with patterns of usage that were not included in clinician-focused evaluations.

A minority of articles evaluated privacy and security of VC platforms (7/62, 11.3%), most often through qualitative themes from inquiry around patient and clinician perceptions (5/7, 71.4%), although none cited objective measures. The most commonly reported client outcomes were clinical outcomes (734/900, 81.5%), including both psychosocial (438/734, 59.7%) and physical (560/734, 76.3%). While there was high heterogeneity of measures that were used, they were generally appropriate to the clinical context or population under study and included widely used objective physical measurements and validated survey instruments.

## Discussion

### Overview of Findings

There has been substantial growth in the literature evaluating outcomes of VC, especially since the start of the COVID-19 pandemic. The majority of review articles evaluating VC focused on client outcomes (900/1233, 73.0%), which included usability or functionality of the VC systems (62/900, 6.88%); satisfaction and experience with VC from the patient, caregiver, or clinician perspectives (403/900, 44.7%), and clinical outcomes of care delivered virtually (734/900, 81.5%), including mortality. Our study highlights gaps in current evaluations of VC. Through this work, we have identified a number of opportunities to improve how VC is evaluated, what aspects of VC have been understudied, and what type of methodological work needs to be undertaken to facilitate future assessments. To our knowledge, this is the largest scoping literature review to date examining the evaluation of VC across all clinical contexts.

### Implementation Outcomes

Despite the growing body of published work stressing the importance of evaluating implementation in understanding interventions, given the influence of context and implementation processes on the success of an intervention [17-19], fewer articles reported outcomes related to implementation of VC ( 476/1233, 38.6%). Of the articles examining implementation, most of these articles focused on capturing costs of implementing, delivering, or receiving virtual visits (221/476, 46.4%), whereas relatively few examined intervention or implementation fidelity (45/476, 9.5%); penetration or access to virtual visits (24/476, 5.0%); or appropriateness of VC for different types of visits, patient populations, or clinical contexts (9/476, 1.9%). In Table 4, we highlight recommendations for the conduct of future VC evaluations, areas where research should fill in current evidence gaps, and areas for future theoretical and conceptual development.

**Table 4.** Recommendations for future work.

Domain	Recommendations		
	Conduct of evaluations	Key research questions	Theory development
Implementation outcomes	When examining concepts of acceptability, satisfaction, and perceived usefulness, studies should adhere to conceptual conventions within the literature Avoid using proxy measures when assessing penetration and sustainability Standardized tools should be used in evaluating implementation and intervention fidelity, and in understanding how they relate to effectiveness of the VC intervention Adopt standardized definitions for adoption, appropriateness, feasibility, and fidelity	Formally evaluate acceptability, adoption, and appropriateness from the perspectives of clinicians and health systems Evaluate costs from the perspectives of patients and society Evaluation of implementation from the caregiver perspective is needed Comprehensive studies of the impact of VC on learners or trainees are needed Mixed methods studies are needed to understand factors influencing penetration More studies are needed on factors influencing long-term adoption of VC and on the impact of VC on climate change	A comprehensive conceptual framework of the impacts of context on VC implementation is needed to inform sustainability planning Standardized definitions for adoption, appropriateness, feasibility, and fidelity need to be developed
Service outcomes	Adhere to standardized definitions of equity-related concepts, such as gender versus sex, when undertaking evaluations Replace proxy measures with direct and objective or validated measures where possible For domains such as patient-centeredness for which validated measures are available but were underutilized, adopt these objective measures Adopt a standardized definition of safety in relation to VC	Additional studies exploring safety of VC from the patient perspective are warranted Additional equity research in VC, which captures the experiences of individuals with intersecting identities, is needed	Conceptualize theory related to disparities and equity considerations, specific to VC, with a focus on intersectionality Conceptualize the quality of clinical interactions as it relates to VC Standardize definition of safety Develop and validate objective measures of health equity considerations around VC Develop and validate an objective measure or evaluation methodology of cultural appropriateness as it relates to VC
Client outcomes	Replace proxy measures with widely accepted, validated measures to assess satisfaction, experience, and usability in VC. Use accepted clinical biomarkers, vitals, and laboratories as measures of clinical effectiveness wherever possible	Evaluate clinicians’ experiences with virtual care Examine factors associated with the usability of VC systems to inform the development of minimum system standards Ascertain which validated measures of satisfaction and experience are most appropriate or perform best when assessing VC Evaluate caregiver satisfaction and experiences with VC separate of patients	Conceptualize relationships between implementation contexts and client outcomes to inform the development of a framework for optimization of VC moving forward Work is needed to develop and validate an objective measure of patient, caregiver, and clinician perceptions and expectations around privacy and security of VC

The quality of evaluation of concepts within the implementation domain was particularly poor, as there appeared to be a lack of consensus on how to measure acceptability, adoption, feasibility, and fidelity, and poor adherence to conventional definitions of these concepts. This may stem from the lack of VC-specific validated measures, as many of the existing measures, including the Telemedicine Satisfaction and Acceptance Scale, Unified Theory of Acceptance, and Use of Technology Scale, combine different conceptual elements of usability and satisfaction with acceptance [20]. Existing organizations with an interest in quality measurement, such as the Agency for Healthcare Research and Quality, likely have a role to play in standardizing these definitions moving forward. Additionally, the development of a centralized repository of VC measures could be beneficial in improving reproducibility and comparability of findings of future VC evaluations.

### Service Outcomes

There are several existing, validated measures of patient-centeredness of care [21], including the Picker [22] and the English Person-Centered Climate Questionnaire-Patient version [23]; however, these measures appear to be underutilized, as none of the included articles reported on using objective, validated measures. Moving forward, research should leverage the existing validated tools when evaluating the impact of virtual visits on patient-centeredness of care.

Few review articles evaluated the impact of VC on health equity (28/507, 5.5%), with most (25/28, 89.3%) focusing on marginalized or vulnerable populations, especially Indigenous populations (8/25, 32.0%) and racialized populations (7/25, 28.0%). Despite growing concerns of the potential impacts of VC on health equity and broadening disparities in access to care [24-26], there was limited evaluation of specific inequities, such as sociodemographic status, language and accessibility, and intersecting identities. Additionally, articles that did examine disparities in care did not always adhere to standardized definitions of concepts, such as reporting on gender versus sex. Cultural appropriateness of VC, particularly around who is involved in or able to observe interactions, and patient preferences surrounding involvement of family and friends in treatment decision-making was highlighted by patients, clinicians, and stakeholders as a major barrier to VC; however, this concept did not appear to be formally evaluated within the current body of literature. Future evaluations should include examinations of cultural competency and cultural appropriateness of virtual visits and make use of existing measures and tools, such as the Consumer Assessment of Healthcare Providers and Systems cultural competence patient-facing questionnaire [27], the Multicultural Counseling Self Efficacy Scale—Racial Diversity Form [27], clinician-facing questionnaire [28], and the Cultural Safety in Health Systems framework to guide system-level evaluation [29]. Additionally, future work should focus on generating robust evidence through multimethod evaluations [30], which triangulate qualitative data on patient perspectives around disparities in their care [25] with objective evaluations of patterns of VC delivery [31,32].

### Client Outcomes

Studies evaluating clinical outcomes used accepted objective clinical biomarkers, vitals, and laboratory values for measures of clinical effectiveness wherever possible and adhered to accepted definitions of measures of mortality. However, appropriate comparator groups were not always used, and care was often hybrid rather than strictly in-person versus virtual, which presented additional challenges in evaluation. Future work should consider appropriate controls and comparators when evaluating clinical effectiveness, such as historical controls or contemporaneous, nonexposed comparators where appropriate.

VC often leverages caregivers as a care partner, providing technical support and enabling virtual interactions [33,34]; however, there was limited evaluation of the caregiver experience, satisfaction with and acceptance of VC as a unique stakeholder in VC delivery, beyond measures of caregiver burden, and impact on mental health. Additionally, clinician experience and acceptance of VC have implications for availability, adoption, and long-term sustainability of VC; however, relative to patients, few articles examined the clinician perspective. Future evaluation and research should include measures of the caregiver and clinician experiences with VC as a unique and pivotal perspective.

### Limitations

Our findings must be interpreted within the limitations of our methods. Given the substantial body of literature surrounding VC and the volume of literature reviews published on the topic, we chose to undertake a review of reviews [35]. This methodology afforded us the opportunity to systematically search across clinical conditions, as there have been many pockets of VC innovation globally, including delivery of specialist care for cardiology and dermatology, ongoing treatment of mental health conditions, and delivery of care to rural and remote communities, while still returning a feasible number of citations to abstract under reasonable timelines. However, this impacted the granularity of the abstraction, as it is dependent on the authors of reviews to abstract the primary articles accurately and may have led to underreporting of some VC measures. Additionally, our definition of VC was restricted to synchronous, outpatient interactions between clinicians and patients. While asynchronous forms of VC, such as secure messaging and text messaging, are gaining traction [36], we felt that this may have introduced too much heterogeneity in the aim of this study, and we did not feel there was sufficient evidence base to include them in a review of reviews at this time. Finally, we restricted our search to only full-text articles published in English for practical reasons. As most countries globally adopted VC in some capacity as part of their pandemic response [37], they may have missed some relevant citations, which were published in other languages.

### Conclusions

A better understanding of the short- and long-term impacts of virtual visits is needed to inform optimization of models of VC delivery, reimbursement structures, critical infrastructure investments, and health policy. Work is needed to standardize measure definitions as they relate to VC for key concepts particularly in the implementation domain. Standardization is pivotal for improving reproducibility, comparability, and generalizability of findings across different clinical settings, VC delivery modalities, and health systems, to drive future VC policy, clinical adoption, and patient engagement. Development of a centralized repository of VC measures could be beneficial in improving reproducibility and comparability of findings of future VC evaluations. Future studies should also include the caregiver and the clinician as unique perspectives in evaluations and should embed systematic evaluations of the impact of social determinants of health on VC access, adoption, and impressions of care using both qualitative and quantitative methodologies to get a more fulsome understanding of disparities.

## Supplementary material

10.2196/65312 Multimedia Appendix 1 Search strategies for all databases.

10.2196/65312 Multimedia Appendix 2 Details of retained articles.

10.2196/65312 Multimedia Appendix 3 Summary of measures from review articles by Proctor outcome domain and construct.

## Abbreviations

VC: virtual care
