# Supplementary material for: Virtual Care, What Are We Measuring and What Should We Measure? Scoping Review of Reviews
Source: J Med Internet Res. 2025 Dec 1;27:e65312. doi: 10.2196/65312 (PMC12670043; doi:10.2196/65312)
Supplement: Multimedia Appendix 1 [file jmir-v27-e65312-s001.docx]

**Multimedia Appendix 1.** Search strategies for all databases

**Ovid MEDLINE(R) ALL 2015 to May 10, 2023**

| **#** | **Searches** | **Results** | **Type** |  |  |  |
| --- | --- | --- | --- | --- | --- | --- |
|  | | | | | | |
| 1 | exp Telemedicine/ | 44246 | Advanced |  |  |  |
| 2 | Remote Consultation/ | 5694 | Advanced |  |  |  |
| 3 | Distance Counseling/ | 77 | Advanced |  |  |  |
| 4 | Internet-Based Intervention/ | 1111 | Advanced |  |  |  |
| 5 | virtual*.tw,kf. | 168542 | Advanced |  |  |  |
| 6 | (tele*med* or tele*-med*).tw,kf. | 25412 | Advanced |  |  |  |
| 7 | (tele*health* or tele*-health*).tw,kf. | 14341 | Advanced |  |  |  |
| 8 | (tele*interven* or (tele* adj3 interven*)).tw,kf. | 4196 | Advanced |  |  |  |
| 9 | (tele*therap* or (tele* adj3 therap*)).tw,kf. | 3872 | Advanced |  |  |  |
| 10 | (tele*treat* or (tele* adj3 treat*)).tw,kf. | 2066 | Advanced |  |  |  |
| 11 | (tele*surg* or (tele* adj3 surg*)).tw,kf. | 1925 | Advanced |  |  |  |
| 12 | (tele*diagnos* or (tele* adj3 diagnos*)).tw,kf. | 1721 | Advanced |  |  |  |
| 13 | (tele*rad* or (tele* adj3 rad*)).tw,kf. | 5800 | Advanced |  |  |  |
| 14 | (tele*visit* or (tele* adj3 visit*)).tw,kf. | 3520 | Advanced |  |  |  |
| 15 | (tele* adj3 ambulator*).tw,kf. | 179 | Advanced |  |  |  |
| 16 | (tele* adj3 urgent care?).tw,kf. | 48 | Advanced |  |  |  |
| 17 | (tele*rehab* or (tele* adj3 rehab*)).tw,kf. | 2410 | Advanced |  |  |  |
| 18 | (tele*clinic* or (tele* adj3 clinic*)).tw,kf. | 4177 | Advanced |  |  |  |
| 19 | (tele*care? or (tele* adj3 care?)).tw,kf. | 6817 | Advanced |  |  |  |
| 20 | (tele*navigat* or (tele* adj3 navigat*)).tw,kf. | 145 | Advanced |  |  |  |
| 21 | (tele*consult* or (tele* adj3 consult*)).tw,kf. | 5835 | Advanced |  |  |  |
| 22 | (tele*counsel* or (tele* adj3 counsel*)).tw,kf. | 1936 | Advanced |  |  |  |
| 23 | (tele* adj3 (aftercare? or after care?)).tw,kf. | 50 | Advanced |  |  |  |
| 24 | (tele*service? or (tele* adj3 service?)).tw,kf. | 6935 | Advanced |  |  |  |
| 25 | (tele*follow* up? or (tele* adj3 follow* up?)).tw,kf. | 6449 | Advanced |  |  |  |
| 26 | (mobile* adj3 health*).tw,kf. | 10279 | Advanced |  |  |  |
| 27 | (mobile* adj3 interven*).tw,kf. | 2790 | Advanced |  |  |  |
| 28 | (mobile* adj3 therap*).tw,kf. | 295 | Advanced |  |  |  |
| 29 | (mobile* adj3 treat*).tw,kf. | 744 | Advanced |  |  |  |
| 30 | (mobile* adj3 surg*).tw,kf. | 394 | Advanced |  |  |  |
| 31 | (mobile* adj3 diagnos*).tw,kf. | 336 | Advanced |  |  |  |
| 32 | (mobile* adj3 rad*).tw,kf. | 1089 | Advanced |  |  |  |
| 33 | (mobile* adj3 visit*).tw,kf. | 209 | Advanced |  |  |  |
| 34 | (mobile* adj3 ambulator*).tw,kf. | 39 | Advanced |  |  |  |
| 35 | (mobile* adj3 urgent care?).tw,kf. | 5 | Advanced |  |  |  |
| 36 | (mobile* adj3 rehab*).tw,kf. | 142 | Advanced |  |  |  |
| 37 | (mobile* adj3 clinic*).tw,kf. | 1665 | Advanced |  |  |  |
| 38 | (mobile* adj3 care?).tw,kf. | 1751 | Advanced |  |  |  |
| 39 | (mobile* adj3 navigat*).tw,kf. | 210 | Advanced |  |  |  |
| 40 | (mobile* adj3 consult*).tw,kf. | 101 | Advanced |  |  |  |
| 41 | (mobile* adj3 counsel*).tw,kf. | 124 | Advanced |  |  |  |
| 42 | (mobile* adj3 (aftercare? or after care?)).tw,kf. | 11 | Advanced |  |  |  |
| 43 | (mobile* adj3 service?).tw,kf. | 1550 | Advanced |  |  |  |
| 44 | (mobile* adj3 follow* up?).tw,kf. | 138 | Advanced |  |  |  |
| 45 | ((online? or on-line?) adj3 health*).tw,kf. | 5468 | Advanced |  |  |  |
| 46 | ((online? or on-line?) adj3 interven*).tw,kf. | 3199 | Advanced |  |  |  |
| 47 | ((online? or on-line?) adj3 therap*).tw,kf. | 1112 | Advanced |  |  |  |
| 48 | ((online? or on-line?) adj3 treat*).tw,kf. | 1318 | Advanced |  |  |  |
| 49 | ((online? or on-line?) adj3 surg*).tw,kf. | 537 | Advanced |  |  |  |
| 50 | ((online? or on-line?) adj3 diagnos*).tw,kf. | 497 | Advanced |  |  |  |
| 51 | ((online? or on-line?) adj3 rad*).tw,kf. | 945 | Advanced |  |  |  |
| 52 | ((online? or on-line?) adj3 visit*).tw,kf. | 611 | Advanced |  |  |  |
| 53 | ((online? or on-line?) adj3 ambulator*).tw,kf. | 14 | Advanced |  |  |  |
| 54 | ((online? or on-line?) adj3 urgent care?).tw,kf. | 2 | Advanced |  |  |  |
| 55 | ((online? or on-line?) adj3 rehab*).tw,kf. | 140 | Advanced |  |  |  |
| 56 | ((online? or on-line?) adj3 clinic*).tw,kf. | 1973 | Advanced |  |  |  |
| 57 | ((online? or on-line?) adj3 care?).tw,kf. | 1137 | Advanced |  |  |  |
| 58 | ((online? or on-line?) adj3 navigat*).tw,kf. | 153 | Advanced |  |  |  |
| 59 | ((online? or on-line?) adj3 consult*).tw,kf. | 791 | Advanced |  |  |  |
| 60 | ((online? or on-line?) adj3 counsel*).tw,kf. | 327 | Advanced |  |  |  |
| 61 | ((online? or on-line?) adj3 (aftercare? or after care?)).tw,kf. | 16 | Advanced |  |  |  |
| 62 | ((online? or on-line?) adj3 service?).tw,kf. | 2070 | Advanced |  |  |  |
| 63 | ((online? or on-line?) adj3 follow* up?).tw,kf. | 403 | Advanced |  |  |  |
| 64 | (remote* adj3 health*).tw,kf. | 2694 | Advanced |  |  |  |
| 65 | (remote* adj3 interven*).tw,kf. | 874 | Advanced |  |  |  |
| 66 | (remote* adj3 therap*).tw,kf. | 661 | Advanced |  |  |  |
| 67 | (remote* adj3 treat*).tw,kf. | 1215 | Advanced |  |  |  |
| 68 | (remote* adj3 surg*).tw,kf. | 1237 | Advanced |  |  |  |
| 69 | (remote* adj3 diagnos*).tw,kf. | 929 | Advanced |  |  |  |
| 70 | (remote* adj3 rad*).tw,kf. | 821 | Advanced |  |  |  |
| 71 | (remote* adj3 visit*).tw,kf. | 358 | Advanced |  |  |  |
| 72 | (remote* adj3 ambulator*).tw,kf. | 57 | Advanced |  |  |  |
| 73 | (remote* adj3 urgent care?).tw,kf. | 4 | Advanced |  |  |  |
| 74 | (remote* adj3 rehab*).tw,kf. | 285 | Advanced |  |  |  |
| 75 | (remote* adj3 clinic*).tw,kf. | 1518 | Advanced |  |  |  |
| 76 | (remote* adj3 care?).tw,kf. | 2256 | Advanced |  |  |  |
| 77 | (remote* adj3 navigat*).tw,kf. | 334 | Advanced |  |  |  |
| 78 | (remote* adj3 consult*).tw,kf. | 1445 | Advanced |  |  |  |
| 79 | (remote* adj3 counsel*).tw,kf. | 113 | Advanced |  |  |  |
| 80 | (remote* adj3 (aftercare? or after care?)).tw,kf. | 1 | Advanced |  |  |  |
| 81 | (remote* adj3 service?).tw,kf. | 1586 | Advanced |  |  |  |
| 82 | (remote* adj3 follow* up?).tw,kf. | 480 | Advanced |  |  |  |
| 83 | (ehealth* or e-health*).tw,kf. | 10959 | Advanced |  |  |  |
| 84 | (mhealth* or m-health*).tw,kf. | 9962 | Advanced |  |  |  |
| 85 | (thealth* or t-health*).tw,kf. | 31 | Advanced |  |  |  |
| 86 | (uhealth* or u-health*).tw,kf. | 89 | Advanced |  |  |  |
| 87 | (vhealth* or v-health*).tw,kf. | 98 | Advanced |  |  |  |
| 88 | or/1-87 | 291807 | Advanced |  |  |  |
| 89 | "Quality of Health Care"/ | 77360 | Advanced |  |  |  |
| 90 | Quality Assurance, Health Care/ | 56880 | Advanced |  |  |  |
| 91 | Quality Improvement/ | 32316 | Advanced |  |  |  |
| 92 | exp Quality Indicators, Health Care/ | 24794 | Advanced |  |  |  |
| 93 | Total Quality Management/ | 12732 | Advanced |  |  |  |
| 94 | "United States Agency for Healthcare Research and Quality"/ | 1653 | Advanced |  |  |  |
| 95 | exp "Costs and Cost Analysis"/ | 264223 | Advanced |  |  |  |
| 96 | Cost Allocation/ | 2018 | Advanced |  |  |  |
| 97 | Cost-Benefit Analysis/ | 92303 | Advanced |  |  |  |
| 98 | exp Cost Control/ | 34139 | Advanced |  |  |  |
| 99 | Cost-Effectiveness Analysis/ | 280 | Advanced |  |  |  |
| 100 | Health Care Costs/ | 44064 | Advanced |  |  |  |
| 101 | Direct Service Costs/ | 1217 | Advanced |  |  |  |
| 102 | Technology/ | 17471 | Advanced |  |  |  |
| 103 | Wireless Technology/ | 4642 | Advanced |  |  |  |
| 104 | "Facilities and Services Utilization"/ | 1268 | Advanced |  |  |  |
| 105 | Patient Reported Outcome Measures/ | 13311 | Advanced |  |  |  |
| 106 | Program Evaluation/ | 67150 | Advanced |  |  |  |
| 107 | Evaluation Study/ | 261881 | Advanced |  |  |  |
| 108 | Health Equity/ | 3475 | Advanced |  |  |  |
| 109 | exp Health Inequities/ | 19896 | Advanced |  |  |  |
| 110 | Healthcare Disparities/ | 21994 | Advanced |  |  |  |
| 111 | Health Status Disparities/ | 19547 | Advanced |  |  |  |
| 112 | "Health Disparate, Minority and Vulnerable Populations"/ | 13 | Advanced |  |  |  |
| 113 | Socioeconomic Disparities in Health/ | 57 | Advanced |  |  |  |
| 114 | "Social Determinants of Health"/ | 6316 | Advanced |  |  |  |
| 115 | exp Social Class/ | 45304 | Advanced |  |  |  |
| 116 | exp Patient Satisfaction/ | 99205 | Advanced |  |  |  |
| 117 | Patient Preference/ | 10619 | Advanced |  |  |  |
| 118 | Patient Safety/ | 25319 | Advanced |  |  |  |
| 119 | Safety/ | 41920 | Advanced |  |  |  |
| 120 | Safety Management/ | 21418 | Advanced |  |  |  |
| 121 | (qualit* adj3 health*).tw,kf. | 118141 | Advanced |  |  |  |
| 122 | (qualit* adj3 care?).tw,kf. | 102902 | Advanced |  |  |  |
| 123 | (qualit* adj3 assuran*).tw,kf. | 30585 | Advanced |  |  |  |
| 124 | (qualit* adj3 assess*).tw,kf. | 113565 | Advanced |  |  |  |
| 125 | (qualit* adj3 improv*).tw,kf. | 211852 | Advanced |  |  |  |
| 126 | (qualit* adj3 indicat*).tw,kf. | 27368 | Advanced |  |  |  |
| 127 | (qualit* adj3 metric?).tw,kf. | 5960 | Advanced |  |  |  |
| 128 | (qualit* adj3 measur*).tw,kf. | 51257 | Advanced |  |  |  |
| 129 | (qualit* adj3 manag*).tw,kf. | 16779 | Advanced |  |  |  |
| 130 | (qualit* adj3 evaluat*).tw,kf. | 60280 | Advanced |  |  |  |
| 131 | (quality adj3 outcome?).tw,kf. | 27986 | Advanced |  |  |  |
| 132 | (quality adj3 (framework? or frame-work?)).tw,kf. | 2507 | Advanced |  |  |  |
| 133 | (quality adj3 tool?).tw,kf. | 9245 | Advanced |  |  |  |
| 134 | (cost?? or costing).tw,kf. | 746618 | Advanced |  |  |  |
| 135 | (service? adj3 utili*).tw,kf. | 18355 | Advanced |  |  |  |
| 136 | (service? adj3 evaluat*).tw,kf. | 9720 | Advanced |  |  |  |
| 137 | (resource? adj3 utili*).tw,kf. | 21753 | Advanced |  |  |  |
| 138 | (resource? adj3 evaluat*).tw,kf. | 3297 | Advanced |  |  |  |
| 139 | (process* adj3 measur*).tw,kf. | 18790 | Advanced |  |  |  |
| 140 | (process* adj3 evaluat*).tw,kf. | 27815 | Advanced |  |  |  |
| 141 | (technolog* adj3 access*).tw,kf. | 4019 | Advanced |  |  |  |
| 142 | (technolog* adj3 limit*).tw,kf. | 5185 | Advanced |  |  |  |
| 143 | (effective* adj3 evaluat*).tw,kf. | 73324 | Advanced |  |  |  |
| 144 | (outcome? adj3 evaluat*).tw,kf. | 74689 | Advanced |  |  |  |
| 145 | (care? adj3 evaluat*).tw,kf. | 17008 | Advanced |  |  |  |
| 146 | ((framework? or frame-work?) adj3 evaluat*).tw,kf. | 8264 | Advanced |  |  |  |
| 147 | (integrat* adj3 evaluat*).tw,kf. | 5158 | Advanced |  |  |  |
| 148 | evaluation?.tw,kf. | 1526188 | Advanced |  |  |  |
| 149 | (report* adj3 outcome?).tw,kf. | 90513 | Advanced |  |  |  |
| 150 | PROM?.tw,kf. | 8559 | Advanced |  |  |  |
| 151 | equit*.tw,kf. | 44424 | Advanced |  |  |  |
| 152 | inequit*.tw,kf. | 19886 | Advanced |  |  |  |
| 153 | equalit*.tw,kf. | 11530 | Advanced |  |  |  |
| 154 | inequalit*.tw,kf. | 46431 | Advanced |  |  |  |
| 155 | dispar*.tw,kf. | 125580 | Advanced |  |  |  |
| 156 | "social* determin* of health?".tw,kf. | 11586 | Advanced |  |  |  |
| 157 | (social* adj3 gradient*).tw,kf. | 1326 | Advanced |  |  |  |
| 158 | satisf*.tw,kf. | 416056 | Advanced |  |  |  |
| 159 | experienc*.tw,kf. | 1394408 | Advanced |  |  |  |
| 160 | safet*.tw,kf. | 683483 | Advanced |  |  |  |
| 161 | or/89-160 | 5333006 | Advanced |  |  |  |
| 162 | 88 and 161 | 109422 | Advanced |  |  |  |
| 163 | review/ | 3147889 | Advanced |  |  |  |
| 164 | (medline or medlars or pubmed or grateful med or CINAHL or scisearch or psychinfo or psycinfo or psychlit or psyclit or handsearch* or hand search* or manual* search* or electronic database* or bibliographic database* or embase or lilacs or scopus or web of science).mp,kw. | 380748 | Advanced |  |  |  |
| 165 | 163 and 164 | 201723 | Advanced |  |  |  |
| 166 | "systematic review"/ | 227939 | Advanced |  |  |  |
| 167 | systematic reviews as topic/ | 10472 | Advanced |  |  |  |
| 168 | "scientific integrity review"/ | 280 | Advanced |  |  |  |
| 169 | meta-analysis/ | 180546 | Advanced |  |  |  |
| 170 | exp meta-analysis as topic/ | 26922 | Advanced |  |  |  |
| 171 | network meta-analysis/ | 4835 | Advanced |  |  |  |
| 172 | exp technology assessment, biomedical/ | 12113 | Advanced |  |  |  |
| 173 | technology, high-cost/ | 1208 | Advanced |  |  |  |
| 174 | "systematic review".pt. | 227939 | Advanced |  |  |  |
| 175 | "scientific integrity review".pt. | 280 | Advanced |  |  |  |
| 176 | meta-analysis.pt. | 180546 | Advanced |  |  |  |
| 177 | (cochrane or (health adj2 technology assessment) or evidence report).jw. | 21349 | Advanced |  |  |  |
| 178 | (quantitative* adj3 (synthes* or review? or overview?)).tw,kf. | 8365 | Advanced |  |  |  |
| 179 | (qualitative* adj3 (synthes* or review? or overview?)).tw,kf. | 13011 | Advanced |  |  |  |
| 180 | (knowledge adj3 synthes*).tw,kf. | 2311 | Advanced |  |  |  |
| 181 | (systematic* adj3 (synthes* or review? or overview? or study or studies)).tw,kf. | 343194 | Advanced |  |  |  |
| 182 | (scoping adj3 (synthes* or review? or overview? or study or studies)).tw,kf. | 20840 | Advanced |  |  |  |
| 183 | (mapping adj3 (synthes* or review? or overview? or study or studies)).tw,kf. | 8973 | Advanced |  |  |  |
| 184 | (rapid adj3 (synthes* or review? or overview? or study or studies)).tw,kf. | 13646 | Advanced |  |  |  |
| 185 | (umbrella adj3 (synthes* or review? or overview? or study or studies)).tw,kf. | 1593 | Advanced |  |  |  |
| 186 | ("review of review?" or "review of systematic review?").tw,kf. | 10331 | Advanced |  |  |  |
| 187 | ("overview? of review?" or "overview? of systematic review?").tw,kf. | 1462 | Advanced |  |  |  |
| 188 | ("summar* of review?" or "summar* of systematic review?").tw,kf. | 3582 | Advanced |  |  |  |
| 189 | ("synthes* of review?" or "synthes* of systematic review?").tw,kf. | 1364 | Advanced |  |  |  |
| 190 | (integrative* adj3 (synthes* or review? or overview? or study or studies)).tw,kf. | 7923 | Advanced |  |  |  |
| 191 | (narrative adj3 (synthes* or review? or overview? or study or studies)).tw,kf. | 36392 | Advanced |  |  |  |
| 192 | (methodologic* adj3 (synthes* or review? or overview? or study or studies)).tw,kf. | 18796 | Advanced |  |  |  |
| 193 | (methodologic* adj3 (synthes* or review? or overview? or study or studies)).tw,kf. | 18796 | Advanced |  |  |  |
| 194 | (research adj3 (synthes* or review? or overview? or study or studies)).tw,kf. | 112062 | Advanced |  |  |  |
| 195 | (collaborative* adj3 (synthes* or review? or overview? or study or studies)).tw,kf. | 11939 | Advanced |  |  |  |
| 196 | (metaanal* or metanal* or (meta adj2 anal*)).tw,kf. | 272026 | Advanced |  |  |  |
| 197 | (metasummar* or (meta adj2 summar*)).tw,kf. | 1464 | Advanced |  |  |  |
| 198 | (metasynthes* or (meta adj2 synthes*)).tw,kf. | 3875 | Advanced |  |  |  |
| 199 | (metareview* or (meta adj2 review*)).tw,kf. | 119427 | Advanced |  |  |  |
| 200 | (metanetwork* or (meta adj2 network*)).tw,kf. | 9065 | Advanced |  |  |  |
| 201 | (metaaggregat* or (meta adj2 aggregat*)).tw,kf. | 629 | Advanced |  |  |  |
| 202 | (metaregression* or (meta adj2 regression*)).tw,kf. | 14515 | Advanced |  |  |  |
| 203 | (metaethnograph* or (meta adj2 ethnograph*)).tw,kf. | 860 | Advanced |  |  |  |
| 204 | (metanarrative* or (meta adj2 narrative*)).tw,kf. | 481 | Advanced |  |  |  |
| 205 | (pooled adj1 anal*).tw,kf. | 15968 | Advanced |  |  |  |
| 206 | (statistical* adj1 pooling).tw,kf. | 439 | Advanced |  |  |  |
| 207 | (statistical* adj1 summar*).tw,kf. | 532 | Advanced |  |  |  |
| 208 | (mathematical* adj1 pooling).tw,kf. | 2 | Advanced |  |  |  |
| 209 | (mathematical* adj1 summar*).tw,kf. | 30 | Advanced |  |  |  |
| 210 | (data adj3 (synthes* or extraction* or abstraction*)).tw,kf. | 52125 | Advanced |  |  |  |
| 211 | (mantel haenszel or peto or der simonian or dersimonian or fixed effect* or latin square*).tw,kf. | 35232 | Advanced |  |  |  |
| 212 | (comparative adj3 (efficacy or effectiveness)).tw,kf. | 17385 | Advanced |  |  |  |
| 213 | (outcomes research or relative effectiveness).tw,kf. | 11159 | Advanced |  |  |  |
| 214 | ((indirect or indirect treatment?) adj3 comparison*).tw,kf. | 3366 | Advanced |  |  |  |
| 215 | (mixed adj3 treatment? adj3 comparison*).tw,kf. | 545 | Advanced |  |  |  |
| 216 | (multi* adj3 treatment? adj3 comparison*).tw,kf. | 369 | Advanced |  |  |  |
| 217 | (bayesian adj3 comparison*).tw,kf. | 646 | Advanced |  |  |  |
| 218 | (multi* adj2 paramet* adj2 evidence adj2 synthesis).tw,kf. | 14 | Advanced |  |  |  |
| 219 | ((multiparamet* or multi paramet*) adj2 evidence adj2 synthesis).tw,kf. | 30 | Advanced |  |  |  |
| 220 | (handsearch* or hand search*).tw,kf. | 11074 | Advanced |  |  |  |
| 221 | (technology assessment* or technology overview* or technology appraisal*).tw,kf. | 9435 | Advanced |  |  |  |
| 222 | (HTA or HTAs).tw,kf. | 4195 | Advanced |  |  |  |
| 223 | or/165-222 | 848808 | Advanced |  |  |  |
| 224 | 162 and 223 | 10229 | Advanced |  |  |  |
| 225 | (virtual* adj10 care?).tw,kf. | 4174 | Advanced |  |  |  |
| 226 | 223 and 225 | 394 | Advanced |  |  |  |
| 227 | 224 or 226 | 10343 | Advanced |  |  |  |
| 228 | exp animals/ not (exp animals/ and exp humans/) | 5119747 | Advanced |  |  |  |
| 229 | 227 not 228 | 10311 | Advanced |  |  |  |
| 230 | limit 229 to yr="2015 -Current" | 8347 | Advanced |  |  |  |
| 231 | limit 230 to english language | 8218 | Advanced |  |  |  |

**Ovid Embase 2015 to 2023 May 10**

| **#** | **Searches** | **Results** | **Type** |  |  |  |
| --- | --- | --- | --- | --- | --- | --- |
|  | | | | | | |
| 1 | exp telehealth/ | 85711 | Advanced |  |  |  |
| 2 | exp telemedicine/ | 70046 | Advanced |  |  |  |
| 3 | telecare/ | 985 | Advanced |  |  |  |
| 4 | exp teleconsultation/ | 15586 | Advanced |  |  |  |
| 5 | electronic consultation/ | 462 | Advanced |  |  |  |
| 6 | e-counseling/ | 451 | Advanced |  |  |  |
| 7 | web-based intervention/ | 2634 | Advanced |  |  |  |
| 8 | virtual*.tw,kf. | 212951 | Advanced |  |  |  |
| 9 | (tele*med* or tele*-med*).tw,kf. | 35128 | Advanced |  |  |  |
| 10 | (tele*health* or tele*-health*).tw,kf. | 18801 | Advanced |  |  |  |
| 11 | (tele*interven* or (tele* adj3 interven*)).tw,kf. | 5724 | Advanced |  |  |  |
| 12 | (tele*therap* or (tele* adj3 therap*)).tw,kf. | 3657 | Advanced |  |  |  |
| 13 | (tele*treat* or (tele* adj3 treat*)).tw,kf. | 2953 | Advanced |  |  |  |
| 14 | (tele*surg* or (tele* adj3 surg*)).tw,kf. | 2583 | Advanced |  |  |  |
| 15 | (tele*diagnos* or (tele* adj3 diagnos*)).tw,kf. | 2454 | Advanced |  |  |  |
| 16 | (tele*rad* or (tele* adj3 rad*)).tw,kf. | 6069 | Advanced |  |  |  |
| 17 | (tele*visit* or (tele* adj3 visit*)).tw,kf. | 6392 | Advanced |  |  |  |
| 18 | (tele* adj3 ambulator*).tw,kf. | 274 | Advanced |  |  |  |
| 19 | (tele* adj3 urgent care?).tw,kf. | 62 | Advanced |  |  |  |
| 20 | (tele*rehab* or (tele* adj3 rehab*)).tw,kf. | 3050 | Advanced |  |  |  |
| 21 | (tele*clinic* or (tele* adj3 clinic*)).tw,kf. | 6989 | Advanced |  |  |  |
| 22 | (tele*care? or (tele* adj3 care?)).tw,kf. | 9558 | Advanced |  |  |  |
| 23 | (tele*navigat* or (tele* adj3 navigat*)).tw,kf. | 209 | Advanced |  |  |  |
| 24 | (tele*consult* or (tele* adj3 consult*)).tw,kf. | 9143 | Advanced |  |  |  |
| 25 | (tele*counsel* or (tele* adj3 counsel*)).tw,kf. | 2668 | Advanced |  |  |  |
| 26 | (tele* adj3 (aftercare? or after care?)).tw,kf. | 72 | Advanced |  |  |  |
| 27 | (tele*service? or (tele* adj3 service?)).tw,kf. | 9538 | Advanced |  |  |  |
| 28 | (tele*follow* up? or (tele* adj3 follow* up?)).tw,kf. | 10481 | Advanced |  |  |  |
| 29 | (mobile* adj3 health*).tw,kf. | 10723 | Advanced |  |  |  |
| 30 | (mobile* adj3 interven*).tw,kf. | 2899 | Advanced |  |  |  |
| 31 | (mobile* adj3 therap*).tw,kf. | 426 | Advanced |  |  |  |
| 32 | (mobile* adj3 treat*).tw,kf. | 1045 | Advanced |  |  |  |
| 33 | (mobile* adj3 surg*).tw,kf. | 505 | Advanced |  |  |  |
| 34 | (mobile* adj3 diagnos*).tw,kf. | 465 | Advanced |  |  |  |
| 35 | (mobile* adj3 rad*).tw,kf. | 1407 | Advanced |  |  |  |
| 36 | (mobile* adj3 visit*).tw,kf. | 284 | Advanced |  |  |  |
| 37 | (mobile* adj3 ambulator*).tw,kf. | 55 | Advanced |  |  |  |
| 38 | (mobile* adj3 urgent care?).tw,kf. | 6 | Advanced |  |  |  |
| 39 | (mobile* adj3 rehab*).tw,kf. | 196 | Advanced |  |  |  |
| 40 | (mobile* adj3 clinic*).tw,kf. | 2137 | Advanced |  |  |  |
| 41 | (mobile* adj3 care?).tw,kf. | 2294 | Advanced |  |  |  |
| 42 | (mobile* adj3 navigat*).tw,kf. | 230 | Advanced |  |  |  |
| 43 | (mobile* adj3 consult*).tw,kf. | 166 | Advanced |  |  |  |
| 44 | (mobile* adj3 counsel*).tw,kf. | 152 | Advanced |  |  |  |
| 45 | (mobile* adj3 (aftercare? or after care?)).tw,kf. | 15 | Advanced |  |  |  |
| 46 | (mobile* adj3 service?).tw,kf. | 1950 | Advanced |  |  |  |
| 47 | (mobile* adj3 follow* up?).tw,kf. | 209 | Advanced |  |  |  |
| 48 | ((online? or on-line?) adj3 health*).tw,kf. | 6785 | Advanced |  |  |  |
| 49 | ((online? or on-line?) adj3 interven*).tw,kf. | 4070 | Advanced |  |  |  |
| 50 | ((online? or on-line?) adj3 therap*).tw,kf. | 1794 | Advanced |  |  |  |
| 51 | ((online? or on-line?) adj3 treat*).tw,kf. | 2321 | Advanced |  |  |  |
| 52 | ((online? or on-line?) adj3 surg*).tw,kf. | 871 | Advanced |  |  |  |
| 53 | ((online? or on-line?) adj3 diagnos*).tw,kf. | 680 | Advanced |  |  |  |
| 54 | ((online? or on-line?) adj3 rad*).tw,kf. | 1620 | Advanced |  |  |  |
| 55 | ((online? or on-line?) adj3 visit*).tw,kf. | 530 | Advanced |  |  |  |
| 56 | ((online? or on-line?) adj3 ambulator*).tw,kf. | 28 | Advanced |  |  |  |
| 57 | ((online? or on-line?) adj3 urgent care?).tw,kf. | 3 | Advanced |  |  |  |
| 58 | ((online? or on-line?) adj3 rehab*).tw,kf. | 216 | Advanced |  |  |  |
| 59 | ((online? or on-line?) adj3 clinic*).tw,kf. | 3340 | Advanced |  |  |  |
| 60 | ((online? or on-line?) adj3 care?).tw,kf. | 1761 | Advanced |  |  |  |
| 61 | ((online? or on-line?) adj3 navigat*).tw,kf. | 200 | Advanced |  |  |  |
| 62 | ((online? or on-line?) adj3 consult*).tw,kf. | 1251 | Advanced |  |  |  |
| 63 | ((online? or on-line?) adj3 counsel*).tw,kf. | 447 | Advanced |  |  |  |
| 64 | ((online? or on-line?) adj3 (aftercare? or after care?)).tw,kf. | 19 | Advanced |  |  |  |
| 65 | ((online? or on-line?) adj3 service?).tw,kf. | 2878 | Advanced |  |  |  |
| 66 | ((online? or on-line?) adj3 follow* up?).tw,kf. | 624 | Advanced |  |  |  |
| 67 | (remote* adj3 health*).tw,kf. | 3350 | Advanced |  |  |  |
| 68 | (remote* adj3 interven*).tw,kf. | 1168 | Advanced |  |  |  |
| 69 | (remote* adj3 therap*).tw,kf. | 875 | Advanced |  |  |  |
| 70 | (remote* adj3 treat*).tw,kf. | 1542 | Advanced |  |  |  |
| 71 | (remote* adj3 surg*).tw,kf. | 1324 | Advanced |  |  |  |
| 72 | (remote* adj3 diagnos*).tw,kf. | 1309 | Advanced |  |  |  |
| 73 | (remote* adj3 rad*).tw,kf. | 942 | Advanced |  |  |  |
| 74 | (remote* adj3 visit*).tw,kf. | 673 | Advanced |  |  |  |
| 75 | (remote* adj3 ambulator*).tw,kf. | 83 | Advanced |  |  |  |
| 76 | (remote* adj3 urgent care?).tw,kf. | 8 | Advanced |  |  |  |
| 77 | (remote* adj3 rehab*).tw,kf. | 378 | Advanced |  |  |  |
| 78 | (remote* adj3 clinic*).tw,kf. | 2437 | Advanced |  |  |  |
| 79 | (remote* adj3 care?).tw,kf. | 3046 | Advanced |  |  |  |
| 80 | (remote* adj3 navigat*).tw,kf. | 660 | Advanced |  |  |  |
| 81 | (remote* adj3 consult*).tw,kf. | 2145 | Advanced |  |  |  |
| 82 | (remote* adj3 counsel*).tw,kf. | 193 | Advanced |  |  |  |
| 83 | (remote* adj3 (aftercare? or after care?)).tw,kf. | 3 | Advanced |  |  |  |
| 84 | (remote* adj3 service?).tw,kf. | 2110 | Advanced |  |  |  |
| 85 | (remote* adj3 follow* up?).tw,kf. | 922 | Advanced |  |  |  |
| 86 | (ehealth* or e-health*).tw,kf. | 12562 | Advanced |  |  |  |
| 87 | (mhealth* or m-health*).tw,kf. | 9720 | Advanced |  |  |  |
| 88 | (thealth* or t-health*).tw,kf. | 46 | Advanced |  |  |  |
| 89 | (uhealth* or u-health*).tw,kf. | 161 | Advanced |  |  |  |
| 90 | (vhealth* or v-health*).tw,kf. | 132 | Advanced |  |  |  |
| 91 | or/1-90 | 387452 | Advanced |  |  |  |
| 92 | health care quality/ | 271063 | Advanced |  |  |  |
| 93 | performance measurement system/ | 4354 | Advanced |  |  |  |
| 94 | "healthcare access and quality index"/ | 60 | Advanced |  |  |  |
| 95 | performance indicator/ | 2557 | Advanced |  |  |  |
| 96 | quality control/ | 232692 | Advanced |  |  |  |
| 97 | quality control procedures/ | 2268 | Advanced |  |  |  |
| 98 | total quality management/ | 90171 | Advanced |  |  |  |
| 99 | quality improvement study/ | 1498 | Advanced |  |  |  |
| 100 | quality by design/ | 1357 | Advanced |  |  |  |
| 101 | "cost"/ | 62414 | Advanced |  |  |  |
| 102 | exp "health care cost"/ | 336162 | Advanced |  |  |  |
| 103 | health care financing/ | 13855 | Advanced |  |  |  |
| 104 | health economics/ | 35567 | Advanced |  |  |  |
| 105 | "cost benefit analysis"/ | 93828 | Advanced |  |  |  |
| 106 | "cost control"/ | 75929 | Advanced |  |  |  |
| 107 | "cost effectiveness analysis"/ | 179911 | Advanced |  |  |  |
| 108 | technology/ | 110928 | Advanced |  |  |  |
| 109 | digital technology/ | 3687 | Advanced |  |  |  |
| 110 | wireless communication/ | 7422 | Advanced |  |  |  |
| 111 | exp health care utilization/ | 93317 | Advanced |  |  |  |
| 112 | "facilities and services utilization"/ | 64 | Advanced |  |  |  |
| 113 | "procedures and techniques utilization"/ | 113 | Advanced |  |  |  |
| 114 | patient-reported outcome/ | 52446 | Advanced |  |  |  |
| 115 | exp program evaluation/ | 34423 | Advanced |  |  |  |
| 116 | "program cost effectiveness"/ | 1025 | Advanced |  |  |  |
| 117 | evaluation study/ | 59836 | Advanced |  |  |  |
| 118 | health equity/ | 9694 | Advanced |  |  |  |
| 119 | health disparity/ | 33423 | Advanced |  |  |  |
| 120 | health care disparity/ | 21899 | Advanced |  |  |  |
| 121 | social inequality/ | 1249 | Advanced |  |  |  |
| 122 | exp socioeconomic vulnerability/ | 711 | Advanced |  |  |  |
| 123 | "social determinants of health"/ | 18829 | Advanced |  |  |  |
| 124 | social class/ | 33778 | Advanced |  |  |  |
| 125 | patient satisfaction/ | 167350 | Advanced |  |  |  |
| 126 | patient preference/ | 26102 | Advanced |  |  |  |
| 127 | patient safety/ | 148678 | Advanced |  |  |  |
| 128 | patient safety indicator/ | 77 | Advanced |  |  |  |
| 129 | safety/ | 263819 | Advanced |  |  |  |
| 130 | (qualit* adj3 health*).tw,kf. | 165769 | Advanced |  |  |  |
| 131 | (qualit* adj3 care?).tw,kf. | 142433 | Advanced |  |  |  |
| 132 | (qualit* adj3 assuran*).tw,kf. | 48229 | Advanced |  |  |  |
| 133 | (qualit* adj3 assess*).tw,kf. | 155950 | Advanced |  |  |  |
| 134 | (qualit* adj3 improv*).tw,kf. | 317696 | Advanced |  |  |  |
| 135 | (qualit* adj3 indicat*).tw,kf. | 39501 | Advanced |  |  |  |
| 136 | (qualit* adj3 metric?).tw,kf. | 10288 | Advanced |  |  |  |
| 137 | (qualit* adj3 measur*).tw,kf. | 72947 | Advanced |  |  |  |
| 138 | (qualit* adj3 manag*).tw,kf. | 26193 | Advanced |  |  |  |
| 139 | (qualit* adj3 evaluat*).tw,kf. | 82847 | Advanced |  |  |  |
| 140 | (quality adj3 outcome?).tw,kf. | 41941 | Advanced |  |  |  |
| 141 | (quality adj3 (framework? or frame-work?)).tw,kf. | 3540 | Advanced |  |  |  |
| 142 | (quality adj3 tool?).tw,kf. | 12984 | Advanced |  |  |  |
| 143 | (cost?? or costing).tw,kf. | 1009898 | Advanced |  |  |  |
| 144 | (service? adj3 utili*).tw,kf. | 24019 | Advanced |  |  |  |
| 145 | (service? adj3 evaluat*).tw,kf. | 15037 | Advanced |  |  |  |
| 146 | (resource? adj3 utili*).tw,kf. | 38163 | Advanced |  |  |  |
| 147 | (resource? adj3 evaluat*).tw,kf. | 4806 | Advanced |  |  |  |
| 148 | (process* adj3 measur*).tw,kf. | 24075 | Advanced |  |  |  |
| 149 | (process* adj3 evaluat*).tw,kf. | 36695 | Advanced |  |  |  |
| 150 | (technolog* adj3 access*).tw,kf. | 5426 | Advanced |  |  |  |
| 151 | (technolog* adj3 limit*).tw,kf. | 6530 | Advanced |  |  |  |
| 152 | (effective* adj3 evaluat*).tw,kf. | 103642 | Advanced |  |  |  |
| 153 | (outcome? adj3 evaluat*).tw,kf. | 119182 | Advanced |  |  |  |
| 154 | (care? adj3 evaluat*).tw,kf. | 24716 | Advanced |  |  |  |
| 155 | ((framework? or frame-work?) adj3 evaluat*).tw,kf. | 9610 | Advanced |  |  |  |
| 156 | (integrat* adj3 evaluat*).tw,kf. | 6911 | Advanced |  |  |  |
| 157 | evaluation?.tw,kf. | 2135470 | Advanced |  |  |  |
| 158 | (report* adj3 outcome?).tw,kf. | 145981 | Advanced |  |  |  |
| 159 | PROM?.tw,kf. | 13862 | Advanced |  |  |  |
| 160 | equit*.tw,kf. | 55841 | Advanced |  |  |  |
| 161 | inequit*.tw,kf. | 23224 | Advanced |  |  |  |
| 162 | equalit*.tw,kf. | 13097 | Advanced |  |  |  |
| 163 | inequalit*.tw,kf. | 50375 | Advanced |  |  |  |
| 164 | dispar*.tw,kf. | 166992 | Advanced |  |  |  |
| 165 | "social* determin* of health?".tw,kf. | 14477 | Advanced |  |  |  |
| 166 | (social* adj3 gradient*).tw,kf. | 1540 | Advanced |  |  |  |
| 167 | satisf*.tw,kf. | 556573 | Advanced |  |  |  |
| 168 | experienc*.tw,kf. | 1970051 | Advanced |  |  |  |
| 169 | safet*.tw,kf. | 1073400 | Advanced |  |  |  |
| 170 | or/92-169 | 7587681 | Advanced |  |  |  |
| 171 | 91 and 170 | 163459 | Advanced |  |  |  |
| 172 | review/ | 2908369 | Advanced |  |  |  |
| 173 | (medline or medlars or pubmed or grateful med or CINAHL or scisearch or psychinfo or psycinfo or psychlit or psyclit or handsearch* or hand search* or manual* search* or electronic database* or bibliographic database* or embase or lilacs or scopus or web of science).mp,kw. | 497065 | Advanced |  |  |  |
| 174 | 172 and 173 | 200747 | Advanced |  |  |  |
| 175 | "systematic review"/ | 433903 | Advanced |  |  |  |
| 176 | "systematic review (topic)"/ | 31471 | Advanced |  |  |  |
| 177 | exp meta analysis/ | 293001 | Advanced |  |  |  |
| 178 | network meta-analysis/ | 7762 | Advanced |  |  |  |
| 179 | "meta analysis (topic)"/ | 52722 | Advanced |  |  |  |
| 180 | exp biomedical technology assessment/ | 17102 | Advanced |  |  |  |
| 181 | high-cost technology/ | 63 | Advanced |  |  |  |
| 182 | (cochrane or (health adj2 technology assessment) or evidence report).jw. | 31243 | Advanced |  |  |  |
| 183 | (quantitative* adj3 (synthes* or review? or overview?)).tw,kf. | 9915 | Advanced |  |  |  |
| 184 | (qualitative* adj3 (synthes* or review? or overview?)).tw,kf. | 15602 | Advanced |  |  |  |
| 185 | (knowledge adj3 synthes*).tw,kf. | 2580 | Advanced |  |  |  |
| 186 | (systematic* adj3 (synthes* or review? or overview? or study or studies)).tw,kf. | 432232 | Advanced |  |  |  |
| 187 | (scoping adj3 (synthes* or review? or overview? or study or studies)).tw,kf. | 23145 | Advanced |  |  |  |
| 188 | (mapping adj3 (synthes* or review? or overview? or study or studies)).tw,kf. | 10846 | Advanced |  |  |  |
| 189 | (rapid adj3 (synthes* or review? or overview? or study or studies)).tw,kf. | 16956 | Advanced |  |  |  |
| 190 | (umbrella adj3 (synthes* or review? or overview? or study or studies)).tw,kf. | 1884 | Advanced |  |  |  |
| 191 | ("review of review?" or "review of systematic review?").tw,kf. | 11676 | Advanced |  |  |  |
| 192 | ("overview? of review?" or "overview? of systematic review?").tw,kf. | 1746 | Advanced |  |  |  |
| 193 | ("summar* of review?" or "summar* of systematic review?").tw,kf. | 4207 | Advanced |  |  |  |
| 194 | ("synthes* of review?" or "synthes* of systematic review?").tw,kf. | 1595 | Advanced |  |  |  |
| 195 | (integrative* adj3 (synthes* or review? or overview? or study or studies)).tw,kf. | 8618 | Advanced |  |  |  |
| 196 | (narrative adj3 (synthes* or review? or overview? or study or studies)).tw,kf. | 40265 | Advanced |  |  |  |
| 197 | (methodologic* adj3 (synthes* or review? or overview? or study or studies)).tw,kf. | 20704 | Advanced |  |  |  |
| 198 | (methodologic* adj3 (synthes* or review? or overview? or study or studies)).tw,kf. | 20704 | Advanced |  |  |  |
| 199 | (research adj3 (synthes* or review? or overview? or study or studies)).tw,kf. | 145137 | Advanced |  |  |  |
| 200 | (collaborative* adj3 (synthes* or review? or overview? or study or studies)).tw,kf. | 15523 | Advanced |  |  |  |
| 201 | (metaanal* or metanal* or (meta adj2 anal*)).tw,kf. | 362037 | Advanced |  |  |  |
| 202 | (metasummar* or (meta adj2 summar*)).tw,kf. | 1848 | Advanced |  |  |  |
| 203 | (metasynthes* or (meta adj2 synthes*)).tw,kf. | 4392 | Advanced |  |  |  |
| 204 | (metareview* or (meta adj2 review*)).tw,kf. | 148846 | Advanced |  |  |  |
| 205 | (metanetwork* or (meta adj2 network*)).tw,kf. | 13401 | Advanced |  |  |  |
| 206 | (metaaggregat* or (meta adj2 aggregat*)).tw,kf. | 798 | Advanced |  |  |  |
| 207 | (metaregression* or (meta adj2 regression*)).tw,kf. | 18673 | Advanced |  |  |  |
| 208 | (metaethnograph* or (meta adj2 ethnograph*)).tw,kf. | 978 | Advanced |  |  |  |
| 209 | (metanarrative* or (meta adj2 narrative*)).tw,kf. | 521 | Advanced |  |  |  |
| 210 | (pooled adj1 anal*).tw,kf. | 26054 | Advanced |  |  |  |
| 211 | (statistical* adj1 pooling).tw,kf. | 528 | Advanced |  |  |  |
| 212 | (statistical* adj1 summar*).tw,kf. | 652 | Advanced |  |  |  |
| 213 | (mathematical* adj1 pooling).tw,kf. | 2 | Advanced |  |  |  |
| 214 | (mathematical* adj1 summar*).tw,kf. | 42 | Advanced |  |  |  |
| 215 | (data adj3 (synthes* or extraction* or abstraction*)).tw,kf. | 65683 | Advanced |  |  |  |
| 216 | (mantel haenszel or peto or der simonian or dersimonian or fixed effect* or latin square*).tw,kf. | 47624 | Advanced |  |  |  |
| 217 | (comparative adj3 (efficacy or effectiveness)).tw,kf. | 26137 | Advanced |  |  |  |
| 218 | (outcomes research or relative effectiveness).tw,kf. | 16373 | Advanced |  |  |  |
| 219 | ((indirect or indirect treatment?) adj3 comparison*).tw,kf. | 6249 | Advanced |  |  |  |
| 220 | (mixed adj3 treatment? adj3 comparison*).tw,kf. | 1032 | Advanced |  |  |  |
| 221 | (multi* adj3 treatment? adj3 comparison*).tw,kf. | 535 | Advanced |  |  |  |
| 222 | (bayesian adj3 comparison*).tw,kf. | 836 | Advanced |  |  |  |
| 223 | (multi* adj2 paramet* adj2 evidence adj2 synthesis).tw,kf. | 28 | Advanced |  |  |  |
| 224 | ((multiparamet* or multi paramet*) adj2 evidence adj2 synthesis).tw,kf. | 44 | Advanced |  |  |  |
| 225 | (handsearch* or hand search*).tw,kf. | 13681 | Advanced |  |  |  |
| 226 | (technology assessment* or technology overview* or technology appraisal*).tw,kf. | 14037 | Advanced |  |  |  |
| 227 | (HTA or HTAs).tw,kf. | 9403 | Advanced |  |  |  |
| 228 | or/174-227 | 1181853 | Advanced |  |  |  |
| 229 | 171 and 228 | 14247 | Advanced |  |  |  |
| 230 | (virtual* adj10 care?).tw,kf. | 6052 | Advanced |  |  |  |
| 231 | 228 and 230 | 494 | Advanced |  |  |  |
| 232 | 229 or 231 | 14368 | Advanced |  |  |  |
| 233 | (exp animals/ or exp animal experimentation/ or nonhuman/) not ((exp animals/ or exp animal experimentation/ or nonhuman/) and exp human/) | 7253813 | Advanced |  |  |  |
| 234 | 232 not 233 | 14286 | Advanced |  |  |  |
| 235 | limit 234 to (books or chapter or conference abstract or conference paper or "conference review" or tombstone) | 2781 | Advanced |  |  |  |
| 236 | 234 not 235 | 11505 | Advanced |  |  |  |
| 237 | limit 236 to yr="2015 -Current" | 9132 | Advanced |  |  |  |
| 238 | limit 237 to english language | 8973 | Advanced |  |  |  |

**Elsevier Scopus 2015 to May 15, 2023**

| # | Search | Results |
| --- | --- | --- |
| 1 | TITLE-ABS-KEY ( virtual* OR telemed* OR tele-med* OR telehealth* OR tele-health* OR teleinterven* OR tele-interven* OR teletherap* OR tele-therap* OR teletreat* OR tele-treat* OR telesurg* OR tele-surg* OR telediagnos* OR tele-diagnos* OR telerad* OR tele-rad* OR televisit* OR tele-visit* OR tele-ambulator* OR “tele-urgent care?” OR telerehab* OR tele-rehab* OR teleclinic* OR tele-clinic* OR telecare? OR tele-care? OR telenavigat* OR tele-navigat* OR teleconsult* OR tele-consult* OR telecounsel* OR tele-counsel* OR tele-aftercare? OR “tele-after care?” OR teleservice? OR tele-service? OR telefollow* OR tele-follow* OR ( mobile* W/3 health* ) OR ( mobile* W/3 interven* ) OR ( mobile* W/3 therap* ) OR ( mobile* W/3 treat* ) OR ( mobile* W/3 surg* ) OR ( mobile* W/3 diagnos* ) OR ( mobile* W/3 rad* ) OR ( mobile* W/3 visit* ) OR ( mobile* W/3 ambulator* ) OR ( mobile* W/3 urgent AND care? ) OR ( mobile* W/3 rehab* ) OR ( mobile* W/3 clinic* ) OR ( mobile* W/3 care? ) OR ( mobile* W/3 navigat* ) OR ( mobile* W/3 consult* ) OR ( mobile* W/3 counsel* ) OR ( mobile* W/3 service? ) OR ( mobile* W/3 follow* ) OR ( ( online? OR on-line? ) W/3 health* ) OR ( ( online? OR on-line? ) W/3 interven* ) OR ( ( online? OR on-line? ) W/3 therap* ) OR ( ( online? OR on-line? ) W/3 treat* ) OR ( ( online? OR on-line? ) W/3 surg* ) OR ( ( online? OR on-line? ) W/3 diagnos* ) OR ( ( online? OR on-line? ) W/3 rad* ) OR ( ( online? OR on-line? ) W/3 visit* ) OR ( ( online? OR on-line? ) W/3 ambulator* ) OR ( ( online? OR on-line? ) W/3 urgent AND care? ) OR ( ( online? OR on-line? ) W/3 rehab* ) OR ( ( online? OR on-line? ) W/3 clinic* ) OR ( ( online? OR on-line? ) W/3 care? ) OR ( ( online? OR on-line? ) W/3 navigat* ) OR ( ( online? OR on-line? ) W/3 consult* ) OR ( ( online? OR on-line? ) W/3 counsel* ) OR ( ( online? OR on-line? ) W/3 service? ) OR ( ( online? OR on-line? ) W/3 follow* ) OR ( remote* W/3 health* ) OR ( remote* W/3 interven* ) OR ( remote* W/3 therap* ) OR ( remote* W/3 treat* ) OR ( remote* W/3 surg* ) OR ( remote* W/3 diagnos* ) OR ( remote* W/3 rad* ) OR ( remote* W/3 visit* ) OR ( remote* W/3 ambulator* ) OR ( remote* W/3 urgent AND care? ) OR ( remote* W/3 rehab* ) OR ( remote* W/3 clinic* ) OR ( remote* W/3 care? ) OR ( remote* W/3 navigat* ) OR ( remote* W/3 consult* ) OR ( remote* W/3 counsel* ) OR ( remote* W/3 service? ) OR ( remote* W/3 follow* ) OR ehealth* OR e-health* OR mhealth* OR m-health* OR thealth* OR t-health* OR uhealth* OR u-health* OR vhealth* OR v-health* ) | 997,061 |
| 2 | TITLE-ABS-KEY ( ( qualit* W/3 health* ) OR ( qualit* W/3 care? ) OR ( qualit* W/3 assuran* ) OR ( qualit* W/3 assess* ) OR ( qualit* W/3 improv* ) OR ( qualit* W/3 indicat* ) OR ( qualit* W/3 metric? ) OR ( qualit* W/3 measur* ) OR ( qualit* W/3 manag* ) OR ( qualit* W/3 evaluat* ) OR ( quality W/3 outcome? ) OR ( quality W/3 ( framework? OR frame-work? ) ) OR ( quality W/3 tool? ) OR ( cost?? OR costing ) OR ( service? W/3 utili* ) OR ( service? W/3 evaluat* ) OR ( resource? W/3 utili* ) OR ( resource? W/3 evaluat* ) OR ( process* W/3 measur* ) OR ( process* W/3 evaluat* ) OR ( technolog* W/3 access* ) OR ( technolog* W/3 limit* ) OR ( effective* W/3 evaluat* ) OR ( outcome? W/3 evaluat* ) OR ( care? W/3 evaluat* ) OR ( ( framework? OR frame-work? ) W/3 evaluat* ) OR ( integrat* W/3 evaluat* ) OR evaluation? OR ( report* W/3 outcome? ) OR prom? OR equit* OR inequit* OR equalit* OR inequalit* OR dispar* OR "social* determin* of health?" OR ( social* W/3 gradient* ) OR satisf* OR experienc* OR safet* ) | 9,332,431 |
| 3 | TITLE-ABS-KEY ( ( systematic* W/3 ( review* OR overview* ) ) OR ( methodologic* W/3 ( review* OR overview* ) ) ) OR TITLE-ABS-KEY ( ( quantitative W/3 ( review* OR overview* OR synthes* ) ) OR ( research W/3 ( integrati* OR overview* ) ) ) OR TITLE-ABS-KEY ( ( integrative W/3 ( review* OR overview* ) ) OR ( collaborative W/3 ( review* OR overview* ) ) OR ( pool* W/3 analy* ) ) OR TITLE-ABS-KEY ( "data synthes*" OR "data extraction*" OR "data abstraction*" ) OR TITLE-ABS-KEY ( handsearch* OR "hand search*" ) OR TITLE-ABS-KEY ( "mantel haenszel" OR peto OR "der simonian" OR dersimonian OR "fixed effect*" OR "latin square*" ) OR TITLE-ABS-KEY ( "met analy*" OR metanaly* OR "technology assessment*" OR hta OR htas OR "technology overview*" OR "technology appraisal*" ) OR TITLE-ABS-KEY ( "meta regression*" OR metaregression* ) OR TITLE-ABS-KEY ( meta-analy* OR metaanaly* OR "systematic review*" OR "biomedical technology assessment*" OR "bio-medical technology assessment*" ) OR TITLE-ABS-KEY ( medline OR cochrane OR pubmed OR medlars OR embase OR cinahl ) OR SRCTITLE ( cochrane OR ( health W/2 "technology assessment" ) OR "evidence report" ) OR TITLE-ABS-KEY ( comparative W/3 ( efficacy OR effectiveness ) ) OR TITLE-ABS-KEY ( "outcomes research" OR "relative effectiveness" ) OR TITLE-ABS-KEY ( ( indirect OR "indirect treatment" OR mixed-treatment OR bayesian ) W/3 comparison* ) OR TITLE-ABS-KEY ( multi* W/3 treatment W/3 comparison* ) OR TITLE-ABS-KEY ( mixed W/3 treatment W/3 ( meta-analy* OR metaanaly* ) ) OR TITLE-ABS-KEY ( "umbrella review*" ) OR TITLE-ABS-KEY ( multi* W/2 paramet* W/2 evidence W/2 synthesis ) OR TITLE-ABS-KEY ( multiparamet* W/2 evidence W/2 synthesis ) OR TITLE-ABS-KEY ( multi-paramet* W/2 evidence W/2 synthesis ) | 1,149,326 |
| 4 | 1 AND 2 AND 3 | 9,676 |
| 5 | 4 AND PUBYEAR > 2014 AND PUBYEAR > 2014 AND ( EXCLUDE ( DOCTYPE , "cp" ) OR EXCLUDE ( DOCTYPE , "cr" ) OR EXCLUDE ( DOCTYPE , "ch" ) OR EXCLUDE ( DOCTYPE , "no" ) ) AND ( EXCLUDE ( DOCTYPE , "bk" ) OR EXCLUDE ( DOCTYPE , "tb" ) ) | 7,078 |
| 6 | 5 AND ( LIMIT-TO ( LANGUAGE , "English" ) ) | 6,835 |

**Clarivate Web of Science 2015 to May 15, 2023**

| # | Search | Results |
| --- | --- | --- |
| 1 | TS=(virtual* OR telemed* OR tele-med* OR telehealth* OR tele-health* OR teleinterven* OR (tele* NEAR/3 interven*) OR teletherap* OR (tele* NEAR/3 therap*) OR teletreat* OR (tele* NEAR/3 treat*) OR telesurg* OR (tele* NEAR/3 surg*) OR telediagnos* OR (tele* NEAR/3 diagnos*) OR telerad* OR (tele* NEAR/3 rad*) OR televisit* OR (tele* NEAR/3 visit*) OR (tele* NEAR/3 ambulator*) OR (tele* NEAR/3 urgent care?) OR telerehab* OR (tele* NEAR/3 rehab*) OR teleclinic* OR (tele* NEAR/3 clinic*) OR telecare? OR (tele* NEAR/3 care?) OR telenavigat* OR (tele* NEAR/3 navigat*) OR teleconsult* or (tele* NEAR/3 consult*) OR telecounsel* OR (tele* NEAR/3 counsel*) OR (tele* (aftercare? or after care?)) OR teleservice? OR (tele* NEAR/3 service?) OR telefollow* OR (tele* NEAR/3 follow*) OR (mobile* NEAR/3 health*) OR (mobile* NEAR/3 interven*) OR (mobile* NEAR/3 therap*) OR (mobile* NEAR/3 treat*) OR (mobile* NEAR/3 surg*) OR (mobile* NEAR/3 diagnos*) OR (mobile* NEAR/3 rad*) OR (mobile* NEAR/3 visit*) OR (mobile* NEAR/3 ambulator*) OR (mobile* NEAR/3 urgent care?) OR (mobile* NEAR/3 rehab*) OR (mobile* NEAR/3 clinic*) OR (mobile* NEAR/3 care?) OR (mobile* NEAR/3 navigat*) OR (mobile* NEAR/3 consult*) OR (mobile* NEAR/3 counsel*) OR (mobile* (aftercare? or after care?)) OR (mobile* NEAR/3 service?) OR (mobile* NEAR/3 follow*) OR ((online? or on-line?) NEAR/3 health*) OR ((online? or on-line?) NEAR/3 interven*) OR ((online? or on-line?) NEAR/3 therap*) OR ((online? or on-line?) NEAR/3 treat*) OR ((online? or on-line?) NEAR/3 surg*) OR ((online? or on-line?) NEAR/3 diagnos*) OR ((online? or on-line?) NEAR/3 rad*) OR ((online? or on-line?) NEAR/3 visit*) OR ((online? or on-line?) NEAR/3 ambulator*) OR ((online? or on-line?) NEAR/3 urgent care?) OR ((online? or on-line?) NEAR/3 rehab*) OR ((online? or on-line?) NEAR/3 clinic*) OR ((online? or on-line?) NEAR/3 care?) OR ((online? or on-line?) NEAR/3 navigat*) OR ((online? or on-line?) NEAR/3 consult*) OR ((online? or on-line?) NEAR/3 counsel*) OR ((online? or on-line?) NEAR/3 service?) OR ((online? or on-line?) NEAR/3 follow*) OR (remote* NEAR/3 health*) OR (remote* NEAR/3 interven*) OR (remote* NEAR/3 therap*) OR (remote* NEAR/3 treat*) OR (remote* NEAR/3 surg*) OR (remote* NEAR/3 diagnos*) OR (remote* NEAR/3 rad*) OR (remote* NEAR/3 visit*) OR (remote* NEAR/3 ambulator*) OR (remote* NEAR/3 urgent care?) OR (remote* NEAR/3 rehab*) OR (remote* NEAR/3 clinic*) OR (remote* NEAR/3 care?) OR (remote* NEAR/3 navigat*) OR (remote* NEAR/3 consult*) OR (remote* NEAR/3 counsel*) OR (remote* NEAR/3 service?) OR (remote* NEAR/3 follow*) OR ehealth* OR e-health* OR mhealth* OR m-health* OR thealth* OR t-health* OR uhealth* OR u-health* OR vhealth* OR v-health*) | 715,601 |
| 2 | TS=((qualit* NEAR/3 health*) OR (qualit* NEAR/3 care?) OR (qualit* NEAR/3 assuran*) OR (qualit* NEAR/3 assess*) OR (qualit* NEAR/3 improv*) OR (qualit* NEAR/3 indicat*) OR (qualit* NEAR/3 metric?) OR (qualit* NEAR/3 measur*) OR (qualit* NEAR/3 manag*) OR (qualit* NEAR/3 evaluat*) OR (quality NEAR/3 outcome?) OR (quality NEAR/3 (framework? or frame-work?)) OR (quality NEAR/3 tool?) OR (cost?? or costing) OR (service? NEAR/3 utili*) OR (service? NEAR/3 evaluat*) OR (resource? NEAR/3 utili*) OR (resource? NEAR/3 evaluat*) OR (process* NEAR/3 measur*) OR (process* NEAR/3 evaluat*) OR (technolog* NEAR/3 access*) OR (technolog* NEAR/3 limit*) OR (effective* NEAR/3 evaluat*) OR (outcome? NEAR/3 evaluat*) OR (care? NEAR/3 evaluat*) OR ((framework? or frame-work?) NEAR/3 evaluat*) OR (integrat* NEAR/3 evaluat*) OR evaluation? OR (report* NEAR/3 outcome?) OR PROM? OR equit* OR inequit* OR equalit* OR inequalit* OR dispar* OR "social* determin* of health?" OR (social* NEAR/3 gradient*) OR satisf* OR experienc* OR safet*) | 8,532,387 |
| 3 | TS=((systematic* AND (review* OR overview* )) OR (methodologic* AND (review* OR overview* )) OR (quantitative AND (review* OR overview* OR synthes* )) OR (research AND (integrati* OR overview* )) OR (integrative AND (review* OR overview* )) OR (collaborative AND (review* OR overview* )) OR (pool* NEAE/3 analy* ) OR "data synthes*" OR "data extraction*" OR "data abstraction*" OR handsearch* OR "hand search*" OR "mantel haenszel" OR peto OR "der simonian" OR dersimonian OR "fixed effect*" OR "latin square*" OR "met analy*" OR metanaly* OR "technology assessment*" OR HTA OR HTAs OR "technology overview*" OR "technology appraisal*" OR "meta regression*" OR metaregression* OR meta-analy* OR metaanaly* OR "systematic review*" OR "biomedical technology assessment*" OR "bio-medical technology assessment*" OR (health NEAE/2 "technology assessment" ) OR "evidence report" OR (comparative AND (efficacy OR effectiveness )) OR "outcomes research" OR "relative effectiveness" OR ((indirect OR "indirect treatment" OR mixed-treatment OR bayesian ) AND comparison* ) OR (treatment NEAE/3 comparison* ) OR "umbrella review*" OR (evidence NEAE/2 synthesis )) | 1,470,375 |
| 4 | 1 AND 2 AND 3 | 14,733 |
| 5 | 4 AND Proceeding Paper or Book Chapters or Meeting Abstract or Meeting or Reprint or Retracted Publication or Book or Book Review or Discussion (Exclude – Document Types) | 12,582 |
| 6 | 5 \|Timespan: 2015-01-01 to 2023-12-31 (Index Date) | 10,195 |
| 5 | 6 AND English (Languages) | 9,799 |

**Ovid Cochrane Database of Systematic Reviews May 9, 2023**

| **#** | **Searches** | **Results** | **Type** |  |  |  |
| --- | --- | --- | --- | --- | --- | --- |
|  | | | | | | |
| 1 | virtual*.ti,ab,kw. | 59 | Advanced |  |  |  |
| 2 | (tele*med* or tele*-med*).ti,ab,kw. | 28 | Advanced |  |  |  |
| 3 | (tele*health* or tele*-health*).ti,ab,kw. | 13 | Advanced |  |  |  |
| 4 | (tele*interven* or (tele* adj3 interven*)).ti,ab,kw. | 27 | Advanced |  |  |  |
| 5 | (tele*therap* or (tele* adj3 therap*)).ti,ab,kw. | 5 | Advanced |  |  |  |
| 6 | (tele*treat* or (tele* adj3 treat*)).ti,ab,kw. | 3 | Advanced |  |  |  |
| 7 | (tele*surg* or (tele* adj3 surg*)).ti,ab,kw. | 0 | Advanced |  |  |  |
| 8 | (tele*diagnos* or (tele* adj3 diagnos*)).ti,ab,kw. | 5 | Advanced |  |  |  |
| 9 | (tele*rad* or (tele* adj3 rad*)).ti,ab,kw. | 3 | Advanced |  |  |  |
| 10 | (tele*visit* or (tele* adj3 visit*)).ti,ab,kw. | 4 | Advanced |  |  |  |
| 11 | (tele* adj3 ambulator*).ti,ab,kw. | 0 | Advanced |  |  |  |
| 12 | (tele* adj3 urgent care?).ti,ab,kw. | 0 | Advanced |  |  |  |
| 13 | (tele*rehab* or (tele* adj3 rehab*)).ti,ab,kw. | 13 | Advanced |  |  |  |
| 14 | (tele*clinic* or (tele* adj3 clinic*)).ti,ab,kw. | 7 | Advanced |  |  |  |
| 15 | (tele*care? or (tele* adj3 care?)).ti,ab,kw. | 15 | Advanced |  |  |  |
| 16 | (tele*navigat* or (tele* adj3 navigat*)).ti,ab,kw. | 0 | Advanced |  |  |  |
| 17 | (tele*consult* or (tele* adj3 consult*)).ti,ab,kw. | 15 | Advanced |  |  |  |
| 18 | (tele*counsel* or (tele* adj3 counsel*)).ti,ab,kw. | 8 | Advanced |  |  |  |
| 19 | (tele* adj3 (aftercare? or after care?)).ti,ab,kw. | 0 | Advanced |  |  |  |
| 20 | (tele*service? or (tele* adj3 service?)).ti,ab,kw. | 10 | Advanced |  |  |  |
| 21 | (tele*follow* up? or (tele* adj3 follow* up?)).ti,ab,kw. | 11 | Advanced |  |  |  |
| 22 | (mobile* adj3 health*).ti,ab,kw. | 9 | Advanced |  |  |  |
| 23 | (mobile* adj3 interven*).ti,ab,kw. | 14 | Advanced |  |  |  |
| 24 | (mobile* adj3 therap*).ti,ab,kw. | 0 | Advanced |  |  |  |
| 25 | (mobile* adj3 treat*).ti,ab,kw. | 1 | Advanced |  |  |  |
| 26 | (mobile* adj3 surg*).ti,ab,kw. | 0 | Advanced |  |  |  |
| 27 | (mobile* adj3 diagnos*).ti,ab,kw. | 0 | Advanced |  |  |  |
| 28 | (mobile* adj3 rad*).ti,ab,kw. | 0 | Advanced |  |  |  |
| 29 | (mobile* adj3 visit*).ti,ab,kw. | 1 | Advanced |  |  |  |
| 30 | (mobile* adj3 ambulator*).ti,ab,kw. | 0 | Advanced |  |  |  |
| 31 | (mobile* adj3 urgent care?).ti,ab,kw. | 0 | Advanced |  |  |  |
| 32 | (mobile* adj3 rehab*).ti,ab,kw. | 0 | Advanced |  |  |  |
| 33 | (mobile* adj3 clinic*).ti,ab,kw. | 3 | Advanced |  |  |  |
| 34 | (mobile* adj3 care?).ti,ab,kw. | 3 | Advanced |  |  |  |
| 35 | (mobile* adj3 navigat*).ti,ab,kw. | 0 | Advanced |  |  |  |
| 36 | (mobile* adj3 consult*).ti,ab,kw. | 0 | Advanced |  |  |  |
| 37 | (mobile* adj3 counsel*).ti,ab,kw. | 0 | Advanced |  |  |  |
| 38 | (mobile* adj3 (aftercare? or after care?)).ti,ab,kw. | 0 | Advanced |  |  |  |
| 39 | (mobile* adj3 service?).ti,ab,kw. | 2 | Advanced |  |  |  |
| 40 | (mobile* adj3 follow* up?).ti,ab,kw. | 0 | Advanced |  |  |  |
| 41 | ((online? or on-line?) adj3 health*).ti,ab,kw. | 3 | Advanced |  |  |  |
| 42 | ((online? or on-line?) adj3 interven*).ti,ab,kw. | 6 | Advanced |  |  |  |
| 43 | ((online? or on-line?) adj3 therap*).ti,ab,kw. | 0 | Advanced |  |  |  |
| 44 | ((online? or on-line?) adj3 treat*).ti,ab,kw. | 0 | Advanced |  |  |  |
| 45 | ((online? or on-line?) adj3 surg*).ti,ab,kw. | 0 | Advanced |  |  |  |
| 46 | ((online? or on-line?) adj3 diagnos*).ti,ab,kw. | 0 | Advanced |  |  |  |
| 47 | ((online? or on-line?) adj3 rad*).ti,ab,kw. | 0 | Advanced |  |  |  |
| 48 | ((online? or on-line?) adj3 visit*).ti,ab,kw. | 0 | Advanced |  |  |  |
| 49 | ((online? or on-line?) adj3 ambulator*).ti,ab,kw. | 0 | Advanced |  |  |  |
| 50 | ((online? or on-line?) adj3 urgent care?).ti,ab,kw. | 0 | Advanced |  |  |  |
| 51 | ((online? or on-line?) adj3 rehab*).ti,ab,kw. | 1 | Advanced |  |  |  |
| 52 | ((online? or on-line?) adj3 clinic*).ti,ab,kw. | 49 | Advanced |  |  |  |
| 53 | ((online? or on-line?) adj3 care?).ti,ab,kw. | 6 | Advanced |  |  |  |
| 54 | ((online? or on-line?) adj3 navigat*).ti,ab,kw. | 0 | Advanced |  |  |  |
| 55 | ((online? or on-line?) adj3 consult*).ti,ab,kw. | 1 | Advanced |  |  |  |
| 56 | ((online? or on-line?) adj3 counsel*).ti,ab,kw. | 0 | Advanced |  |  |  |
| 57 | ((online? or on-line?) adj3 (aftercare? or after care?)).ti,ab,kw. | 0 | Advanced |  |  |  |
| 58 | ((online? or on-line?) adj3 service?).ti,ab,kw. | 1 | Advanced |  |  |  |
| 59 | ((online? or on-line?) adj3 follow* up?).ti,ab,kw. | 0 | Advanced |  |  |  |
| 60 | (remote* adj3 health*).ti,ab,kw. | 2 | Advanced |  |  |  |
| 61 | (remote* adj3 interven*).ti,ab,kw. | 10 | Advanced |  |  |  |
| 62 | (remote* adj3 therap*).ti,ab,kw. | 2 | Advanced |  |  |  |
| 63 | (remote* adj3 treat*).ti,ab,kw. | 1 | Advanced |  |  |  |
| 64 | (remote* adj3 surg*).ti,ab,kw. | 1 | Advanced |  |  |  |
| 65 | (remote* adj3 diagnos*).ti,ab,kw. | 1 | Advanced |  |  |  |
| 66 | (remote* adj3 rad*).ti,ab,kw. | 0 | Advanced |  |  |  |
| 67 | (remote* adj3 visit*).ti,ab,kw. | 1 | Advanced |  |  |  |
| 68 | (remote* adj3 ambulator*).ti,ab,kw. | 0 | Advanced |  |  |  |
| 69 | (remote* adj3 urgent care?).ti,ab,kw. | 0 | Advanced |  |  |  |
| 70 | (remote* adj3 rehab*).ti,ab,kw. | 1 | Advanced |  |  |  |
| 71 | (remote* adj3 clinic*).ti,ab,kw. | 2 | Advanced |  |  |  |
| 72 | (remote* adj3 care?).ti,ab,kw. | 2 | Advanced |  |  |  |
| 73 | (remote* adj3 navigat*).ti,ab,kw. | 0 | Advanced |  |  |  |
| 74 | (remote* adj3 consult*).ti,ab,kw. | 8 | Advanced |  |  |  |
| 75 | (remote* adj3 counsel*).ti,ab,kw. | 1 | Advanced |  |  |  |
| 76 | (remote* adj3 (aftercare? or after care?)).ti,ab,kw. | 0 | Advanced |  |  |  |
| 77 | (remote* adj3 service?).ti,ab,kw. | 2 | Advanced |  |  |  |
| 78 | (remote* adj3 follow* up?).ti,ab,kw. | 0 | Advanced |  |  |  |
| 79 | (ehealth* or e-health*).ti,ab,kw. | 3 | Advanced |  |  |  |
| 80 | (mhealth* or m-health*).ti,ab,kw. | 10 | Advanced |  |  |  |
| 81 | (thealth* or t-health*).ti,ab,kw. | 0 | Advanced |  |  |  |
| 82 | (uhealth* or u-health*).ti,ab,kw. | 0 | Advanced |  |  |  |
| 83 | (vhealth* or v-health*).ti,ab,kw. | 0 | Advanced |  |  |  |
| 84 | or/1-83 | 249 | Advanced |  |  |  |
| 85 | (qualit* adj3 health*).ti,ab,kw. | 632 | Advanced |  |  |  |
| 86 | (qualit* adj3 care?).ti,ab,kw. | 131 | Advanced |  |  |  |
| 87 | (qualit* adj3 assuran*).ti,ab,kw. | 6 | Advanced |  |  |  |
| 88 | (qualit* adj3 assess*).ti,ab,kw. | 2684 | Advanced |  |  |  |
| 89 | (qualit* adj3 improv*).ti,ab,kw. | 479 | Advanced |  |  |  |
| 90 | (qualit* adj3 indicat*).ti,ab,kw. | 124 | Advanced |  |  |  |
| 91 | (qualit* adj3 metric?).ti,ab,kw. | 1 | Advanced |  |  |  |
| 92 | (qualit* adj3 measur*).ti,ab,kw. | 222 | Advanced |  |  |  |
| 93 | (qualit* adj3 manag*).ti,ab,kw. | 10 | Advanced |  |  |  |
| 94 | (qualit* adj3 evaluat*).ti,ab,kw. | 265 | Advanced |  |  |  |
| 95 | (quality adj3 outcome?).ti,ab,kw. | 472 | Advanced |  |  |  |
| 96 | (quality adj3 (framework? or frame-work?)).ti,ab,kw. | 2 | Advanced |  |  |  |
| 97 | (quality adj3 tool?).ti,ab,kw. | 40 | Advanced |  |  |  |
| 98 | (cost?? or costing).ti,ab,kw. | 1320 | Advanced |  |  |  |
| 99 | (service? adj3 utili*).ti,ab,kw. | 58 | Advanced |  |  |  |
| 100 | (service? adj3 evaluat*).ti,ab,kw. | 9 | Advanced |  |  |  |
| 101 | (resource? adj3 utili*).ti,ab,kw. | 24 | Advanced |  |  |  |
| 102 | (resource? adj3 evaluat*).ti,ab,kw. | 2 | Advanced |  |  |  |
| 103 | (process* adj3 measur*).ti,ab,kw. | 10 | Advanced |  |  |  |
| 104 | (process* adj3 evaluat*).ti,ab,kw. | 18 | Advanced |  |  |  |
| 105 | (technolog* adj3 access*).ti,ab,kw. | 3 | Advanced |  |  |  |
| 106 | (technolog* adj3 limit*).ti,ab,kw. | 1 | Advanced |  |  |  |
| 107 | (effective* adj3 evaluat*).ti,ab,kw. | 577 | Advanced |  |  |  |
| 108 | (outcome? adj3 evaluat*).ti,ab,kw. | 181 | Advanced |  |  |  |
| 109 | (care? adj3 evaluat*).ti,ab,kw. | 23 | Advanced |  |  |  |
| 110 | ((framework? or frame-work?) adj3 evaluat*).ti,ab,kw. | 3 | Advanced |  |  |  |
| 111 | (integrat* adj3 evaluat*).ti,ab,kw. | 3 | Advanced |  |  |  |
| 112 | evaluation?.ti,ab,kw. | 1015 | Advanced |  |  |  |
| 113 | (report* adj3 outcome?).ti,ab,kw. | 1553 | Advanced |  |  |  |
| 114 | PROM?.ti,ab,kw. | 15 | Advanced |  |  |  |
| 115 | equit*.ti,ab,kw. | 44 | Advanced |  |  |  |
| 116 | inequit*.ti,ab,kw. | 13 | Advanced |  |  |  |
| 117 | equalit*.ti,ab,kw. | 2 | Advanced |  |  |  |
| 118 | inequalit*.ti,ab,kw. | 14 | Advanced |  |  |  |
| 119 | dispar*.ti,ab,kw. | 30 | Advanced |  |  |  |
| 120 | "social* determin* of health?".ti,ab,kw. | 3 | Advanced |  |  |  |
| 121 | (social* adj3 gradient*).ti,ab,kw. | 1 | Advanced |  |  |  |
| 122 | satisf*.ti,ab,kw. | 662 | Advanced |  |  |  |
| 123 | experienc*.ti,ab,kw. | 1166 | Advanced |  |  |  |
| 124 | safet*.ti,ab,kw. | 2745 | Advanced |  |  |  |
| 125 | or/85-124 | 7347 | Advanced |  |  |  |
| 126 | 84 and 125 | 199 | Advanced |  |  |  |
| 127 | limit 126 to protocols | 2 | Advanced |  |  |  |
| 128 | 126 not 127 | 197 | Advanced |  |  |  |

**EBSCOhost CINAHL 2015 to May 12, 2023**

| \| **#** \| **Query** \| **Limiters/Expanders** \| **Last Run Via** \| **Results** \| \| --- \| --- \| --- \| --- \| --- \| \| S159 \| S88 AND S156 AND S157 \| Limiters - Published Date: 20150101-20231231  Expanders - Apply equivalent subjects  Narrow by Language: - english  Search modes - Boolean/Phrase \| Interface - EBSCOhost Research Databases  Search Screen - Advanced Search  Database - CINAHL Ultimate \| 5,025 \| \| S158 \| S88 AND S156 AND S157 \| Expanders - Apply equivalent subjects  Narrow by Language: - english  Search modes - Boolean/Phrase \| Interface - EBSCOhost Research Databases  Search Screen - Advanced Search  Database - CINAHL Ultimate \| 6,618 \| \| S157 \| (MH "meta analysis" OR MH "systematic review" OR MH "Technology, Medical/EV" OR PT "systematic review" OR PT "meta analysis" OR (((TI systematic* OR AB systematic*) N3 ((TI review* OR AB review*) OR (TI overview* OR AB overview*))) OR ((TI methodologic* OR AB methodologic*) N3 ((TI review* OR AB review*) OR (TI overview* OR AB overview*)))) OR (((TI quantitative OR AB quantitative) N3 ((TI review* OR AB review*) OR (TI overview* OR AB overview*) OR (TI synthes* OR AB synthes*))) OR ((TI research OR AB research) N3 ((TI integrati* OR AB integrati*) OR (TI overview* OR AB overview*)))) OR (((TI integrative OR AB integrative) N3 ((TI review* OR AB review*) OR (TI overview* OR AB overview*))) OR ((TI collaborative OR AB collaborative) N3 ((TI review* OR AB review*) OR (TI overview* OR AB overview*))) OR ((TI pool* OR AB pool*) N3 (TI analy* OR AB analy*))) OR ((TI "data synthes*" OR AB "data synthes*") OR (TI "data extraction*" OR AB "data extraction*") OR (TI "data abstraction*" OR AB "data abstraction*")) OR ((TI handsearch* OR AB handsearch*) OR (TI "hand search*" OR AB "hand search*")) OR ((TI "mantel haenszel" OR AB "mantel haenszel") OR (TI peto OR AB peto) OR (TI "der simonian" OR AB "der simonian") OR (TI dersimonian OR AB dersimonian) OR (TI "fixed effect*" OR AB "fixed effect*") OR (TI "latin square*" OR AB "latin square*")) OR ((TI "met analy*" OR AB "met analy*") OR (TI metanaly* OR AB metanaly*) OR (TI "technology assessment*" OR AB "technology assessment*") OR (TI HTA OR AB HTA) OR (TI HTAs OR AB HTAs) OR (TI "technology overview*" OR AB "technology overview*") OR (TI "technology appraisal*" OR AB "technology appraisal*")) OR ((TI "meta regression*" OR AB "meta regression*") OR (TI metaregression* OR AB metaregression*)) OR (MW meta-analy* OR MW metaanaly* OR MW "systematic review*" OR MW "biomedical technology assessment*" OR MW "bio-medical technology assessment*") OR ((TI medline OR AB medline OR MW medline) OR (TI cochrane OR AB cochrane OR MW cochrane) OR (TI pubmed OR AB pubmed OR MW pubmed) OR (TI medlars OR AB medlars OR MW medlars) OR (TI embase OR AB embase OR MW embase) OR (TI cinahl OR AB cinahl OR MW cinahl)) OR (SO Cochrane OR SO health technology assessment OR SO evidence report) OR ((TI comparative OR AB comparative) N3 ((TI efficacy OR AB efficacy) OR (TI effectiveness OR AB effectiveness))) OR ((TI "outcomes research" OR AB "outcomes research") OR (TI "relative effectiveness" OR AB "relative effectiveness")) OR (((TI indirect OR AB indirect) OR (TI "indirect treatment" OR AB "indirect treatment") OR (TI mixed-treatment OR AB mixed-treatment) OR (TI bayesian OR AB bayesian)) N3 (TI comparison* OR AB comparison*)) OR ((TI multi* OR AB multi*) N3 (TI treatment OR AB treatment) N3 (TI comparison* OR AB comparison*)) OR ((TI mixed OR AB mixed) N3 (TI treatment OR AB treatment) N3 ((TI meta-analy* OR AB meta-analy*) OR (TI metaanaly* OR AB metaanaly*))) OR (TI "umbrella review*" OR AB "umbrella review*") OR ((TI multi* OR AB multi*) N2 (TI paramet* OR AB paramet*) N2 (TI evidence OR AB evidence) N2 (TI synthesis OR AB synthesis)) OR ((TI multiparamet* OR AB multiparamet*) N2 (TI evidence OR AB evidence) N2 (TI synthesis OR AB synthesis)) OR ((TI multi-paramet* OR AB multi-paramet*) N2 (TI evidence OR AB evidence) N2 (TI synthesis OR AB synthesis)) \| Expanders - Apply equivalent subjects  Search modes - Boolean/Phrase \| Interface - EBSCOhost Research Databases  Search Screen - Advanced Search  Database - CINAHL Ultimate \| 300,975 \| \| S156 \| S89 OR S90 OR S91 OR S92 OR S93 OR S94 OR S95 OR S96 OR S97 OR S98 OR S99 OR S100 OR S101 OR S102 OR S103 OR S104 OR S105 OR S106 OR S107 OR S108 OR S109 OR S110 OR S111 OR S112 OR S113 OR S114 OR S115 OR S116 OR S117 OR S118 OR S119 OR S120 OR S121 OR S122 OR S123 OR S124 OR S125 OR S126 OR S127 OR S128 OR S129 OR S130 OR S131 OR S132 OR S133 OR S134 OR S135 OR S136 OR S137 OR S138 OR S139 OR S140 OR S141 OR S142 OR S143 OR S144 OR S145 OR S146 OR S147 OR S148 OR S149 OR S150 OR S151 OR S152 OR S153 OR S154 OR S155 \| Expanders - Apply equivalent subjects  Search modes - Boolean/Phrase \| Interface - EBSCOhost Research Databases  Search Screen - Advanced Search  Database - CINAHL Ultimate \| 2,479,864 \| \| S155 \| safet* \| Expanders - Apply equivalent subjects  Search modes - Boolean/Phrase \| Interface - EBSCOhost Research Databases  Search Screen - Advanced Search  Database - CINAHL Ultimate \| 319,783 \| \| S154 \| experienc* \| Expanders - Apply equivalent subjects  Search modes - Boolean/Phrase \| Interface - EBSCOhost Research Databases  Search Screen - Advanced Search  Database - CINAHL Ultimate \| 589,111 \| \| S153 \| satisf* \| Expanders - Apply equivalent subjects  Search modes - Boolean/Phrase \| Interface - EBSCOhost Research Databases  Search Screen - Advanced Search  Database - CINAHL Ultimate \| 198,983 \| \| S152 \| (social* N3 gradient*) \| Expanders - Apply equivalent subjects  Search modes - Boolean/Phrase \| Interface - EBSCOhost Research Databases  Search Screen - Advanced Search  Database - CINAHL Ultimate \| 643 \| \| S151 \| "social* determin* of health?" \| Expanders - Apply equivalent subjects  Search modes - Boolean/Phrase \| Interface - EBSCOhost Research Databases  Search Screen - Advanced Search  Database - CINAHL Ultimate \| 13,702 \| \| S150 \| dispar* \| Expanders - Apply equivalent subjects  Search modes - Boolean/Phrase \| Interface - EBSCOhost Research Databases  Search Screen - Advanced Search  Database - CINAHL Ultimate \| 65,947 \| \| S149 \| inequalit* \| Expanders - Apply equivalent subjects  Search modes - Boolean/Phrase \| Interface - EBSCOhost Research Databases  Search Screen - Advanced Search  Database - CINAHL Ultimate \| 22,473 \| \| S148 \| equalit* \| Expanders - Apply equivalent subjects  Search modes - Boolean/Phrase \| Interface - EBSCOhost Research Databases  Search Screen - Advanced Search  Database - CINAHL Ultimate \| 6,762 \| \| S147 \| inequit* \| Expanders - Apply equivalent subjects  Search modes - Boolean/Phrase \| Interface - EBSCOhost Research Databases  Search Screen - Advanced Search  Database - CINAHL Ultimate \| 11,611 \| \| S146 \| equit* \| Expanders - Apply equivalent subjects  Search modes - Boolean/Phrase \| Interface - EBSCOhost Research Databases  Search Screen - Advanced Search  Database - CINAHL Ultimate \| 22,779 \| \| S145 \| PROM? \| Expanders - Apply equivalent subjects  Search modes - Boolean/Phrase \| Interface - EBSCOhost Research Databases  Search Screen - Advanced Search  Database - CINAHL Ultimate \| 3,020 \| \| S144 \| (report* N3 outcome?) \| Expanders - Apply equivalent subjects  Search modes - Boolean/Phrase \| Interface - EBSCOhost Research Databases  Search Screen - Advanced Search  Database - CINAHL Ultimate \| 44,772 \| \| S143 \| evaluation? \| Expanders - Apply equivalent subjects  Search modes - Boolean/Phrase \| Interface - EBSCOhost Research Databases  Search Screen - Advanced Search  Database - CINAHL Ultimate \| 1,153,253 \| \| S142 \| (integrat* N3 evaluat*) \| Expanders - Apply equivalent subjects  Search modes - Boolean/Phrase \| Interface - EBSCOhost Research Databases  Search Screen - Advanced Search  Database - CINAHL Ultimate \| 2,928 \| \| S141 \| ((framework? or frame-work?) N3 evaluat*) \| Expanders - Apply equivalent subjects  Search modes - Boolean/Phrase \| Interface - EBSCOhost Research Databases  Search Screen - Advanced Search  Database - CINAHL Ultimate \| 3,899 \| \| S140 \| (care? N3 evaluat*) \| Expanders - Apply equivalent subjects  Search modes - Boolean/Phrase \| Interface - EBSCOhost Research Databases  Search Screen - Advanced Search  Database - CINAHL Ultimate \| 38,487 \| \| S139 \| (outcome? N3 evaluat*) \| Expanders - Apply equivalent subjects  Search modes - Boolean/Phrase \| Interface - EBSCOhost Research Databases  Search Screen - Advanced Search  Database - CINAHL Ultimate \| 55,973 \| \| S138 \| (effective* N3 evaluat*) \| Expanders - Apply equivalent subjects  Search modes - Boolean/Phrase \| Interface - EBSCOhost Research Databases  Search Screen - Advanced Search  Database - CINAHL Ultimate \| 29,670 \| \| S137 \| (technolog* N3 limit*) \| Expanders - Apply equivalent subjects  Search modes - Boolean/Phrase \| Interface - EBSCOhost Research Databases  Search Screen - Advanced Search  Database - CINAHL Ultimate \| 1,448 \| \| S136 \| (technolog* N3 access*) \| Expanders - Apply equivalent subjects  Search modes - Boolean/Phrase \| Interface - EBSCOhost Research Databases  Search Screen - Advanced Search  Database - CINAHL Ultimate \| 2,565 \| \| S135 \| (process* N3 evaluat*) \| Expanders - Apply equivalent subjects  Search modes - Boolean/Phrase \| Interface - EBSCOhost Research Databases  Search Screen - Advanced Search  Database - CINAHL Ultimate \| 13,315 \| \| S134 \| (process* N3 measur*) \| Expanders - Apply equivalent subjects  Search modes - Boolean/Phrase \| Interface - EBSCOhost Research Databases  Search Screen - Advanced Search  Database - CINAHL Ultimate \| 7,032 \| \| S133 \| (resource? N3 evaluat*) \| Expanders - Apply equivalent subjects  Search modes - Boolean/Phrase \| Interface - EBSCOhost Research Databases  Search Screen - Advanced Search  Database - CINAHL Ultimate \| 4,472 \| \| S132 \| (resource? N3 utili*) \| Expanders - Apply equivalent subjects  Search modes - Boolean/Phrase \| Interface - EBSCOhost Research Databases  Search Screen - Advanced Search  Database - CINAHL Ultimate \| 30,207 \| \| S131 \| (service? N3 evaluat*) \| Expanders - Apply equivalent subjects  Search modes - Boolean/Phrase \| Interface - EBSCOhost Research Databases  Search Screen - Advanced Search  Database - CINAHL Ultimate \| 14,886 \| \| S130 \| (service? N3 utili*) \| Expanders - Apply equivalent subjects  Search modes - Boolean/Phrase \| Interface - EBSCOhost Research Databases  Search Screen - Advanced Search  Database - CINAHL Ultimate \| 27,521 \| \| S129 \| (cost?? or costing) \| Expanders - Apply equivalent subjects  Search modes - Boolean/Phrase \| Interface - EBSCOhost Research Databases  Search Screen - Advanced Search  Database - CINAHL Ultimate \| 263,351 \| \| S128 \| (quality N3 tool?) \| Expanders - Apply equivalent subjects  Search modes - Boolean/Phrase \| Interface - EBSCOhost Research Databases  Search Screen - Advanced Search  Database - CINAHL Ultimate \| 5,275 \| \| S127 \| (quality N3 (framework? or frame-work?)) \| Expanders - Apply equivalent subjects  Search modes - Boolean/Phrase \| Interface - EBSCOhost Research Databases  Search Screen - Advanced Search  Database - CINAHL Ultimate \| 2,071 \| \| S126 \| (quality N3 outcome?) \| Expanders - Apply equivalent subjects  Search modes - Boolean/Phrase \| Interface - EBSCOhost Research Databases  Search Screen - Advanced Search  Database - CINAHL Ultimate \| 18,807 \| \| S125 \| (qualit* N3 evaluat*) \| Expanders - Apply equivalent subjects  Search modes - Boolean/Phrase \| Interface - EBSCOhost Research Databases  Search Screen - Advanced Search  Database - CINAHL Ultimate \| 42,742 \| \| S124 \| (qualit* N3 manag*) \| Expanders - Apply equivalent subjects  Search modes - Boolean/Phrase \| Interface - EBSCOhost Research Databases  Search Screen - Advanced Search  Database - CINAHL Ultimate \| 10,688 \| \| S123 \| (qualit* N3 measur*) \| Expanders - Apply equivalent subjects  Search modes - Boolean/Phrase \| Interface - EBSCOhost Research Databases  Search Screen - Advanced Search  Database - CINAHL Ultimate \| 28,123 \| \| S122 \| (qualit* N3 metric?) \| Expanders - Apply equivalent subjects  Search modes - Boolean/Phrase \| Interface - EBSCOhost Research Databases  Search Screen - Advanced Search  Database - CINAHL Ultimate \| 2,231 \| \| S121 \| (qualit* N3 indicat*) \| Expanders - Apply equivalent subjects  Search modes - Boolean/Phrase \| Interface - EBSCOhost Research Databases  Search Screen - Advanced Search  Database - CINAHL Ultimate \| 12,380 \| \| S120 \| (qualit* N3 improv*) \| Expanders - Apply equivalent subjects  Search modes - Boolean/Phrase \| Interface - EBSCOhost Research Databases  Search Screen - Advanced Search  Database - CINAHL Ultimate \| 139,223 \| \| S119 \| (qualit* N3 assess*) \| Expanders - Apply equivalent subjects  Search modes - Boolean/Phrase \| Interface - EBSCOhost Research Databases  Search Screen - Advanced Search  Database - CINAHL Ultimate \| 55,479 \| \| S118 \| (qualit* N3 assuran*) \| Expanders - Apply equivalent subjects  Search modes - Boolean/Phrase \| Interface - EBSCOhost Research Databases  Search Screen - Advanced Search  Database - CINAHL Ultimate \| 26,513 \| \| S117 \| (qualit* N3 care?) \| Expanders - Apply equivalent subjects  Search modes - Boolean/Phrase \| Interface - EBSCOhost Research Databases  Search Screen - Advanced Search  Database - CINAHL Ultimate \| 149,924 \| \| S116 \| (qualit* N3 health*) \| Expanders - Apply equivalent subjects  Search modes - Boolean/Phrase \| Interface - EBSCOhost Research Databases  Search Screen - Advanced Search  Database - CINAHL Ultimate \| 146,548 \| \| S115 \| (MH "Safety") \| Expanders - Apply equivalent subjects  Search modes - Boolean/Phrase \| Interface - EBSCOhost Research Databases  Search Screen - Advanced Search  Database - CINAHL Ultimate \| 31,119 \| \| S114 \| (MH "Patient Safety") \| Expanders - Apply equivalent subjects  Search modes - Boolean/Phrase \| Interface - EBSCOhost Research Databases  Search Screen - Advanced Search  Database - CINAHL Ultimate \| 73,334 \| \| S113 \| (MH "Patient Preference") \| Expanders - Apply equivalent subjects  Search modes - Boolean/Phrase \| Interface - EBSCOhost Research Databases  Search Screen - Advanced Search  Database - CINAHL Ultimate \| 2,430 \| \| S112 \| (MH "Patient Satisfaction+") \| Expanders - Apply equivalent subjects  Search modes - Boolean/Phrase \| Interface - EBSCOhost Research Databases  Search Screen - Advanced Search  Database - CINAHL Ultimate \| 63,524 \| \| S111 \| (MH "Social Class") \| Expanders - Apply equivalent subjects  Search modes - Boolean/Phrase \| Interface - EBSCOhost Research Databases  Search Screen - Advanced Search  Database - CINAHL Ultimate \| 14,059 \| \| S110 \| (MH "Social Determinants of Health") \| Expanders - Apply equivalent subjects  Search modes - Boolean/Phrase \| Interface - EBSCOhost Research Databases  Search Screen - Advanced Search  Database - CINAHL Ultimate \| 10,679 \| \| S109 \| (MH "Health Status Disparities") \| Expanders - Apply equivalent subjects  Search modes - Boolean/Phrase \| Interface - EBSCOhost Research Databases  Search Screen - Advanced Search  Database - CINAHL Ultimate \| 9,977 \| \| S108 \| (MH "Healthcare Disparities") \| Expanders - Apply equivalent subjects  Search modes - Boolean/Phrase \| Interface - EBSCOhost Research Databases  Search Screen - Advanced Search  Database - CINAHL Ultimate \| 18,874 \| \| S107 \| (MH "Health Inequities") \| Expanders - Apply equivalent subjects  Search modes - Boolean/Phrase \| Interface - EBSCOhost Research Databases  Search Screen - Advanced Search  Database - CINAHL Ultimate \| 886 \| \| S106 \| (MH "Evaluation") \| Expanders - Apply equivalent subjects  Search modes - Boolean/Phrase \| Interface - EBSCOhost Research Databases  Search Screen - Advanced Search  Database - CINAHL Ultimate \| 2,275 \| \| S105 \| (MH "Program Evaluation") \| Expanders - Apply equivalent subjects  Search modes - Boolean/Phrase \| Interface - EBSCOhost Research Databases  Search Screen - Advanced Search  Database - CINAHL Ultimate \| 48,511 \| \| S104 \| (MH "Patient-Reported Outcomes") \| Expanders - Apply equivalent subjects  Search modes - Boolean/Phrase \| Interface - EBSCOhost Research Databases  Search Screen - Advanced Search  Database - CINAHL Ultimate \| 5,737 \| \| S103 \| (MH "Health Resource Utilization") \| Expanders - Apply equivalent subjects  Search modes - Boolean/Phrase \| Interface - EBSCOhost Research Databases  Search Screen - Advanced Search  Database - CINAHL Ultimate \| 22,690 \| \| S102 \| (MH "Digital Technology") \| Expanders - Apply equivalent subjects  Search modes - Boolean/Phrase \| Interface - EBSCOhost Research Databases  Search Screen - Advanced Search  Database - CINAHL Ultimate \| 2,092 \| \| S101 \| (MH "Quality Control (Technology)") \| Expanders - Apply equivalent subjects  Search modes - Boolean/Phrase \| Interface - EBSCOhost Research Databases  Search Screen - Advanced Search  Database - CINAHL Ultimate \| 6,243 \| \| S100 \| (MH "Technology") \| Expanders - Apply equivalent subjects  Search modes - Boolean/Phrase \| Interface - EBSCOhost Research Databases  Search Screen - Advanced Search  Database - CINAHL Ultimate \| 20,712 \| \| S99 \| (MH "Health Care Costs") \| Expanders - Apply equivalent subjects  Search modes - Boolean/Phrase \| Interface - EBSCOhost Research Databases  Search Screen - Advanced Search  Database - CINAHL Ultimate \| 62,538 \| \| S98 \| (MH "Cost Control") \| Expanders - Apply equivalent subjects  Search modes - Boolean/Phrase \| Interface - EBSCOhost Research Databases  Search Screen - Advanced Search  Database - CINAHL Ultimate \| 7,291 \| \| S97 \| (MH "Cost Benefit Analysis") \| Expanders - Apply equivalent subjects  Search modes - Boolean/Phrase \| Interface - EBSCOhost Research Databases  Search Screen - Advanced Search  Database - CINAHL Ultimate \| 38,488 \| \| S96 \| (MH "Costs and Cost Analysis+") \| Expanders - Apply equivalent subjects  Search modes - Boolean/Phrase \| Interface - EBSCOhost Research Databases  Search Screen - Advanced Search  Database - CINAHL Ultimate \| 133,163 \| \| S95 \| (MH "United States Agency for Healthcare Research and Quality") \| Expanders - Apply equivalent subjects  Search modes - Boolean/Phrase \| Interface - EBSCOhost Research Databases  Search Screen - Advanced Search  Database - CINAHL Ultimate \| 3,284 \| \| S94 \| (MH "Quality Management, Organizational") \| Expanders - Apply equivalent subjects  Search modes - Boolean/Phrase \| Interface - EBSCOhost Research Databases  Search Screen - Advanced Search  Database - CINAHL Ultimate \| 1,322 \| \| S93 \| (MH "Evaluation and Quality Improvement Program") \| Expanders - Apply equivalent subjects  Search modes - Boolean/Phrase \| Interface - EBSCOhost Research Databases  Search Screen - Advanced Search  Database - CINAHL Ultimate \| 29 \| \| S92 \| (MH "Quality Improvement") \| Expanders - Apply equivalent subjects  Search modes - Boolean/Phrase \| Interface - EBSCOhost Research Databases  Search Screen - Advanced Search  Database - CINAHL Ultimate \| 65,700 \| \| S91 \| (MH "Quality Assessment") \| Expanders - Apply equivalent subjects  Search modes - Boolean/Phrase \| Interface - EBSCOhost Research Databases  Search Screen - Advanced Search  Database - CINAHL Ultimate \| 10,833 \| \| S90 \| (MH "Quality Assurance") \| Expanders - Apply equivalent subjects  Search modes - Boolean/Phrase \| Interface - EBSCOhost Research Databases  Search Screen - Advanced Search  Database - CINAHL Ultimate \| 21,434 \| \| S89 \| (MH "Quality of Health Care") \| Expanders - Apply equivalent subjects  Search modes - Boolean/Phrase \| Interface - EBSCOhost Research Databases  Search Screen - Advanced Search  Database - CINAHL Ultimate \| 82,099 \| \| S88 \| S1 OR S2 OR S3 OR S4 OR S5 OR S6 OR S7 OR S8 OR S9 OR S10 OR S11 OR S12 OR S13 OR S14 OR S15 OR S16 OR S17 OR S18 OR S19 OR S20 OR S21 OR S22 OR S23 OR S24 OR S25 OR S26 OR S27 OR S28 OR S29 OR S30 OR S31 OR S32 OR S33 OR S34 OR S35 OR S36 OR S37 OR S38 OR S39 OR S40 OR S41 OR S42 OR S43 OR S44 OR S45 OR S46 OR S47 OR S48 OR S49 OR S50 OR S51 OR S52 OR S53 OR S54 OR S55 OR S56 OR S57 OR S58 OR S59 OR S60 OR S61 OR S62 OR S63 OR S64 OR S65 OR S66 OR S67 OR S68 OR S69 OR S70 OR S71 OR S72 OR S73 OR S74 OR S75 OR S76 OR S77 OR S78 OR S79 OR S80 OR S81 OR S82 OR S83 OR S84 OR S85 OR S86 OR S87 \| Expanders - Apply equivalent subjects  Search modes - Boolean/Phrase \| Interface - EBSCOhost Research Databases  Search Screen - Advanced Search  Database - CINAHL Ultimate \| 164,700 \| \| S87 \| (vhealth* or v-health*) \| Expanders - Apply equivalent subjects  Search modes - Boolean/Phrase \| Interface - EBSCOhost Research Databases  Search Screen - Advanced Search  Database - CINAHL Ultimate \| 41 \| \| S86 \| (uhealth* or u-health*) \| Expanders - Apply equivalent subjects  Search modes - Boolean/Phrase \| Interface - EBSCOhost Research Databases  Search Screen - Advanced Search  Database - CINAHL Ultimate \| 23 \| \| S85 \| (thealth* or t-health*) \| Expanders - Apply equivalent subjects  Search modes - Boolean/Phrase \| Interface - EBSCOhost Research Databases  Search Screen - Advanced Search  Database - CINAHL Ultimate \| 9 \| \| S84 \| (mhealth* or m-health*) \| Expanders - Apply equivalent subjects  Search modes - Boolean/Phrase \| Interface - EBSCOhost Research Databases  Search Screen - Advanced Search  Database - CINAHL Ultimate \| 21,695 \| \| S83 \| (ehealth* or e-health*) \| Expanders - Apply equivalent subjects  Search modes - Boolean/Phrase \| Interface - EBSCOhost Research Databases  Search Screen - Advanced Search  Database - CINAHL Ultimate \| 23,251 \| \| S82 \| (remote* N3 follow* up?) \| Expanders - Apply equivalent subjects  Search modes - Boolean/Phrase \| Interface - EBSCOhost Research Databases  Search Screen - Advanced Search  Database - CINAHL Ultimate \| 134 \| \| S81 \| (remote* N3 service?) \| Expanders - Apply equivalent subjects  Search modes - Boolean/Phrase \| Interface - EBSCOhost Research Databases  Search Screen - Advanced Search  Database - CINAHL Ultimate \| 1,169 \| \| S80 \| (remote* N3 (aftercare? or after care?)) \| Expanders - Apply equivalent subjects  Search modes - Boolean/Phrase \| Interface - EBSCOhost Research Databases  Search Screen - Advanced Search  Database - CINAHL Ultimate \| 2 \| \| S79 \| (remote* N3 counsel*) \| Expanders - Apply equivalent subjects  Search modes - Boolean/Phrase \| Interface - EBSCOhost Research Databases  Search Screen - Advanced Search  Database - CINAHL Ultimate \| 50 \| \| S78 \| (remote* N3 consult*) \| Expanders - Apply equivalent subjects  Search modes - Boolean/Phrase \| Interface - EBSCOhost Research Databases  Search Screen - Advanced Search  Database - CINAHL Ultimate \| 3,348 \| \| S77 \| (remote* N3 navigat*) \| Expanders - Apply equivalent subjects  Search modes - Boolean/Phrase \| Interface - EBSCOhost Research Databases  Search Screen - Advanced Search  Database - CINAHL Ultimate \| 167 \| \| S76 \| (remote* N3 care?) \| Expanders - Apply equivalent subjects  Search modes - Boolean/Phrase \| Interface - EBSCOhost Research Databases  Search Screen - Advanced Search  Database - CINAHL Ultimate \| 1,397 \| \| S75 \| (remote* N3 clinic*) \| Expanders - Apply equivalent subjects  Search modes - Boolean/Phrase \| Interface - EBSCOhost Research Databases  Search Screen - Advanced Search  Database - CINAHL Ultimate \| 804 \| \| S74 \| (remote* N3 rehab*) \| Expanders - Apply equivalent subjects  Search modes - Boolean/Phrase \| Interface - EBSCOhost Research Databases  Search Screen - Advanced Search  Database - CINAHL Ultimate \| 132 \| \| S73 \| (remote* N3 urgent care?) \| Expanders - Apply equivalent subjects  Search modes - Boolean/Phrase \| Interface - EBSCOhost Research Databases  Search Screen - Advanced Search  Database - CINAHL Ultimate \| 3 \| \| S72 \| (remote* N3 ambulator*) \| Expanders - Apply equivalent subjects  Search modes - Boolean/Phrase \| Interface - EBSCOhost Research Databases  Search Screen - Advanced Search  Database - CINAHL Ultimate \| 26 \| \| S71 \| (remote* N3 visit*) \| Expanders - Apply equivalent subjects  Search modes - Boolean/Phrase \| Interface - EBSCOhost Research Databases  Search Screen - Advanced Search  Database - CINAHL Ultimate \| 178 \| \| S70 \| (remote* N3 rad*) \| Expanders - Apply equivalent subjects  Search modes - Boolean/Phrase \| Interface - EBSCOhost Research Databases  Search Screen - Advanced Search  Database - CINAHL Ultimate \| 127 \| \| S69 \| (remote* N3 diagnos*) \| Expanders - Apply equivalent subjects  Search modes - Boolean/Phrase \| Interface - EBSCOhost Research Databases  Search Screen - Advanced Search  Database - CINAHL Ultimate \| 261 \| \| S68 \| (remote* N3 surg*) \| Expanders - Apply equivalent subjects  Search modes - Boolean/Phrase \| Interface - EBSCOhost Research Databases  Search Screen - Advanced Search  Database - CINAHL Ultimate \| 252 \| \| S67 \| (remote* N3 treat*) \| Expanders - Apply equivalent subjects  Search modes - Boolean/Phrase \| Interface - EBSCOhost Research Databases  Search Screen - Advanced Search  Database - CINAHL Ultimate \| 415 \| \| S66 \| (remote* N3 therap*) \| Expanders - Apply equivalent subjects  Search modes - Boolean/Phrase \| Interface - EBSCOhost Research Databases  Search Screen - Advanced Search  Database - CINAHL Ultimate \| 291 \| \| S65 \| (remote* N3 interven*) \| Expanders - Apply equivalent subjects  Search modes - Boolean/Phrase \| Interface - EBSCOhost Research Databases  Search Screen - Advanced Search  Database - CINAHL Ultimate \| 513 \| \| S64 \| (remote* N3 health*) \| Expanders - Apply equivalent subjects  Search modes - Boolean/Phrase \| Interface - EBSCOhost Research Databases  Search Screen - Advanced Search  Database - CINAHL Ultimate \| 1,759 \| \| S63 \| ((online? or on-line?) N3 follow* up?) \| Expanders - Apply equivalent subjects  Search modes - Boolean/Phrase \| Interface - EBSCOhost Research Databases  Search Screen - Advanced Search  Database - CINAHL Ultimate \| 277 \| \| S62 \| ((online? or on-line?) N3 service?) \| Expanders - Apply equivalent subjects  Search modes - Boolean/Phrase \| Interface - EBSCOhost Research Databases  Search Screen - Advanced Search  Database - CINAHL Ultimate \| 7,782 \| \| S61 \| ((online? or on-line?) N3 (aftercare? or after care?)) \| Expanders - Apply equivalent subjects  Search modes - Boolean/Phrase \| Interface - EBSCOhost Research Databases  Search Screen - Advanced Search  Database - CINAHL Ultimate \| 13 \| \| S60 \| ((online? or on-line?) N3 counsel*) \| Expanders - Apply equivalent subjects  Search modes - Boolean/Phrase \| Interface - EBSCOhost Research Databases  Search Screen - Advanced Search  Database - CINAHL Ultimate \| 448 \| \| S59 \| ((online? or on-line?) N3 consult*) \| Expanders - Apply equivalent subjects  Search modes - Boolean/Phrase \| Interface - EBSCOhost Research Databases  Search Screen - Advanced Search  Database - CINAHL Ultimate \| 541 \| \| S58 \| ((online? or on-line?) N3 navigat*) \| Expanders - Apply equivalent subjects  Search modes - Boolean/Phrase \| Interface - EBSCOhost Research Databases  Search Screen - Advanced Search  Database - CINAHL Ultimate \| 146 \| \| S57 \| ((online? or on-line?) N3 care?) \| Expanders - Apply equivalent subjects  Search modes - Boolean/Phrase \| Interface - EBSCOhost Research Databases  Search Screen - Advanced Search  Database - CINAHL Ultimate \| 3,789 \| \| S56 \| ((online? or on-line?) N3 clinic*) \| Expanders - Apply equivalent subjects  Search modes - Boolean/Phrase \| Interface - EBSCOhost Research Databases  Search Screen - Advanced Search  Database - CINAHL Ultimate \| 4,334 \| \| S55 \| ((online? or on-line?) N3 rehab*) \| Expanders - Apply equivalent subjects  Search modes - Boolean/Phrase \| Interface - EBSCOhost Research Databases  Search Screen - Advanced Search  Database - CINAHL Ultimate \| 224 \| \| S54 \| ((online? or on-line?) N3 urgent care?) \| Expanders - Apply equivalent subjects  Search modes - Boolean/Phrase \| Interface - EBSCOhost Research Databases  Search Screen - Advanced Search  Database - CINAHL Ultimate \| 4 \| \| S53 \| ((online? or on-line?) N3 ambulator*) \| Expanders - Apply equivalent subjects  Search modes - Boolean/Phrase \| Interface - EBSCOhost Research Databases  Search Screen - Advanced Search  Database - CINAHL Ultimate \| 51 \| \| S52 \| ((online? or on-line?) N3 visit*) \| Expanders - Apply equivalent subjects  Search modes - Boolean/Phrase \| Interface - EBSCOhost Research Databases  Search Screen - Advanced Search  Database - CINAHL Ultimate \| 477 \| \| S51 \| ((online? or on-line?) N3 rad*) \| Expanders - Apply equivalent subjects  Search modes - Boolean/Phrase \| Interface - EBSCOhost Research Databases  Search Screen - Advanced Search  Database - CINAHL Ultimate \| 2,188 \| \| S50 \| ((online? or on-line?) N3 diagnos*) \| Expanders - Apply equivalent subjects  Search modes - Boolean/Phrase \| Interface - EBSCOhost Research Databases  Search Screen - Advanced Search  Database - CINAHL Ultimate \| 1,105 \| \| S49 \| ((online? or on-line?) N3 surg*) \| Expanders - Apply equivalent subjects  Search modes - Boolean/Phrase \| Interface - EBSCOhost Research Databases  Search Screen - Advanced Search  Database - CINAHL Ultimate \| 1,216 \| \| S48 \| ((online? or on-line?) N3 treat*) \| Expanders - Apply equivalent subjects  Search modes - Boolean/Phrase \| Interface - EBSCOhost Research Databases  Search Screen - Advanced Search  Database - CINAHL Ultimate \| 19,110 \| \| S47 \| ((online? or on-line?) N3 interven*) \| Expanders - Apply equivalent subjects  Search modes - Boolean/Phrase \| Interface - EBSCOhost Research Databases  Search Screen - Advanced Search  Database - CINAHL Ultimate \| 2,956 \| \| S46 \| ((online? or on-line?) N3 therap*) \| Expanders - Apply equivalent subjects  Search modes - Boolean/Phrase \| Interface - EBSCOhost Research Databases  Search Screen - Advanced Search  Database - CINAHL Ultimate \| 12,486 \| \| S45 \| ((online? or on-line?) N3 health*) \| Expanders - Apply equivalent subjects  Search modes - Boolean/Phrase \| Interface - EBSCOhost Research Databases  Search Screen - Advanced Search  Database - CINAHL Ultimate \| 7,376 \| \| S44 \| (mobile* N3 follow* up?) \| Expanders - Apply equivalent subjects  Search modes - Boolean/Phrase \| Interface - EBSCOhost Research Databases  Search Screen - Advanced Search  Database - CINAHL Ultimate \| 50 \| \| S43 \| (mobile* N3 service?) \| Expanders - Apply equivalent subjects  Search modes - Boolean/Phrase \| Interface - EBSCOhost Research Databases  Search Screen - Advanced Search  Database - CINAHL Ultimate \| 1,153 \| \| S42 \| (mobile* N3 (aftercare? or after care?)) \| Expanders - Apply equivalent subjects  Search modes - Boolean/Phrase \| Interface - EBSCOhost Research Databases  Search Screen - Advanced Search  Database - CINAHL Ultimate \| 4 \| \| S41 \| (mobile* N3 counsel*) \| Expanders - Apply equivalent subjects  Search modes - Boolean/Phrase \| Interface - EBSCOhost Research Databases  Search Screen - Advanced Search  Database - CINAHL Ultimate \| 70 \| \| S40 \| (mobile* N3 consult*) \| Expanders - Apply equivalent subjects  Search modes - Boolean/Phrase \| Interface - EBSCOhost Research Databases  Search Screen - Advanced Search  Database - CINAHL Ultimate \| 61 \| \| S39 \| (mobile* N3 navigat*) \| Expanders - Apply equivalent subjects  Search modes - Boolean/Phrase \| Interface - EBSCOhost Research Databases  Search Screen - Advanced Search  Database - CINAHL Ultimate \| 51 \| \| S38 \| (mobile* N3 care?) \| Expanders - Apply equivalent subjects  Search modes - Boolean/Phrase \| Interface - EBSCOhost Research Databases  Search Screen - Advanced Search  Database - CINAHL Ultimate \| 1,139 \| \| S37 \| (mobile* N3 clinic*) \| Expanders - Apply equivalent subjects  Search modes - Boolean/Phrase \| Interface - EBSCOhost Research Databases  Search Screen - Advanced Search  Database - CINAHL Ultimate \| 1,033 \| \| S36 \| (mobile* N3 rehab*) \| Expanders - Apply equivalent subjects  Search modes - Boolean/Phrase \| Interface - EBSCOhost Research Databases  Search Screen - Advanced Search  Database - CINAHL Ultimate \| 94 \| \| S35 \| (mobile* N3 urgent care?) \| Expanders - Apply equivalent subjects  Search modes - Boolean/Phrase \| Interface - EBSCOhost Research Databases  Search Screen - Advanced Search  Database - CINAHL Ultimate \| 2 \| \| S34 \| (mobile* N3 ambulator*) \| Expanders - Apply equivalent subjects  Search modes - Boolean/Phrase \| Interface - EBSCOhost Research Databases  Search Screen - Advanced Search  Database - CINAHL Ultimate \| 22 \| \| S33 \| (mobile* N3 visit*) \| Expanders - Apply equivalent subjects  Search modes - Boolean/Phrase \| Interface - EBSCOhost Research Databases  Search Screen - Advanced Search  Database - CINAHL Ultimate \| 114 \| \| S32 \| (mobile* N3 rad*) \| Expanders - Apply equivalent subjects  Search modes - Boolean/Phrase \| Interface - EBSCOhost Research Databases  Search Screen - Advanced Search  Database - CINAHL Ultimate \| 273 \| \| S31 \| (mobile* N3 diagnos*) \| Expanders - Apply equivalent subjects  Search modes - Boolean/Phrase \| Interface - EBSCOhost Research Databases  Search Screen - Advanced Search  Database - CINAHL Ultimate \| 156 \| \| S30 \| (mobile* N3 surg*) \| Expanders - Apply equivalent subjects  Search modes - Boolean/Phrase \| Interface - EBSCOhost Research Databases  Search Screen - Advanced Search  Database - CINAHL Ultimate \| 195 \| \| S29 \| (mobile* N3 treat*) \| Expanders - Apply equivalent subjects  Search modes - Boolean/Phrase \| Interface - EBSCOhost Research Databases  Search Screen - Advanced Search  Database - CINAHL Ultimate \| 353 \| \| S28 \| (mobile* N3 therap*) \| Expanders - Apply equivalent subjects  Search modes - Boolean/Phrase \| Interface - EBSCOhost Research Databases  Search Screen - Advanced Search  Database - CINAHL Ultimate \| 184 \| \| S27 \| (mobile* N3 interven*) \| Expanders - Apply equivalent subjects  Search modes - Boolean/Phrase \| Interface - EBSCOhost Research Databases  Search Screen - Advanced Search  Database - CINAHL Ultimate \| 1,431 \| \| S26 \| (mobile* N3 health*) \| Expanders - Apply equivalent subjects  Search modes - Boolean/Phrase \| Interface - EBSCOhost Research Databases  Search Screen - Advanced Search  Database - CINAHL Ultimate \| 6,441 \| \| S25 \| (tele*follow* up? or (tele* N3 follow* up?)) \| Expanders - Apply equivalent subjects  Search modes - Boolean/Phrase \| Interface - EBSCOhost Research Databases  Search Screen - Advanced Search  Database - CINAHL Ultimate \| 1,916 \| \| S24 \| (tele*service? or (tele* N3 service?)) \| Expanders - Apply equivalent subjects  Search modes - Boolean/Phrase \| Interface - EBSCOhost Research Databases  Search Screen - Advanced Search  Database - CINAHL Ultimate \| 7,727 \| \| S23 \| (tele* N3 (aftercare? or after care?)) \| Expanders - Apply equivalent subjects  Search modes - Boolean/Phrase \| Interface - EBSCOhost Research Databases  Search Screen - Advanced Search  Database - CINAHL Ultimate \| 30 \| \| S22 \| (tele*counsel* or (tele* N3 counsel*)) \| Expanders - Apply equivalent subjects  Search modes - Boolean/Phrase \| Interface - EBSCOhost Research Databases  Search Screen - Advanced Search  Database - CINAHL Ultimate \| 1,318 \| \| S21 \| (tele*consult* or (tele* N3 consult*)) \| Expanders - Apply equivalent subjects  Search modes - Boolean/Phrase \| Interface - EBSCOhost Research Databases  Search Screen - Advanced Search  Database - CINAHL Ultimate \| 2,505 \| \| S20 \| (tele*navigat* or (tele* N3 navigat*)) \| Expanders - Apply equivalent subjects  Search modes - Boolean/Phrase \| Interface - EBSCOhost Research Databases  Search Screen - Advanced Search  Database - CINAHL Ultimate \| 91 \| \| S19 \| (tele*care? or (tele* N3 care?)) \| Expanders - Apply equivalent subjects  Search modes - Boolean/Phrase \| Interface - EBSCOhost Research Databases  Search Screen - Advanced Search  Database - CINAHL Ultimate \| 5,397 \| \| S18 \| (tele*clinic* or (tele* N3 clinic*)) \| Expanders - Apply equivalent subjects  Search modes - Boolean/Phrase \| Interface - EBSCOhost Research Databases  Search Screen - Advanced Search  Database - CINAHL Ultimate \| 2,571 \| \| S17 \| (tele*rehab* or (tele* N3 rehab*)) \| Expanders - Apply equivalent subjects  Search modes - Boolean/Phrase \| Interface - EBSCOhost Research Databases  Search Screen - Advanced Search  Database - CINAHL Ultimate \| 1,478 \| \| S16 \| (tele* N3 urgent care?) \| Expanders - Apply equivalent subjects  Search modes - Boolean/Phrase \| Interface - EBSCOhost Research Databases  Search Screen - Advanced Search  Database - CINAHL Ultimate \| 34 \| \| S15 \| (tele* N3 ambulator*) \| Expanders - Apply equivalent subjects  Search modes - Boolean/Phrase \| Interface - EBSCOhost Research Databases  Search Screen - Advanced Search  Database - CINAHL Ultimate \| 123 \| \| S14 \| (tele*visit* or (tele* N3 visit*)) \| Expanders - Apply equivalent subjects  Search modes - Boolean/Phrase \| Interface - EBSCOhost Research Databases  Search Screen - Advanced Search  Database - CINAHL Ultimate \| 2,053 \| \| S13 \| (tele*rad* or (tele* N3 rad*)) \| Expanders - Apply equivalent subjects  Search modes - Boolean/Phrase \| Interface - EBSCOhost Research Databases  Search Screen - Advanced Search  Database - CINAHL Ultimate \| 1,815 \| \| S12 \| (tele*diagnos* or (tele* N3 diagnos*)) \| Expanders - Apply equivalent subjects  Search modes - Boolean/Phrase \| Interface - EBSCOhost Research Databases  Search Screen - Advanced Search  Database - CINAHL Ultimate \| 723 \| \| S11 \| (tele*surg* or (tele* N3 surg*)) \| Expanders - Apply equivalent subjects  Search modes - Boolean/Phrase \| Interface - EBSCOhost Research Databases  Search Screen - Advanced Search  Database - CINAHL Ultimate \| 692 \| \| S10 \| (tele*treat* or (tele* N3 treat*)) \| Expanders - Apply equivalent subjects  Search modes - Boolean/Phrase \| Interface - EBSCOhost Research Databases  Search Screen - Advanced Search  Database - CINAHL Ultimate \| 1,272 \| \| S9 \| (tele*therap* or (tele* N3 therap*)) \| Expanders - Apply equivalent subjects  Search modes - Boolean/Phrase \| Interface - EBSCOhost Research Databases  Search Screen - Advanced Search  Database - CINAHL Ultimate \| 1,163 \| \| S8 \| (tele*interven* or (tele* N3 interven*)) \| Expanders - Apply equivalent subjects  Search modes - Boolean/Phrase \| Interface - EBSCOhost Research Databases  Search Screen - Advanced Search  Database - CINAHL Ultimate \| 3,152 \| \| S7 \| (tele*health* or tele*-health*) \| Expanders - Apply equivalent subjects  Search modes - Boolean/Phrase \| Interface - EBSCOhost Research Databases  Search Screen - Advanced Search  Database - CINAHL Ultimate \| 18,202 \| \| S6 \| (tele*med* or tele*-med*) \| Expanders - Apply equivalent subjects  Search modes - Boolean/Phrase \| Interface - EBSCOhost Research Databases  Search Screen - Advanced Search  Database - CINAHL Ultimate \| 19,631 \| \| S5 \| virtual* \| Expanders - Apply equivalent subjects  Search modes - Boolean/Phrase \| Interface - EBSCOhost Research Databases  Search Screen - Advanced Search  Database - CINAHL Ultimate \| 47,748 \| \| S4 \| (MH "Internet-Based Intervention") \| Expanders - Apply equivalent subjects  Search modes - Boolean/Phrase \| Interface - EBSCOhost Research Databases  Search Screen - Advanced Search  Database - CINAHL Ultimate \| 769 \| \| S3 \| (MH "Remote Consultation") \| Expanders - Apply equivalent subjects  Search modes - Boolean/Phrase \| Interface - EBSCOhost Research Databases  Search Screen - Advanced Search  Database - CINAHL Ultimate \| 3,069 \| \| S2 \| (MH "Telemedicine+") \| Expanders - Apply equivalent subjects  Search modes - Boolean/Phrase \| Interface - EBSCOhost Research Databases  Search Screen - Advanced Search  Database - CINAHL Ultimate \| 19,442 \| \| S1 \| (MH "Telehealth+") \| Expanders - Apply equivalent subjects  Search modes - Boolean/Phrase \| Interface - EBSCOhost Research Databases  Search Screen - Advanced Search  Database - CINAHL Ultimate \| 34,677 \| |
| --- | --- | --- | --- | --- | --- | --- | --- | --- | --- | --- | --- | --- | --- | --- | --- | --- | --- | --- | --- | --- | --- | --- | --- | --- | --- | --- | --- | --- | --- | --- | --- | --- | --- | --- | --- | --- | --- | --- | --- | --- | --- | --- | --- | --- | --- | --- | --- | --- | --- | --- | --- | --- | --- | --- | --- | --- | --- | --- | --- | --- | --- | --- | --- | --- | --- | --- | --- | --- | --- | --- | --- | --- | --- | --- | --- | --- | --- | --- | --- | --- | --- | --- | --- | --- | --- | --- | --- | --- | --- | --- | --- | --- | --- | --- | --- | --- | --- | --- | --- | --- | --- | --- | --- | --- | --- | --- | --- | --- | --- | --- | --- | --- | --- | --- | --- | --- | --- | --- | --- | --- | --- | --- | --- | --- | --- | --- | --- | --- | --- | --- | --- | --- | --- | --- | --- | --- | --- | --- | --- | --- | --- | --- | --- | --- | --- | --- | --- | --- | --- | --- | --- | --- | --- | --- | --- | --- | --- | --- | --- | --- | --- | --- | --- | --- | --- | --- | --- | --- | --- | --- | --- | --- | --- | --- | --- | --- | --- | --- | --- | --- | --- | --- | --- | --- | --- | --- | --- | --- | --- | --- | --- | --- | --- | --- | --- | --- | --- | --- | --- | --- | --- | --- | --- | --- | --- | --- | --- | --- | --- | --- | --- | --- | --- | --- | --- | --- | --- | --- | --- | --- | --- | --- | --- | --- | --- | --- | --- | --- | --- | --- | --- | --- | --- | --- | --- | --- | --- | --- | --- | --- | --- | --- | --- | --- | --- | --- | --- | --- | --- | --- | --- | --- | --- | --- | --- | --- | --- | --- | --- | --- | --- | --- | --- | --- | --- | --- | --- | --- | --- | --- | --- | --- | --- | --- | --- | --- | --- | --- | --- | --- | --- | --- | --- | --- | --- | --- | --- | --- | --- | --- | --- | --- | --- | --- | --- | --- | --- | --- | --- | --- | --- | --- | --- | --- | --- | --- | --- | --- | --- | --- | --- | --- | --- | --- | --- | --- | --- | --- | --- | --- | --- | --- | --- | --- | --- | --- | --- | --- | --- | --- | --- | --- | --- | --- | --- | --- | --- | --- | --- | --- | --- | --- | --- | --- | --- | --- | --- | --- | --- | --- | --- | --- | --- | --- | --- | --- | --- | --- | --- | --- | --- | --- | --- | --- | --- | --- | --- | --- | --- | --- | --- | --- | --- | --- | --- | --- | --- | --- | --- | --- | --- | --- | --- | --- | --- | --- | --- | --- | --- | --- | --- | --- | --- | --- | --- | --- | --- | --- | --- | --- | --- | --- | --- | --- | --- | --- | --- | --- | --- | --- | --- | --- | --- | --- | --- | --- | --- | --- | --- | --- | --- | --- | --- | --- | --- | --- | --- | --- | --- | --- | --- | --- | --- | --- | --- | --- | --- | --- | --- | --- | --- | --- | --- | --- | --- | --- | --- | --- | --- | --- | --- | --- | --- | --- | --- | --- | --- | --- | --- | --- | --- | --- | --- | --- | --- | --- | --- | --- | --- | --- | --- | --- | --- | --- | --- | --- | --- | --- | --- | --- | --- | --- | --- | --- | --- | --- | --- | --- | --- | --- | --- | --- | --- | --- | --- | --- | --- | --- | --- | --- | --- | --- | --- | --- | --- | --- | --- | --- | --- | --- | --- | --- | --- | --- | --- | --- | --- | --- | --- | --- | --- | --- | --- | --- | --- | --- | --- | --- | --- | --- | --- | --- | --- | --- | --- | --- | --- | --- | --- | --- | --- | --- | --- | --- | --- | --- | --- | --- | --- | --- | --- | --- | --- | --- | --- | --- | --- | --- | --- | --- | --- | --- | --- | --- | --- | --- | --- | --- | --- | --- | --- | --- | --- | --- | --- | --- | --- | --- | --- | --- | --- | --- | --- | --- | --- | --- | --- | --- | --- | --- | --- | --- | --- | --- | --- | --- | --- | --- | --- | --- | --- | --- | --- | --- | --- | --- | --- | --- | --- | --- | --- | --- | --- | --- | --- | --- | --- | --- | --- | --- | --- | --- | --- | --- | --- | --- | --- | --- | --- | --- | --- | --- | --- | --- | --- | --- | --- | --- | --- | --- | --- | --- | --- | --- | --- | --- | --- | --- | --- | --- | --- | --- | --- | --- | --- | --- | --- | --- | --- | --- | --- | --- | --- | --- | --- | --- | --- | --- | --- | --- | --- | --- | --- | --- | --- | --- | --- | --- | --- | --- | --- | --- | --- | --- | --- | --- | --- | --- | --- | --- | --- | --- | --- | --- | --- | --- | --- | --- | --- | --- | --- | --- | --- | --- | --- | --- | --- | --- | --- | --- | --- | --- | --- | --- | --- | --- | --- | --- | --- | --- | --- | --- | --- | --- | --- | --- | --- | --- | --- | --- | --- | --- | --- | --- | --- | --- | --- | --- | --- | --- | --- | --- | --- | --- | --- | --- | --- | --- | --- | --- | --- | --- | --- | --- | --- | --- | --- | --- | --- | --- | --- | --- | --- | --- | --- | --- | --- | --- | --- | --- | --- | --- | --- | --- | --- | --- | --- | --- | --- | --- | --- | --- | --- | --- | --- | --- | --- | --- | --- | --- | --- | --- | --- | --- | --- | --- | --- | --- | --- | --- |

**Ovid Emcare 2015 to May 10, 2023**

| **#** | **Searches** | **Results** | **Type** | |  |  |  |
| --- | --- | --- | --- | --- | --- | --- | --- |
|  | | | | | | | |
| 1 | exp telehealth/ | 25839 | Advanced |  | |  |  |
| 2 | exp telemedicine/ | 18529 | Advanced |  | |  |  |
| 3 | telecare/ | 235 | Advanced |  | |  |  |
| 4 | exp teleconsultation/ | 4282 | Advanced |  | |  |  |
| 5 | electronic consultation/ | 64 | Advanced |  | |  |  |
| 6 | e-counseling/ | 149 | Advanced |  | |  |  |
| 7 | web-based intervention/ | 696 | Advanced |  | |  |  |
| 8 | virtual*.tw,kf. | 53126 | Advanced |  | |  |  |
| 9 | (tele*med* or tele*-med*).tw,kf. | 15573 | Advanced |  | |  |  |
| 10 | (tele*health* or tele*-health*).tw,kf. | 9749 | Advanced |  | |  |  |
| 11 | (tele*interven* or (tele* adj3 interven*)).tw,kf. | 2899 | Advanced |  | |  |  |
| 12 | (tele*therap* or (tele* adj3 therap*)).tw,kf. | 1080 | Advanced |  | |  |  |
| 13 | (tele*treat* or (tele* adj3 treat*)).tw,kf. | 1058 | Advanced |  | |  |  |
| 14 | (tele*surg* or (tele* adj3 surg*)).tw,kf. | 866 | Advanced |  | |  |  |
| 15 | (tele*diagnos* or (tele* adj3 diagnos*)).tw,kf. | 822 | Advanced |  | |  |  |
| 16 | (tele*rad* or (tele* adj3 rad*)).tw,kf. | 2056 | Advanced |  | |  |  |
| 17 | (tele*visit* or (tele* adj3 visit*)).tw,kf. | 1898 | Advanced |  | |  |  |
| 18 | (tele* adj3 ambulator*).tw,kf. | 90 | Advanced |  | |  |  |
| 19 | (tele* adj3 urgent care?).tw,kf. | 35 | Advanced |  | |  |  |
| 20 | (tele*rehab* or (tele* adj3 rehab*)).tw,kf. | 1473 | Advanced |  | |  |  |
| 21 | (tele*clinic* or (tele* adj3 clinic*)).tw,kf. | 2259 | Advanced |  | |  |  |
| 22 | (tele*care? or (tele* adj3 care?)).tw,kf. | 4784 | Advanced |  | |  |  |
| 23 | (tele*navigat* or (tele* adj3 navigat*)).tw,kf. | 70 | Advanced |  | |  |  |
| 24 | (tele*consult* or (tele* adj3 consult*)).tw,kf. | 3423 | Advanced |  | |  |  |
| 25 | (tele*counsel* or (tele* adj3 counsel*)).tw,kf. | 1309 | Advanced |  | |  |  |
| 26 | (tele* adj3 (aftercare? or after care?)).tw,kf. | 38 | Advanced |  | |  |  |
| 27 | (tele*service? or (tele* adj3 service?)).tw,kf. | 4735 | Advanced |  | |  |  |
| 28 | (tele*follow* up? or (tele* adj3 follow* up?)).tw,kf. | 3683 | Advanced |  | |  |  |
| 29 | (mobile* adj3 health*).tw,kf. | 5424 | Advanced |  | |  |  |
| 30 | (mobile* adj3 interven*).tw,kf. | 1524 | Advanced |  | |  |  |
| 31 | (mobile* adj3 therap*).tw,kf. | 153 | Advanced |  | |  |  |
| 32 | (mobile* adj3 treat*).tw,kf. | 334 | Advanced |  | |  |  |
| 33 | (mobile* adj3 surg*).tw,kf. | 163 | Advanced |  | |  |  |
| 34 | (mobile* adj3 diagnos*).tw,kf. | 129 | Advanced |  | |  |  |
| 35 | (mobile* adj3 rad*).tw,kf. | 347 | Advanced |  | |  |  |
| 36 | (mobile* adj3 visit*).tw,kf. | 113 | Advanced |  | |  |  |
| 37 | (mobile* adj3 ambulator*).tw,kf. | 19 | Advanced |  | |  |  |
| 38 | (mobile* adj3 urgent care?).tw,kf. | 5 | Advanced |  | |  |  |
| 39 | (mobile* adj3 rehab*).tw,kf. | 83 | Advanced |  | |  |  |
| 40 | (mobile* adj3 clinic*).tw,kf. | 868 | Advanced |  | |  |  |
| 41 | (mobile* adj3 care?).tw,kf. | 1080 | Advanced |  | |  |  |
| 42 | (mobile* adj3 navigat*).tw,kf. | 50 | Advanced |  | |  |  |
| 43 | (mobile* adj3 consult*).tw,kf. | 58 | Advanced |  | |  |  |
| 44 | (mobile* adj3 counsel*).tw,kf. | 77 | Advanced |  | |  |  |
| 45 | (mobile* adj3 (aftercare? or after care?)).tw,kf. | 6 | Advanced |  | |  |  |
| 46 | (mobile* adj3 service?).tw,kf. | 1007 | Advanced |  | |  |  |
| 47 | (mobile* adj3 follow* up?).tw,kf. | 74 | Advanced |  | |  |  |
| 48 | ((online? or on-line?) adj3 health*).tw,kf. | 3948 | Advanced |  | |  |  |
| 49 | ((online? or on-line?) adj3 interven*).tw,kf. | 2273 | Advanced |  | |  |  |
| 50 | ((online? or on-line?) adj3 therap*).tw,kf. | 799 | Advanced |  | |  |  |
| 51 | ((online? or on-line?) adj3 treat*).tw,kf. | 692 | Advanced |  | |  |  |
| 52 | ((online? or on-line?) adj3 surg*).tw,kf. | 240 | Advanced |  | |  |  |
| 53 | ((online? or on-line?) adj3 diagnos*).tw,kf. | 224 | Advanced |  | |  |  |
| 54 | ((online? or on-line?) adj3 rad*).tw,kf. | 374 | Advanced |  | |  |  |
| 55 | ((online? or on-line?) adj3 visit*).tw,kf. | 212 | Advanced |  | |  |  |
| 56 | ((online? or on-line?) adj3 ambulator*).tw,kf. | 9 | Advanced |  | |  |  |
| 57 | ((online? or on-line?) adj3 urgent care?).tw,kf. | 1 | Advanced |  | |  |  |
| 58 | ((online? or on-line?) adj3 rehab*).tw,kf. | 100 | Advanced |  | |  |  |
| 59 | ((online? or on-line?) adj3 clinic*).tw,kf. | 1157 | Advanced |  | |  |  |
| 60 | ((online? or on-line?) adj3 care?).tw,kf. | 892 | Advanced |  | |  |  |
| 61 | ((online? or on-line?) adj3 navigat*).tw,kf. | 101 | Advanced |  | |  |  |
| 62 | ((online? or on-line?) adj3 consult*).tw,kf. | 494 | Advanced |  | |  |  |
| 63 | ((online? or on-line?) adj3 counsel*).tw,kf. | 327 | Advanced |  | |  |  |
| 64 | ((online? or on-line?) adj3 (aftercare? or after care?)).tw,kf. | 13 | Advanced |  | |  |  |
| 65 | ((online? or on-line?) adj3 service?).tw,kf. | 1511 | Advanced |  | |  |  |
| 66 | ((online? or on-line?) adj3 follow* up?).tw,kf. | 285 | Advanced |  | |  |  |
| 67 | (remote* adj3 health*).tw,kf. | 1581 | Advanced |  | |  |  |
| 68 | (remote* adj3 interven*).tw,kf. | 516 | Advanced |  | |  |  |
| 69 | (remote* adj3 therap*).tw,kf. | 292 | Advanced |  | |  |  |
| 70 | (remote* adj3 treat*).tw,kf. | 442 | Advanced |  | |  |  |
| 71 | (remote* adj3 surg*).tw,kf. | 317 | Advanced |  | |  |  |
| 72 | (remote* adj3 diagnos*).tw,kf. | 436 | Advanced |  | |  |  |
| 73 | (remote* adj3 rad*).tw,kf. | 185 | Advanced |  | |  |  |
| 74 | (remote* adj3 visit*).tw,kf. | 154 | Advanced |  | |  |  |
| 75 | (remote* adj3 ambulator*).tw,kf. | 24 | Advanced |  | |  |  |
| 76 | (remote* adj3 urgent care?).tw,kf. | 3 | Advanced |  | |  |  |
| 77 | (remote* adj3 rehab*).tw,kf. | 158 | Advanced |  | |  |  |
| 78 | (remote* adj3 clinic*).tw,kf. | 774 | Advanced |  | |  |  |
| 79 | (remote* adj3 care?).tw,kf. | 1273 | Advanced |  | |  |  |
| 80 | (remote* adj3 navigat*).tw,kf. | 206 | Advanced |  | |  |  |
| 81 | (remote* adj3 consult*).tw,kf. | 898 | Advanced |  | |  |  |
| 82 | (remote* adj3 counsel*).tw,kf. | 67 | Advanced |  | |  |  |
| 83 | (remote* adj3 (aftercare? or after care?)).tw,kf. | 0 | Advanced |  | |  |  |
| 84 | (remote* adj3 service?).tw,kf. | 1027 | Advanced |  | |  |  |
| 85 | (remote* adj3 follow* up?).tw,kf. | 211 | Advanced |  | |  |  |
| 86 | (ehealth* or e-health*).tw,kf. | 8009 | Advanced |  | |  |  |
| 87 | (mhealth* or m-health*).tw,kf. | 5281 | Advanced |  | |  |  |
| 88 | (thealth* or t-health*).tw,kf. | 11 | Advanced |  | |  |  |
| 89 | (uhealth* or u-health*).tw,kf. | 46 | Advanced |  | |  |  |
| 90 | (vhealth* or v-health*).tw,kf. | 35 | Advanced |  | |  |  |
| 91 | or/1-90 | 122432 | Advanced |  | |  |  |
| 92 | health care quality/ | 77475 | Advanced |  | |  |  |
| 93 | performance measurement system/ | 1708 | Advanced |  | |  |  |
| 94 | "healthcare access and quality index"/ | 1 | Advanced |  | |  |  |
| 95 | performance indicator/ | 400 | Advanced |  | |  |  |
| 96 | quality control/ | 45307 | Advanced |  | |  |  |
| 97 | quality control procedures/ | 321 | Advanced |  | |  |  |
| 98 | total quality management/ | 26997 | Advanced |  | |  |  |
| 99 | quality improvement study/ | 201 | Advanced |  | |  |  |
| 100 | quality by design/ | 28 | Advanced |  | |  |  |
| 101 | "cost"/ | 5400 | Advanced |  | |  |  |
| 102 | exp "health care cost"/ | 97473 | Advanced |  | |  |  |
| 103 | health care financing/ | 6121 | Advanced |  | |  |  |
| 104 | health economics/ | 10160 | Advanced |  | |  |  |
| 105 | "cost benefit analysis"/ | 22522 | Advanced |  | |  |  |
| 106 | "cost control"/ | 20498 | Advanced |  | |  |  |
| 107 | "cost effectiveness analysis"/ | 48607 | Advanced |  | |  |  |
| 108 | technology/ | 52991 | Advanced |  | |  |  |
| 109 | digital technology/ | 740 | Advanced |  | |  |  |
| 110 | wireless communication/ | 1307 | Advanced |  | |  |  |
| 111 | exp health care utilization/ | 31998 | Advanced |  | |  |  |
| 112 | "facilities and services utilization"/ | 8 | Advanced |  | |  |  |
| 113 | "procedures and techniques utilization"/ | 4 | Advanced |  | |  |  |
| 114 | patient-reported outcome/ | 6222 | Advanced |  | |  |  |
| 115 | exp program evaluation/ | 7379 | Advanced |  | |  |  |
| 116 | "program cost effectiveness"/ | 235 | Advanced |  | |  |  |
| 117 | evaluation study/ | 4841 | Advanced |  | |  |  |
| 118 | health equity/ | 3261 | Advanced |  | |  |  |
| 119 | health disparity/ | 11261 | Advanced |  | |  |  |
| 120 | health care disparity/ | 4570 | Advanced |  | |  |  |
| 121 | social inequality/ | 177 | Advanced |  | |  |  |
| 122 | exp socioeconomic vulnerability/ | 116 | Advanced |  | |  |  |
| 123 | "social determinants of health"/ | 6630 | Advanced |  | |  |  |
| 124 | social class/ | 7731 | Advanced |  | |  |  |
| 125 | patient satisfaction/ | 45087 | Advanced |  | |  |  |
| 126 | patient preference/ | 5324 | Advanced |  | |  |  |
| 127 | patient safety/ | 45066 | Advanced |  | |  |  |
| 128 | patient safety indicator/ | 12 | Advanced |  | |  |  |
| 129 | safety/ | 76234 | Advanced |  | |  |  |
| 130 | (qualit* adj3 health*).tw,kf. | 72512 | Advanced |  | |  |  |
| 131 | (qualit* adj3 care?).tw,kf. | 71365 | Advanced |  | |  |  |
| 132 | (qualit* adj3 assuran*).tw,kf. | 12659 | Advanced |  | |  |  |
| 133 | (qualit* adj3 assess*).tw,kf. | 53446 | Advanced |  | |  |  |
| 134 | (qualit* adj3 improv*).tw,kf. | 105598 | Advanced |  | |  |  |
| 135 | (qualit* adj3 indicat*).tw,kf. | 14317 | Advanced |  | |  |  |
| 136 | (qualit* adj3 metric?).tw,kf. | 2573 | Advanced |  | |  |  |
| 137 | (qualit* adj3 measur*).tw,kf. | 27145 | Advanced |  | |  |  |
| 138 | (qualit* adj3 manag*).tw,kf. | 9233 | Advanced |  | |  |  |
| 139 | (qualit* adj3 evaluat*).tw,kf. | 26926 | Advanced |  | |  |  |
| 140 | (quality adj3 outcome?).tw,kf. | 15534 | Advanced |  | |  |  |
| 141 | (quality adj3 (framework? or frame-work?)).tw,kf. | 1673 | Advanced |  | |  |  |
| 142 | (quality adj3 tool?).tw,kf. | 4829 | Advanced |  | |  |  |
| 143 | (cost?? or costing).tw,kf. | 272143 | Advanced |  | |  |  |
| 144 | (service? adj3 utili*).tw,kf. | 12862 | Advanced |  | |  |  |
| 145 | (service? adj3 evaluat*).tw,kf. | 6437 | Advanced |  | |  |  |
| 146 | (resource? adj3 utili*).tw,kf. | 9832 | Advanced |  | |  |  |
| 147 | (resource? adj3 evaluat*).tw,kf. | 1737 | Advanced |  | |  |  |
| 148 | (process* adj3 measur*).tw,kf. | 8160 | Advanced |  | |  |  |
| 149 | (process* adj3 evaluat*).tw,kf. | 13081 | Advanced |  | |  |  |
| 150 | (technolog* adj3 access*).tw,kf. | 2230 | Advanced |  | |  |  |
| 151 | (technolog* adj3 limit*).tw,kf. | 1721 | Advanced |  | |  |  |
| 152 | (effective* adj3 evaluat*).tw,kf. | 34252 | Advanced |  | |  |  |
| 153 | (outcome? adj3 evaluat*).tw,kf. | 33155 | Advanced |  | |  |  |
| 154 | (care? adj3 evaluat*).tw,kf. | 10540 | Advanced |  | |  |  |
| 155 | ((framework? or frame-work?) adj3 evaluat*).tw,kf. | 4215 | Advanced |  | |  |  |
| 156 | (integrat* adj3 evaluat*).tw,kf. | 2394 | Advanced |  | |  |  |
| 157 | evaluation?.tw,kf. | 518606 | Advanced |  | |  |  |
| 158 | (report* adj3 outcome?).tw,kf. | 43998 | Advanced |  | |  |  |
| 159 | PROM?.tw,kf. | 4134 | Advanced |  | |  |  |
| 160 | equit*.tw,kf. | 27459 | Advanced |  | |  |  |
| 161 | inequit*.tw,kf. | 13474 | Advanced |  | |  |  |
| 162 | equalit*.tw,kf. | 7305 | Advanced |  | |  |  |
| 163 | inequalit*.tw,kf. | 30168 | Advanced |  | |  |  |
| 164 | dispar*.tw,kf. | 61476 | Advanced |  | |  |  |
| 165 | "social* determin* of health?".tw,kf. | 8376 | Advanced |  | |  |  |
| 166 | (social* adj3 gradient*).tw,kf. | 901 | Advanced |  | |  |  |
| 167 | satisf*.tw,kf. | 186270 | Advanced |  | |  |  |
| 168 | experienc*.tw,kf. | 653206 | Advanced |  | |  |  |
| 169 | safet*.tw,kf. | 266656 | Advanced |  | |  |  |
| 170 | or/92-169 | 2155548 | Advanced |  | |  |  |
| 171 | 91 and 170 | 61500 | Advanced |  | |  |  |
| 172 | review/ | 306292 | Advanced |  | |  |  |
| 173 | (medline or medlars or pubmed or grateful med or CINAHL or scisearch or psychinfo or psycinfo or psychlit or psyclit or handsearch* or hand search* or manual* search* or electronic database* or bibliographic database* or embase or lilacs or scopus or web of science).mp,kw. | 185897 | Advanced |  | |  |  |
| 174 | 172 and 173 | 44707 | Advanced |  | |  |  |
| 175 | "systematic review"/ | 118600 | Advanced |  | |  |  |
| 176 | "systematic review (topic)"/ | 8634 | Advanced |  | |  |  |
| 177 | exp meta analysis/ | 57752 | Advanced |  | |  |  |
| 178 | network meta-analysis/ | 773 | Advanced |  | |  |  |
| 179 | "meta analysis (topic)"/ | 10574 | Advanced |  | |  |  |
| 180 | exp biomedical technology assessment/ | 4090 | Advanced |  | |  |  |
| 181 | high-cost technology/ | 4 | Advanced |  | |  |  |
| 182 | (cochrane or (health adj2 technology assessment) or evidence report).jw. | 13521 | Advanced |  | |  |  |
| 183 | (quantitative* adj3 (synthes* or review? or overview?)).tw,kf. | 4059 | Advanced |  | |  |  |
| 184 | (qualitative* adj3 (synthes* or review? or overview?)).tw,kf. | 8725 | Advanced |  | |  |  |
| 185 | (knowledge adj3 synthes*).tw,kf. | 1111 | Advanced |  | |  |  |
| 186 | (systematic* adj3 (synthes* or review? or overview? or study or studies)).tw,kf. | 164210 | Advanced |  | |  |  |
| 187 | (scoping adj3 (synthes* or review? or overview? or study or studies)).tw,kf. | 14379 | Advanced |  | |  |  |
| 188 | (mapping adj3 (synthes* or review? or overview? or study or studies)).tw,kf. | 2320 | Advanced |  | |  |  |
| 189 | (rapid adj3 (synthes* or review? or overview? or study or studies)).tw,kf. | 3399 | Advanced |  | |  |  |
| 190 | (umbrella adj3 (synthes* or review? or overview? or study or studies)).tw,kf. | 816 | Advanced |  | |  |  |
| 191 | ("review of review?" or "review of systematic review?").tw,kf. | 6082 | Advanced |  | |  |  |
| 192 | ("overview? of review?" or "overview? of systematic review?").tw,kf. | 825 | Advanced |  | |  |  |
| 193 | ("summar* of review?" or "summar* of systematic review?").tw,kf. | 1769 | Advanced |  | |  |  |
| 194 | ("synthes* of review?" or "synthes* of systematic review?").tw,kf. | 681 | Advanced |  | |  |  |
| 195 | (integrative* adj3 (synthes* or review? or overview? or study or studies)).tw,kf. | 6268 | Advanced |  | |  |  |
| 196 | (narrative adj3 (synthes* or review? or overview? or study or studies)).tw,kf. | 19281 | Advanced |  | |  |  |
| 197 | (methodologic* adj3 (synthes* or review? or overview? or study or studies)).tw,kf. | 9933 | Advanced |  | |  |  |
| 198 | (methodologic* adj3 (synthes* or review? or overview? or study or studies)).tw,kf. | 9933 | Advanced |  | |  |  |
| 199 | (research adj3 (synthes* or review? or overview? or study or studies)).tw,kf. | 60186 | Advanced |  | |  |  |
| 200 | (collaborative* adj3 (synthes* or review? or overview? or study or studies)).tw,kf. | 3271 | Advanced |  | |  |  |
| 201 | (metaanal* or metanal* or (meta adj2 anal*)).tw,kf. | 119616 | Advanced |  | |  |  |
| 202 | (metasummar* or (meta adj2 summar*)).tw,kf. | 750 | Advanced |  | |  |  |
| 203 | (metasynthes* or (meta adj2 synthes*)).tw,kf. | 2941 | Advanced |  | |  |  |
| 204 | (metareview* or (meta adj2 review*)).tw,kf. | 55819 | Advanced |  | |  |  |
| 205 | (metanetwork* or (meta adj2 network*)).tw,kf. | 4071 | Advanced |  | |  |  |
| 206 | (metaaggregat* or (meta adj2 aggregat*)).tw,kf. | 415 | Advanced |  | |  |  |
| 207 | (metaregression* or (meta adj2 regression*)).tw,kf. | 6629 | Advanced |  | |  |  |
| 208 | (metaethnograph* or (meta adj2 ethnograph*)).tw,kf. | 794 | Advanced |  | |  |  |
| 209 | (metanarrative* or (meta adj2 narrative*)).tw,kf. | 370 | Advanced |  | |  |  |
| 210 | (pooled adj1 anal*).tw,kf. | 6414 | Advanced |  | |  |  |
| 211 | (statistical* adj1 pooling).tw,kf. | 260 | Advanced |  | |  |  |
| 212 | (statistical* adj1 summar*).tw,kf. | 156 | Advanced |  | |  |  |
| 213 | (mathematical* adj1 pooling).tw,kf. | 0 | Advanced |  | |  |  |
| 214 | (mathematical* adj1 summar*).tw,kf. | 14 | Advanced |  | |  |  |
| 215 | (data adj3 (synthes* or extraction* or abstraction*)).tw,kf. | 30671 | Advanced |  | |  |  |
| 216 | (mantel haenszel or peto or der simonian or dersimonian or fixed effect* or latin square*).tw,kf. | 13250 | Advanced |  | |  |  |
| 217 | (comparative adj3 (efficacy or effectiveness)).tw,kf. | 7416 | Advanced |  | |  |  |
| 218 | (outcomes research or relative effectiveness).tw,kf. | 6767 | Advanced |  | |  |  |
| 219 | ((indirect or indirect treatment?) adj3 comparison*).tw,kf. | 1348 | Advanced |  | |  |  |
| 220 | (mixed adj3 treatment? adj3 comparison*).tw,kf. | 262 | Advanced |  | |  |  |
| 221 | (multi* adj3 treatment? adj3 comparison*).tw,kf. | 172 | Advanced |  | |  |  |
| 222 | (bayesian adj3 comparison*).tw,kf. | 225 | Advanced |  | |  |  |
| 223 | (multi* adj2 paramet* adj2 evidence adj2 synthesis).tw,kf. | 8 | Advanced |  | |  |  |
| 224 | ((multiparamet* or multi paramet*) adj2 evidence adj2 synthesis).tw,kf. | 19 | Advanced |  | |  |  |
| 225 | (handsearch* or hand search*).tw,kf. | 6321 | Advanced |  | |  |  |
| 226 | (technology assessment* or technology overview* or technology appraisal*).tw,kf. | 5452 | Advanced |  | |  |  |
| 227 | (HTA or HTAs).tw,kf. | 2228 | Advanced |  | |  |  |
| 228 | or/174-227 | 397475 | Advanced |  | |  |  |
| 229 | 171 and 228 | 6609 | Advanced |  | |  |  |
| 230 | (virtual* adj10 care?).tw,kf. | 2672 | Advanced |  | |  |  |
| 231 | 228 and 230 | 268 | Advanced |  | |  |  |
| 232 | 229 or 231 | 6681 | Advanced |  | |  |  |
| 233 | (exp animals/ or exp animal experiment/ or nonhuman/) not ((exp animals/ or exp animal experiment/ or nonhuman/) and exp human/) | 405630 | Advanced |  | |  |  |
| 234 | 232 not 233 | 6673 | Advanced |  | |  |  |
| 235 | limit 234 to (books or business article or chapter or conference abstract or conference paper or "conference review" or dissertation or working paper) | 140 | Advanced |  | |  |  |
| 236 | 234 not 235 | 6533 | Advanced |  | |  |  |
| 237 | limit 236 to yr="2015 -Current" | 5135 | Advanced |  | |  |  |
| 238 | limit 237 to english language | 5001 | Advanced |  | |  |  |
