# Supplementary material for: Virtual Care, What Are We Measuring and What Should We Measure? Scoping Review of Reviews
Source: J Med Internet Res. 2025 Dec 1;27:e65312. doi: 10.2196/65312 (PMC12670043; doi:10.2196/65312)
Supplement: Multimedia Appendix 2 [file jmir-v27-e65312-s002.docx]

**Multimedia Appendix 2.** Details of retained articles

| **Author and Year** | **Title** | **Disease** | **Modality** | **Implementation Outcomes** | **Service Outcomes** | **Client Outcomes** |
| --- | --- | --- | --- | --- | --- | --- |
| Aapro 2020 | Digital health for optimal supportive care in oncology: benefits, limits, and future perspectives | Cancer | Both |  |  | X |
| Abasi 2021 | Effectiveness of mobile health-based self-management application for posttransplant cares: A systematic review | Surgery | Both |  | X |  |
| Abaza 2017 | mHealth application areas and technology combinations: A comparison of literature from high and low/middle income countries | Multiple | Both | X | X |  |
| Abbaspur-Behbahani 2022 | Application of mobile health to support the elderly during the COVID-19 outbreak: A systematic review | Infectious disease | Both |  | X | X |
| Abdolkhani 2022 | The Impact of Digital Health Transformation Driven by COVID-19 on Nursing Practice: Systematic Literature Review | Multiple | Both |  |  |  |
| Aboujaoude 2017 | Three decades of telemedicine in obsessive-compulsive disorder: A review across platforms | Mental health | Both |  | X | X |
| Abraham 2021 | Telemental Health Use in the COVID-19 Pandemic: A Scoping Review and Evidence Gap Mapping | Mental health | Both | X | X | X |
| Acharibasam 2018 | Telemental Health in Low- and Middle-Income Countries: A Systematic Review | Mental health | Both |  | X | X |
| Acheampong 2015 | Business models for telemedicine services: a literature review | Multiple | Both | X | X | X |
| Adams 2022 | Technology-Based Assessments and Treatments of Anxiety in Autistic Individuals: Systematic Review and Narrative Synthesis | Mental health | Video |  |  |  |
| Adawiah 2021 | Mhealth in Cardiovascular Diseases (CVD) Self-care: A Systematic Review of Advantages and Challenges | Cardiovascular | Video | X | X | X |
| Adcock 2022 | mHealth impact on secondary stroke prevention: a scoping review of randomized controlled trials among stroke survivors between 2010-2020 | Cardiovascular | Both |  | X | X |
| AgAhmed 2017 | A mixed methods systematic review of success factors of mhealth and telehealth for maternal health in Sub-Saharan Africa | Maternal health | Both | X |  | X |
| Agastiya 2022 | The impact of telehealth on self-management of patients with type 2 diabetes: A systematic review on interventional studies | Metabolic | Both |  | X | X |
| Agbali 2022 | A review of audiovisual telemedicine utilization and satisfaction assessment during the COVID-19 pandemic | Multiple | Both | X |  | X |
| Agboola 2015 | The effect of technology-based interventions on pain, depression, and quality of life in patients with cancer: A systematic review of randomized controlled trials | Cancer | Video |  |  | X |
| Agnew 2022 | An Investigation Into the Use of mHealth in Musculoskeletal Physiotherapy: Scoping Review | Pain | Video | X | X | X |
| Agostini 2015 | Telerehabilitation and recovery of motor function: a systematic review and meta-analysis | Multiple | Telephone |  |  | X |
| Ahern 2018 | Clinical efficacy and economic evaluation of online cognitive behavioral therapy for major depressive disorder: a systematic review and meta-analysis | Mental health | Telephone | X |  | X |
| AhmadiMarzaleh 2022 | Application of Telerehabilitation for Older Adults During the COVID-19 Pandemic: A Systematic Review | Infectious disease | Both |  |  | X |
| Airola 2021 | Learning and Use of eHealth Among Older Adults Living at Home in Rural and Nonrural Settings: Systematic Review | Geriatric | Video |  |  |  |
| Ajmera 2023 | Impact of telehealth interventions on physiological and psychological outcomes in breast cancer survivors: A meta-analysis of randomised controlled trials | Cancer | Both |  |  | X |
| Ajrawat 2021 | The Use of Telehealth for Orthopedic Consultations and Assessments: A Systematic Review | Musckuloskeletal | Video |  | X | X |
| Akingbade 2022 | Effect of mHealth interventions on psychological issues experienced by women undergoing chemotherapy for breast cancer: A systematic review and meta-analysis | Cancer | Telephone |  | X | X |
| Akiyama 2016 | A Systematic Review of the Economic Evaluation of Telemedicine in Japan | All health conditions | Video | X |  |  |
| Alayat 2022 | The Effectiveness of Telerehabilitation on Balance and Functional Mobility in Patients with Stroke: A Systematic Review and Meta-Analysis | Cardiovascular | Both |  |  |  |
| AlBaalharith 2022 | Telehealth and Transformation of Nursing Care in Saudi Arabia: A Systematic Review | All health conditions | Both |  | X | X |
| Albritton 2022 | Video Teleconferencing for Disease Prevention, Diagnosis, and Treatment : A Rapid Review | All health conditions | Video |  |  | X |
| Aldehaim 2016 | The Impact of Technology-Based Interventions on Informal Caregivers of Stroke Survivors: A Systematic Review | Cardiovascular | Both |  |  | X |
| AlDossary 2017 | A systematic review of the methodologies used to evaluate telemedicine service initiatives in hospital facilities | Multiple | Both |  | X | X |
| Alfuraydan 2020 | Use of telehealth for facilitating the diagnostic assessment of Autism Spectrum Disorder (ASD): A scoping review | CNS | Video | X | X | X |
| Ali 2021 | Role of the Telemental Health Services During the COVID-19 Pandemic: A Systematic Review | Mental health | Both | X | X |  |
| Alipour 2021 | Opportunities and Challenges of Telehealth in Disease Management during COVID-19 Pandemic: A Scoping Review | Multiple | Both |  |  |  |
| Aljohani 2021 | Effect of Teledentistry and Outcome for Dental Professionals at Saudi Arabia: A Systematic scoping Review | Denistry | Video | X |  |  |
| AlKhoury 2022 | Patient Perspectives and Expectations in Inflammatory Bowel Disease: A Systematic Review | GI | Video | X | X | X |
| Allam 2021 | Web-based dietary and physical activity intervention programs for Patients with Hypertension: Scoping Review | Multiple | Both | X |  | X |
| Almaqhawi 2022 | Effect of telemedicine on glycated haemoglobin in people with type 2 diabetes in the MENA region: a systematic review and meta-analysis | Metabolic | Telephone |  |  | X |
| Almasi 2022 | Efficacy of Telemedicine for the Management of Asthma: A Systematic Review | Respiratory | Both | X | X | X |
| Almathami 2020 | Barriers and facilitators that influence telemedicine-based, real-time, online consultation at patients' homes: Systematic literature review | All health conditions | Both |  |  | X |
| Almeida 2019 | An integrative review of nurse-led virtual clinics | Multiple | Telephone | X | X | X |
| Almojaibel 2016 | Delivering pulmonary rehabilitation for patients with chronic obstructive pulmonary disease at home using telehealth: A review of the literature | Respiratory | Video | X | X | X |
| Al-Naher 2022 | Factors Affecting Patient and Physician Engagement in Remote Health Care for Heart Failure: Systematic Review | Cardiovascular | Both |  | X | X |
| Alsabeeha 2023 | Older Adults' Satisfaction with Telemedicine During the COVID-19 Pandemic: A Systematic Review | Geriatric | Both | X |  | X |
| Alsaif 2022 | Virtual consultations for patients with obstructive sleep apnoea: a systematic review and meta-analysis | Respiratory | Both | X |  | X |
| Al-Samarraie 2020 | Telemedicine in Middle Eastern countries: Progress, barriers, and policy recommendations | All health conditions | Video | X |  |  |
| Alshawwa 2020 | mHealth Interventions for Cancer Care and Support A Systematic Literature Review | Cancer | Telephone | X | X | X |
| Alvarado 2017 | Barriers to remote health interventions for type 2 diabetes: A systematic review and proposed classification scheme | Metabolic | Both | X | X |  |
| Amankwaa 2018 | Effectiveness of short message services and voice call interventions for antiretroviral therapy adherence and other outcomes: A systematic review and meta-analysis | Infectious disease | Telephone |  | X | X |
| Amatya 2015 | Effectiveness of telerehabilitation interventions in persons with multiple sclerosis: A systematic review | CNS | Both |  |  | X |
| Ambegaonkar 2021 | The Use of Mobile Applications as Communication Aids for People with Dementia: Opportunities and Limitations | CNS | Telephone | X | X | X |
| Amin 2022 | Rehabilitation Professional and Patient Satisfaction with Telerehabilitation of Musculoskeletal Disorders: A Systematic Review | Musckuloskeletal | Both | X |  | X |
| Amiri 2022 | Objectives, Outcomes, Facilitators, and Barriers of Telemedicine Systems for Patients with Alzheimer's Disease and their Caregivers and Care Providers: A Systematic Review | CNS | Both | X | X | X |
| Amsah 2023 | Impact of COVID-19 Pandemic on Healthcare Utilization among Patients with Type 2 Diabetes Mellitus: A Systematic Review | Metabolic | Both | X | X | X |
| Amundson 2023 | Comprehensive medication management services provided via telehealth or hybrid models: A scoping review | Multiple | Video | X |  | X |
| Anderson 2021 | Web-Based and mHealth Interventions for Intimate Partner Violence Victimization Prevention: A Systematic Review | Assault | Video | X | X | X |
| Anderson 2022 | Telehealth Interventions to Improve Diabetes Management Among Black and Hispanic Patients: a Systematic Review and Meta-Analysis | Metabolic | Both |  |  | X |
| Andrades-Gonzalez 2021 | e-Health as a tool to improve the quality of life of informal caregivers dealing with stroke patients: Systematic review with meta-analysis | Cardiovascular | Multiple |  | X | X |
| Andre 2022 | Telephone and Smartphone-Based Interventions for Cognitive and Cardio-Metabolic Health in Middle-Aged and Older Adults: A Systematic Review | CNS | Telephone |  |  | X |
| Andrees 2020 | Live interactive teledermatology compared to in-person care - a systematic review | Dermatology | Video | X | X | X |
| Andrews 2020 | Satisfaction with the use of telehealth during COVID-19: An integrative review | All health conditions | Both |  |  | X |
| Ang 2021 | Efficacy of interventions that incorporate mobile apps in facilitating weight loss and health behavior change in the asian population: Systematic review and meta-analysis | Metabolic | Both | X | X |  |
| Anonymous 2021 | Internet-Delivered Cognitive Behavioural Therapy for Post-traumatic Stress Disorder or Acute Stress Disorder: A Health Technology Assessment | Mental health | Internet-based NOS | X |  |  |
| Ansari 2022 | How technology can improve communication and health outcomes in patients with advanced cancer: an integrative review | Cancer | Both |  | X |  |
| Appleton 2021 | Implementation, Adoption, and Perceptions of Telemental Health during the COVID-19 Pandemic: Systematic Review | Mental health | Both | X |  | X |
| Appleton 2023 | Implementation strategies for telemental health: a systematic review | Mental health | Both | X |  |  |
| AraujoDuarte 2021 | Repercussion of Telemonitoring as a Self-Care Strategy for Diabetes Mellitus People | Metabolic | Telephone |  | X | X |
| Ardito 2023 | Evaluating Barriers and Facilitators to the Uptake of mHealth Apps in Cancer Care Using the Consolidated Framework for Implementation Research: Scoping Literature Review | Cancer | Both | X | X | X |
| Aromatario 2019 | How do mobile health applications support behaviour changes? A scoping review of mobile health applications relating to physical activity and eating behaviours | Multiple | Telephone |  | X |  |
| Arshad 2020 | A Systematic Review of the Evidence Supporting Mobile- and Internet-Based Psychological Interventions For Self-Harm | Mental health | Telephone | X |  | X |
| Asiri 2018 | The Use of Telemedicine in Surgical Care: a Systematic Review | Surgery | Both | X |  | X |
| Aslani 2022 | Advantages and Challenges of Telecardiology and Providing Solutions for Its Successful Implementation: A Scoping Review | Cardiovascular | Video |  |  |  |
| Attard 2022 | How acceptable do parents experiencing mental health challenges find e-Health interventions for mental health in the postnatal period: a systematic review | Mental health | Both | X |  | X |
| Ayoub 2022 | Synchronous psychological interventions by videoconferencing for caregivers of people with dementia: an integrative review | CNS | Video | X |  | X |
| Baatiema 2023 | Health system adaptions to improve care for people living with non-communicable diseases during COVID-19 in low-middle income countries: A scoping review | Multiple | Both |  | X | X |
| Babaei 2023 | A scoping review of virtual care in the health system: infrastructures, barriers, and facilitators | Multiple | Both |  |  |  |
| Bailey 2018 | Telephone triage and midwifery: A scoping review | Maternal health | Telephone |  |  |  |
| Bailey 2021 | Early Patient-Centered Outcomes Research Experience with the Use of Telehealth to Address Disparities: Scoping Review | Multiple | Video |  |  |  |
| Baines 2018 | A Scoping Review of the Quality and the Design of Evaluations of Mobile Health, Telehealth, Smart Pump and Monitoring Technologies Performed in a Pharmacy-Related Setting | Multiple | Both | X |  | X |
| Baines 2020 | Barriers and Enablers in Implementing Electronic Consultations in Primary Care: Scoping Review | Multiple | Video |  |  |  |
| Baird 2022 | Virtual Support and Intimate Partner Violence Services: A Scoping Review | Assault | Both | X | X | X |
| Baker 2018 | Telephone-delivered psychosocial interventions targeting key health priorities in adults with a psychotic disorder: systematic review | Mental health | Telephone | X | X | X |
| Baluszek 2022 | Specialized healthcare practitioners' challenges in performing video consultations to patients in Nordic Countries - a systematic review and narrative synthesis | Multiple | Video |  |  |  |
| Banbury 2018 | Telehealth Interventions Delivering Home-based Support Group Videoconferencing: Systematic Review | Multiple | Video | X | X | X |
| Banstola 2023 | Economic evaluations of interventional opportunities for the management of mental-physical multimorbidity: a systematic review | Multiple | Telephone | X |  |  |
| Barakat 2019 | Evaluating the role of digital intervention design in treatment outcomes and adherence to eTherapy programs for eating disorders: A systematic review and meta-analysis | Mental health | Both |  | X | X |
| Baratloo 2018 | Effects of Telestroke on Thrombolysis Times and Outcomes: A Meta-analysis | Cardiovascular | Both |  | X | X |
| Barnes 2022 | "Double whammy": a rapid review of rural vs urban psychosocial cancer experiences and telehealth service in five countries during the COVID-19 pandemic | Cancer | Both | X | X | X |
| Barsom 2020 | Measuring patient satisfaction with video consultation: a systematic review of assessment tools and their measurement properties | Multiple | Video |  |  | X |
| Bartolo 2019 | Effectiveness of psycho-educational interventions with telecommunication technologies on emotional distress and quality of life of adult cancer patients: a systematic review | Cancer | Telephone |  |  | X |
| Bashshur 2015 | The empirical evidence for the telemedicine intervention in diabetes management | Metabolic | Both | X | X | X |
| Bashshur 2015 | The Empirical Foundations of Teledermatology: A Review of the Research Evidence | Dermatology | Video | X |  | X |
| Batalik 2020 | Remotely monitored telerehabilitation for cardiac patients: A review of the current situation | Cardiovascular | Both | X | X | X |
| Batalik 2023 | The cost-effectiveness of exercise-based cardiac telerehabilitation intervention: a systematic review | Cardiovascular | Both | X |  |  |
| Batastini 2016 | Telepsychological services with criminal justice and substance abuse clients: A systematic review and meta-analysis | Mental health | Both | X | X | X |
| Batsis 2019 | Effectiveness of Ambulatory Telemedicine Care in Older Adults: A Systematic Review | Multiple | Video | X | X | X |
| Battineni 2021 | The benefits of telemedicine in personalized prevention of cardiovascular diseases (CVD): A systematic review | Cardiovascular | Telephone | X | X | X |
| Bauce 2018 | Videoconferencing for Management of Heart Failure: An Integrative Review | Cardiovascular | Video |  | X | X |
| Bauernschmidt 2023 | Technology-Based Counselling for People with Dementia and Their Informal Carers: A Systematic Review and Meta-Analysis | CNS | Both |  | X | X |
| Bayati 2021 | Comprehensive review of factors influencing the use of telepractice in stuttering treatment | CNS | Both |  |  |  |
| Beatty 2016 | A Systematic Review of Predictors of, and Reasons for, Adherence to Online Psychological Interventions | Mental health | Telephone | X |  |  |
| Bedi 2021 | Telemedicine in International Cleft Care: A Systematic Review | Denistry | Both |  |  |  |
| Beks 2022 | Community Health Programs Delivered Through Information and Communications Technology in High-Income Countries: Scoping Review | Multiple | Both | X | X | X |
| Bekteshi 2023 | Teleintervention for users of augmentative and alternative communication devices: A systematic review | Multiple | Video | X | X | X |
| Beland 2022 | Case Management and Telehealth: A Scoping Review | Multiple | Both | X | X | X |
| Bellanti 2022 | Rapid Review on the Effectiveness of Telehealth Interventions for the Treatment of Behavioral Health Disorders | Mental health | Both | X | X | X |
| Benz 2022 | Use of Teletherapy for allied health interventions in community-based disability services: A scoping review of user perspectives | Multiple | Video |  | X |  |
| Beratarrechea 2015 | The impact of mobile health interventions on chronic disease outcomes in developing countries: A systematic review | Multiple | Telephone | X |  | X |
| Bernal-Utrera 2022 | Therapeutic Exercise Interventions through Telerehabilitation in Patients with Post COVID-19 Symptoms: A Systematic Review | Infectious disease | Both |  |  | X |
| Bernhardsson 2023 | Digital physiotherapy assessment vs conventional face-to-face physiotherapy assessment of patients with musculoskeletal disorders: A systematic review | Musckuloskeletal | Video |  |  | X |
| Berryhill 2019 | Videoconferencing Psychotherapy and Depression: A Systematic Review | Mental health | Video |  |  | X |
| Berryhill 2019 | Videoconferencing psychological therapy and anxiety: a systematic review | Mental health | Video |  |  |  |
| Bervell 2019 | A comparative review of mobile health and electronic health utilization in sub-Saharan African countries | Multiple | Both | X |  | X |
| Betancourt 2020 | The Impact of COVID-19 on Telemedicine Utilization Across Multiple Service Lines in the United States | Multiple | Both | X |  |  |
| Betts 2018 | Telerehabilitation versus standard care for improving cognitive function and quality of life for adults with traumatic brain injury: A systematic review | CNS | Both |  |  | X |
| Beyer 2023 | Practitioner and digitally delivered interventions for reducing hazardous and harmful alcohol consumption in people not seeking alcohol treatment: a systematic review and network metaâ€analysis | Mental health | Telephone |  | X |  |
| Bhattarai 2017 | The role of digital health technologies in management of pain in older people: An integrative review | Pain | Both |  |  |  |
| Bhochhibhoya 2021 | Interventions Using mHealth Strategies to Improve Screening Rates of Cervical Cancer: A Scoping Review | Cancer | Telephone | X | X | X |
| Bhui 2015 | Interventions designed to improve therapeutic communications between black and minority ethnic people and professionals working in psychiatric services: a systematic review of the evidence for their effectiveness | Mental health | Internet-based NOS | X | X | X |
| Biebuyck 2022 | Impact of telehealth interventions added to peritoneal dialysis-care: a systematic review | GU | Both | X | X | X |
| Bingham 2021 | Impact of Telehealth Interventions on Medication Adherence for Patients With Type 2 Diabetes, Hypertension, and/or Dyslipidemia: A Systematic Review | Multiple | Telephone |  | X |  |
| Binng 2021 | Distance Assessment for Detecting Cognitive Impairment in Older Adults: A Systematic Review of Psychometric Evidence | Multiple | Both | X |  |  |
| Blackmore 2022 | The Effectiveness of Virtual Lactation Support: A Systematic Review and Meta-Analysis | Maternal health | Both |  |  | X |
| Blackport 2021 | Online psychosocial intervention for persons with spinal cord injury: A meta-analysis | Mental health | Telephone |  |  | X |
| Blackwood 2021 | Outcomes of Telehealth-Delivered Physical Activity Programs in Adult Cancer Survivors: A Systematic Review | Cancer | Telephone |  | X | X |
| Blount 2023 | Patient reported satisfaction levels with the use of telemedicine for general surgery-A systematic review of randomized control trials | Surgery | Both |  |  | X |
| Bodle 2022 | Comparison of teledermatological examinations with conventional office visits in management of acne vulgaris: A review of current literature | Dermatology | Video | X | X | X |
| Boggan 2020 | Effectiveness of Acute Care Remote Triage Systems: a Systematic Review | Multiple | Telephone |  | X | X |
| Bolton 2015 | Telepsychology for Posttraumatic Stress Disorder: A systematic review | Mental health | Both | X | X | X |
| Bond 2021 | Systematic Review of RCTs Assessing the Effectiveness of mHealth Interventions to Improve Statin Medication Adherence: Using the Behaviour-Change Technique Taxonomy to Identify the Techniques That Improve Adherence | Cardiovascular | Telephone |  | X |  |
| Bonnechere 2021 | Is mhealth a useful tool for self-assessment and rehabilitation of people with multiple sclerosis? A systematic review | CNS | Telephone |  |  | X |
| Bonnevie 2021 | Advanced telehealth technology improves home-based exercise therapy for people with stable chronic obstructive pulmonary disease: a systematic review | Respiratory | Both | X | X | X |
| Booth 2022 | What is the content of virtually delivered pain management programmes for people with persistent musculoskeletal pain? A systematic review | Musckuloskeletal | Both |  |  | X |
| Borges 2021 | Telemedicine applied to plastic surgery: An integrative review | Surgery | Both | X |  | X |
| Borghouts 2021 | Barriers to and facilitators of user engagement with digital mental health interventions: Systematic review | Mental health | Both | X |  | X |
| Borries 2019 | The impact of telemedicine on patient self-management processes and clinical outcomes for patients with Types I or II Diabetes Mellitus in the United States: A scoping review | Metabolic | Both |  | X | X |
| Bowman 2023 | Videoconferencing interventions and COPD patient outcomes: A systematic review | Respiratory | Both | X |  | X |
| Bradford 2016 | Telehealth services in rural and remote Australia: A systematic review of models of care and factors influencing success and sustainability | Multiple | Video |  |  |  |
| Branowicki 2017 | Meta-Analysis of Clinical Trials That Evaluate the Effectiveness of Hospital-Initiated Postdischarge Interventions on Hospital Readmission | Multiple | Telephone |  |  | X |
| Brick 2022 | The influence of telehealth-based cancer rehabilitation interventions on disability: a systematic review | Cancer | Both |  |  | X |
| Brigo 2022 | Using Telehealth to Guarantee the Continuity of Rehabilitation during the COVID-19 Pandemic: A Systematic Review | Rehabilitation | Both |  |  | X |
| Brody 2020 | Chat-based hotlines for health promotion: a systematic review | Not specified | Telephone | X | X | X |
| Brors 2019 | Modes of e-Health delivery in secondary prevention programmes for patients with coronary artery disease: A systematic review | Cardiovascular | Telephone |  | X | X |
| Brown 2021 | A systematic review of economic evaluations of web-based or telephone-delivered interventions for preventing overweight and obesity and/or improving obesity-related behaviors | Metabolic | Telephone | X |  |  |
| Brown 2021 | Videoconferencing to deliver genetics services: a systematic review of telegenetics in light of the COVID-19 pandemic | Multiple | Both | X | X | X |
| Brown 2022 | Effectiveness of exercise via telehealth for chronic disease: a systematic review and meta-analysis of exercise interventions delivered via videoconferencing | Multiple | Both | X |  | X |
| Bu 2022 | Optimising implementation of telehealth in oncology: A systematic review examining barriers and enablers using the RE-AIM planning and evaluation framework | Cancer | Both | X |  | X |
| Bucki 2021 | Scoping Review of Telehealth for Musculoskeletal Disorders: Applications for the COVID-19 Pandemic | Musckuloskeletal | Telephone |  |  | X |
| Budd 2022 | Empathy in patient-clinician interactions when using telecommunication: A rapid review of the evidence | Multiple | Telephone |  |  | X |
| Buneviciene 2021 | Can mHealth interventions improve quality of life of cancer patients? A systematic review and meta-analysis | Cancer | Video |  |  | X |
| Buonanno 2023 | Telemedicine in Cancer Pain Management: A Systematic Review and Meta-Analysis of Randomized Controlled Trials | Cancer | Telephone | X |  | X |
| Burke 2023 | Videoconferencing of Movement-Based and Psychologically Informed Interventions for Chronic Pain: A Systematic Review and Horizon Scan | Pain | Both |  |  | X |
| Burton 2022 | Telerehabilitation physical exercise for patients with lung cancer through the course of their disease: A systematic review | Cancer | Both | X | X | X |
| Bush 2016 | The Role of Telemedicine in Auditory Rehabilitation: A Systematic Review | Rehabilitation | Both |  |  | X |
| Butz 2022 | User experience reevaluation and diffusion of technology in the context of compulsory usage illustrated by the example of telepsychotherapy-a literature review | Mental health | Both |  |  | X |
| Byaruhanga 2020 | Effectiveness of individual real-time video counseling on smoking, nutrition, alcohol, physical activity, and obesity health risks: Systematic review | Multiple | Video |  |  | X |
| Cabrera 2021 | Systematic Review of Telehealth Cost Minimization for Patients and Health Systems in Otolaryngology | ENT | Video | X |  |  |
| Caffery 2017 | Outcomes of using telehealth for the provision of healthcare to Aboriginal and Torres Strait Islander people: a systematic review | Multiple | Video | X | X | X |
| Calleja 2023 | Offsite primary care providers using telehealth to support a sustainable workforce in rural and remote general practice: A rapid review of the literature | Multiple | Both | X |  | X |
| Camacho 2019 | Smartphone apps to support coordinated specialty care for prodromal and early course schizophrenia disorders: Systematic review | Mental health | Mobile App | X |  | X |
| Cameron 2021 | Systematic Review of Telehospice Telemedicine and e-Health | End of life | Both | X | X | X |
| Caminiti 2023 | Psychosocial Impact of Virtual Cancer Care through Technology: A Systematic Review and Meta-Analysis of Randomized Controlled Trials | Cancer | Both |  |  | X |
| Campanati 2022 | Treatment of Moderate to Severe Psoriasis during the COVID-19 Pandemic: Lessons Learned and Opportunities | Autoimmune | Telemedicine NOS |  |  |  |
| Cantor 2022 | Telehealth Strategies for the Delivery of Maternal Health Care : A Rapid Review | Maternal health | Both |  | X | X |
| Cantor 2023 | Telehealth for Women's Preventive Services for Reproductive Health and Intimate Partner Violence: a Comparative Effectiveness Review | Assault | Both | X |  | X |
| Cao 2022 | mHealth Interventions for Self-management of Hypertension: Framework and Systematic Review on Engagement, Interactivity, and Tailoring | Cardiovascular | Both | X |  | X |
| Cao 2022 | Effectiveness of telepharmacy diabetes services: A systematic review and meta-analysis | Metabolic | Both |  |  | X |
| Caputo 2022 | Telehealth Interventions in Head and Neck Cancer Patients: A Systematic Review | Cancer | Telephone | X | X | X |
| Cardona 2023 | Clinical and cost-effectiveness of telehealth for Indigenous and culturally and linguistically diverse (CALD) people: a scoping review | Multiple | Both | X | X | X |
| Carotenuto 2021 | Tele-Neuropsychological Assessment of Alzheimer's Disease | CNS | Both | X |  |  |
| CarrillodeAlbornoz 2022 | The effectiveness of teleconsultations in primary care: systematic review | Multiple | Both | X | X | X |
| Carrington 2022 | The Use of Telehealth to Perform Occupational Therapy Home Assessments: An Integrative Literature Review | Occupational therapy | Video | X | X | X |
| Cartujano-Barrera 2022 | Smoking Cessation Mobile Interventions in Latin America: A Systematic Review | Mental health | Both |  | X |  |
| Cartwright 2021 | eHealth interventions to support patients in delivering and managing peritoneal dialysis at home: A systematic review | GU | Both | X | X | X |
| Castro 2020 | Effectiveness and adherence of telephone-administered psychotherapy for depression: A systematic review and meta-analysis | Mental health | Telephone | X |  | X |
| Catapan 2021 | Same goals, different challenges: A systematic review of perspectives of people with diabetes and healthcare professionals on Type 2 diabetes care | Metabolic | Telemedicine NOS |  |  | X |
| Cavalheiro 2021 | Effectiveness of Tele-rehabilitation Programs in Heart Failure: A Systematic Review and Meta-analysis | Cardiovascular | Telephone | X | X | X |
| Cavero-Redondo 2021 | Comparative effect of eHealth interventions on hypertension management-related outcomes: A network meta-analysis | Cardiovascular | Telephone |  | X | X |
| Cen 2022 | Systematic literature review of adopting eHealth in pharmaceutical care during COVID-19 pandemic: recommendations for strengthening pharmacy services | Multiple | Video |  | X | X |
| Chae 2021 | Internet-based prenatal interventions for maternal health among pregnant women: A systematic review and meta-analysis | Maternal health | Telephone |  | X | X |
| Chan 2016 | Exercise Telemonitoring and Telerehabilitation Compared with Traditional Cardiac and Pulmonary Rehabilitation: A Systematic Review and Meta-Analysis | Cardiovascular | Video | X | X | X |
| Chan 2021 | The effectiveness of e-interventions on fall, neuromuscular functions and quality of life in community-dwelling older adults: A systematic review and meta-analysis | Not specified | Telephone |  |  | X |
| Chan 2022 | Effectiveness of eHealthâ€based cognitive behavioural therapy on depression: A systematic review and metaâ€analysis | Mental health | Telephone | X |  | X |
| Chan 2022 | Opioid treatment programs, telemedicine and COVID-19: A scoping review | Pain | Both |  | X | X |
| Chandeying 2021 | Online Interventions to Improve Mental Health of Pediatric, Adolescent, and Young Adult Cancer Survivors: A Systematic Review and Meta-Analysis | Mental health | Both |  |  | X |
| Chandler 2022 | Developing Culturally Tailored mHealth Tools to Address Sexual and Reproductive Health Outcomes Among Black and Latina Women: A Systematic Review | Maternal health | Telephone |  | X |  |
| Charova 2015 | Web-based interventions for comorbid depression and chronic illness: a systematic review | Multiple | Both |  |  | X |
| Charron 2022 | Virtual versus Face-to-Face Cognitive Behavioral Treatment of Depression: Meta-Analytic Test of a Noninferiority Hypothesis and Men's Mental Health Inequities | Mental health | Both |  | X |  |
| Chaudhry 2021 | How Satisfied Are Patients and Surgeons with Telemedicine in Orthopaedic Care During the COVID-19 Pandemic? A Systematic Review and Meta-analysis | Musckuloskeletal | Both |  | X | X |
| Chen 2015 | Telerehabilitation Approaches for Stroke Patients: Systematic Review and Meta-analysis of Randomized Controlled Trials | Cardiovascular | Both |  |  | X |
| Chen 2018 | Effect of telehealth intervention on breast cancer patients' quality of life and psychological outcomes: A meta-analysis | Cancer | Both |  | X | X |
| Chen 2019 | Home-based technologies for stroke rehabilitation: A systematic review | Cardiovascular | Both |  |  |  |
| Chen 2020 | Application of telehealth intervention in Parkinson's disease: A systematic review and meta-analysis | Parkinsons | Video |  |  | X |
| Chen 2020 | Effect of electronic health interventions on metabolic syndrome: A systematic review and meta-analysis | Metabolic | Telephone |  |  | X |
| Chen 2020 | Telemedicine in Chronic Wound Management: Systematic Review And Meta-Analysis | Wound | Both | X | X | X |
| Chen 2021 | Efficacy of Mobile Health in Patients With Low Back Pain: Systematic Review and Meta-analysis of Randomized Controlled Trials | Pain | Telephone |  |  | X |
| Chen 2022 | Evidence of Phone vs Video-Conferencing for Mental Health Treatments: A Review of the Literature | Mental health | Both | X | X | X |
| Chen 2022 | Effectiveness of Digital Health Interventions on Unintentional Injury, Violence, and Suicide: Meta-Analysis | Mental health | Video |  | X | X |
| Chen 2022 | Telemedicine for Preventing and Treating Pressure Injury after Spinal Cord Injury: Systematic Review and Meta-analysis | Musckuloskeletal | Both | X |  | X |
| Chen 2022 | The application of eHealth in cancer survivorship care: A review of web-based dyadic interventions for post-treatment cancer survivors and caregivers | Cancer | Both | X |  | X |
| Chen 2022 | The Effect of Tele-palliative Care on Patient and Caregiver Outcomes: A Systematic Review | End of life | Both | X |  | X |
| Chen 2023 | The efficacy of telemedicine for pain management in patients with cancer: a systematic review and meta-analysis | Pain | Both |  |  | X |
| Cheng 2020 | Technology-Delivered Psychotherapeutic Interventions in Improving Depressive Symptoms Among People with HIV/AIDS: A Systematic Review and Meta-analysis of Randomised Controlled Trials | Mental health | Telephone |  |  | X |
| Chi 2015 | A systematic review of telehealth tools and interventions to support family caregivers | Multiple | Both |  |  | X |
| Chi 2022 | The effectiveness and associated factors of online psychotherapy on COVID-19 related distress: A systematic review and meta-analysis | Infectious disease | Both |  |  | X |
| Chib 2018 | Theoretical Advancements in mHealth: A Systematic Review of Mobile Apps | Multiple | Both | X |  | X |
| Chilala 2023 | Evaluating the Effectiveness of Remote Behavioral Interventions Facilitated by Health Care Providers at Improving Medication Adherence in Cardiometabolic Conditions: A Systematic Review and Meta-Analysis | Metabolic | Both |  | X | X |
| Cho 2023 | The effectiveness of non-pharmacological interventions using information and communication technologies for behavioral and psychological symptoms of dementia: A systematic review and meta-analysis | CNS | Both |  |  | X |
| Choi 2018 | mHealth Approaches in Managing Skin Cancer: Systematic Review of Evidence-Based Research Using Integrative Mapping | Cancer | Multiple | X |  | X |
| Choi 2023 | Effectiveness of Remote Interventions to Improve Medication Adherence in Patients after Stroke: A Systematic Literature Review and Meta-Analysis | Cardiovascular | Both | X | X | X |
| Chong 2021 | Teleophthalmology and its evolving role in a COVID-19 pandemic: A scoping review | Infectious disease | Both |  | X | X |
| Chongmelaxme 2019 | The Effects of Telemedicine on Asthma Control and Patients' Quality of Life in Adults: A Systematic Review and Meta-analysis | Respiratory | Both |  |  | X |
| Choukou 2021 | Digital health technology for Indigenous older adults: A scoping review | Multiple | Both |  | X | X |
| Choukou 2021 | Digital health technology for remote care in response to the COVID-19 pandemic: a scoping review | All health conditions | Both | X | X | X |
| Christensen 2020 | Patients' and providers' experiences with video consultations used in the treatment of older patients with unipolar depression: A systematic review | Mental health | Video | X | X | X |
| Christopoulou 2018 | Evidence-based health and clinical informatics: a systematic review on randomized controlled trials | Multiple | Multiple | X | X | X |
| Chua 2022 | The Willingness to Pay for Telemedicine Among Patients With Chronic Diseases: Systematic Review | Multiple | Both | X |  |  |
| Chuchu 2018 | Teledermatology for diagnosing skin cancer in adults | Cancer | Video | X |  |  |
| Chung-Lee 2023 | A new approach to digital health? Virtual COVID-19 care: A scoping review | Infectious disease | Both |  |  | X |
| Claborn 2015 | Computer-based HIV adherence promotion interventions: a systematic review: Translation Behavioral Medicine | Infectious disease | Video |  | X |  |
| Clark 2015 | Alternative models of cardiac rehabilitation: a systematic review | Cardiovascular | Both | X |  | X |
| Clark 2018 | Systematic review of mobile phone-based teledermatology | Multiple | Telephone | X |  |  |
| Clark 2022 | Are remote mental healthcare interventions cost-effective? A systematic review of economic evaluations of remote mental healthcare | Mental health | Both | X |  |  |
| Coleman 2015 | Assessment and Treatment of Cognition and Communication Skills in Adults With Acquired Brain Injury via Telepractice: A Systematic Review | CNS | Both | X |  | X |
| Colombo 2022 | Low-Risk Antenatal Care Enhanced by Telemedicine: A Practical Guideline Model | Maternal health | Video | X |  | X |
| Comendador 2023 | Telephone-Delivered Interventions for Suicide Prevention in Schizophrenia and Related Disorders: A Systematic Review | Mental health | Telephone |  | X | X |
| Connolly 2020 | A systematic review of providers' attitudes toward telemental health via videoconferencing | Mental health | Video |  |  | X |
| Cordeiro 2022 | Quality of Life in Patients With Heart Failure Assisted By Telerehabilitation: A Systematic Review and Meta-Analysis | Cardiovascular | Video |  |  | X |
| Correia 2021 | Telemedicine to deliver diabetes care in low-and middle-income countries: A systematic review and meta-analysis | Metabolic | Both |  | X | X |
| Corry 2019 | Telephone interventions, delivered by healthcare professionals, for providing education and psychosocial support for informal caregivers of adults with diagnosed illnesses | Multiple | Telephone |  | X | X |
| Corso 2022 | Are Nonpharmacologic Interventions Delivered Through Synchronous Telehealth as Effective and Safe as In-Person Interventions for the Management of Patients With Nonacute Musculoskeletal Conditions? A Systematic Rapid Review | Musckuloskeletal | Both |  |  | X |
| Cotelli 2019 | Cognitive telerehabilitation in mild cognitive impairment, Alzheimer's disease and frontotemporal dementia: A systematic review | CNS | Video | X |  | X |
| Cottrell 2017 | Real-time telerehabilitation for the treatment of musculoskeletal conditions is effective and comparable to standard practice: a systematic review and meta-analysis | Musckuloskeletal | Both |  | X | X |
| Coughtrey 2018 | The effectiveness of telephone-delivered psychological therapies for depression and anxiety: A systematic review | Mental health | Telephone |  |  | X |
| Coulibaly 2022 | Attributes Underlying Patient Choice for Telerehabilitation Treatment: A Mixed-Methods Systematic Review to Support a Discrete Choice Experiment Study Design | Multiple | Telemedicine NOS |  |  |  |
| Coumoundouros 2022 | Implementation of e-Mental Health Interventions for Informal Caregivers of Adults With Chronic Diseases: Mixed Methods Systematic Review With a Qualitative Comparative Analysis and Thematic Synthesis | Multiple | Both |  |  |  |
| Coustasse 2019 | Use of Teledermatology to Improve Dermatological Access in Rural Areas | Dermatology | Both | X | X | X |
| Cowan 2019 | Barriers to Use of Telepsychiatry: Clinicians as Gatekeepers | Mental health | Video |  |  | X |
| Cox 2017 | Cancer survivors' experience with telehealth: A systematic review and thematic synthesis | Cancer | Both |  |  | X |
| Cox 2021 | Telerehabilitation for chronic respiratory disease | Respiratory | Both |  |  | X |
| Coxe 2020 | Telebehavioral Interventions for Family Caregivers of Individuals With Traumatic Brain Injury: A Systematic Review | CNS | Both |  | X | X |
| Cruz-Cobo 2022 | Effectiveness of mHealth Interventions in the Control of Lifestyle and Cardiovascular Risk Factors in Patients After a Coronary Event: Systematic Review and Meta-analysis | Cardiovascular | Both |  | X | X |
| Cuenca-Martinez 2022 | Implementation of Online Behavior Modification Techniques in the Management of Chronic Musculoskeletal Pain: A Systematic Review and Meta-Analysis | Musckuloskeletal | Both | X | X | X |
| Currie 2022 | Effectiveness of Live Health Professional-Led Group eHealth Interventions for Adult Mental Health: Systematic Review of Randomized Controlled Trials | Mental health | Both |  | X | X |
| Curry 2021 | Feasibility, acceptability, and efficacy of online supportive care for individuals living with and beyond lung cancer: a systematic review | Cancer | Video | X | X | X |
| DÃ¼lsen 2020 | Digital interventions in adult mental healthcare settings: recent evidence and future directions | Mental health | Both |  |  |  |
| daCosta 2020 | How Has Teledentistry Been Applied in Public Dental Health Services? An Integrative Review | Denistry | Video | X |  |  |
| Daifi 2016 | Evolving Frontier: A Review of the Role of Mobile Medical Application Prescribing | All health conditions | Mobile App | X | X | X |
| Dalley 2021 | Health Care Professionals' and Patients' Management of the Interactional Practices in Telemedicine Videoconferencing: A Conversation Analytic and Discursive Systematic Review | All health conditions | Both |  | X |  |
| daMata 2021 | Telehealth in the rehabilitation of female pelvic floor dysfunction: a systematic literature review | Multiple | Video | X |  | X |
| D'Amore 2022 | Interventions Including Smart Technology Compared With Face-to-face Physical Activity Interventions in Older Adults: Systematic Review and Meta-analysis | Not specified | Both |  |  | X |
| Danylchuk 2021 | Telehealth for genetic counseling: A systematic evidence review | Multiple | Both | X | X | X |
| Dao 2021 | Smartphone-Delivered Ecological Momentary Interventions Based on Ecological Momentary Assessments to Promote Health Behaviors: Systematic Review and Adapted Checklist for Reporting Ecological Momentary Assessment and Intervention Studies | Multiple | Telephone |  | X | X |
| daSilva 2021 | The role of teledentistry in oral cancer patients during the COVID-19 pandemic: an integrative literature review | Cancer | Both |  |  |  |
| daSilvaNegreiros 2021 | Digital technologies in the care of people with diabetes during the COVID-19 pandemic: a scoping review | Metabolic | Both | X | X | X |
| Davey 2020 | Virtual Fracture Clinics in Orthopaedic Surgery - A Systematic Review of Current Evidence | Surgery | Video | X | X | X |
| David 2016 | Innovative health informatics as an effective modern strategy in diabetes management: a critical review | Metabolic | Both |  | X | X |
| Davis 2020 | A systematic review of tailored mHealth interventions for physical activity promotion among adults | Multiple | Telephone |  | X |  |
| Davis 2021 | Telehealth and mobile health interventions in adults with inflammatory bowel disease: A mixed-methods systematic review | GI | Both | X | X | X |
| Dawson 2020 | Telehealth and indigenous populations around the world: a systematic review on current modalities for physical and mental health | Multiple | Both |  |  | X |
| DeGuzman 2021 | Economic evaluations of videoconference and telephone consultations in primary care: A systematic review | Multiple | Both | X |  |  |
| delaTorre-Diez 2015 | Cost-utility and cost-effectiveness studies of telemedicine, electronic, and mobile health systems in the literature: a systematic review | Not specified | Both | X |  |  |
| delaTorreDiez 2016 | Monitoring and Follow-up of Chronic Heart Failure: a Literature Review of eHealth Applications and Systems | Cardiovascular | Telephone |  |  |  |
| delaTorreDiez 2018 | Systematic Review about QoS and QoE in Telemedicine and eHealth Services and Applications | All health conditions | Both |  |  | X |
| Deldar 2016 | Teleconsultation and Clinical Decision Making: a Systematic Review | Multiple | Both |  |  | X |
| deLeo 2022 | Cancer Patients and Telenursing Interventions in Italy: A Systematic Review | Cancer | Telephone |  | X | X |
| Delgoshaei 2017 | Telemedicine: A systematic review of economic evaluations | All health conditions | Both | X | X | X |
| DelHoyo 2023 | Are we ready for telemonitoring inflammatory bowel disease? A review of advances, enablers, and barriers | GI | Both |  |  |  |
| DelPino 2022 | Costs and effects of telerehabilitation in neurological and cardiological diseases: A systematic review | Multiple | Telemedicine NOS | X |  | X |
| Demoen 2023 | Effectiveness of Telerehabilitation Interventions for Self-management of Tinnitus: Systematic Review | ENT | Telephone |  |  | X |
| Deng 2018 | Effects of telephone support on exercise capacity and quality of life in patients with chronic obstructive pulmonary disease: a meta-analysis | Respiratory | Telephone |  | X | X |
| Deng 2022 | The effect of telemedicine on secondary prevention of atherosclerotic cardiovascular disease: A systematic review and meta-analysis | Cardiovascular | Both |  | X | X |
| Deng 2023 | eHealth-Based Psychosocial Interventions for Adults With Insomnia: Systematic Review and Meta-analysis of Randomized Controlled Trials | CNS | Telephone |  |  | X |
| DeNicola 2020 | Telehealth Interventions to Improve Obstetric and Gynecologic Health Outcomes: A Systematic Review | Maternal health | Both | X | X | X |
| Dening 2020 | Web-based interventions for dietary behavior in adults with Type 2 diabetes: Systematic review of randomized controlled trials | Metabolic | Internet-based NOS |  | X | X |
| Dequanter 2021 | The Effectiveness of e-Health Solutions for Aging With Cognitive Impairment: A Systematic Review | Geriatric | Both |  |  | X |
| DeRavin 2023 | Feasibility and Cost of Telehealth Head and Neck Cancer Survivorship Care: A Systematic Review | Cancer | Both | X |  |  |
| DeSimone 2022 | Implementations and strategies of telehealth during COVID-19 outbreak: a systematic review | Multiple | Both |  |  | X |
| Devani 2022 | Digital health RCT interventions for cardiovascular disease risk reduction: a systematic review and meta-analysis | Cardiovascular | Telephone |  |  | X |
| DeVera 2022 | Virtual Primary Care Implementation During COVID-19 in High-Income Countries: A Scoping Review | Multiple | Both |  |  |  |
| DeZeeuw 2018 | Impact of telephonic comprehensive medication reviews on patient outcomes | Multiple | Telephone |  |  | X |
| Dhippayom 2020 | Comparative effects of telemedicine and face-to-face warfarin management: A systematic review and network meta-analysis | Multiple | Both |  |  | X |
| Diano 2023 | A Systematic Review of Mobile Apps as an Adjunct to Psychological Interventions for Emotion Dysregulation | Mental health | Mobile App |  | X | X |
| Diaz 2022 | Providing Contraceptive Health Services to Adolescents and Young Adults by Telemedicine: A Scoping Review of Patient and Provider Perspectives | Multiple | Both | X |  | X |
| Diedrich 2021 | Video-based teleconsultations in pharmaceutical care - A systematic review | Multiple | Video |  | X | X |
| DiFabio 2022 | A Scoping Review of Technology and Infrastructure Needs in the Delivery of Virtual Hearing Aid Services | ENT | Both |  |  |  |
| Dionisi 2021 | The Use of mHealth in Orthopedic Surgery: A Scoping Review | Surgery | Telephone |  |  | X |
| DiTella 2020 | Integrated telerehabilitation approach in multiple sclerosis: A systematic review and meta-analysis | CNS | Both |  | X | X |
| Dol 2020 | Impact of mobile health interventions during the perinatal period on maternal psychosocial outcomes: a systematic review | Maternal health | Both |  | X | X |
| Dolan 2021 | Virtual care in end of life and palliative care: A rapid evidence check | End of life | Both | X |  | X |
| Domhardt 2020 | Are Internet- and mobile-based interventions effective in adults with diagnosed panic disorder and/or agoraphobia? A systematic review and meta-analysis | Mental health | Both | X |  | X |
| Donker 2015 | Economic evaluations of Internet interventions for mental health: a systematic review | Mental health | Telephone | X |  |  |
| Donnell 2022 | Digital Interventions to Save Lives From the Opioid Crisis Prior and During the SARS COVID-19 Pandemic: A Scoping Review of Australian and Canadian Experiences | Pain | Telephone |  | X | X |
| Doraiswamy 2020 | Use of telehealth during the COVID-19 pandemic: Scoping review | All health conditions | Both |  |  |  |
| Doraiswamy 2021 | Telehealth Use in Geriatrics Care during the COVID-19 Pandemic-A Scoping Review and Evidence Synthesis | Not specified | Both |  |  |  |
| Dosani 2020 | mHealth and Perinatal Depression in Low-and Middle-Income Countries: A Scoping Review of the Literature | Maternal health | Both |  |  | X |
| Douglass 2023 | Clinician Perspectives of Telehealth Pre-COVID-19: A Systematic Review and Qualitative Metasynthesis | Rehabilitation | Video |  |  | X |
| Doumen 2022 | Engagement and attrition with eHealth tools for remote monitoring in chronic arthritis: a systematic review and meta-analysis | Musckuloskeletal | Telephone | X |  |  |
| Dovigi 2020 | A Framework-Driven Systematic Review of the Barriers and Facilitators to Teledermatology Implementation | Dermatology | Both | X |  |  |
| Downes 2017 | Telephone consultations for general practice: A systematic review | Multiple | Telephone |  | X | X |
| Drissi 2021 | A Systematic Literature Review on e-Mental Health Solutions to Assist Health Care Workers During COVID-19 | Mental health | Telephone |  |  |  |
| Driver 2022 | The impact of the SARS-CoV-2 pandemic on the management of chronic limb-threatening ischemia and wound care | Not specified | Both |  |  | X |
| Duettmann 2021 | eHealth in transplantation | Surgery | Video |  |  |  |
| Duff 2017 | Behavior Change Techniques in Physical Activity eHealth Interventions for People With Cardiovascular Disease: Systematic Review | Cardiovascular | Both |  | X |  |
| Duke 2018 | Distal technologies and type 1 diabetes management | Metabolic | Both | X |  | X |
| Durojaiye 2022 | Effectiveness of telemedicine in outpatient parenteral antimicrobial therapy (Tele-OPAT): A systematic review | Multiple | Both | X | X | X |
| Eastwood 2015 | Secondary triage in prehospital emergency ambulance services: a systematic review | Acute/ urgent care | Telephone |  | X | X |
| Eberle 2021 | Clinical improvements by telemedicine interventions managing type 1 and type 2 diabetes: Systematic meta-review | Metabolic | Both | X | X | X |
| Eberle 2021 | Telemetric Interventions Offer New Opportunities for Managing Type 1 Diabetes Mellitus: Systematic Meta-review | Metabolic | Both | X | X | X |
| Eberle 2021 | Effects of Telemetric Interventions on Maternal and Fetal or Neonatal Outcomes in Gestational Diabetes: Systematic Meta-Review | Maternal health | Both |  | X | X |
| Echelard 2021 | Use of Telemedicine in Depression Care by Physicians: Scoping Review | Mental health | Both | X |  | X |
| Edge 2023 | The efficacy of self-guided internet and mobile-based interventions for preventing anxiety and depression - A systematic review and meta-analysis | Mental health | Both |  |  | X |
| Edwards 2022 | Pre COVID-19 Pandemic Use of Telemedicine for Adherence Promotion in Patients with Psychotic Disorders: A Literature Review | Mental health | Both |  | X |  |
| Edwards 2022 | A rapid review of the effectiveness of remote consultations versus face-to-face consultations in secondary care surgical outpatient settings | Surgery | Both | X |  | X |
| Edwards 2022 | Mobile health as a primary mode of intervention for women at risk of or diagnosed with gestational diabetes mellitus: a scoping review | Maternal health | Video |  | X | X |
| Elbadawi 2022 | Digital health intervention in patients with recent hospitalization for acute heart failure: A systematic review and meta-analysis of randomized trials | Cardiovascular | Telephone |  |  | X |
| Eldaly 2022 | Patient satisfaction with telemedicine in acute care setting: A systematic review | Acute/ urgent care | Both |  |  | X |
| ElKefi 2021 | How technology impacts communication between cancer patients and their health care providers: A systematic literature review | Cancer | Both | X | X |  |
| Ellis 2020 | Implementation of e-mental health for depression and anxiety: A critical scoping review | Mental health | Both | X |  |  |
| Ellis 2021 | The Application of e-Mental Health in Response to COVID-19: Scoping Review and Bibliometric Analysis | Mental health | Both |  |  |  |
| ElMorr 2020 | Effectiveness of ICT-based intimate partner violence interventions: A systematic review | Assault | Both | X |  | X |
| Elsner 2020 | Teledermatology in the times of COVID-19 - a systematic review | Dermatology | Both | X |  | X |
| Emonena 2022 | The Efficacy of Tele-Monitoring in Maintaining Glycated Haemoglobin Levels in Patients with Type 2 Diabetes Mellitus: A Systematic Review | Metabolic | Both | X |  |  |
| EmtekaerHaesum 2016 | Influence of health literacy on outcomes using telehomecare technology: A systematic review | Multiple | Telephone |  |  | X |
| Endler 2019 | Telemedicine for medical abortion: a systematic review | Maternal health | Both | X | X | X |
| Engelsma 2021 | Considerate mHealth design for older adults with Alzheimer's disease and related dementias (ADRD): A scoping review on usability barriers and design suggestions | CNS | Both |  |  |  |
| Erridge 2019 | Telementoring of Surgeons: A Systematic Review | Surgery | Both | X | X | X |
| EscrivaBoulley 2018 | Digital health interventions to help living with cancer: A systematic review of participants' engagement and psychosocial effects | Cancer | Video | X |  | X |
| Esfandiari 2021 | The Effect of Telehealth Interventions on Function and Quality of Life for Older Adults with Pre-Frailty or Frailty: A Systematic Review and Meta-Analysis | Geriatric | Both |  | X | X |
| Esfandiari 2022 | Telehealth interventions for mobility after lower limb loss: A systematic review and meta-analysis of randomized controlled trials | Geriatric | Both |  | X | X |
| EslamiJahromi 2023 | Impact of telecare interventions on quality of life in older adults: a systematic review | Geriatric | Video |  |  | X |
| EslamiJahromi 2023 | Utilization of telehealth to manage the Covid-19 pandemic in low- and middle-income countries: a scoping review | Multiple | Both |  |  | X |
| Etzelmueller 2020 | Effects of Internet-Based Cognitive Behavioral Therapy in Routine Care for Adults in Treatment for Depression and Anxiety: Systematic Review and Meta-Analysis | Mental health | Both | X |  | X |
| Eustache 2021 | Do postoperative telemedicine interventions with a communication feature reduce emergency department visits and readmissions?-a systematic review and meta-analysis | Surgery | Both |  |  | X |
| Evans 2022 | The Potential of Telecommunication Technology to Address Racial/Ethnic Disparities in HIV PrEP Awareness, Uptake, Adherence, and Persistence in Care: A Review | Infectious disease | Both |  | X |  |
| Evans 2022 | Remotely Delivered Interventions to Support Women With Symptoms of Anxiety in Pregnancy: Mixed Methods Systematic Review and Meta-analysis | Maternal health | Both | X |  | X |
| Ewart 2022 | Patient perspectives and experiences of remote consultations in people receiving kidney care: A scoping review | GU | Both |  |  | X |
| Fahey 2022 | Telemedicine in Orthopedic Surgery: A Systematic Review of Current Evidence | Surgery | Both | X |  | X |
| Falconer 2018 | Use of technology for care coordination initiatives for patients with mental health issues: A systematic literature review | Mental health | Both |  |  |  |
| Fan 2022 | Mobile health technology: a novel tool in chronic disease management | Multiple | Both | X | X | X |
| Fantinelli 2019 | Assessment of Psychological Dimensions in Telemedicine Care for Gestational Diabetes Mellitus: A Systematic Review of Qualitative and Quantitative Studies | Maternal health | Telephone | X | X | X |
| Farabi 2020 | Economic evaluation of the utilization of telemedicine for patients with cardiovascular disease: a systematic review | Cardiovascular | Both | X |  |  |
| Farrell 2022 | A systematic review of the literature on telepsychiatry for bipolar disorder | Mental health | Both |  | X | X |
| Faruque 2017 | Effect of telemedicine on glycated hemoglobin in diabetes: a systematic review and meta-analysis of randomized trials | Metabolic | Both |  |  | X |
| Farzandipour 2023 | The effectiveness of tele-triage during the COVID-19 pandemic: A systematic review and narrative synthesis | Multiple | Telephone | X | X | X |
| Fatehi 2020 | Teleophthalmology for the elderly population: A review of the literature | CNS | Video |  |  | X |
| Fatrin 2022 | Telemedicine to Support Heart Failure Patients during Social Distancing: A Systematic Review | Cardiovascular | Both |  |  | X |
| Feldhacker 2022 | Telehealth Interventions Within the Scope of Occupational Therapy Practice: A Systematic Review | CNS | Both |  |  | X |
| Fernandes 2022 | At my own pace, space, and place: a systematic review of qualitative studies of enablers and barriers to telehealth interventions for people with chronic pain | Pain | Both |  |  | X |
| FernandesBezerra 2021 | Telemedicine Application and Assessment During the COVID-19 Pandemic | Multiple | Video | X |  | X |
| Fernandez 2021 | Teledentistry and mHealth for Promotion and Prevention of Oral Health: A Systematic Review and Meta-analysis | Denistry | Both |  | X | X |
| Ferorelli 2022 | Digital Health Care, Telemedicine, and Medicolegal Issues in Orthopedics: A Review | Musckuloskeletal | Video |  | X | X |
| Feroz 2017 | Role of mHealth applications for improving antenatal and postnatal care in low and middle income countries: a systematic review | Maternal health | Telephone | X | X |  |
| FerrazDosSantos 2020 | Telehealth and Breastfeeding: An Integrative Review | Maternal health | Both |  | X | X |
| Ferreri 2019 | How New Technologies Can Improve Prediction, Assessment, and Intervention in Obsessive-Compulsive Disorder (e-OCD): Review | Mental health | Both |  |  |  |
| Field 2018 | Cardiac rehabilitation services for people in rural and remote areas: an integrative literature review | Cardiovascular | Telephone |  |  |  |
| Fien 2022 | Feasibility, satisfaction, acceptability and safety of telehealth for First Nations and culturally and linguistically diverse people: a scoping review | All health conditions | Both | X | X | X |
| Finnane 2017 | Teledermatology for the Diagnosis and Management of Skin Cancer: A Systematic Review | Dermatology | Video | X |  |  |
| Fithriyyah 2022 | Using mobile phone applications in engaging nurses for preventing healthcare-associated infections: A systematic review | Infectious disease | Both |  |  | X |
| Fjellsa 2022 | eHealth in Care Coordination for Older Adults Living at Home: Scoping Review | Geriatric | Both |  | X | X |
| Flodgren 2016 | Interactive telemedicine: effects on professional practice and health care outcomes | Not specified | Both | X |  | X |
| Flores 2020 | Teledentistry in the diagnosis of oral lesions: A systematic review of the literature | Cancer | Video | X |  |  |
| Foong 2020 | Facilitators and barriers of using digital technology for the management of diabetic foot ulcers: A qualitative systematic review | Metabolic | Both |  |  | X |
| Ford 2023 | Comparisons of Communication in Medical Face-To-Face and Teleconsultations: A Systematic Review and Narrative Synthesis | All health conditions | Both |  | X |  |
| Fraser 2017 | Use of telehealth for health care of Indigenous peoples with chronic conditions: A systematic review | Multiple | Video | X | X | X |
| Frias 2020 | Technology-Based Psychosocial Interventions for People with Borderline Personality Disorder: A Scoping Review of the Literature | Mental health | Video |  |  | X |
| Fridriksdottir 2018 | Effects of web-based interventions on cancer patients' symptoms: review of randomized trials | Cancer | Both |  |  | X |
| Ftouni 2022 | Challenges of Telemedicine during the COVID-19 pandemic: a systematic review | Infectious disease | Both |  |  |  |
| Fuertes-Guiro 2017 | Opportunity cost of the dermatologist's consulting time in the economic evaluation of teledermatology | Dermatology | Video | X | X |  |
| Fung 2022 | Effectiveness of eHealth mindfulness-based interventions on cancer-related symptoms among cancer patients and survivors: A systematic review and meta-analysis | Cancer | Telephone | X |  | X |
| Furness 2020 | Impact of the method of delivering electronic health behavior change interventions in survivors of cancer on engagement, health behaviors, and health outcomes: Systematic review and meta-analysis | Cancer | Both | X | X | X |
| Gaigher 2022 | Dementia and Mental Health During the COVID-19 Pandemic: A Systematic Review | Mental health | Both |  |  | X |
| Galvin 2022 | Patient and provider perspectives of the implementation of remote consultations for community-dwelling people with mental health conditions: A systematic mixed studies review | Mental health | Both |  |  |  |
| Gan 2022 | Technology-supported strategies for promoting user engagement with digital mental health interventions: A systematic review | Mental health | Both | X |  |  |
| Ganguli 2016 | The impact of patient support programs on adherence, clinical, humanistic, and economic patient outcomes: A targeted systematic review | All health conditions | Telephone | X | X | X |
| Ganjali 2022 | Clinical informatics solutions in COVID-19 pandemic: Scoping literature review | All health conditions | Both |  | X | X |
| Ganjali 2022 | Telemedicine solutions for clinical care delivery during COVID-19 pandemic: A scoping review | Multiple | Both | X | X | X |
| Gao 2023 | Implementation and impacts of virtual team-based care planning for older persons in formal care settings: A scoping review | Multiple | Both |  |  |  |
| Gao 2023 | Effectiveness of home-based cardiac telerehabilitation in patients with heart failure: A systematic review and meta-analysis of randomised controlled trials | Cardiovascular | Both |  |  | X |
| Garavand 2022 | Advantages and disadvantages of teleworking in healthcare institutions during COVID-19: A systematic review | All health conditions | Both |  |  |  |
| Garcia-Diaz 2023 | Utility of Telehealth Platforms Applied to Burns Management: A Systematic Review | Dermatology | Both | X | X | X |
| Garfan 2021 | Telehealth utilization during the Covid-19 pandemic: A systematic review | All health conditions | Both |  |  |  |
| Garnett 2022 | mHealth Interventions to Support Caregivers of Older Adults: Equity-Focused Systematic Review | Not specified | Both | X |  | X |
| Gasch 2016 | Personalized Health, eLearning, and mHealth Interventions to Improve Nutritional Status | Nutrition | Telephone |  | X | X |
| Gaspar 2021 | eHealth for Addressing Balance Disorders in the Elderly: Systematic Review | CNS | Video |  |  | X |
| Gately 2019 | In-Home Video Telehealth for Dementia Management: Implications for Rehabilitation | CNS | Video | X |  | X |
| Gates 2016 | The effectiveness of telephone counselling in the treatment of illicit drug and alcohol use concerns | Mental health | Telephone | X | X | X |
| Gavine 2022 | Remote provision of breastfeeding support and education: Systematic review and metaâ€analysis | Maternal health | Both |  | X | X |
| Gentili 2022 | The cost-effectiveness of digital health interventions: A systematic review of the literature | All health conditions | Both | X |  |  |
| Gentry 2019 | Evidence for telehealth group-based treatment: A systematic review | Mental health | Both | X |  | X |
| Gentry 2019 | Geriatric Telepsychiatry: Systematic Review and Policy Considerations | Multiple | Both | X |  | X |
| Getty 2019 | Mobile telephone-delivered contingency management interventions promoting behaviour change in individuals with substance use disorders: a meta-analysis | Mental health | Telephone |  | X | X |
| Ghimire 2023 | Virtual prenatal care: A systematic review of pregnant women's and healthcare professionals' experiences, needs, and preferences for quality care | Maternal health | Both |  | X | X |
| Gholamzadeh 2022 | Telemedicine in lung transplant to improve patient-centered care: A systematic review | Surgery | Both |  | X | X |
| Gidora 2019 | Effects of Telenursing Triage and Advice on Healthcare Costs and Resource Use | Not specified | Telephone | X | X |  |
| Gijsbers 2022 | Enablers and barriers in upscaling telemonitoring across geographic boundaries: A scoping review | Multiple | Telemonitoring |  |  |  |
| Gilbert 2020 | Use of virtual consultations in an orthopaedic rehabilitation setting: how do changes in the work of being a patient influence patient preferences? A systematic review and qualitative synthesis | Musckuloskeletal | Both |  |  |  |
| Gill 2021 | Abortion hotlines around the world: a mixed-methods systematic and descriptive review | Maternal health | Telephone |  |  | X |
| Gillam 2022 | Implementation of eHealth to Support Assessment and Decision-making for Residents with Dementia in Long-term Care: Systematic Review | CNS | Video |  |  |  |
| Giustiniani 2023 | Use of Telemedicine to Improve Cognitive Functions and Psychological Well-Being in Patients with Breast Cancer: A Systematic Review of the Current Literature | Cancer | Both |  |  | X |
| Gogovor 2017 | Informing the development of an Internet-based chronic pain self-management program | Pain | Telephone |  |  |  |
| Goldberg 2018 | Measuring Psychiatric Symptoms Remotely: a Systematic Review of Remote Measurement-Based Care | Mental health | Telephone | X |  | X |
| Goldstein 2018 | Telehealth Interventions Designed for Women: an Evidence Map | Maternal health | Both |  | X | X |
| Goncalves 2023 | Usability of Telehealth Systems for Noncommunicable Diseases in Primary Care From the COVID-19 Pandemic Onward: Systematic Review | Multiple | Both |  |  | X |
| Gonzalez 2022 | Practice of Telehealth in Otolaryngology: A Scoping Review in the Era of COVID-19 | ENT | Video | X |  | X |
| Gonzalez-Fraile 2021 | Remotely delivered information, training and support for informal caregivers of people with dementia | CNS | Telephone |  | X | X |
| GonzalezGarcia 2019 | A Review of Randomized Controlled Trials Utilizing Telemedicine for Improving Heart Failure Readmission: Can a Realist Approach Bridge the Translational Divide? | Cardiovascular | Telephone | X | X | X |
| Goodarzi 2023 | Efficacy of virtual interventions for reducing symptoms of depression in community-dwelling older adults: a systematic review | Mental health | Both |  |  | X |
| Goode 2015 | Telephone, print, and Web-based interventions for physical activity, diet, and weight control among cancer survivors: a systematic review | Cancer | Telephone |  | X | X |
| Gordon 2022 | Leveraging Telehealth for Delivery of Palliative Care to Remote Communities: A Rapid Review | End of life | Both |  |  | X |
| Gordon 2023 | Remote care through telehealth for people with inflammatory bowel disease | GI | Both |  |  | X |
| Gorrie 2021 | Benefits and limitations of telegenetics: A literature review | Multiple | Both | X |  | X |
| Gorton 2022 | Introducing Virtual Prehabilitation to a UK Elective Total Hip Replacement service to improve patient outcomes: An overview of the literature and proposed implementation plan | Musckuloskeletal | Virtual NOS |  |  | X |
| Graham 2020 | Telehealth delivery of remote assessment of wheelchair and seating needs for adults and children: a scoping review | Multiple | Both |  | X | X |
| Greenwood 2022 | Telehealth Versus Face-to-face Psychotherapy for Less Common Mental Health Conditions: Systematic Review and Meta-analysis of Randomized Controlled Trials | Mental health | Both | X | X | X |
| Gregersen 2016 | Do telemedical interventions improve quality of life in patients with COPD? A systematic review | Respiratory | Both |  |  | X |
| Grigorovich 2022 | A systematic review of economic analyses of home-based telerehabilitation | Rehabilitation | Both | X |  |  |
| Grona 2018 | Use of videoconferencing for physical therapy in people with musculoskeletal conditions: A systematic review | Rehabilitation | Video | X |  | X |
| Groom 2021 | Telemedicine and Telehealth in Nursing Homes: An Integrative Review | Multiple | Both | X | X | X |
| Gu 2022 | Review of Telemedicine for Management of Acne Patients | Dermatology | Both |  | X | X |
| Guaiana 2021 | A Systematic Review of the Use of Telepsychiatry in Depression | Mental health | Both | X |  | X |
| Guillen 2022 | Utilization of Telehealth Solutions for Patients with Opioid Use Disorder Using Buprenorphine: A Scoping Review | Mental health | Both | X | X | X |
| Gulliver 2015 | Technology-based interventions for tobacco and other drug use in university and college students: a systematic review and meta-analysis | Mental health | Telephone |  | X |  |
| GunesOzturk 2022 | The impact of telehealth applications on pregnancy outcomes and costs in high-risk pregnancy: A systematic review and meta-analysis | Maternal health | Both | X |  | X |
| Gunter 2016 | Current Use of Telemedicine for Post-Discharge Surgical Care: A Systematic Review | Surgery | Both | X | X | X |
| Gupta 2021 | A systematic review of outcomes of remote consultation in ENT | ENT | Both | X | X | X |
| Gutierrez 2022 | A systematic review of telehealth applications in hospital medicine | Multiple | Video | X | X | X |
| Gyawali 2023 | A systematic review of eHealth technologies for breast cancer supportive care | Cancer | Both | X | X | X |
| Haberlin 2018 | The use of eHealth to promote physical activity in cancer survivors: a systematic review | Cancer | Both |  | X |  |
| Hadeler 2021 | Definitions, survey methods, and findings of patient satisfaction studies in teledermatology: a systematic review | Dermatology | Both |  |  | X |
| Haider 2022 | Telemedicine in orthopaedics and its potential applications during COVID-19 and beyond: A systematic review | Musckuloskeletal | Both | X | X | X |
| Haider 2022 | Telemedicine in orthopaedics during COVID-19 and beyond: A systematic review | Musckuloskeletal | Both | X | X | X |
| Haidous 2021 | A review of evaluation approaches for telemental health programs | Mental health | Video | X |  | X |
| Haimi 2022 | Application and implementation of telehealth services designed for the elderly population during the COVID-19 pandemic: A systematic review | All health conditions | Video |  |  |  |
| Hajesmaeel-Gohari 2022 | Travel Prevention Using Telepsychiatric Services: A Review | Mental health | Both |  |  |  |
| Hakala 2017 | Effectiveness of physical activity promoting technology-based distance interventions compared to usual care. Systematic review, meta-analysis and meta-regression | Multiple | Telephone |  | X |  |
| Hall 2022 | Diabetes care in the time of COVID-19: video consultation as a means of diabetes management | Metabolic | Video | X |  | X |
| Halldorsdottir 2020 | Impact of technology-based patient education on modifiable cardiovascular risk factors of people with coronary heart disease: A systematic review | Cardiovascular | Telephone |  | X | X |
| Hallensleben 2019 | eHealth for people with COPD in the Netherlands: a scoping review | Respiratory | Telemonitoring | X |  | X |
| Halligan 2021 | Reducing weight and BMI following gestational diabetes: a systematic review and meta-analysis of digital and telemedicine interventions | Maternal health | Both |  | X | X |
| Hamasaki 2022 | Patient Satisfaction with Telemedicine in Adults with Diabetes: A Systematic Review | Metabolic | Video | X |  | X |
| Hamid 2022 | Internet-based Cognitive Behaviour Therapy for the Prevention, Treatment and Relapse Prevention of Eating Disorders: A Systematic Review | Mental health | Internet-based NOS |  | X | X |
| Hamilton 2018 | Smartphones in the secondary prevention of cardiovascular disease: a systematic review | Cardiovascular | Both | X | X | X |
| Hamine 2015 | Impact of mHealth chronic disease management on treatment adherence and patient outcomes: A systematic review | Multiple | Telephone |  | X | X |
| Hamurajib 2019 | Mobile health (mhealth) intervention for family planning program: An evidence from systematic review | Maternal health | Telephone | X | X |  |
| Han 2020 | Impact of Remote Consultations on Antibiotic Prescribing in Primary Health Care: Systematic Review | Multiple | Both |  |  | X |
| Han 2021 | Effectiveness of telemedicine for cardiovascular disease management: systematic review and meta-analysis | Cardiovascular | Telephone | X |  | X |
| Hanach 2021 | The effectiveness of telemedicine interventions, delivered exclusively during the postnatal period, on postpartum depression in mothers without history or existing mental disorders: A systematic review and meta-analysis | Maternal health | Both | X |  | X |
| Hancock 2019 | Telehealth in palliative care is being described but not evaluated: a systematic review | End of life | Both | X | X | X |
| Hangaard 2023 | The Effectiveness of Telemedicine Solutions for the Management of Type 2 Diabetes: A Systematic Review, Meta-Analysis, and Meta-Regression | Metabolic | Both |  |  | X |
| Hanley 2021 | A systematic review of higher education students' experiences of engaging with online therapy | Mental health | Both |  |  |  |
| Hao 2023 | Effectiveness of telehealth interventions on psychological outcomes and quality of life in community adults during the COVID-19 pandemic: A systematic review and meta-analysis | Mental health | Both |  | X | X |
| Harada 2023 | Effectiveness of telenursing for people with lung cancer at home: A systematic review and meta-analysis | Cancer | Both | X |  | X |
| Harerimana 2019 | The use of technology for mental healthcare delivery among older adults with depressive symptoms: A systematic literature review | Mental health | Both |  |  | X |
| Haridy 2021 | eHealth Technologies for Screening, Diagnosis, and Management of Viral Hepatitis: A Systematic Review | Infectious disease | Multiple |  | X | X |
| Harkey 2020 | Patient Satisfaction with Telehealth in Rural Settings: A Systematic Review | CNS | Both |  |  | X |
| Harst 2019 | Theories predicting end-user acceptance of telemedicine use: Systematic review | All health conditions | Video | X |  |  |
| Hartasanchez 2022 | Remote shared decision making through telemedicine: A systematic review of the literature | Multiple | Both |  |  |  |
| Hassan 2019 | Efficacy of Telepsychiatry in Refugee Populations: A Systematic Review of the Evidence | Mental health | Video |  |  | X |
| Haun 2022 | Delivery of Complementary and Integrative Health Using Virtual Health Resources: A Scoping Review | Multiple | Both |  | X | X |
| Haveland 2022 | Key Considerations in Ensuring a Safe Regional Telehealth Care Model: A Systematic Review | All health conditions | Both |  | X | X |
| Haveman 2019 | Telemedicine in patients with peripheral arterial disease: is it worth the effort? | Cardiovascular | Both |  | X | X |
| Hawley-Hague 2022 | A scoping review of the feasibility, acceptability, and effects of physiotherapy delivered remotely | Multiple | Both | X |  | X |
| Hayotte 2022 | Effects and Acceptability of Technology-Based Physical Activity Interventions in Bariatric Surgery: a Scoping Review | Surgery | Both |  | X | X |
| Hazenberg 2020 | Telehealth and telemedicine applications for the diabetic foot: A systematic review | Metabolic | Both | X |  | X |
| Head 2017 | Telehealth in Palliative Care: A Systematic Review of Patient-Reported Outcomes | End of life | Both |  | X | X |
| Heapy 2015 | A Systematic Review of Technology-assisted Self-Management Interventions for Chronic Pain: Looking Across Treatment Modalities | Pain | Telephone | X |  | X |
| Hechinger 2022 | A Conceptual Model of Experiences With Digital Technologies in Aging in Place: Qualitative Systematic Review and Meta-synthesis | Multiple | Both |  |  | X |
| Heckel 2019 | Are cancer helplines effective in supporting caregivers? A systematic review | Cancer | Telephone |  |  | X |
| Helleman 2019 | The current use of telehealth in ALS care and the barriers to and facilitators of implementation: a systematic review | CNS | Both |  |  |  |
| Hellfritz 2021 | Quality indicators of telemedical care offshore-a scoping review | All health conditions | Both | X | X |  |
| Helsel 2018 | Telemedicine and Mobile Health Technology Are Effective in the Management of Digestive Diseases: A Systematic Review | GI | Both | X |  | X |
| Hennemann 2018 | Internet- and mobile-based aftercare and relapse prevention in mental disorders: A systematic review and recommendations for future research | Mental health | Internet-based NOS |  | X | X |
| Henny 2018 | A Rapid Review of eHealth Interventions Addressing the Continuum of HIV Care (2007-2017) | Infectious disease | Both |  | X | X |
| Henry 2017 | Clinician behaviors in telehealth care delivery: a systematic review | Not specified | Both |  | X |  |
| Hidayah 2022 | Telehealth Model in Improving Health Service during COVID-19 Pandemic | All health conditions | Both |  |  |  |
| Hilty 2018 | An Update on Telepsychiatry and How It Can Leverage Collaborative, Stepped, and Integrated Services to Primary Care | Mental health | Both | X | X | X |
| Hilty 2021 | A Literature Review Comparing Clinicians' Approaches and Skills to In-Person, Synchronous, and Asynchronous Care: Moving Toward Competencies to Ensure Quality Care | Multiple | Both |  |  |  |
| Hilty 2022 | Findings and Guidelines on Provider Technology, Fatigue, and Well-being: Scoping Review | All health conditions | Both | X |  | X |
| Hilty 2023 | A Telehealth and Telepsychiatry Economic Cost Analysis Framework: Scoping Review | Mental health | Both | X | X | X |
| Hincapie 2020 | Implementation and Usefulness of Telemedicine During the COVID-19 Pandemic: A Scoping Review | All health conditions | Both |  |  | X |
| Hoff 2022 | Physician Satisfaction With Telehealth: A Systematic Review and Agenda for Future Research | Multiple | Video |  |  | X |
| Hofmann 2023 | Telemedicine in orthopaedics and trauma surgery during the first year of COVID pandemic: a systematic review | Surgery | Both | X |  | X |
| Holmes 2019 | A systematic review of technology-assisted interventions for co-morbid depression and substance use | Mental health | Video | X | X | X |
| Hong 2019 | Effectiveness of tele-monitoring by patient severity and intervention type in chronic obstructive pulmonary disease patients: A systematic review and meta-analysis | Respiratory | Both |  | X | X |
| Hong 2020 | Digital interventions to facilitate patient-provider communication in cancer care: A systematic review | Cancer | Internet-based NOS |  | X | X |
| Hormaza-Jaramillo 2022 | Effectiveness of Telemedicine Compared with Standard Care for Patients with Rheumatic Diseases: A Systematic Review | Autoimmune | Both |  |  | X |
| Horsley 2020 | Use of real-time videoconferencing to deliver physical therapy services: A scoping review of published and emerging evidence | Multiple | Video |  |  |  |
| Hossain 2019 | Digital interventions for people living with non-communicable diseases in India: A systematic review of intervention studies and recommendations for future research and development | Multiple | Telephone |  | X | X |
| Hosseiniravandi 2020 | Home-based telerehabilitation software systems for remote supervising: a systematic review | Rehabilitation | Both | X |  | X |
| Houlding 2021 | Barriers to Use of Remote Monitoring Technologies Used to Support Patients With COVID-19: Rapid Review | Infectious disease | Both |  |  |  |
| Houser 2023 | Privacy and Security Risk Factors Related to Telehealth Services -- A Systematic Review | Multiple | Both |  |  |  |
| Houston 2022 | Telemedicine in Neurology: A Scoping Review of Key Outcomes in Movement Disorders | Musckuloskeletal | Both | X | X | X |
| Howarth 2018 | The impact of digital health interventions on health-related outcomes in the workplace: A systematic review | All health conditions | Telephone |  | X | X |
| Howes 2021 | Relative Effectiveness of Online Cognitive Behavioural Therapy with Anxious or Depressed Young People: Rapid Review and Meta-analysis | Mental health | Internet-based NOS |  |  | X |
| Howland 2021 | Assessing telehealth interventions for physical activity and sedentary behavior selfâ€management in adults with type 2 diabetes mellitus: An integrative review | Metabolic | Both |  | X |  |
| Huang 2015 | Telehealth interventions versus center-based cardiac rehabilitation of coronary artery disease: A systematic review and meta-analysis | Cardiovascular | Telephone | X |  | X |
| Huang 2019 | The effectiveness of telemedicine on body mass index: A systematic review and meta-analysis | Metabolic | Both |  |  | X |
| Huang 2023 | Diagnostic accuracy of eHealth literacy measurement tools in older adults: a systematic review | Not specified | Telephone |  | X |  |
| Hubschman-Shahar 2022 | Lactation Telehealth in Primary Care: A Systematic Review | Maternal health | Both |  | X | X |
| Hudson 2019 | Impact of telephone delivered casemanagement on the effectiveness of collaborative care for depression and antidepressant use: A systematic review and meta-regression | Mental health | Telephone |  |  | X |
| HungKn 2019 | Effects of telerehabilitation in occupational therapy practice: A systematic review | Occupational therapy | Telephone | X |  | X |
| Hurt 2016 | mHealth Interventions in Low and Middle-Income Countries: A Systematic Review | Not specified | Telephone | X | X | X |
| Hutchesson 2015 | eHealth interventions for the prevention and treatment of overweight and obesity in adults: a systematic review with meta-analysis | Metabolic | Telephone |  |  | X |
| Hvidt 2016 | The impact of telephone crisis services on suicidal users: A systematic review of the past 45 years | Mental health | Telephone | X | X | X |
| Hwang 2015 | A systematic review of the effects of telerehabilitation in patients with cardiopulmonary diseases | Cardiovascular | Both | X |  | X |
| Hwang 2020 | Information and Communications Technology-Based Telehealth Approach for Occupational Therapy Interventions for Cancer Survivors: A Systematic Review | Cancer | Telephone | X | X | X |
| Iacono 2016 | A scoping review of Australian allied health research in ehealth | Rehabilitation | Both |  |  | X |
| ImanuelTonapa 2022 | Outcomes of nurse-led telecoaching intervention for patients with heart failure: A systematic review and meta-analysis of randomised controlled trials | Cardiovascular | Telephone |  | X | X |
| Indraratna 2020 | Mobile Phone Technologies in the Management of Ischemic Heart Disease, Heart Failure, and Hypertension: Systematic Review and Meta-Analysis | Cardiovascular | Telephone |  | X | X |
| Inglis 2015 | Structured telephone support or non-invasive telemonitoring for patients with heart failure | Cardiovascular | Both | X | X | X |
| Irurita-Morales 2022 | Use of Telehealth Among Cancer Survivors: A Scoping Review | Cancer | Both |  |  | X |
| Irving 2018 | Using teledentistry in clinical practice as an enabler to improve access to clinical care: A qualitative systematic review | Denistry | Both | X | X |  |
| Jackson 2016 | EHealth Technologies in Inflammatory Bowel Disease: A Systematic Review | GI | Both | X | X | X |
| Jackson 2016 | A systematic review of the effect of telephone, internet or combined support for carers of people living with Alzheimer's, vascular or mixed dementia in the community | CNS | Both |  | X | X |
| Jackson 2022 | Telemedicine in rheumatology care: A systematic review | Autoimmune | Telephone | X | X | X |
| Jacob 2020 | Social, Organizational, and Technological Factors Impacting Clinicians' Adoption of Mobile Health Tools: Systematic Literature Review | All health conditions | Multiple |  |  |  |
| Jacob 2023 | Assessing the Quality and Impact of eHealth Tools: Systematic Literature Review and Narrative Synthesis | All health conditions | Both |  |  |  |
| Jaen-Extremera 2023 | Effectiveness of Telemedicine for Reducing Cardiovascular Risk: A Systematic Review and Meta-Analysis | All health conditions | Both |  | X | X |
| Jaffar 2022 | Persuasive Technology in an mHealth App Designed for Pelvic Floor Muscle Training Among Women: Systematic Review | Multiple | Multiple |  | X | X |
| Jaffe 2021 | Telehealth use in emergency care during coronavirus disease 2019: a systematic review | Acute/ urgent care | Both |  |  |  |
| Jakob 2022 | Factors Influencing Adherence to mHealth Apps for Prevention or Management of Noncommunicable Diseases: Systematic Review | Multiple | Mobile App | X |  |  |
| James 2021 | Spread, Scale-up, and Sustainability of Video Consulting in Health Care: Systematic Review and Synthesis Guided by the NASSS Framework | Multiple | Video |  |  | X |
| Jang 2021 | A Systematic Review and Meta-Analysis of Telemonitoring Interventions on Severe COPD Exacerbations | Respiratory | Both |  |  | X |
| Janjua 2021 | Telehealth interventions: remote monitoring and consultations for people with chronic obstructive pulmonary disease (COPD) | Respiratory | Both |  | X | X |
| Janssen 2017 | Interdisciplinary eHealth Practice in Cancer Care: A Review of the Literature | Cancer | Video |  |  |  |
| Jansson 2020 | Computer- and Telephone-Delivered Interventions on Patient Outcomes and Resource Utilization in Patients With Orthopaedic Conditions: A Systematic Review and Narrative Synthesis | Musckuloskeletal | Both |  | X | X |
| Jansson 2022 | The effects and safety of telerehabilitation in patients with lower-limb joint replacement: A systematic review and narrative synthesis | Musckuloskeletal | Both |  | X | X |
| Jayakody 2016 | Effectiveness of interventions utilising telephone follow up in reducing hospital readmission within 30 days for individuals with chronic disease: a systematic review | Cardiovascular | Telephone |  |  | X |
| Jenkins-Guarnieri 2015 | Patient Perceptions of Telemental Health: Systematic Review of Direct Comparisons to In-Person Psychotherapeutic Treatments | Mental health | Both |  | X | X |
| Jess 2019 | Video consultations in palliative care: A systematic integrative review | End of life | Video |  |  |  |
| Jhaveri 2015 | Telestroke, tele-oncology and teledialysis: a systematic review to analyse the outcomes of active therapies delivered with telemedicine support | Multiple | Both |  | X | X |
| Jiang 2017 | Beyond face-to-face individual counseling: A systematic review on alternative modes of motivational interviewing in substance abuse treatment and prevention | Mental health | Telephone | X | X |  |
| Jiang 2018 | The comparison of telerehabilitation and face-to-face rehabilitation after total knee arthroplasty: A systematic review and meta-analysis | Musckuloskeletal | Telephone |  |  | X |
| Jiang 2019 | The Cost-Effectiveness of Digital Health Interventions on the Management of Cardiovascular Diseases: Systematic Review | Cardiovascular | Both | X |  |  |
| Jimenez 2021 | The role of health technologies in multicomponent primary care interventions: Systematic review | Multiple | Telephone | X |  | X |
| Jimenez-Molina 2019 | Internet-Based Interventions for the Prevention and Treatment of Mental Disorders in Latin America: A Scoping Review | Mental health | Multiple |  | X | X |
| Jin 2019 | Telehealth interventions for the secondary prevention of coronary heart disease: A systematic review and meta-analysis | Cardiovascular | Both | X | X | X |
| JinChoo 2022 | Effects of telecardiac rehabilitation on coronary heart disease: A PRISMA-compliant systematic review and meta-analysis | Cardiovascular | Telephone |  |  | X |
| Jirasakulsuk 2022 | Real-Time Telerehabilitation in Older Adults With Musculoskeletal Conditions: Systematic Review and Meta-analysis | Musckuloskeletal | Video | X |  | X |
| Johnsen 2021 | eHealth interventions to facilitate work participation: a scoping review | All health conditions | Both |  | X |  |
| Joiner 2017 | Lifestyle interventions based on the diabetes prevention program delivered via eHealth: A systematic review and meta-analysis | Metabolic | Both |  |  | X |
| Jokinen 2021 | Ethical issues related to eHealth: An integrative review | All health conditions | Video |  |  |  |
| Jonasdottir 2022 | Health professionals' perspective towards challenges and opportunities of telehealth service provision: A scoping review | Not specified | Both |  |  |  |
| Jones 2016 | The patient experience of remote telemonitoring for heart failure in the rural setting: a literature review | Cardiovascular | Both |  |  | X |
| Jones 2017 | Development and Use of Health-Related Technologies in Indigenous Communities: Critical Review | Multiple | Telemedicine NOS |  | X |  |
| Jones 2022 | Real-time remote outpatient consultations in secondary and tertiary care: A systematic review of inequalities in invitation and uptake | Multiple | Video | X | X |  |
| Jones 2022 | Scoping review of remote rehabilitation (telerehabilitation) services to support people with vision impairment | CNS | Both | X | X | X |
| Jongerius 2019 | Research-Tested Mobile Apps for Breast Cancer Care: Systematic Review | Cancer | Multiple |  |  | X |
| Jonker 2020 | Feasibility of Perioperative eHealth Interventions for Older Surgical Patients: A Systematic Review | Surgery | Both | X |  |  |
| Jonnagaddala 2021 | From telehealth to virtual primary care in Australia? A Rapid scoping review | Multiple | Both |  |  |  |
| Jonsson 2022 | Internet-delivered psychological treatment as an add-on to treatment as usual for common mental disorders: A systematic review with meta-analysis of randomized trials | Mental health | Both |  | X | X |
| Joo 2021 | A Scoping Review of Telehealth-Assisted Case Management for Chronic Illnesses | Multiple | Both |  |  |  |
| Joo 2022 | Nurse-Led Telehealth Interventions During COVID-19: A Scoping Review | Not specified | Both |  |  |  |
| Jorgensen 2023 | Patients' perspectives on telemedicine in the encounter between healthcare and patients with mental illness: A systematic review | Mental health | Both |  |  | X |
| Joseph 2017 | Patient satisfaction of telephone or video interpreter services compared with in-person services: A systematic review | Multiple | Video |  | X | X |
| Josephine 2017 | Internet- and mobile-based depression interventions for people with diagnosed depression: A systematic review and meta-analysis | Mental health | Multiple |  |  | X |
| Joshi 2021 | A Scoping review of challenges, scope and assessment approaches of teledentistry: an Indian perspective | Denistry | Video |  |  |  |
| Joshi 2021 | Teledermatology in the Control of Skin Neglected Tropical Diseases: A Systematic Review | Dermatology | Video |  | X | X |
| Kabore 2022 | Barriers and facilitators for the sustainability of digital health interventions in low and middle-income countries: A systematic review | Not specified | Both |  |  |  |
| Kabukye 2022 | Digital health in oncology in Africa: A scoping review and cross-sectional survey | Cancer | Both |  |  |  |
| Kahlke 2022 | Systematic review of economic evaluations for internet- and mobile-based interventions for mental health problems | Mental health | Both | X |  |  |
| Kalankesh 2016 | Effect of Telehealth Interventions on Hospitalization Indicators: A Systematic Review | Acute/ urgent care | Both |  |  | X |
| Kamaratos-Sevdalis 2022 | Telehealth has comparable outcomes to in-person diabetic foot care during the COVID-19 pandemic | Metabolic | Video | X | X | X |
| Kamecka 2022 | Telemedicine Technologies Selection for the Posthospital Patient Care Process after Total Hip Arthroplasty | Musckuloskeletal | Both |  |  |  |
| KamiluSulaiman 2023 | The use of mobile health technology in the management of osteoarthritis: A scoping review with scientometric analyses | Musckuloskeletal | Telephone | X | X | X |
| Kane 2022 | Opportunities and Challenges for Professionals in Psychiatry and Mental Health Care Using Digital Technologies During the COVID-19 Pandemic: Systematic Review | Mental health | Both |  |  |  |
| Kane-Gill 2017 | Use of telemedicine to enhance pharmacist services in the nursing facility | Multiple | Telephone |  |  | X |
| Kaonga 2019 | Common themes and emerging trends for the use of technology to support mental health and psychosocial well-being in limited resource settings: A review of the literature: Common themes and emerging trends for eMental health in limited resource settings | Mental health | Both |  |  |  |
| Kappes 2023 | Nurse-led telehealth intervention effectiveness on reducing hypertension: a systematic review | Cardiovascular | Both |  | X | X |
| Karim 2020 | Mobile health applications for improving the sexual health outcomes among adults with chronic diseases: A systematic review | Multiple | Telephone |  | X | X |
| Karlsen 2017 | Experiences of community-dwelling older adults with the use of telecare in home care services: a qualitative systematic review | Geriatric | Telephone |  |  | X |
| Kaur 2022 | Patient satisfaction for telemedicine health services in the era of COVID-19 pandemic: A systematic review | Multiple | Both |  |  | X |
| Kauw 2018 | eHealth in patients with congenital heart disease: a review | Cardiovascular | Video |  | X | X |
| Kavandi 2020 | Factors that affect health information technology adoption by seniors: A systematic review | Geriatric | Video |  |  |  |
| Kaveh 2021 | Telehealth impact on biomedical, psychosocial, and behavioural outcomes in patients with diabetes older than 50 years: A systematic synthesis without meta-analysis | Metabolic | Both |  | X | X |
| Kazemi 2017 | A Systematic Review of the mHealth Interventions to Prevent Alcohol and Substance Abuse | Mental health | Telephone | X | X |  |
| Kebapci 2020 | Effects of eHealth-Based Interventions on Adherence to Components of Cardiac Rehabilitation: A Systematic Review | Cardiovascular | Both |  | X |  |
| Keikha 2022 | Telerehabilitation and Monitoring Physical Activity in Patient with Breast Cancer: Systematic Review | Cancer | Both |  |  |  |
| Kelly 2016 | Telehealth methods to deliver dietary interventions in adults with chronic disease: a systematic review and meta-analysis | Multiple | Both |  | X | X |
| Kelly 2023 | Cost-effectiveness of telehealth-delivered nutrition interventions: a systematic review of randomized controlled trials | Multiple | Telephone | X |  |  |
| Kelson 2019 | Internet-delivered acceptance and commitment therapy for anxiety treatment: Systematic review | Mental health | Both |  |  | X |
| Kemp 2020 | Delivery of Compassionate Mental Health Care in a Digital Technology-Driven Age: Scoping Review | Mental health | Both |  |  |  |
| Kemp 2021 | Effectiveness of family-based eHealth interventions in cardiovascular disease risk reduction: A systematic review | Cardiovascular | Telephone |  | X | X |
| Kepplinger 2016 | Safety and efficacy of thrombolysis in telestroke: A systematic review and meta-analysis | Cardiovascular | Both |  |  | X |
| Keshvardoost 2022 | Telemedicine for Patients with Schizophrenia: A Systematic Literature Review on Applications and Outcomes | Mental health | Video | X | X | X |
| Kew 2016 | Remote versus face-to-face check-ups for asthma | Respiratory | Telephone |  |  | X |
| Kew 2016 | Home telemonitoring and remote feedback between clinic visits for asthma | Respiratory | Telemonitoring |  | X | X |
| Khan 2015 | Telerehabilitation for persons with multiple sclerosis | CNS | Both |  | X | X |
| Khan 2020 | How useful are virtual fracture clinics?: a systematic review | Musckuloskeletal | Video | X | X | X |
| Khanal 2015 | Recommendations for the Improved Effectiveness and Reporting of Telemedicine Programs in Developing Countries: Results of a Systematic Literature Review | All health conditions | Both | X |  |  |
| Khanijahani 2022 | A Systematic Review of the Role of Telemedicine in Blood Pressure Control: Focus on Patient Engagement | Cardiovascular | Telephone |  | X | X |
| Khoja 2021 | Telehealth as an important player in the management of Hepatitis C virus | Infectious disease | Both |  | X | X |
| Khoo 2021 | mHealth Interventions to Address Physical Activity and Sedentary Behavior in Cancer Survivors: A Systematic Review | Cancer | Telephone |  | X |  |
| Khoshrounejad 2021 | Telehealth-Based Services During the COVID-19 Pandemic: A Systematic Review of Features and Challenges | Infectious disease | Video | X | X | X |
| Khosravi 2016 | Investigating the effectiveness of technologies applied to assist seniors: A systematic literature review | Multiple | Telemedicine NOS |  |  |  |
| Kim 2017 | Smart Devices for Older Adults Managing Chronic Disease: A Scoping Review | Multiple | Telephone |  | X | X |
| Kim 2020 | Decision support capabilities of telemedicine in emergency prehospital care: Systematic review | Acute/ urgent care | Both |  |  | X |
| Kim 2023 | Effectiveness of Digital Mental Health Tools to Reduce Depressive and Anxiety Symptoms in Low- and Middle-Income Countries: Systematic Review and Meta-analysis | Mental health | Both |  |  | X |
| Kim 2023 | The Effectiveness of Telenursing Interventions on Patient Outcomes for Colorectal Cancer Patients: A Systematic Review and Meta-Analysis | Cancer | Both |  |  | X |
| Kim 2023 | e-Health for Traumatized Refugees: A Scoping Review | Multiple | Both | X | X | X |
| Kincaid 2021 | Delivering HIV pre-exposure prophylaxis (PrEP) care online: A scoping review | Infectious disease | Both | X |  | X |
| Kip 2018 | eHealth in Treatment of Offenders in Forensic Mental Health: A Review of the Current State | Mental health | Both |  |  |  |
| Kirakalaprathapan 2022 | Efficacy of telehealth in integrated chronic disease management for older, multimorbid adults with heart failure: A systematic review | Cardiovascular | Both |  |  | X |
| Kissi 2022 | Telehealth during COVID-19 pandemic era: a systematic review | Infectious disease | Both |  |  |  |
| KlÃ¶sch 2020 | eHealth systems for the optimised care of patients with type 2 diabetes | Metabolic | Internet-based NOS |  | X | X |
| Knapp 2021 | Use of Patient-Reported Outcome Measures and Patient-Reported Experience Measures Within Evaluation Studies of Telemedicine Applications: Systematic Review | Not specified | Both |  |  | X |
| Knepley 2021 | Impact of Telerehabilitation for Stroke-Related Deficits | Cardiovascular | Telerehabilitation | X | X | X |
| Knight 2017 | Online interventions to address HIV and other sexually transmitted and blood-borne infections among young gay, bisexual and other men who have sex with men: A systematic review | Infectious disease | Video | X | X |  |
| Knutsen 2016 | A Systematic Review of Telemedicine in Autism Spectrum Disorders | CNS | Both | X |  |  |
| Koc 2022 | The effectiveness of telehealth programs on the mental health of women with breast cancer: A systematic review | Cancer | Telephone |  |  | X |
| Kolcun 2020 | Systematic review of telemedicine in spine surgery | Surgery | Both |  | X | X |
| Komariah 2021 | A Scoping Review of Telenursing's Potential as a Nursing Care Delivery Model in Lung Cancer During the COVID-19 Pandemic | Cancer | Telephone |  |  |  |
| Konnyu 2023 | Changes to Prenatal Care Visit Frequency and Telehealth: A Systematic Review of Qualitative Evidence | Maternal health | Telemedicine NOS |  |  |  |
| KorkmazYaylagul 2022 | Trends in Telecare Use among Community-Dwelling Older Adults: A Scoping Review | Geriatric | Both | X |  |  |
| Kostovich 2022 | Outcomes of Telehealth for Wound Care: A Scoping Review | Wound | Both | X | X | X |
| Kotb 2015 | Comparative effectiveness of different forms of telemedicine for individuals with heart failure (HF): a systematic review and network meta-analysis | Cardiovascular | Both |  |  | X |
| Koutras 2015 | Socioeconomic impact of e-Health services in major joint replacement: A scoping review | Musckuloskeletal | Video | X | X | X |
| Kraaijkamp 2021 | eHealth in geriatric rehabilitation: Systematic review of effectiveness, feasibility, and usability | Geriatric | Both | X | X | X |
| Kraef 2020 | Digital telemedicine interventions for patients with multimorbidity: A systematic review and meta-analysis | Multiple | Both |  | X | X |
| Kruse 2016 | Telemedicine Use in Rural Native American Communities in the Era of the ACA: a Systematic Literature Review | All health conditions | Video | X | X |  |
| Kruse 2017 | Telehealth and patient satisfaction: A systematic review and narrative analysis | Not specified | Both |  |  | X |
| Kruse 2017 | The effectiveness of telemedicine in the management of chronic heart disease - a systematic review | Cardiovascular | Telephone | X | X | X |
| Kruse 2018 | Factors influencing the adoption of telemedicine for treatment of military veterans with post-traumatic stress disorder | Mental health | Both |  |  |  |
| Kruse 2019 | Telemonitoring to Manage Chronic Obstructive Pulmonary Disease: Systematic Literature Review | Respiratory | Telemonitoring |  |  |  |
| Kruse 2020 | Measures of Effectiveness, Efficiency, and Quality of Telemedicine in the Management of Alcohol Abuse, Addiction, and Rehabilitation: Systematic Review | Mental health | Both | X | X |  |
| Kruse 2020 | Utilization Barriers and Medical Outcomes Commensurate With the Use of Telehealth Among Older Adults: Systematic Review | Geriatric | Both |  | X | X |
| Kruse 2022 | Facilitators and Barriers to the Adoption of Telemedicine During the First Year of COVID-19: Systematic Review | Multiple | Both |  |  | X |
| Kruse 2022 | Leveraging Telehealth for the Management of Breast Cancer: A Systematic Review | Cancer | Both |  |  | X |
| Kruse 2022 | Leveraging Mobile Health to Manage Mental Health/Behavioral Health Disorders: Systematic Literature Review | Mental health | Both |  | X | X |
| Kruse 2023 | Analyzing the Effect of Telemedicine on Domains of Quality Through Facilitators and Barriers to Adoption: Systematic Review | Multiple | Both |  | X | X |
| Kruse 2023 | Analyzing the Effectiveness of mHealth to Manage Diabetes Mellitus Among Adults Over 50: A Systematic Literature Review | Metabolic | Telemedicine NOS |  |  | X |
| Kruse 2023 | Leveraging mHealth for the Treatment and Management of PLHIV | Infectious disease | Both | X | X | X |
| Krzyzaniak 2021 | The effectiveness of telehealth versus face-to face interventions for anxiety disorders: A systematic review and meta-analysis | Mental health | Both |  |  | X |
| Krzyzaniak 2023 | Telerehabilitation versus face-to-face rehabilitation in the management of musculoskeletal conditions: a systematic review and meta-analysis | Musckuloskeletal | Both | X |  | X |
| Kuan 2022 | Efficacy of telemedicine for the management of cardiovascular disease: a systematic review and meta-analysis | Cardiovascular | Both |  |  | X |
| Kunutsor 2022 | Are remote clinical assessments a feasible and acceptable method of assessment? A systematic review | Not specified | Video | X |  | X |
| KuriakoseKuzhiyanjal 2023 | Management of Inflammatory Bowel Disease Using E-Health Technologies: A Systematic Review and Meta-Analysis | GI | Both |  | X | X |
| Kusuma 2022 | A review of the effectiveness of telemedicine in glycemic control in diabetes mellitus patients | Metabolic | Telephone |  | X | X |
| Kwok 2022 | Nurse-led telehealth interventions for symptom management in patients with cancer receiving systemic or radiation therapy: a systematic review and meta-analysis | Cancer | Both |  | X | X |
| Kwon 2023 | The Effectiveness of eHealth Interventions on Lifestyle Modification in Patients With Nonalcoholic Fatty Liver Disease: Systematic Review and Meta-analysis | GI | Telephone |  |  | X |
| Laar 2022 | Perspectives of health care professionals' on delivering mHealth sexual and reproductive health services in rural settings in low-and-middle-income countries: a qualitative systematic review | Maternal health | Telephone |  |  |  |
| Ladds 2023 | The impact of remote care approaches on continuity in primary care: a mixed-studies systematic review | Multiple | Both |  | X |  |
| Lamb 2019 | Update of Recent Literature on Remotely Delivered Psychotherapy Interventions for Anxiety and Depression | Mental health | Both |  |  | X |
| Lampickiene 2022 | Healthcare Professionals' Experience of Performing Digital Care Visits-A Scoping Review | Infectious disease | Video |  |  | X |
| Langarizadeh 2017 | Telemental Health Care, an Effective Alternative to Conventional Mental Care: a Systematic Review | Mental health | Both |  |  |  |
| Lapointe 2020 | Impact of telemedicine on diagnosis, clinical management and outcomes in rural trauma patients: A rapid review | Mental health | Video |  |  | X |
| Lara-Cinisomo 2021 | A Systematic Review of Technology-Based Prevention and Treatment Interventions for Perinatal Depression and Anxiety in Latina and African American Women | Mental health | Telephone |  |  | X |
| Larson 2018 | The Effect of Telehealth Interventions on Quality of Life of Cancer Patients: A Systematic Review and Meta-Analysis | Cancer | Both |  |  | X |
| Lathan 2022 | Diagnostic accuracy of telemedicine for detection of surgical site infection: a systematic review and meta-analysis | Surgery | Both | X |  |  |
| Lattie 2019 | Digital Mental Health Interventions for Depression, Anxiety, and Enhancement of Psychological Well-Being Among College Students: Systematic Review | Mental health | Telephone | X |  | X |
| Lau 2020 | Personalised eHealth interventions in adults with overweight and obesity: A systematic review and meta-analysis of randomised controlled trials | Metabolic | Multiple |  |  | X |
| Laukka 2020 | Health care professionals' experiences of patient-professional communication over patient portals: Systematic review of qualitative studies | Not specified | Both |  |  | X |
| Laursen 2022 | Effectiveness of Telemedicine in Managing Diabetes in Pregnancy: A Systematic Review and Meta-Analysis | Maternal health | Telephone |  |  | X |
| Laver 2020 | Telerehabilitation services for stroke | Cardiovascular | Both |  | X | X |
| Law 2022 | Cost-effectiveness of telehealth-delivered diet and exercise interventions: A systematic review | Metabolic | Both | X |  |  |
| Lawes-Wickwar 2018 | Application and Effectiveness of Telehealth to Support Severe Mental Illness Management: Systematic Review | Mental health | Telephone |  | X | X |
| Lazarus 2020 | Telestroke strategies to enhance acute stroke management in rural settings: A systematic review and meta-analysis | Cardiovascular | Both | X |  | X |
| Leach 2022 | Digital Health Interventions for Mental Health, Substance Use, and Co-occurring Disorders in the Criminal Justice Population: A Scoping Review | Multiple | Video | X | X | X |
| LeBlanc 2020 | Patient and provider perspectives on eHealth interventions in Canada and Australia: a scoping review | Not specified | Both |  |  |  |
| Lee 2016 | Effectiveness of mHealth interventions for maternal, newborn and child health in low-and middle-income countries: Systematic review and meta-analysis | Maternal health | Both |  | X | X |
| Lee 2016 | Web-based interventions for prevention and treatment of perinatal mood disorders: a systematic review | Maternal health | Telephone | X |  | X |
| Lee 2017 | Telemedicine for the Management of Glycemic Control and Clinical Outcomes of Type 1 Diabetes Mellitus: A Systematic Review and Meta-Analysis of Randomized Controlled Studies | Metabolic | Telephone |  | X | X |
| Lee 2018 | Do telehealth interventions improve oral anticoagulation management? A systematic review and meta-analysis | Cardiovascular | Both |  |  | X |
| Lee 2018 | Telemedicine Cost-Effectiveness for Diabetes Management: A Systematic Review | Metabolic | Both | X |  |  |
| Lee 2022 | Nurse-led Telehealth Intervention for Rehabilitation (Telerehabilitation) Among Community-Dwelling Patients With Chronic Diseases: Systematic Review and Meta-analysis | Multiple | Both |  | X | X |
| Lee 2023 | Digital Health Interventions for Adult Patients with Cancer Evaluated in Randomized Controlled Trials: Scoping Review | Cancer | Telephone | X | X | X |
| Lee 2023 | Virtual healthcare solutions for cardiac rehabilitation: a literature review | Cardiovascular | Telephone |  |  | X |
| Leochico 2020 | Challenges to the Emergence of Telerehabilitation in a Developing Country: A Systematic Review | Multiple | Both |  | X |  |
| Leonardsen 2020 | Patient experiences with technology enabled care across healthcare settings- a systematic review | Somatic diseases | Both |  |  | X |
| Leone 2021 | Exploration of implementation, financial and technical considerations within allied health professional (AHP) telehealth consultation guidance: a scoping review including UK AHP professional bodies' guidance | All health conditions | Both |  |  |  |
| Leong 2022 | Characteristics of Mobile Health Platforms for Depression and Anxiety: Content Analysis through a Systematic Review of the Literature and Systematic Search of Two App Stores | Mental health | Internet-based NOS |  |  |  |
| Leon-Salas 2023 | Telemedicine for neurological diseases: A systematic review and meta-analysis | CNS | Both | X | X | X |
| Leslie 2021 | The Effectiveness of Exercise Interventions Supported by Telerehabilitation For Recently Hospitalized Adult Medical Patients: A Systematic Review | Acute/ urgent care | Video | X |  | X |
| Levoy 2022 | Palliative care delivery changes during COVID-19 and enduring implications in oncology nursing: a rapid review | End of life | Both |  |  |  |
| Lewinski 2022 | Telehealth for the Longitudinal Management of Chronic Conditions: Systematic Review | Multiple | Both |  | X | X |
| Lewis 2018 | Internet-based cognitive and behavioural therapies for post-traumatic stress disorder (PTSD) in adults | Mental health | Both | X |  | X |
| Li 2019 | A review of technology-based interventions in improving type 2 diabetes management in Chinese Americans | Metabolic | Both |  |  | X |
| Li 2020 | Technology-supported lifestyle interventions to improve maternal-fetal outcomes in women with gestational diabetes mellitus: A meta-analysis | Maternal health | Telephone |  |  | X |
| Li 2020 | The effectiveness of self-management of hypertension in adults using mobile health: Systematic review and meta-analysis | Cardiovascular | Mobile App | X | X | X |
| Li 2021 | Perceptions of patients with chronic obstructive pulmonary disease towards telemedicine: A qualitative systematic review | Respiratory | Telephone |  |  | X |
| Li 2021 | Effect of telehealth interventions on quality of life in cancer survivors: A systematic review and meta-analysis of randomized controlled trials | Cancer | Both |  |  | X |
| Li 2021 | Connecting the World of Healthcare Virtually: A Scoping Review on Virtual Care Delivery | All health conditions | Both |  |  | X |
| Li 2022 | The effectiveness of e-Health interventions on caregiver burden, depression, and quality of life in informal caregivers of patients with cancer: A systematic review and meta-analysis of randomized controlled trials | Cancer | Both |  |  | X |
| Li 2022 | Transition of Mental Health Service Delivery to Telepsychiatry in Response to COVID-19: A Literature Review | Mental health | Both | X | X | X |
| Liegl 2015 | Guided self-help interventions for irritable bowel syndrome: a systematic review and meta-analysis | GI | Telephone |  |  | X |
| Liem 2021 | Digital Health Applications in Mental Health Care for Immigrants and Refugees: A Rapid Review | Mental health | Video |  |  | X |
| Lieneck 2020 | Rapid Telehealth Implementation during the COVID-19 Global Pandemic: A Rapid Review | Infectious disease | Both |  |  |  |
| Lieneck 2021 | Outpatient Telehealth Implementation in the United States during the COVID-19 Global Pandemic: A Systematic Review | All health conditions | Video |  |  |  |
| Lim 2019 | Health Professionals' and Postpartum Women's Perspectives on Digital Health Interventions for Lifestyle Management in the Postpartum Period: A Systematic Review of Qualitative Studies | Maternal health | Both |  |  | X |
| Lim 2022 | Remote Care Management for Older Adult Populations With Elevated Prevalence of Depression or Anxiety and Comorbid Chronic Medical Illness: A Systematic Review | Mental health | Both | X |  | X |
| Lima 2016 | Information and communication technologies for adherence to antiretroviral treatment in adults with HIV/AIDS | Infectious disease | Telephone | X |  |  |
| Lin 2019 | Telemedicine-delivered treatment interventions for substance use disorders: A systematic review | Mental health | Video | X | X | X |
| Lin 2020 | The effectiveness of online interventions for patients with gynecological cancer: An integrative review | Cancer | Both |  |  | X |
| Lin 2021 | The Efficacy of Synchronous Teletherapy Versus In-Person Therapy: A Meta-Analysis of Randomized Clinical Trials | Mental health | Both | X |  |  |
| Lin 2023 | Telemedicine along the cascade of care for substance use disorders during the COVID-19 pandemic in the United States | Mental health | Both | X | X | X |
| Linde 2020 | Intimate partner violence and electronic health interventions: Systematic review and meta-analysis of randomized trials | Assault | Telephone |  | X |  |
| Linn 2021 | Digital Health Interventions among People Living with Frailty: A Scoping Review | Geriatric | Both | X | X | X |
| Liptrott 2018 | Acceptability of telephone support as perceived by patients with cancer: A systematic review | Cancer | Telephone |  |  | X |
| Litchfield 2021 | Impact of COVID-19 on the digital divide: A rapid review | All health conditions | Video | X |  |  |
| Liu 2020 | Effectiveness of Mobile App-Assisted Self-Care Interventions for Improving Patient Outcomes in Type 2 Diabetes and/or Hypertension: Systematic Review and Meta-Analysis of Randomized Controlled Trials | Multiple | Both |  | X | X |
| Liu 2022 | Effects of internet-based cognitive behavioral therapy on anxiety and depression symptoms in cancer patients: A meta-analysis | Cancer | Both |  |  | X |
| Liu 2022 | The effectiveness of telemedicine interventions on women with postpartum depression: A systematic review and meta-analysis | Maternal health | Both |  |  | X |
| Lobo 2022 | Technology-based support for stroke caregiving: A rapid review of evidence | Cardiovascular | Both |  |  | X |
| Lopez-Del-Hoyo 2023 | Effects of eHealth interventions on stress reduction and mental health promotion in healthcare professionals: A systematic review | Mental health | Both |  |  | X |
| Lopez-Liria 2022 | Teledermatology versus Face-to-Face Dermatology: An Analysis of Cost-Effectiveness from Eight Studies from Europe and the United States | Dermatology | Telephone | X |  | X |
| Lowry 2020 | Optimising health outcomes via pharmacist delivered telehealth medicines management: a systematic review | Multiple | Telephone |  |  | X |
| Lu 2022 | A Systematic Review of Physical Examination Components Adapted for Telemedicine | Multiple | Both | X |  |  |
| Luisa 2021 | Telerehabilitation for people with aphasia: A systematic review and meta-analysis | Cardiovascular | Video | X |  | X |
| Lundereng 2023 | Health Care Professionals' Experiences and Perspectives on Using Telehealth for Home-based Palliative Care: Scoping Review | End of life | Both |  |  | X |
| Lunney 2018 | Impact of Telehealth Interventions on Processes and Quality of Care for Patients With ESRD | GU | Both |  | X | X |
| Luo 2020 | A critical literature review of dyadic web-based interventions to support cancer patients and their caregivers, and directions for future research | Cancer | Internet-based NOS |  |  | X |
| Lv 2021 | Effects of Telemedicine and mHealth on Systolic Blood Pressure Management in Stroke Patients: Systematic Review and Meta-Analysis of Randomized Controlled Trials | Cardiovascular | Telephone |  |  | X |
| Lyu 2022 | Benefits of and barriers to telehealth for the informal caregivers of elderly individuals in rural areas: A scoping review | Geriatric | Both |  |  |  |
| Ma 2019 | The effectiveness of electronic health interventions on blood pressure control, self-care behavioural outcomes and psychosocial well-being in patients with hypertension: A systematic review and meta-analysis | Cardiovascular | Telephone |  | X | X |
| Ma 2022 | Telemedicine application in patients with chronic disease: a systematic review and meta-analysis | Multiple | Internet-based NOS |  | X | X |
| Macdonald 2018 | Enablers and barriers to using two-way information technology in the management of adults with diabetes: A descriptive systematic review | Metabolic | Telephone |  |  |  |
| Madanian 2020 | Disaster eHealth: Scoping Review | Not specified | Video |  |  |  |
| Madhavan 2019 | Telepsychiatry in intellectual disability psychiatry: Literature review | Mental health | Video | X |  | X |
| Mahdavi 2022 | Teledentistry during COVID-19 pandemic: scientometric and content analysis approach | Denistry | Both |  |  |  |
| Mahdi 2022 | The promise of telemedicine in Pakistan: A systematic review | All health conditions | Video | X |  | X |
| Mahmoud 2021 | Usability of Telemedicine in Physical Therapy Rehabilitation: Systematic review | Rehabilitation | Video |  |  | X |
| Mahmoud 2022 | Telemedicine in Low- and Middle-Income Countries During the COVID-19 Pandemic: A Scoping Review | All health conditions | Both |  |  |  |
| Maisto 2021 | Digital interventions for psychological comorbidities in chronic diseases-a systematic review | Multiple | Both |  |  | X |
| Malandraki 2021 | Telehealth for Dysphagia Across the Life Span: Using Contemporary Evidence and Expertise to Guide Clinical Practice During and After COVID-19 | ENT | Video | X |  |  |
| Manby 2021 | Effectiveness of eHealth Interventions for HIV Prevention and Management in Sub-Saharan Africa: Systematic Review and Meta-analyses | Infectious disease | Telephone |  | X | X |
| Manyati 2021 | Exploring the effectiveness of telehealth interventions for diagnosis, contact tracing and care of Corona Virus Disease of 2019 (COVID19) patients in sub Saharan Africa: a rapid review | Infectious disease | Both |  |  | X |
| Manyazewal 2021 | The potential use of digital health technologies in the African context: a systematic review of evidence from Ethiopia | Not specified | Both | X |  |  |
| Mao 2020 | Impact and efficacy of mobile health intervention in the management of diabetes and hypertension: a systematic review and meta-analysis | Multiple | Both |  | X | X |
| Maresca 2020 | Tele-Neuro-Rehabilitation in Italy: State of the Art and Future Perspectives | CNS | Video |  |  | X |
| Margineanu 2022 | eHealth in TB clinical management | Infectious disease | Video | X | X |  |
| Mariano 2021 | Online teletherapy for chronic pain: A systematic review | Pain | Both |  | X | X |
| Mark 2022 | Addiction Treatment and Telehealth: Review of Efficacy and Provider Insights During the COVID-19 Pandemic | Mental health | Both | X | X |  |
| Markert 2021 | The Use of Telehealth Technology to Support Health Coaching for Older Adults: Literature Review | Not specified | Both |  |  | X |
| Marks 2022 | The Health Economic Impact of Musculoskeletal Physiotherapy Delivered by Telehealth: A Systematic Review | Musckuloskeletal | Both | X |  |  |
| Marsh 2021 | Diabetes management among underserved older adults through telemedicine and community health workers | Metabolic | Video |  | X | X |
| Marthick 2021 | Supportive Care Interventions for People With Cancer Assisted by Digital Technology: Systematic Review | Cancer | Both |  |  | X |
| Martin 2018 | A systematic review of the factors that influence the quality and effectiveness of telesupervision for health professionals | All health conditions | Both |  |  |  |
| Martin 2020 | Teledermatology for Skin Cancer: The Australian Experience | Cancer | Video | X | X | X |
| MartinezAgulleiro 2023 | A systematic review of digital interventions for smoking cessation in patients with serious mental illness | Mental health | Video | X | X | X |
| Martinez-Alcala 2016 | Information and Communication Technologies in the Care of the Elderly: Systematic Review of Applications Aimed at Patients With Dementia and Caregivers | CNS | Both |  |  |  |
| Martinez-Guijarro 2023 | Feasibility and efficacy of telerehabilitation in the management of patients with head and neck cancer during and after oncological treatment: A systematic review | Cancer | Both |  | X | X |
| Martiniuk 2023 | A review of risks, adverse effects and mitigation strategies when delivering mental health services using telehealth | Mental health | Both |  | X |  |
| Martin-Martin 2021 | Behavior Change Techniques and the Effects Associated With Digital Behavior Change Interventions in Sedentary Behavior in the Clinical Population: A Systematic Review | All health conditions | Telephone |  | X | X |
| Marx 2018 | Is telehealth effective in managing malnutrition in community-dwelling older adults? A systematic review and meta-analysis | Nutrition | Telephone | X |  | X |
| Marzorati 2018 | Telemedicine Use Among Caregivers of Cancer Patients: Systematic Review | Cancer | Telephone |  |  | X |
| Mashhadi 2021 | Post Discharge mHealth and Teach-Back Communication Effectiveness on Hospital Readmissions: A Systematic Review | Not specified | Multiple |  |  | X |
| Maspero 2020 | Available technologies, applications and benefits of teleorthodontics. A literature review and possible applications during the COVID-19 pandemic | Denistry | Video |  |  | X |
| Massie 2022 | The Role of Optometry in the Delivery of Eye Care via Telehealth: A Systematic Literature Review | CNS | Video | X |  |  |
| Massoudi 2019 | The effectiveness and cost-effectiveness of e-health interventions for depression and anxiety in primary care: A systematic review and meta-analysis | Mental health | Both | X |  | X |
| Mastronardo 2021 | Digital health technologies for osteopaths and allied healthcare service providers: A scoping review | Musckuloskeletal | Both |  | X | X |
| Matamala-Gomez 2020 | The Role of Engagement in Teleneurorehabilitation: A Systematic Review | CNS | Both | X | X | X |
| Matsumoto 2021 | Effectiveness of Videoconference-Delivered Cognitive Behavioral Therapy for Adults With Psychiatric Disorders: Systematic and Meta-Analytic Review | Mental health | Both |  |  | X |
| Matthew-Maich 2016 | Designing, Implementing, and Evaluating Mobile Health Technologies for Managing Chronic Conditions in Older Adults: A Scoping Review | Multiple | Both | X | X | X |
| Maulana 2022 | The Potential of Cardiac Telerehabilitation as Delivery Rehabilitation Care Model in Heart Failure during COVID-19 and Transmissible Disease Outbreak: A Systematic Scoping Review of the Latest RCTs | Cardiovascular | Video | X | X |  |
| Mboweni 2022 | The Impact of The COVID-19 Pandemic on the Management of Chronic Disease in South Africa: A Systematic Review | Multiple | Telephone |  |  |  |
| Mbunge 2022 | Virtual healthcare services and digital health technologies deployed during coronavirus disease 2019 (COVID-19) pandemic in South Africa: a systematic review | Infectious disease | Both |  |  |  |
| Mbunge 2022 | Are we there yet? Unbundling the potential adoption and integration of telemedicine to improve virtual healthcare services in African health systems | All health conditions | Video |  |  |  |
| McCall 2021 | A systematic review of telehealth interventions for managing anxiety and depression in African American adults | Mental health | Both | X | X | X |
| McCarroll 2017 | Effectiveness of mobile health (mHealth) interventions for promoting healthy eating in adults: A systematic review | All health conditions | Telephone |  | X | X |
| McCleery 2021 | Diagnostic test accuracy of telehealth assessment for dementia and mild cognitive impairment | CNS | Both | X |  | X |
| McDaniel 2022 | Telehealth delivery of motivational interviewing for diabetes management: A systematic review of randomized controlled trials | Metabolic | Both | X | X | X |
| McDonnell 2022 | The efficacy of remote virtual care in comparison to traditional clinical visits for elective orthopaedic patients: A meta-analysis of prospective randomised controlled trials | Musckuloskeletal | Video | X |  | X |
| McDougall 2017 | Telerheumatology: A Systematic Review | Autoimmune | Both | X |  |  |
| McFarland 2021 | The effect of telehealth versus usual care for home-care patients with long-term conditions: A systematic review, meta-analysis and qualitative synthesis | Multiple | Both | X |  | X |
| McHugh 2022 | Effectiveness of remote exercise programs in reducing pain for patients with knee osteoarthritis: A systematic review of randomized trials | Musckuloskeletal | Both |  |  | X |
| McKoy 2015 | Are Telehealth Technologies for Hypertension Care and Self-Management Effective or Simply Risky and Costly? | Cardiovascular | Telephone |  |  |  |
| McLaughlin 2021 | Associations Between Digital Health Intervention Engagement, Physical Activity, and Sedentary Behavior: Systematic Review and Meta-analysis | Metabolic | Multiple |  | X |  |
| McLendon 2017 | Interactive video telehealth models to improve access to diabetes specialty care and education in the rural setting: A systematic review | Metabolic | Both | X |  | X |
| MdFadzil 2022 | A Scoping Review for Usage of Telerehabilitation among Older Adults with Mild Cognitive Impairment or Cognitive Frailty | CNS | Both |  | X | X |
| MegatKamaruddin 2023 | A Meta-Analysis of eHealth Interventions on Ischaemic Heart Disease Health Outcomes | Cardiovascular | Both |  | X | X |
| Mehraeen 2023 | Telemedicine technologies and applications in the era of COVID-19 pandemic: A systematic review | Infectious disease | Both |  |  |  |
| Meinhardt 2023 | The Role of Telemedicine for Evaluation and Management of Dizzy Patients: A Systematic Review | Vesibular disease | Both |  |  |  |
| Melian 2022 | Teleconsultation in orthopaedic surgery: A systematic review and meta-analysis of patient and physician experiences | Surgery | Video | X | X | X |
| Melton 2021 | Describing the delivery of clinical pharmacy services via telehealth: A systematic review | All health conditions | Both |  |  |  |
| Meneses 2023 | Experiences of Women With Breast Cancer Using Telehealth: A Qualitative Systematic Review | Cancer | Both |  |  | X |
| Meng 2022 | Service process factors affecting patients' and clinicians' experiences on rapid teleconsultation implementation in out-patient neurology services during COVID-19 pandemic: a scoping review | CNS | Both |  |  | X |
| Meyer 2015 | Telepathology Impacts and Implementation Challenges | Multiple | Video | X |  |  |
| Miao 2022 | Implementation of Web-Based Psychosocial Interventions for Adults With Acquired Brain Injury and Their Caregivers: Systematic Review | CNS | Telephone |  |  |  |
| Michaelchuk 2022 | Design and delivery of home-based telehealth pulmonary rehabilitation programs in COPD: A systematic review and meta-analysis | Respiratory | Both | X |  | X |
| Mikkonen 2022 | Randomised controlled trials addressing how the clinical application of information and communication technology impacts the quality of patient care-A systematic review and meta-analysis | Multiple | Telephone |  | X | X |
| Miles 2017 | Barriers and facilitators of effective self-management in asthma: systematic review and thematic synthesis of patient and healthcare professional views | Respiratory | Video |  |  |  |
| Mileski 2017 | Adopting Telemedicine for the Self-Management of Hypertension: Systematic Review | Cardiovascular | Both | X |  | X |
| Miller 2022 | Shaping the future of teledermatology: a literature review of patient and provider satisfaction with synchronous teledermatology during the COVID-19 pandemic | Dermatology | Both |  |  | X |
| Ming 2016 | Telemedicine technologies for diabetes in pregnancy: A systematic review and meta-analysis | Maternal health | Telephone |  |  | X |
| Mistry 2022 | Inequity in Access and Delivery of Virtual Care Interventions: A Scoping Review | Multiple | Both | X |  |  |
| Mitchell 2021 | Economic Evaluations of Internet-Based Psychological Interventions for Anxiety Disorders and Depression: A Systematic Review | Mental health | Telephone | X |  |  |
| Moecke 2023 | Scoping review of telehealth use by Indigenous populations from Australia, Canada, New Zealand, and the United States | Multiple | Both |  |  |  |
| Moeller 2022 | Patients' acceptance of video consultations in the mental health services: A systematic review and synthesis of qualitative research | Mental health | Video |  |  | X |
| Moentmann 2021 | Using telemedicine to facilitate social distancing in otolaryngology: A systematic review | ENT | Both |  |  | X |
| Moghimi 2021 | The efficacy of ehealth interventions for the treatment of adults diagnosed with full or subthreshold binge eating disorder: Systematic review and meta-analysis | Mental health | Both |  | X | X |
| Mohammadi 2020 | A systematic review of the use of telemedicine in the military forces worldwide | Multiple | Both |  |  |  |
| Mohammadi 2021 | Effectiveness of eHealth Tools for Hip and Knee Arthroplasty: A Systematic Review | Musckuloskeletal | Both | X | X | X |
| Mohammadzadeh 2022 | Telecardiology interventions for patients with cardiovascular Disease: A systematic review on characteristics and effects | Cardiovascular | Both | X | X | X |
| Mohammadzadeh 2022 | Role of Telemental Health During the COVID-19 Pandemic: An Early Review | Mental health | Both |  |  |  |
| Mohammadzadeh 2023 | Telemedicine for Patient Management in Remote Areas and Underserved Populations | All health conditions | Both |  |  |  |
| Moise 2023 | Lessons from digital technology-enabled health interventions implemented during the coronavirus pandemic to improve maternal and birth outcomes: a global scoping review | Maternal health | Both |  |  |  |
| Mokaya 2022 | Clinical and patient-centered implementation outcomes of mHealth interventions for type 2 diabetes in low-and-middle income countries: a systematic review | Metabolic | Video | X |  | X |
| Mold 2019 | Electronic Consultation in Primary Care Between Providers and Patients: Systematic Review | Multiple | Both | X | X | X |
| Molini-Avejonas 2015 | A systematic review of the use of telehealth in speech, language and hearing sciences | CNS | Telephone |  |  |  |
| Monaghesh 2020 | The role of telehealth during COVID-19 outbreak: A systematic review based on current evidence | Infectious disease | Video |  |  | X |
| Montanes-Masias 2022 | Online psychological interventions to improve symptoms in multiple sclerosis: A systematic review: Online psychological interventions in Multiple Sclerosis | CNS | Both |  |  | X |
| Moon 2022 | Digital and Mobile Health Technology in Collaborative Behavioral Health Care: Scoping Review | Mental health | Video | X |  | X |
| Moreira 2022 | Telephone calls and glycemic control in type 2 diabetes: A PRISMA-compliant systematic review and meta-analysis of randomized clinical trials | Metabolic | Telephone |  |  | X |
| Moretto 2019 | Telephone follow-up as a nursing intervention for patients receiving outpatient chemotherapy: integrative review | Cancer | Video |  | X | X |
| Morimoto 2022 | Web Portals for Patients with Chronic Diseases: Scoping Review of the Functional Features and Theoretical Frameworks of Telerehabilitation Platforms | Multiple | Both |  |  |  |
| Morris 2022 | The role of digital health technology in rural cancer care delivery: A systematic review | Cancer | Telephone | X |  | X |
| Morrison 2020 | The Feasibility of Exercise Interventions Delivered via Telehealth for People Affected by Cancer: A Rapid Review of the Literature | Cancer | Both | X |  | X |
| Morrison 2022 | Understanding the use of telehealth in the context of the Family Nurse Partnership and other early years home visiting programmes: A rapid review | Multiple | Both |  |  |  |
| Mostafaei 2022 | Experiences of patients and providers while using telemedicine in cancer care during COVID-19 pandemic: a systematic review and meta-synthesis of qualitative literature | Cancer | Both |  |  | X |
| MoukhtarHammad 2023 | The pandemic, telemedicine, and andrology: what have we learned? | Multiple | Both | X |  | X |
| Moulaei 2022 | Telerehabilitation for upper limb disabilities: a scoping review on functions, outcomes, and evaluation methods | Multiple | Video |  | X | X |
| Mounessa 2018 | A systematic review of satisfaction with teledermatology | Dermatology | Both |  |  | X |
| Muftah 2023 | Telehealth interventions in patients with chronic liver diseases: a systematic review | Surgery | Video |  |  | X |
| Muir 2020 | Barriers and facilitators of videoconferencing psychotherapy implementation in veteran mental health care environments: a systematic review | Mental health | Video |  |  | X |
| Munoz-Tomas 2023 | Telerehabilitation as a Therapeutic Exercise Tool versus Face-to-Face Physiotherapy: A Systematic Review | Not specified | Both | X | X | X |
| Murphie 2019 | Remote consulting with telemonitoring of continuous positive airway pressure usage data for the routine review of people with obstructive sleep apnoea hypopnoea syndrome: A systematic review | CNS | Both | X | X | X |
| Murphy 2020 | Virtual geriatric clinics and the COVID-19 catalyst: a rapid review | Geriatric | Video |  |  | X |
| Murthy 2022 | Digital health innovations for non-communicable disease management during the COVID-19 pandemic: A rapid scoping review | Multiple | Both | X |  | X |
| Murugesu 2020 | Evaluating the use of telemedicine in gynaecological practice: A systematic review | Maternal health | Both |  |  |  |
| Mwase 2022 | The role of digital health in palliative care for people living with HIV in sub-Saharan Africa: A systematic review | Infectious disease | Telephone |  | X | X |
| Myers-Ingram 2023 | Effectiveness of eHealth weight management interventions in overweight and obese adults from low socioeconomic groups: a systematic review | Metabolic | Telephone |  | X | X |
| Nacke 2021 | A systematic review of reach, adoption, implementation and maintenance of Internet-based interventions to prevent eating disorders in adults | Mental health | Telephone | X |  | X |
| Nagase 2022 | Effectiveness of remote home monitoring for patients with Chronic Obstructive Pulmonary Disease (COPD): systematic review | Respiratory | Telephone | X | X | X |
| Namasivayam 2022 | The use of telehealth in the provision of after-hours palliative care services in rural and remote Australia: A scoping review | End of life | Both |  |  |  |
| Nanda 2021 | A Review of Patient Satisfaction and Experience with Telemedicine: A Virtual Solution During and Beyond COVID-19 Pandemic | Not specified | Both |  |  | X |
| Naoum 2021 | Economic Evaluation of Digital Health Interventions in Palliative Care: A Systematic Review of the Literature | End of life | Both | X |  |  |
| Narasimha 2017 | Designing Telemedicine Systems for Geriatric Patients: A Review of the Usability Studies | CNS | Both | X |  | X |
| Naslund 2022 | Economic evaluation and costs of telepsychiatry programmes: A systematic review | Mental health | Both | X |  |  |
| Ndayishimiye 2023 | A systematic scoping review of digital health technologies during COVID-19: a new normal in primary health care delivery | Multiple | Video |  |  |  |
| Neher 2019 | Implementing internet-delivered cognitive behavioural therapy for patients with cardiovascular disease and psychological distress: a scoping review | Cardiovascular | Internet-based NOS | X |  | X |
| Nelson 2021 | A systematic review exploring pre-COVID-19 telehealthcare models used in the management of patients with rheumatological disease | Autoimmune | Both | X |  | X |
| Newbould 2017 | Videoconferencing for Health Care Provision for Older Adults in Care Homes: A Review of the Research Evidence | Geriatric | Video | X |  | X |
| Nguyen 2022 | Digital Health Technologies for Remote Monitoring and Management of Inflammatory Bowel Disease: A Systematic Review | GI | Telephone | X | X | X |
| Ni 2022 | Effects of eHealth Interventions on Quality of Life and Psychological Outcomes in Cardiac Surgery Patients: Systematic Review and Meta-analysis | Cardiovascular | Telephone |  | X | X |
| Nie 2022 | A Systematic Review of Outpatient Telemedicine Use in Neurosurgery Since the Start of Coronavirus Disease 2019 | Surgery | Both |  |  | X |
| Nieblas 2022 | Impact and future of telemedicine amidst the COVID-19 pandemic: a systematic review of the state-of-the-art in Latin America | Infectious disease | Both |  |  |  |
| Niknejad 2021 | Understanding Telerehabilitation Technology to Evaluate Stakeholders' Adoption of Telerehabilitation Services: A Systematic Literature Review and Directions for Further Research | Infectious disease | Both | X |  | X |
| Ning 2021 | Telemedicine in Otolaryngology: A Systematic Review of Image Quality, Diagnostic Concordance, and Patient and Provider Satisfaction | ENT | Video |  |  | X |
| Nizeyimana 2022 | A scoping review of feasibility, cost, access to rehabilitation services and implementation of telerehabilitation: Implications for low- and middle-income countries | All health conditions | Both | X |  |  |
| Niznik 2018 | Impact of clinical pharmacist services delivered via telemedicine in the outpatient or ambulatory care setting: A systematic review | All health conditions | Both |  | X | X |
| Njoroge 2017 | Assessing the feasibility of eHealth and mHealth: a systematic review and analysis of initiatives implemented in Kenya | Multiple | Multiple | X |  |  |
| Nkodo 2022 | The Role of Telemedicine in the Management of the Behavioral and Psychological Symptoms of Dementia: A Systematic Review | CNS | Both |  |  | X |
| Nordtug 2018 | The use of videoconferencing in nursing for people in their homes | Multiple | Video |  |  |  |
| Nourimand 2022 | A systematic review of eHealth modes in preventing sexually transmitted infections | Infectious disease | Telephone | X | X |  |
| Novara 2020 | Telehealth in Urology: A Systematic Review of the Literature. How Much Can Telemedicine Be Useful During and After the COVID-19 Pandemic? | GU | Both | X | X | X |
| Nutarelli 2021 | Home-Based vs Supervised Inpatient and/or Outpatient Rehabilitation Following Knee Meniscectomy A Systematic Review and Meta-analysis | Surgery | Telerehabilitation |  |  | X |
| Oates 2021 | Are Surgical Patients Satisfied With Remote Consultations? A Comparison of Remote Versus Conventional Outpatient Clinic Follow-Up for Surgical Patients: A Systematic Review and Meta-Analysis of Randomized Controlled Trials | Surgery | Both | X |  | X |
| Obasola 2015 | A Review of e-Health Interventions for Maternal and Child Health in Sub-Sahara Africa | Maternal health | Telephone | X |  |  |
| Obilor 2022 | Use of Information Communication Technology Tools in Diabetic Foot Ulcer Prevention Programs: A Scoping Review | Metabolic | Both | X | X | X |
| O'Brien 2018 | Effectiveness of telephone-based interventions for managing osteoarthritis and spinal pain: a systematic review and meta-analysis | Pain | Video | X | X | X |
| Obro 2021 | Combining mHealth and health-coaching for improving self-management in chronic care. A scoping review | Multiple | Both |  |  |  |
| O'Cathail 2020 | The Use of Patient-Facing Teleconsultations in the National Health Service: Scoping Review | All health conditions | Video | X |  | X |
| O'Connor 2016 | Understanding factors affecting patient and public engagement and recruitment to digital health interventions: A systematic review of qualitative studies | All health conditions | Telephone | X |  | X |
| Odendaal 2020 | Health workers' perceptions and experiences of using mHealth technologies to deliver primary healthcare services: a qualitative evidence synthesis | Multiple | Both |  |  | X |
| Odukoya 2021 | mHealth Interventions for Treatment Adherence and Outcomes of Care for Cardiometabolic Disease Among Adults Living With HIV: Systematic Review | Multiple | Telephone |  | X | X |
| Ohannessian 2020 | Acute telestroke in France: A systematic review | Cardiovascular | Video |  | X | X |
| Ojeahere 2022 | Telehealth interventions for substance use disorders in low- and- middle income countries: A scoping review | Mental health | Both | X | X |  |
| Okuyama 2015 | Psychosocial telephone interventions for patients with cancer and survivors: a systematic review | Cancer | Telephone |  |  | X |
| Oliveira 2022 | Hybrid and Remote Psychosocial Interventions Focused on Weight and Sedentary Behavior Management Among Patients with Severe Mental Illnesses: a Systematic Review | Mental health | Telephone |  | X | X |
| Oliveira 2023 | Internet-delivered cognitive behavioral therapy for anxiety among university students: A systematic review and meta-analysis | Mental health | Both |  |  | X |
| Olthuis 2016 | Therapist-supported Internet cognitive behavioural therapy for anxiety disorders in adults | Mental health | Telephone |  |  | X |
| Olthuis 2016 | Distance-delivered interventions for PTSD: A systematic review and meta-analysis | Mental health | Both |  |  | X |
| O'Neil 2020 | Remote supervision of rehabilitation interventions for survivors of moderate or severe traumatic brain injury: A scoping review | CNS | Both | X | X | X |
| Onwumere 2018 | Digital Technology for Caregivers of People With Psychosis: Systematic Review | Mental health | Both | X | X | X |
| Ooi 2020 | Implementation Strategies for Web-Based Apps for Screening: Scoping Review | All health conditions | Telephone | X |  |  |
| Ora 2022 | Efficacy of respiratory tele-rehabilitation in COPD patients: Systematic review and meta-analysis | Respiratory | Both |  | X | X |
| Orlando 2019 | Systematic review of patient and caregivers' satisfaction with telehealth videoconferencing as a mode of service delivery in managing patients' health | Multiple | Video |  |  | X |
| Orsolini 2021 | A Systematic Review on TeleMental Health in Youth Mental Health: Focus on Anxiety, Depression and Obsessive-Compulsive Disorder | Mental health | Both |  |  |  |
| Orton 2018 | Strengthening Delivery of Health Services Using Digital Devices | All health conditions | Telephone |  |  | X |
| Osborn 2020 | Do mHealth applications improve clinical outcomes of patients with cancer? A critical appraisal of the peer-reviewed literature | Cancer | Telephone | X |  | X |
| Osei 2021 | Mapping evidence of mobile health technologies for disease diagnosis and treatment support by health workers in sub-Saharan Africa: a scoping review | All health conditions | Telephone |  |  |  |
| Ostherr 2016 | Death in the Digital Age: A Systematic Review of Information and Communication Technologies in End-of-Life Care | End of life | Both |  |  |  |
| Oter 2022 | The Effectiveness of Using Telemedicine to Follow-up Breast Cancer During the Covid-19 Pandemic: A Scoping Review | Cancer | Both | X | X | X |
| Ovtcharenko 2019 | Interventions to Improve Clinical Outcomes in Indigenous or Remote Patients With Chronic Kidney Disease: A Scoping Review | GU | Telephone | X | X | X |
| Owen 2022 | The effectiveness of cardiac telerehabilitation in comparison to centre-based cardiac rehabilitation programmes: A literature review | Cardiovascular | Both | X | X | X |
| Ownsworth 2018 | Efficacy of Telerehabilitation for Adults With Traumatic Brain Injury: A Systematic Review | CNS | Both | X | X | X |
| Ownsworth 2020 | Use of telehealth platforms for delivering supportive care to adults with primary brain tumors and their family caregivers: A systematic review | Cancer | Both | X | X | X |
| Owolabi 2022 | Telemedicine in Surgical Care in Low- and Middle-Income Countries: A Scoping Review | Surgery | Both | X |  | X |
| Paalimaki-Paakki 2022 | Effectiveness of Digital Counseling Environments on Anxiety, Depression, and Adherence to Treatment Among Patients Who Are Chronically Ill: Systematic Review | Multiple | Video |  | X | X |
| Padmavathi 2023 | Systematic Review on End-Usersâ€™ Perception of Facilitators and Barriers in Accessing Tele-Rehabilitation Services | Mental health | Video |  |  |  |
| Paganini 2018 | Economic evaluations of internet- and mobile-based interventions for the treatment and prevention of depression: A systematic review | Mental health | Multiple | X |  |  |
| Pala 2020 | Teledermatology: idea, benefits and risks of modern age - a systematic review based on melanoma | Dermatology | Video |  |  |  |
| Palacios 2017 | Internet-Delivered Self-management Support for Improving Coronary Heart Disease and Self-management-Related Outcomes A Systematic Review | Cardiovascular | Telephone |  | X | X |
| Palfreman 2023 | The use of telemedicine in forensic psychiatry-a quick scoping review of literature from the time of the COVID-19 pandemic | Mental health | Video | X |  | X |
| Palmer 2018 | The effectiveness of smoking cessation, physical activity/diet and alcohol reduction interventions delivered by mobile phones for the prevention of non-communicable diseases: A systematic review of randomised controlled trials | Multiple | Telephone |  | X | X |
| Palmer 2018 | Mobile phone-based interventions for improving adherence to medication prescribed for the primary prevention of cardiovascular disease in adults | Cardiovascular | Both |  | X | X |
| Palmer 2021 | Mobile phone-based interventions for improving adherence to medication prescribed for the primary prevention of cardiovascular disease in adults | Cardiovascular | Both | X | X | X |
| Palozzi 2020 | Enhancing the Sustainable Goal of Access to Healthcare: Findings from a Literature Review on Telemedicine Employment in Rural Areas | Multiple | Both |  |  | X |
| Pang 2022 | Role of Telemedicine in Inflammatory Bowel Disease: Systematic Review and Meta-analysis of Randomized Controlled Trials | GI | Both |  | X | X |
| Pang 2022 | Telemedicine Acceptance among Older Adult Patients with Cancer: Scoping Review | Cancer | Both | X |  |  |
| Park 2016 | Mobile Phone Interventions for the Secondary Prevention of Cardiovascular Disease | Cardiovascular | Both |  | X | X |
| Park 2022 | Digital Health Interventions by Clinical Pharmacists: A Systematic Review | Not specified | Both |  | X | X |
| Park 2022 | Economic Evaluation of Pharmacist-Led Digital Health Interventions: A Systematic Review | Not specified | Both | X |  |  |
| Park 2022 | Content and Effectiveness of Web-Based Treatments for Online Behavioral Addictions: Systematic Review | Mental health | Video |  |  |  |
| Parker 2018 | Electronic, mobile and telehealth tools for vulnerable patients with chronic disease: A systematic review and realist synthesis | Multiple | Telephone |  | X | X |
| Parkes 2022 | Telemedicine interventions in six conflict-affected countries in the WHO Eastern Mediterranean region: a systematic review | Multiple | Both | X | X | X |
| Parmar 2015 | Use of Telemedicine Technologies in the Management of Infectious Diseases: A Review | Infectious disease | Both |  |  |  |
| Pastora-Bernal 2017 | Evidence of benefit of telerehabitation after orthopedic surgery: A systematic review | Surgery | Both |  |  | X |
| Patel 2020 | The Acceptability and Usability of Digital Health Interventions for Adults With Depression, Anxiety, and Somatoform Disorders: Qualitative Systematic Review and Meta-Synthesis | Mental health | Video | X |  | X |
| Patel 2022 | Public Health Implications of Adapting HIV Pre-exposure Prophylaxis Programs for Virtual Service Delivery in the Context of the COVID-19 Pandemic: Systematic Review | Infectious disease | Video | X | X |  |
| Pathak 2021 | A Systematic Review of the Effect of Telepharmacy Services in the Community Pharmacy Setting on Care Quality and Patient Safety | All health conditions | Video |  | X | X |
| Patil 2021 | Specialist to non-specialist teleconsultations in chronic respiratory disease management: A systematic review | Respiratory | Both | X | X | X |
| Patra 2022 | A Scoping Review of Changes to Patient-Doctor Communication During COVID-19 | All health conditions | Both |  |  |  |
| Patterson 2022 | Virtual care and the influence of a pandemic: Necessary policy shifts to drive digital innovation in healthcare | All health conditions | Both |  |  |  |
| Pengput 2022 | Telemedicine in Southeast Asia: A Systematic Review | All health conditions | Both |  |  |  |
| Penny 2018 | Registered nurse and midwife experiences of using videoconferencing in practice: A systematic review of qualitative studies | Not specified | Video |  |  | X |
| Peretz 2018 | Determining the cost of implementing and operating a remote patient monitoring programme for the elderly with chronic conditions: A systematic review of economic evaluations | Multiple | Telephone | X |  |  |
| Perisetti 2021 | Successful Distancing: Telemedicine in Gastroenterology and Hepatology During the COVID-19 Pandemic | GI | Both |  |  |  |
| Peters 2021 | The Effect of Telehealth on Hospital Services Use: Systematic Review and Meta-analysis | Multiple | Both |  |  | X |
| Petersen 2021 | A systematic review about telemedicine in orthopedics | Musckuloskeletal | Video | X |  | X |
| Pfennig 2022 | How does telementoring impact medical education within the surgical field? A scoping review | Surgery | Video |  | X | X |
| Pham 2020 | Virtual care models for cancer survivorship | Cancer | Both | X | X | X |
| Piche 2021 | Physical Examination of the Spine Using Telemedicine: A Systematic Review | Musckuloskeletal | Both |  |  | X |
| PickardStrange 2023 | The Role of Virtual Consulting in Developing Environmentally Sustainable Health Care: Systematic Literature Review | All health conditions | Both | X |  |  |
| Piga 2017 | Telemedicine for patients with rheumatic diseases: Systematic review and proposal for research agenda | Autoimmune | Both | X |  | X |
| Piskulic 2021 | Virtual visits in cardiovascular disease: a rapid review of the evidence | Cardiovascular | Both |  |  | X |
| Platini 2022 | Systematic Review and Meta-Analysis of Telecoaching for Self-Care Management among Persons with Type 2 Diabetes Mellitus | Metabolic | Telephone | X |  | X |
| Platz 2023 | Benefits, Facilitators, and Barriers of Alternative Models of Cardiac Rehabilitation: A QUALITATIVE SYSTEMATIC REVIEW | Cardiovascular | Video |  |  | X |
| Ploeg 2017 | Web-Based Interventions to Improve Mental Health, General Caregiving Outcomes, and General Health for Informal Caregivers of Adults With Chronic Conditions Living in the Community: Rapid Evidence Review | Multiple | Both |  |  | X |
| Pogorzelska 2022 | Patient Satisfaction with Telemedicine during the COVID-19 Pandemic-A Systematic Review | Multiple | Both |  |  | X |
| Poletti 2021 | Telepsychotherapy: a leaflet for psychotherapists in the age of COVID-19. A review of the evidence | Mental health | Video |  |  | X |
| Popovic 2022 | Online Psychological intervention in Breast Cancer Survivors: a Review | Cancer | Internet-based NOS |  |  | X |
| Pouls 2021 | Effect of interactive ehealth interventions on improving medication adherence in adults with long-term medication: Systematic review | Multiple | Telephone |  | X |  |
| Pugliese 2018 | Mobile tablet-based therapies following stroke: A systematic scoping review of administrative methods and patient experiences | Cardiovascular | Video |  | X | X |
| Purnomo 2018 | Using eHealth to engage and retain priority populations in the HIV treatment and care cascade in the Asia-Pacific region: a systematic review of literature | Multiple | Telephone |  | X | X |
| Purohit 2021 | Does telemedicine reduce the carbon footprint of healthcare? A systematic review | All health conditions | Both | X |  |  |
| Qaderi 2023 | Abortion services during the COVID-19 pandemic: a systematic review | Maternal health | Telephone |  |  | X |
| Qan'ir 2019 | Systematic review of technology-based interventions to improve anxiety, depression, and health-related quality of life among patients with prostate cancer | Cancer | Both |  |  | X |
| Qian 2019 | Telehealth Interventions for Improving Self-Management in Patients With Hemophilia: Scoping Review of Clinical Studies | Cardiovascular | Telephone | X | X | X |
| Quilty 2021 | Benefits of Digital Health Resources for Substance Use Concerns in Women: Scoping Review | Mental health | Both |  | X |  |
| Raaijmakers 2015 | Technology-based interventions in the treatment of overweight and obesity: A systematic review | Metabolic | Telephone | X | X | X |
| Racey 2023 | Technology-Supported Integrated Care Innovations to Support Diabetes and Mental Health Care: Scoping Review | Metabolic | Telephone |  | X | X |
| Radhakrishnan 2016 | Barriers and facilitators for sustainability of tele-homecare programs: A systematic review | Multiple | Both |  |  | X |
| Raja 2021 | Telehealth and digital developments in society that persons 75 years and older in European countries have been part of: a scoping review | Geriatric | Both |  |  |  |
| Rajkumar 2023 | Applications, benefits and challenges of telehealth in India during COVID-19 pandemic and beyond: a systematic review | All health conditions | Both | X |  | X |
| Ramachandran 2022 | Technology Acceptance of Home-Based Cardiac Telerehabilitation Programs in Patients With Coronary Heart Disease: Systematic Scoping Review | Cardiovascular | Both | X |  | X |
| Ramachandran 2022 | Effectiveness of home-based cardiac telerehabilitation as an alternative to Phase 2 cardiac rehabilitation of coronary heart disease: A systematic review and meta-analysis | Cardiovascular | Both |  | X | X |
| Ramachandran 2023 | Barriers and facilitators to the adoption of digital health interventions for COPD management: A scoping review | Respiratory | Both |  |  |  |
| Ramage 2021 | Look Before You Leap: Interventions Supervised via Telehealth Involving Activities in Weight-Bearing or Standing Positions for People After Stroke - A Scoping Review | Cardiovascular | Both | X |  | X |
| Randine 2022 | Information and communication technology-based interventions for chronic diseases consultation: Scoping review | Multiple | Video |  |  | X |
| Raphael 2017 | Telephone communication between practice nurses and older patients with long term conditions - a systematic review | Multiple | Telephone |  | X | X |
| Rasouli 2020 | Overview of telemedicine use aspects in natural and technical disaster and combat situation | Multiple | Both | X |  | X |
| Ratnasari 2022 | Safety of Pregnant Women during the Covid-19 Pandemic by Using the Telehealth Application: A Systematic Review | Maternal health | Both |  | X |  |
| Rawal 2021 | Will Remotely Based Pulmonary Rehabilitation Water Down Its Effectiveness? | Respiratory | Both | X | X | X |
| Rawstorn 2016 | Telehealth exercise-based cardiac rehabilitation: A systematic review and meta-analysis | Cardiovascular | Both |  | X | X |
| Raynor 2023 | Leveraging Digital Technology to Support Pregnant and Early Parenting Women in Recovery from Addictive Substances: A Scoping Review | Maternal health | Telephone | X | X |  |
| Ream 2020 | Telephone interventions for symptom management in adults with cancer | Cancer | Both |  |  | X |
| Reangsing 2022 | Effects of Online Mindfulness-Based Interventions on Depressive Symptoms in College and University Students: A Systematic Review and Meta-Analysis | Mental health | Video |  |  | X |
| RebolledoDelToro 2023 | Effectiveness of mobile telemonitoring applications in heart failure patients: systematic review of literature and meta-analysis | Cardiovascular | Telephone |  |  | X |
| Rees 2015 | A Systematic Review of Videoconference-Delivered Psychological Treatment for Anxiety Disorders | Mental health | Video |  |  | X |
| Renner 2021 | Barriers to Accessing Health Care in Rural Regions by Transgender, Non-Binary, and Gender Diverse People: A Case-Based Scoping Review | All health conditions | Video | X |  |  |
| Reverberi 2022 | The neurogenic dysphagia management via telemedicine: a systematic review | CNS | Video |  |  | X |
| Reychler 2022 | Telerehabilitation as a Form of Pulmonary Rehabilitation in Chronic Lung Disease: A Systematic Review | Respiratory | Both | X | X | X |
| Rezapour 2021 | Economic evaluation of E-health interventions compared with alternative treatments in older persons' care: A systematic review | Geriatric | Video | X |  | X |
| Rezende 2023 | Telehealth and telemedicine in the management of adult patients after hospitalization for COPD exacerbation: a scoping review | Respiratory | Both |  |  |  |
| Riadi 2022 | Digital interventions for depression and anxiety in older adults: a systematic review of randomised controlled trials | Mental health | Both |  |  | X |
| Ridho 2022 | Digital Health Technologies to Improve Medication Adherence and Treatment Outcomes in Patients With Tuberculosis: Systematic Review of Randomized Controlled Trials | Infectious disease | Both |  | X | X |
| Riley 2022 | Accuracy of telephone screening tools to identify dementia patients remotely: systematic review | CNS | Telephone | X |  |  |
| Rinn 2023 | Digital Interventions for Treating Post-COVID or Long-COVID Symptoms: Scoping Review | Infectious disease | Both |  |  | X |
| Robson 2021 | Impact of Telehealth Care among Adults Living with Type 2 Diabetes in Primary Care: A Systematic Review and Meta-Analysis of Randomised Controlled Trials | Metabolic | Both |  |  | X |
| Rocco 2022 | Some key aspects of telemedicine development to support human illness: a systematic review | All health conditions | Both |  |  |  |
| Rodgers 2022 | An integrative review of the person-centred and experiential therapy literature on delivering individual video counselling and psychotherapy | Mental health | Video |  |  | X |
| Rohde 2019 | Impact of eHealth technologies on patient outcomes: a meta-analysis of chronic gastrointestinal illness interventions | GI | Internet-based NOS |  | X | X |
| Rohrbach 2023 | Cost-effectiveness of Internet Interventions Compared With Treatment as Usual for People With Mental Disorders: Systematic Review and Meta-analysis of Randomized Controlled Trials | Mental health | Telephone | X |  |  |
| Rootes-Murdy 2018 | Mobile technology for medication adherence in people with mood disorders: A systematic review | Mental health | Telephone | X | X | X |
| Rosenlund 2023 | The Use of Digital Health Services Among Patients and Citizens Living at Home: Scoping Review | All health conditions | Both |  |  | X |
| Ruggiano 2018 | Rural Dementia Caregivers and Technology: What Is the Evidence? | CNS | Both |  | X | X |
| Ruiz-Cosignani 2022 | Adaptation models, barriers, and facilitators for cultural safety in telepsychiatry: A systematic scoping review | Mental health | Both |  | X |  |
| Rush 2018 | Videoconference compared to telephone in healthcare delivery: A systematic review | All health conditions | Both | X | X | X |
| Rush 2022 | Telehealth Use for Enhancing the Health of Rural Older Adults: A Systematic Mixed Studies Review | Geriatric | Both |  | X | X |
| Ruyobeza 2022 | Hurdles to developing and scaling remote patients' health management tools and systems: a scoping review | Multiple | Both |  |  |  |
| RysstGustafsson 2021 | Quality indicators in telephone nursing - An integrative review | Multiple | Telephone | X |  | X |
| SÃ¶ylemez 2023 | Telehealth applications used for self-efficacy levels of family caregivers for individuals with dementia: A systematic review and Meta-analysis | CNS | Telephone |  | X | X |
| Saad 2021 | Self-directed Technology-Based Therapeutic Methods for Adult Patients Receiving Mental Health Services: Systematic Review | Mental health | Both | X |  | X |
| Sabahi 2021 | Telemedicine Services in Chronic Obstructive Pulmonary Disease: A Systematic Review of Patients' Adherence | Respiratory | Both | X | X |  |
| Safari 2020 | Digital self-management interventions for people with osteoarthritis: Systematic review with meta-analysis | Musckuloskeletal | Both | X | X | X |
| Safdari 2021 | Telehealth and Telemedicine in Response to Critical Coronavirus: A Systematic Review | Infectious disease | Video |  |  |  |
| Sagaro 2020 | Barriers to Sustainable Telemedicine Implementation in Ethiopia: A Systematic Review | Not specified | Telephone |  |  |  |
| Sagoe 2022 | Internet-based treatment of gambling problems: A systematic review and meta-analysis of randomized controlled trials | Mental health | Internet-based NOS |  |  | X |
| Sahin 2017 | Mixed-methods research in diabetes management via mobile health technologies: A scoping review | Metabolic | Telephone | X |  | X |
| Sahin 2021 | Telemedicine interventions for older adults: A systematic review | All health conditions | Video | X |  | X |
| Sakunrag 2020 | Clinical Outcomes of Telephone Service for Patients on Warfarin: A Systematic Review and Meta-Analysis | Multiple | Telephone |  |  | X |
| Salisbury 2020 | The impact of digital-first consultations on workload in general practice: Modeling study | Multiple | Both |  | X |  |
| Salmoiraghi 2015 | A Systematic Review of the Use of Telepsychiatry in Acute Settings | Mental health | Both | X | X | X |
| Salsabilla 2021 | Cost-Effectiveness of Telemedicine in Asia: A Scoping Review | Multiple | Both | X | X |  |
| Salvemini 2019 | Insomnia and Information and Communication Technologies (ICT) in Elderly People: A Systematic Review | CNS | Video |  |  | X |
| Sanchez-Gutierrez 2022 | Effectiveness of telemedicine psychoeducational interventions for adults with non-oncological chronic disease: A systematic review | Multiple | Both |  |  | X |
| Sanchez-Ramirez 2022 | Effect of telemonitoring and telerehabilitation on physical activity, exercise capacity, health-related quality of life and healthcare use in patients with chronic lung diseases or COVID-19: A scoping review | Respiratory | Both |  | X | X |
| Sandberg 2019 | Using Telemedicine to Diagnose Surgical Site Infections in Low- and Middle-Income Countries: Systematic Review | Surgery | Telephone | X |  | X |
| Santesteban-Echarri 2020 | Telehealth interventions for schizophrenia-spectrum disorders and clinical high-risk for psychosis individuals: A scoping review | Mental health | Video | X |  | X |
| SantiagodeAraujoPio 2019 | Interventions to promote patient utilisation of cardiac rehabilitation | Cardiovascular | Both | X |  | X |
| Santos 2022 | The effectiveness of the use of telehealth programs in the care of individuals with hypertension and, or diabetes mellitus: systematic review and meta-analysis | Metabolic | Video | X |  | X |
| Sanyal 2018 | Economic evaluations of eHealth technologies: A systematic review | All health conditions | Telephone | X |  |  |
| Saragih 2021 | Effects of telehealth interventions on performing activities of daily living and maintaining balance in stroke survivors: A systematic review and meta-analysis of randomised controlled studies | Cardiovascular | Both |  |  | X |
| Saragih 2021 | Effects of telehealth-assisted interventions among people living with HIV/AIDS: A systematic review and meta-analysis of randomized controlled studies | Infectious disease | Both |  | X | X |
| Sartori 2022 | Telemedicine in surgery during COVID-19 pandemic: are we doing enough? | Surgery | Telephone |  | X | X |
| Savira 2023 | Virtual Care Initiatives for Older Adults in Australia: Scoping Review | All health conditions | Both | X | X | X |
| Saylik 2023 | Digital Health Interventions in Patient Management following Acute Coronary Syndrome: A Meta-Analysis of the Literature | Cardiovascular | Both |  | X | X |
| Scherrenberg 2020 | Cost-effectiveness of cardiac telerehabilitation in coronary artery disease and heart failure patients: systematic review of randomized controlled trials | Cardiovascular | Both | X |  |  |
| Schiller 2023 | Efficacy of Virtual Care for Depressive Disorders: Systematic Review and Meta-analysis | Mental health | Internet-based NOS |  |  | X |
| Schlief 2022 | Synthesis of the Evidence on What Works for Whom in Telemental Health: Rapid Realist Review | Mental health | Both |  |  |  |
| Schubert 2023 | Impact of telehealth on the current and future practice of lipidology: a scoping review | Metabolic | Both |  |  | X |
| Schulte 2021 | Effectiveness of eHealth Interventions in Improving Medication Adherence for Patients With Chronic Obstructive Pulmonary Disease or Asthma: Systematic Review | Respiratory | Both |  | X |  |
| Scott 2022 | Real-Time Telehealth Versus Face-to-Face Management for Patients With PTSD in Primary Care: A Systematic Review and Meta-Analysis | Mental health | Both |  | X | X |
| Scott 2022 | Telehealth v. face-to-face provision of care to patients with depression: a systematic review and meta-analysis | Mental health | Both |  | X | X |
| Sekhon 2021 | Telemedicine and the rural dementia population: A systematic review | CNS | Both |  | X | X |
| Sekhon 2021 | Effectiveness of web-based and mobile health interventions designed to enhance adherence to physical activity for people with inflammatory arthritis: a systematic review | Musckuloskeletal | Internet-based NOS |  | X |  |
| Selick 2021 | Virtual health care for adult patients with intellectual and developmental disabilities: A scoping review | Multiple | Both | X | X |  |
| Semi 2022 | Effect of Follow-up Telephone by Enterostomal Nurses on Patients with Permanent Colostomy: A Systematic Review | Cancer | Telephone |  | X | X |
| Senanayake 2018 | Telemedicine in the correctional setting: A scoping review | Multiple | Video | X | X |  |
| Sequi-Dominguez 2020 | Effectiveness of mobile health interventions promoting physical activity and lifestyle interventions to reduce cardiovascular risk among individuals with metabolic syndrome: Systematic review and meta-analysis | Metabolic | Telephone |  |  | X |
| Sexton 2022 | Service use, clinical outcomes and user experience associated with urgent care services that use telephone-based digital triage: A systematic review | Acute/ urgent care | Telephone |  | X | X |
| ShafieeHanjani 2020 | A scoping review of the use and impact of telehealth medication reviews | Multiple | Video | X | X | X |
| Shah 2019 | A systematic review and meta-analysis in the effectiveness of mobile phone interventions used to improve adherence to antiretroviral therapy in HIV infection | Infectious disease | Telephone |  | X |  |
| Shah 2022 | Digital Health Interventions for Depression and Anxiety Among People With Chronic Conditions: Scoping Review | Mental health | Multiple | X |  |  |
| Shah 2023 | Diagnostic accuracy and management concordance of otorhinolaryngological diseases through telehealth or remote visits: A systematic review & meta-analysis | ENT | Both | X |  |  |
| Shahouzaie 2022 | Telehealth in speech and language therapy during the COVID-19 pandemic: a systematic review | CNS | Both |  |  |  |
| Shanbehzadeh 2021 | Systematic review on telemedicine platforms in lockdown periods: Lessons learned from the COVID-19 pandemic | Infectious disease | Both |  |  |  |
| Sharafkhaneh 2022 | Telemedicine and insomnia: a comprehensive systematic review and meta-analysis | CNS | Both |  |  | X |
| Sharififar 2023 | Telerehabilitation service impact on physical function and adherence compared to face-to-face rehabilitation in patients with stroke: A Systematic Review and Meta-Analysis | Cardiovascular | Video | X |  | X |
| Sharpe 2017 | Examining Factors of Engagement With Digital Interventions for Weight Management: Rapid Review | Metabolic | Telephone | X |  |  |
| Shek 2021 | Technology-based interventions for mental health support after stroke: A systematic review of their acceptability and feasibility | Cardiovascular | Both | X |  |  |
| Shen 2019 | Electronic health self-management interventions for patients with chronic kidney disease: Systematic review of quantitative and qualitative evidence | GU | Video | X | X | X |
| Shih 2023 | The effects of treatment via telemedicine interventions for patients with depression on depressive symptoms and quality of life: a systematic review and meta-ranalysis | Mental health | Telephone |  |  | X |
| Shin 2017 | Mobile Phone Interventions for Sleep Disorders and Sleep Quality: Systematic Review | CNS | Telephone |  |  | X |
| Shrivastava 2023 | Mobile App Interventions to Improve Medication Adherence Among Type 2 Diabetes Mellitus Patients: A Systematic Review of Clinical Trials | Metabolic | Mobile App |  | X | X |
| Shukla 2017 | Role of telerehabilitation in patients following total knee arthroplasty: Evidence from a systematic literature review and meta-analysis | Musckuloskeletal | Both |  |  | X |
| Shunsuke 2021 | Telerehabilitation in Subjects With Respiratory Disease: A Scoping Review | Respiratory | Video | X | X |  |
| Siddique 2021 | Interventions to Reduce Hospital Length of Stay in High-risk Populations: A Systematic Review | Multiple | Telemedicine NOS |  |  | X |
| Siegel 2021 | Barriers, benefits and interventions for improving the delivery of telemental health services during the coronavirus disease 2019 pandemic: a systematic review | Mental health | Both |  |  |  |
| Silang 2021 | eHealth Interventions to Treat Substance Use in Pregnancy: A Systematic Review and Meta-Analysis | Mental health | Telephone |  | X | X |
| Silva 2021 | COVID-19 Remote Consultation Services and Population in Health Inequity-Concentrating Territories: A Scoping Review | Multiple | Both |  |  | X |
| Silva 2022 | Digital Health Opportunities to Improve Primary Health Care in the Context of COVID-19: Scoping Review | Multiple | Both |  |  |  |
| Simon 2021 | Internet-based cognitive and behavioural therapies for post-traumatic stress disorder (PTSD) in adults | Mental health | Both |  | X | X |
| Sin 2018 | eHealth interventions for family carers of people with long term illness: A promising approach? | Multiple | Both | X | X | X |
| Sin 2020 | Digital Interventions for Screening and Treating Common Mental Disorders or Symptoms of Common Mental Illness in Adults: Systematic Review and Meta-analysis | Mental health | Internet-based NOS |  | X | X |
| Sinabell 2022 | Challenges and recommendations for eHealth usability evaluation with elderly users: systematic review and case study | Multiple | Virtual NOS |  |  |  |
| Singh 2016 | Role of telehealth in diabetic foot ulcer management - A systematic review | Metabolic | Both | X |  | X |
| Singh 2021 | A Review of Telemedicine Applications in Otorhinolaryngology: Considerations During the Coronavirus Disease of 2019 Pandemic | ENT | Both | X |  | X |
| Slattery 2019 | An evaluation of the effectiveness of the modalities used to deliver electronic health interventions for chronic pain: Systematic review with network meta-analysis | Pain | Both |  |  | X |
| Smith 2022 | Remote Follow-Up Technologies in Traumatic Brain Injury: A Scoping Review | CNS | Both |  |  | X |
| Snoswell 2020 | Determining if Telehealth Can Reduce Health System Costs: Scoping Review | All health conditions | Both | X |  |  |
| Snoswell 2021 | A Systematic Review and Meta-Analysis of Change in Health-Related Quality of Life for Interactive Telehealth Interventions for Patients With Asthma | Respiratory | Video |  | X | X |
| So 2018 | Telehealth for diabetes self-management in primary healthcare: A systematic review and meta-analysis | Metabolic | Telephone |  |  | X |
| Sogomonjan 2018 | A Review Article on Internet-based Psychological Interventions in Primary Care. What Is the Global Experience? How Reliable Are Results from RCTs? Lessons Learned from the European, US and Australian Case Studies | Mental health | Telephone | X |  | X |
| Soh 2023 | The effectiveness of tele-transitions of care interventions in high-risk older adults: A systematic review and meta-analysis | Geriatric | Both |  |  | X |
| Solomon 2022 | Telerehabilitation for individuals with spinal cord injury in low-and middle-income countries: a systematic review of the literature | Musckuloskeletal | Both |  |  | X |
| Son 2020 | Effectiveness of Mobile Phone-Based Interventions for Improving Health Outcomes in Patients with Chronic Heart Failure: A Systematic Review and Meta-Analysis | Cardiovascular | Telephone |  | X | X |
| Sondaal 2016 | Assessing the effect of mHealth interventions in improving maternal and neonatal care in low- And middle-income countries: A systematic review | Maternal health | Telephone |  | X |  |
| Sondergaard 2022 | How patients with diabetic foot ulcers experience telemedicine solutions: A scoping review | Metabolic | Video |  |  | X |
| Song 2022 | Effects of home-based telehealth on the physical condition and psychological status of patients with chronic obstructive pulmonary disease: A systematic review and meta-analysis | Respiratory | Both |  |  | X |
| Song 2022 | Randomized Controlled Trials of Digital Mental Health Interventions on Patients with Schizophrenia Spectrum Disorder: A Systematic Review | Mental health | Multiple |  | X | X |
| Sourtiji 2023 | Telerehabilitation in People with Multiple Sclerosis: A Scoping Review | CNS | Both |  |  | X |
| Souza 2016 | Application of telenursing in nursing practice: an integrative literature review | All health conditions | Both |  |  |  |
| Spelten 2021 | Best practice in the implementation of telehealth-based supportive cancer care: Using research evidence and discipline-based guidance | Cancer | Both |  |  |  |
| Spencer 2016 | A systematic review of Motivational Interviewing interventions in cancer patients and survivors | Cancer | Telephone | X | X | X |
| Spencer 2020 | Telemedicine in the Management of ADHD: Literature Review of Telemedicine in ADHD | CNS | Video | X |  | X |
| Speyer 2018 | Effects of telehealth by allied health professionals and nurses in rural and remote areas: A systematic review and meta-analysis | All health conditions | Both |  |  |  |
| Spijkerman 2016 | Effectiveness of online mindfulness-based interventions in improving mental health: A review and meta-analysis of randomised controlled trials | Mental health | Multiple |  |  | X |
| Spina 2022 | Telemedicine application to headache: a critical review | CNS | Video | X | X | X |
| Stavropoulos 2022 | A scoping review of telehealth diagnosis of autism spectrum disorder | CNS | Video | X |  |  |
| Steindal 2020 | Patients' Experiences of Telehealth in Palliative Home Care: Scoping Review | End of life | Both |  |  | X |
| Steindal 2023 | Advantages and Challenges of Using Telehealth for Home-Based Palliative Care: Systematic Mixed Studies Review | End of life | Both |  |  |  |
| Stellefson 2022 | COPD Self-Management for Adults Living in Rural Areas: Systematic Review of Telehealth and Non-Telehealth Interventions | Respiratory | Both |  | X | X |
| Stephani 2016 | A systematic review of randomized controlled trials of mHealth interventions against non-communicable diseases in developing countries | Multiple | Telephone |  | X | X |
| Stephenson 2022 | Factors influencing the delivery of telerehabilitation for stroke: A systematic review | Cardiovascular | Both | X | X | X |
| Stevens 2019 | EHealth apps replacing or complementing health care contacts: Scoping review on adverse effects | All health conditions | Both |  |  |  |
| Stokes 2022 | Use of technology to prevent, detect, manage and control hypertension in sub-Saharan Africa: a systematic review | Cardiovascular | Telephone |  | X | X |
| Strnad 2018 | A Systematic Review of ICU and Non-ICU Clinical Pharmacy Services Using Telepharmacy | Multiple | Both | X | X | X |
| Su 2020 | Effect of eHealth cardiac rehabilitation on health outcomes of coronary heart disease patients: A systematic review and meta-analysis | Cardiovascular | Mobile App |  | X | X |
| Suh 2017 | Effects of Nurse-Led Telephone-Based Supportive Interventions for Patients With Cancer: A Meta-Analysis | Cancer | Telephone |  |  | X |
| Suleman 2022 | The Use of Virtual Care in Patients with Hematologic Malignancies: A Scoping Review | Cancer | Both |  | X | X |
| Sulz 2021 | eHealth Applications to Support Independent Living of Older Persons: Scoping Review of Costs and Benefits Identified in Economic Evaluations | All health conditions | Both | X | X |  |
| Sunjaya 2020 | Efficacy, patient-doctor relationship, costs and benefits of utilizing telepsychiatry for the management of post-traumatic stress disorder (PTSD): a systematic review | Mental health | Video |  |  | X |
| Sunner 2022 | Does telehealth influence the decision to transfer residents of residential aged care facilities to emergency departments? A scoping review | Multiple | Both | X | X | X |
| Sze 2022 | A systematic review of randomised controlled trials of the effects of digital health interventions on postpartum contraception use | Maternal health | Both | X | X | X |
| Tahan 2023 | Effect of digital health, biomarker feedback and nurse or midwife-led counselling interventions to assist pregnant smokers quit: A systematic review and meta-analysis | Maternal health | Telephone |  | X | X |
| Tahir 2022 | A review of teleradiology in Africa - Towards mobile teleradiology in Nigeria | Radiology | Telemedicine NOS |  |  |  |
| Tan 2015 | Effectiveness of nurse leader rounding and post-discharge telephone calls in patient satisfaction: A systematic review | Acute/ urgent care | Telephone |  |  | X |
| Tan 2017 | Real-time teleophthalmology versus face-to-face consultation: A systematic review | CNS | Both | X |  |  |
| Tan 2021 | Telestroke for acute ischaemic stroke: A systematic review of economic evaluations and a de novo cost-utility analysis for a middle income country | Cardiovascular | Telemedicine NOS | X |  |  |
| Tang 2020 | eHealth Interventions for Solid Organ Transplant Recipients: A Systematic Review and Meta-analysis of Randomized Controlled Trials | Surgery | Both |  | X | X |
| Tao 2018 | Teleaudiology Services for Rehabilitation With Hearing Aids in Adults: A Systematic Review | ENT | Video |  |  |  |
| Tariq 2021 | Covid-19 compels medical practitioners and governments to promote telemedicine practices - a systematic review | Infectious disease | Both |  |  |  |
| Taylor 2020 | The Role of e-Health in the Delivery of Care for Patients with Hematological Cancers: A Systematic Literature Review | Cancer | Telephone | X | X | X |
| Tchero 2017 | Telemedicine in Diabetic Foot Care: A Systematic Literature Review of Interventions and Meta-analysis of Controlled Trials | Metabolic | Both |  |  | X |
| Tchero 2018 | Telerehabilitation for stroke survivors: Systematic review and meta-analysis | Cardiovascular | Both | X |  | X |
| Tchero 2019 | Clinical Effectiveness of Telemedicine in Diabetes Mellitus: A Meta-Analysis of 42 Randomized Controlled Trials | Metabolic | Both | X |  | X |
| Tebeje 2021 | Applications of e-Health to Support Person-Centered Health Care at the Time of COVID-19 Pandemic | Infectious disease | Both | X |  |  |
| Teck 2023 | Key implementation factors in telemedicine-delivered medications for opioid use disorder: a scoping review informed by normalisation process theory | Mental health | Both |  |  |  |
| Thiyagarajan 2020 | Exploring patients' and clinicians' experiences of video consultations in primary care: a systematic scoping review | Multiple | Video |  |  | X |
| Thomas 2021 | Review of the current empirical literature on using videoconferencing to deliver individual psychotherapies to adults with mental health problems | Mental health | Video | X | X | X |
| ThomasCraig 2020 | Rapid review: Identification of digital health interventions in atherosclerotic-related cardiovascular disease populations to address racial, ethnic, and socioeconomic health disparities | Cardiovascular | Both |  | X | X |
| Thompson 2020 | Telemedicine for Family Planning: A Scoping Review | Maternal health | Both | X | X |  |
| Thwaites 2023 | Is telehealth an effective and feasible option for improving falls-related outcomes in community-dwelling adults with neurological conditions? A systematic review and meta-analysis | CNS | Video | X | X | X |
| Tian 2021 | The impacts of and outcomes from telehealth delivered in prisons: A systematic review | Multiple | Video | X | X | X |
| Ting 2021 | Telemedicine for Patient Management on Expeditions in Remote and Austere Environments: A Systematic Review | Multiple | Video |  |  |  |
| Tomlinson 2018 | Training Individuals to Implement Applied Behavior Analytic Procedures via Telehealth: A Systematic Review of the Literature | Not specified | Both | X | X | X |
| Toresdahl 2021 | A Systematic Review of Telehealth and Sport-Related Concussion: Baseline Testing, Diagnosis, and Management | CNS | Video | X |  | X |
| Totten 2022 | Telehealth-guided provider-to-provider communication to improve rural health: A systematic review | All health conditions | Both |  | X | X |
| Trettel 2018 | Telemedicine in dermatology: findings and experiences worldwide - a systematic literature review | Dermatology | Video | X |  | X |
| Triberti 2019 | eHealth for improving quality of life in breast cancer patients: A systematic review | Cancer | Telephone |  |  | X |
| Tripepi 2023 | Telemedicine and Pancreatic Cancer: A Systematic Review | Cancer | Both |  | X | X |
| Truong 2022 | Using telehealth consultations for healthcare provision to patients from non-Indigenous racial/ethnic minorities: a systematic review | All health conditions | Both | X |  | X |
| Tsang 2022 | The effectiveness of telerehabilitation in patients after total knee replacement: A systematic review and meta-analysis of randomized controlled trials | Musckuloskeletal | Both |  | X | X |
| Tse 2018 | Telemonitoring and hemodynamic monitoring to reduce hospitalization rates in heart failure: A systematic review and meta-analysis of randomized controlled trials and real-world studies | Cardiovascular | Telemonitoring |  |  | X |
| Tumma 2022 | Considerations for the Implementation of a Telestroke Network: A Systematic Review | Cardiovascular | Both |  |  | X |
| Turner 2023 | Telehealth interventions for physical activity and exercise participation in postpartum women: A quantitative systematic review | Maternal health | Both |  |  | X |
| Tzelepis 2019 | Real-time video counselling for smoking cessation | Mental health | Video | X | X | X |
| Udsen 2022 | The Effectiveness of Telemedicine Solutions in Type 1 Diabetes Management: A Systematic Review and Meta-analysis | Metabolic | Telephone |  | X | X |
| Uemoto 2022 | Efficacy of Telemedicine Using Videoconferencing Systems in Outpatient Care for Patients With Cancer: A Systematic Review and Meta-Analysis | Cancer | Video | X | X | X |
| Umeh 2021 | Home telemonitoring in heart failure patients and the effect of study design on outcome: A literature review | Cardiovascular | Telemonitoring |  |  | X |
| Vaezipour 2019 | Acceptance of Rehabilitation Technology in Adults with Moderate to Severe Traumatic Brain Injury, Their Caregivers, and Healthcare Professionals: A Systematic Review | CNS | Both | X |  |  |
| Vaikuntharajan 2021 | Telephone-delivered physiotherapy interventions improve physical function for adults with a chronic condition: a systematic review and meta-analysis | Multiple | Telephone |  |  | X |
| VailatiRiboni 2020 | Technologically-enhanced psychological interventions for older adults: A scoping review | Mental health | Multiple | X |  | X |
| Valentijn 2022 | Digital Health Interventions for Musculoskeletal Pain Conditions: Systematic Review and Meta-analysis of Randomized Controlled Trials | Musckuloskeletal | Multiple |  | X | X |
| Valk-Draad 2022 | Nursing Home-Sensitive Hospitalizations and the Relevance of Telemedicine: A Scoping Review | All health conditions | Both | X | X | X |
| Valverde-Martinez 2023 | Telerehabilitation, A Viable Option in Patients with Persistent Post-COVID Syndrome: A Systematic Review | All health conditions | Both |  | X | X |
| vandeGraaf 2021 | Online Acceptance and Commitment Therapy (ACT) interventions for chronic pain: A systematic literature review | Pain | Both | X |  |  |
| vandenBosch 2019 | Oral & Maxillofacial surgery is ready for patient-centred eHealth interventions - the outcomes of a scoping review | Multiple | Virtual NOS | X | X | X |
| VanDenHeuvel 2018 | eHealth as the next-generation perinatal care: An overview of the literature | Maternal health | Both | X | X | X |
| vanderBoom 2022 | Internet-delivered interventions for personality disorders - A scoping review | Mental health | Video | X | X | X |
| vanEgmond 2018 | Effectiveness of physiotherapy with telerehabilitation in surgical patients: a systematic review and meta-analysis | Surgery | Both |  |  | X |
| vanEijck 2023 | Digital Health Applications to Establish a Remote Diagnosis of Orthopedic Knee Disorders: Scoping Review | Musckuloskeletal | Both | X |  |  |
| vanHuizen 2021 | Benefits and drawbacks of videoconferencing for collaborating multidisciplinary teams in regional oncology networks: a scoping review | Cancer | Video |  |  |  |
| vanKessel 2022 | Clients' experiences of online therapy in the early stages of a COVID-19 world: A scoping review | Mental health | Both |  |  | X |
| vanLeeuwen 2021 | Reviewing the availability, efficacy and clinical utility of Telepsychology in dialectical behavior therapy (Tele-DBT) | Mental health | Both | X | X | X |
| vanLoon-vanGaalen 2021 | The effect of a telephone follow-up call for older patients, discharged home from the emergency department on health-related outcomes: a systematic review of controlled studies | Acute/ urgent care | Telephone | X | X | X |
| vanVelthoven 2013 | Telephone delivered interventions for preventing HIV infection in HIV-negative persons | Infectious disease | Telephone |  | X |  |
| Varela-Moreno 2022 | Effectiveness of eHealth-Based Psychological Interventions for Depression Treatment in Patients With Type 1 or Type 2 Diabetes Mellitus: A Systematic Review | Metabolic | Telephone |  |  | X |
| Vargas 2017 | Use of Short Messaging Service for Hypertension Management | Cardiovascular | Telephone |  |  | X |
| Veiga 2022 | A systematic review on smartphone use for activity monitoring during exercise therapy in intermittent claudication | Cardiovascular | Both |  | X | X |
| Velayati 2020 | A Systematic Review of the Effectiveness of Telerehabilitation Interventions for Therapeutic Purposes in the Elderly | Geriatric | Both |  |  | X |
| Vellata 2021 | Effectiveness of Telerehabilitation on Motor Impairments, Non-motor Symptoms and Compliance in Patients With Parkinson's Disease: A Systematic Review | CNS | Both | X |  | X |
| Venkataramanan 2022 | Digital Inequalities in Cancer Care Delivery in India: An Overview of the Current Landscape and Recommendations for Large-Scale Adoption | Cancer | Telephone |  |  | X |
| Verburg 2019 | eHealth to improve patient outcome in rehabilitating myocardial infarction patients | Cardiovascular | Telephone |  | X | X |
| Verma 2021 | Telemedicine in India - an investment of technology for a digitized healthcare industry: a systematic review | All health conditions | Both |  |  |  |
| Verma 2021 | Effect of mHealth Interventions on Glycemic Control and HbA1c Improvement among Type II Diabetes Patients in Asian Population: A Systematic Review and Meta-Analysis | Metabolic | Telephone |  |  | X |
| Verma 2022 | Patients' and physicians' experiences with remote consultations in primary care during the COVID-19 pandemic: a multi-method rapid review of the literature | Multiple | Video |  |  | X |
| Vervloet 2020 | Interventions to Improve Adherence to Cardiovascular Medication: What About Gender Differences? A Systematic Literature Review | Cardiovascular | Telephone |  | X |  |
| Vieira 2022 | Telerehabilitation improves physical function and reduces dyspnoea in people with COVID-19 and post-COVID-19 conditions: a systematic review | Infectious disease | Video | X |  | X |
| Vila 2023 | Telemedicine in the management of chronic obstructive pulmonary disease: A systematic review | Respiratory | Both | X |  | X |
| Virtanen 2021 | Behavior change techniques to promote healthcare professionals' eHealth competency: A systematic review of interventions | All health conditions | Internet-based NOS |  |  |  |
| Vo 2023 | Telepharmacy in oncology care: A scoping review | Cancer | Both |  |  |  |
| Vodicka 2022 | Remote Consultations in General Practice - A Systematic Review | Multiple | Both |  |  |  |
| Vyas 2017 | A Systematic Review of the Use of Telemedicine in Plastic and Reconstructive Surgery and Dermatology | Wound | Both |  |  |  |
| Wake 2022 | Telehealth in trauma: A scoping review | Mental health | Both | X |  | X |
| Wali 2019 | Investigating the Use of Mobile Health Interventions in Vulnerable Populations for Cardiovascular Disease Management: Scoping Review | Cardiovascular | Telephone |  | X |  |
| Walker 2017 | Home Telehealth Interventions for Older Adults With Diabetes | Metabolic | Both | X | X | X |
| Wallace 2021 | Group and individual telehealth for chronic musculoskeletal pain: A scoping review | Pain | Both |  | X | X |
| Waller 2017 | Computer and telephone delivered interventions to support caregivers of people with dementia: A systematic review of research output and quality | CNS | Both | X | X | X |
| Walsh 2020 | Using Telehealth for Pediatric, Adolescent, and Adult Sexual Assault Forensic Medical Examinations: An Integrative Review | Assault | Video |  |  | X |
| Walton 2023 | Telehealth Palliative Care in Nursing Homes: A Scoping Review | End of life | Both |  | X | X |
| Walumbe 2021 | Pain management programmes via video conferencing: a rapid review | Pain | Video | X |  | X |
| Wan 2022 | A systematic review and meta-analysis on the effectiveness of web-based psychosocial interventions among patients with colorectal cancer | Cancer | Multiple |  | X | X |
| Wanderas 2023 | Video consultation in general practice: a scoping review on use, experiences, and clinical decisions | Multiple | Video |  | X | X |
| Wang 2019 | Electronic Health Interventions to Improve Adherence to Antiretroviral Therapy in People Living With HIV: Systematic Review and Meta-Analysis | Infectious disease | Telephone |  | X |  |
| Wang 2019 | Technology-assisted rehabilitation following total knee or hip replacement for people with osteoarthritis: a systematic review and meta-analysis | Musckuloskeletal | Both | X |  | X |
| Wang 2020 | Effects of Internet-based psycho-educational interventions on mental health and quality of life among cancer patients: a systematic review and meta-analysis | Cancer | Video |  |  | X |
| Wang 2021 | The effectiveness of internet-based telerehabilitation among patients after total joint arthroplasty: An integrative review | Musckuloskeletal | Both |  | X | X |
| Wang 2021 | Influencing Factors of Acceptance and Use Behavior of Mobile Health Application Users: Systematic Review | All health conditions | Mobile App |  |  |  |
| Wang 2022 | mHealth Interventions to Promote a Healthy Diet and Physical Activity among Cancer Survivors: A Systematic Review of Randomized Controlled Trials | Cancer | Both | X | X | X |
| Wang 2023 | The effectiveness of internet-based telerehabilitation among patients after total joint arthroplasty: A systematic review and meta-analysis of randomised controlled trials | Musckuloskeletal | Both |  |  | X |
| Ward 2022 | Visit Types in Primary Care With Telehealth Use During the COVID-19 Pandemic: Systematic Review | Multiple | Both |  |  |  |
| Washio 2022 | A Scoping Review of Computer-Based and Telecommunication Technology Interventions to Address Drug and Alcohol Misuse and Smoking in Women | Mental health | Both |  | X | X |
| Watanabe 2023 | Telemental health in rural areas: a systematic review | Mental health | Both |  |  | X |
| Wattanapisit 2020 | Usability and utility of eHealth for physical activity counselling in primary health care: a scoping review | Multiple | Telephone |  |  | X |
| Wechkunanukul 2020 | Utilising digital health to improve medication-related quality of care for hypertensive patients: An integrative literature review | Cardiovascular | Telephone | X | X | X |
| Wei 2019 | Effects of telephone call intervention on cardiovascular risk factors in T2DM: A meta-analysis | Cardiovascular | Telephone |  |  | X |
| Western 2021 | The effectiveness of digital interventions for increasing physical activity in individuals of low socioeconomic status: a systematic review and meta-analysis | Not specified | Both |  | X |  |
| White 2022 | Online psychological interventions to reduce symptoms of depression, anxiety, and general distress in those with chronic health conditions: a systematic review and meta-analysis of randomized controlled trials | Multiple | Internet-based NOS | X |  | X |
| Whitehead 2023 | Barriers to and Facilitators of Digital Health Among Culturally and Linguistically Diverse Populations: Qualitative Systematic Review | Multiple | Both |  | X |  |
| Widberg 2020 | Patients' experiences of eHealth in palliative care: an integrative review | End of life | Both |  |  | X |
| Widmer 2015 | Digital health interventions for the prevention of cardiovascular disease: a systematic review and meta-analysis | Cardiovascular | Multiple |  | X | X |
| Wikstrom 2022 | Patients' and providers' perspectives on e-health applications designed for self-care in association with surgery - a scoping review | Surgery | Both |  |  | X |
| Wildenbos 2016 | Impact of Patient-centered eHealth Applications on Patient Outcomes: A Review on the Mediating Influence of Human Factor Issues | All health conditions | Multiple |  | X |  |
| Wong 2020 | How is telehealth being utilized in the context of rehabilitation for lower limb musculoskeletal disorders: a scoping review | Musckuloskeletal | Both |  |  | X |
| Wong 2022 | Effects of a Nurse-Led Telehealth Self-care Promotion Program on the Quality of Life of Community-Dwelling Older Adults: Systematic Review and Meta-analysis | Geriatric | Telephone |  | X | X |
| Wongvibulsin 2021 | Digital Health Interventions for Cardiac Rehabilitation: Systematic Literature Review | Cardiovascular | Both | X | X | X |
| Woo 2018 | Factors Affecting the Acceptance of Telehealth Services by Heart Failure Patients: An Integrative Review | Cardiovascular | Telephone |  |  |  |
| Woodley 2021 | Can teledermatology meet the needs of the remote and rural population? | Cancer | Video |  |  |  |
| Woods 2019 | Nurse-led postdischarge telephone follow-up calls: A mixed study systematic review | Acute/ urgent care | Telephone | X | X | X |
| Wright 2021 | Are eHealth interventions for adults who are scheduled for or have undergone bariatric surgery as effective as usual care? A systematic review | Surgery | Both |  | X | X |
| Wright 2021 | A systematic review of telehealth for the delivery of emergent neurosurgical care | CNS | Both |  | X |  |
| Wu 2018 | Evaluation of the clinical outcomes of telehealth for managing diabetes: A PRISMA-compliant meta-analysis | Metabolic | Both |  |  | X |
| Wu 2022 | Behavioral Change Factors and Retention in Web-Based Interventions for Informal Caregivers of People Living With Dementia: Scoping Review | CNS | Both | X | X | X |
| Wu 2023 | The Efficacy and Safety of Telerehabilitation for Fibromyalgia: Systematic Review and Meta-analysis of Randomized Controlled Trials | Pain | Multiple |  |  | X |
| Xiao 2021 | A Systematic Review and Meta-analysis of Telephone-Based Therapy Targeting Depressive Symptoms Among Low-Income People Living with HIV | Infectious disease | Telephone |  |  | X |
| Xiao 2023 | Evaluation of the Effectiveness of Telehealth Chronic Disease Management System: Systematic Review and Meta-analysis | Multiple | Telephone |  | X | X |
| Xie 2020 | Effectiveness of telemedicine for pregnant women with gestational diabetes mellitus: an updated meta-analysis of 32 randomized controlled trials with trial sequential analysis | Maternal health | Both |  |  | X |
| Xie 2022 | Consumers' Willingness to Pay for eHealth and Its Influencing Factors: Systematic Review and Meta-analysis | All health conditions | Both | X |  |  |
| Xu 2019 | Effectiveness of e-health based self-management to improve cancer-related fatigue, self-efficacy and quality of life in cancer patients: Systematic review and meta-analysis | Cancer | Telephone |  | X | X |
| Xu 2023 | Telehealth in palliative care during the COVID-19 pandemic: A systematic mixed studies review | End of life | Both |  |  | X |
| Yadav 2019 | Utilising Digital Health Technology to Support Patient-Healthcare Provider Communication in Fragility Fracture Recovery: Systematic Review and Meta-Analysis | Musckuloskeletal | Telephone |  | X | X |
| Yammine 2022 | Telemedicine and diabetic foot ulcer outcomes. A meta-analysis of controlled trials | Metabolic | Video |  |  | X |
| Yang 2019 | The role of telenursing in the management of diabetes:A systematic review and meta-analysis | Metabolic | Both |  |  | X |
| Yang 2020 | Intervention and Evaluation of Mobile Health Technologies in Management of Patients Undergoing Chronic Dialysis: Scoping Review | GU | Both | X | X | X |
| Yang 2022 | Effectiveness of telehealth-based exercise interventions on pain, physical function and quality of life in patients with knee osteoarthritis: A meta-analysis | Musckuloskeletal | Video |  |  | X |
| Yang 2022 | Effect of telehealth interventions on anxiety and depression in cancer patients: A systematic review and meta-analysis of randomized controlled trials | Cancer | Telephone |  |  | X |
| Yanicelli 2021 | Heart failure non-invasive home telemonitoring systems: A systematic review | Cardiovascular | Both |  |  | X |
| Yao 2022 | Inequities in Health Care Services Caused by the Adoption of Digital Health Technologies: Scoping Review | Not specified | Video | X |  |  |
| Yap 2021 | Effectiveness of technology-based psychosocial interventions on diabetes distress and health-relevant outcomes among type 2 diabetes mellitus: A systematic review and meta-analysis | Metabolic | Telephone |  | X | X |
| Yap 2022 | The Feasibility and Efficacy of Telespirometry for Pulmonary Monitoring of Cystic Fibrosis: A Systematic Review | Respiratory | Both | X |  | X |
| Yasmin 2016 | Positive influence of short message service and voice call interventions on adherence and health outcomes in case of chronic disease care: A systematic review | Multiple | Both |  | X | X |
| Ye 2016 | Internet-based cognitive-behavioural therapy for insomnia (ICBT-i): a meta-analysis of randomised controlled trials | CNS | Both |  |  | X |
| Yeroushalmi 2020 | Telemedicine and multiple sclerosis: A comprehensive literature review | CNS | Both | X |  | X |
| Yerrakalva 2019 | Effects of Mobile Health App Interventions on Sedentary Time, Physical Activity, and Fitness in Older Adults: Systematic Review and Meta-Analysis | Not specified | Telephone |  | X | X |
| Yeun 2022 | Psychological Effects of Online-Based Mindfulness Programs during the COVID-19 Pandemic: A Systematic Review of Randomized Controlled Trials | Mental health | Internet-based NOS |  |  | X |
| Yew 2023 | Telemedicine in Vascular Surgery During COVID-19 Pandemic: A Systematic Review and Narrative Synthesis | Cardiovascular | Both | X |  | X |
| Yi 2021 | Telemedicine and Dementia Care: A Systematic Review of Barriers and Facilitators | CNS | Both | X |  | X |
| Young 2021 | Video-Based Telemedicine for Kidney Disease Care: A Scoping Review | GU | Video |  | X | X |
| Yu 2022 | Design of an Integrated Acceptance Framework for Older Users and eHealth: Influential Factor Analysis | Geriatric | Multiple | X |  |  |
| Zaheer 2022 | The use of mobile devices in oculoplastic and oral and maxillofacial surgery: A systematic review | Denistry | Telemedicine NOS |  |  |  |
| Zamarron 2017 | Telemedicine in chronic obstructive pulmonary disease: Clinical, economic and organizational impact | Respiratory | Both | X | X | X |
| Zandbelt 2016 | E-consulting in a medical specialist setting: Medicine of the future? | All health conditions | Video | X | X | X |
| Zangani 2022 | Impact of the COVID-19 Pandemic on the Global Delivery of Mental Health Services and Telemental Health: Systematic Review | Mental health | Both |  |  |  |
| Zeng 2022 | Impact of mobile health and telehealth technology on medication adherence of stroke patients: a systematic review and meta-analysis of randomized controlled trials | Cardiovascular | Telephone |  | X |  |
| Zhai 2015 | Efficacy of telemedicine for thrombolytic therapy in acute ischemic stroke: a meta-analysis | Cardiovascular | Telephone |  |  | X |
| Zhang 2018 | Effectiveness of telephone-based interventions on health-related quality of life and prognostic outcomes in breast cancer patients and survivors-A meta-analysis | Cancer | Telephone |  | X | X |
| Zhang 2021 | Effect of Telemedicine on Quality of Care in Patients with Coexisting Hypertension and Diabetes: A Systematic Review and Meta-Analysis | Metabolic | Telephone |  | X | X |
| Zhang 2022 | Effect of a telehealth-based exercise intervention on the physical activity of patients with breast cancer: A systematic review and meta-analysis | Cancer | Telephone |  | X | X |
| Zhang 2022 | A Meta-Analysis of the Effectiveness of Telemedicine in Glycemic Management among Patients with Type 2 Diabetes in Primary Care | Metabolic | Both |  | X | X |
| Zhang 2022 | Caregivers' experiences and perspectives on caring for the elderly during the COVID-19 pandemic: A qualitative systematic review | All health conditions | Both |  |  | X |
| Zhang 2023 | Effectiveness of synchronous teleconsultation for patients with type 2 diabetes mellitus: A systematic review and meta-analysis | Metabolic | Both |  | X | X |
| Zhao 2021 | Effectiveness of Telehealth Interventions for Women With Postpartum Depression: Systematic Review and Meta-analysis | Maternal health | Telephone |  |  | X |
| Zheng 2016 | A Systematic Review of Telehealth in Palliative Care: Caregiver Outcomes | End of life | Both | X |  | X |
| Zheng 2020 | Technology-based interventions in oral anticoagulation management: Meta-analysis of randomized controlled trials | Multiple | Telephone |  |  | X |
| Zhou 2019 | Reaching people with disabilities in underserved areas through digital interventions: Systematic review | Multiple | Video | X | X | X |
| Zhou 2022 | The effectiveness of mHealth interventions on postpartum depression: A systematic review and meta-analysis | Maternal health | Multiple |  |  | X |
| Zhou 2023 | Effects of e-health interventions on health outcomes in patients with rheumatoid arthritis: a systematic review and meta-analysis | Autoimmune | Telephone |  | X | X |
| Zhu 2017 | Integrative Review on the Effectiveness of Internet-Based Interactive Programs for Women With Breast Cancer Undergoing Treatment | Cancer | Telephone |  | X | X |
| Zhu 2020 | Effectiveness of telemedicine systems for adults with heart failure: a meta-analysis of randomized controlled trials | Cardiovascular | Telemedicine NOS | X |  | X |
| Zischke 2021 | The utility of physiotherapy assessments delivered by telehealth: A systematic review | Rehabilitation | Both | X | X | X |
