# Supplementary material for: Virtual Care, What Are We Measuring and What Should We Measure? Scoping Review of Reviews
Source: J Med Internet Res. 2025 Dec 1;27:e65312. doi: 10.2196/65312 (PMC12670043; doi:10.2196/65312)
Supplement: Multimedia Appendix 3 [file jmir-v27-e65312-s003.docx]

**Multimedia Appendix 3.** Summary of measures from review articles by Proctor outcome domain and construct.

| **Domain** | **Construct** | | | **Measures** | **Summary and Methods** |
| --- | --- | --- | --- | --- | --- |
| **Implementation Outcomes (38.6%; 476/1233)** | Acceptability (22.9%; 109/476) | | Patient Perspective (96.3%; 105/109) | - Acceptability NOS (38.1%; 40/105) - Satisfaction (33.3%; 35/105) - Perceived usefulness/ agreeability (19.0%; 20/105) - Uptake of VC/ Number of sessions attended (19.0%; 20/105) - Retention/ completion rates (16.2%; 17/105) - Ease of use (12.4%; 13/105) - Willingness to use/ recommend VC (10.5%; 11/105) - Drop-out rate (8.6%; 9/105) - Reliability/ rate of technical difficulties (6.7%; 7/105) - Proportion of patients enrolled in VC out of those eligible (5.7%; 6/105) - Preference for VC (5.7%; 6/105) - Privacy or Trust in VC (4.7%; 5/105) - Confidence/ comfort with using VC (4.7%; 5/105) - Factors associated with VC acceptance (3.8%; 4/105) - Clinicians’ perception of patient’s acceptance of VC (2.8%; 3/105) - Likability (1.9%; 2/105) - Prevalence of apps/ VC modalities (1.9%; 2/105) - Compatibility with Clinical Scenario (1.9%; 2/105) - Rate of return users (1.9%; 2/105) - Perceived quality of VC (1.9%; 2/105) - Context of Use (0.95%; 1/105) - Burden of using VC (0.95%; 1/105) - Attitude towards VC (0.95%; 1/105) - Readiness for use of VC (0.95%; 1/105) - Accessibility (0.95%; 1/105) | (29.5%; 31/105) studies reported that VC was to be acceptable to patients, though there was high heterogeneity in the measures/ definitions used whereby 25 separate measures were identified. A large proportion of articles did not include the definition of acceptability (30.1%; 40/105). Most common measures of acceptability were satisfaction either with VC as a whole or with its individual components (33.3%; 35/105), perceived usefulness/ agreeability of VC (19.1%; 20/105), and uptake/ number of VC sessions attended (19.1%; 20/105). Interestingly, some articles (2.8%; 3/105) reported on clinicianr’s perceptions of whether patients found VC acceptable rather than measuring their acceptance directly.  Methods for assessing acceptability were most often:   - Qualitative (23.8%; 25/105):   - Interviews (64%; 16/25)   - Focus groups (12%; 3/25)   - Analysis of video recordings (8%; 2/25) - Quantitative NOS (2.9%; 3/105) - Usage or interaction logs (1.9%; 2/105) - Surveys (38.1%; 40/105); measures included: - Telemedicine Satisfaction and Acceptance Scale (5%; 2/40) - Telehealth Usability Questionnaire (2.5%; 1/40) - System Usability Scale (10%; 4/40) - Client Satisfaction Questionnaire (5%; 2/40) - After Scenario Questionnaire (2.5%; 1/40) - Participant Satisfaction and Global Assessment Scale (5%; 2/40) - Unified Theory of Acceptance and Use of Technology scale (2.5%; 1/40) - Technology Acceptance Model (2.5%; 1/40) - Treatment Acceptability Rating Questionnaire (5%; 2/40) - Mobile Application Rating Scale (5%; 2/40) - Likert scales- NOS (12.5%; 5/40) |
|  |  |  | Clinician Perspective (17.4%; 19/109) | - Acceptability NOS (31.6%; 6/19) - Ease of use (21.1%; 4/19) - Perceived usefulness (10.5%; 2/19) - Perceptions of individual characteristics of VC (10.5%; 2/19) - Willingness to continue (5.2%; 1/19) - Perceived benefits of VC (5.2%; 1/19) - Attitudes towards VC (5.2%; 1/19) - Proportion of visits without technical issues (10.5%; 2/19) - Proportion of visits completed using VC (5.2%; 1/19) - Recruitment rate (5.2%; 1/19) - Refusal rate (5.2%; 1/19) | Most articles reported that virtual care was acceptable to clinicians, and/or reported positive perceptions (63.2%; 12/19). There was significant heterogeneity in measures of acceptability. Most commonly, acceptability was not defined (31.6%; 6/19), utilized surveys to capture perceptions particularly around ease of use (10.5%; 2/19) and perceived usefulness (10.5%; 2/19), or used administrative or chart abstraction data to capture quantitative measures such as the proportion of visits without technical issues (10.5%; 2/19).  Methods most commonly used to assess acceptability were:   - Qualitative NOS (21.1%; 4/19) - Narrative results (10.5%; 2/19) - Surveys (68.4%; 13/19); no validated measures were cited, but few articles indicated that measures utilized:   - Likert (23.1%; 3/13)   - Visual analog scale (7.7%; 1/13) - Interviews (21.1%; 4/19) - Administrative billing data/ chart data (15.8%; 3/19) - Observational data NOS (5.2%; 1/19) |
|  | Adoption (28.4%; 135/476) | | Patient Perspective (92.6%; 125/135) | - Number of VC visits/ Attendance (40%; 50/125) - Completion rates (28.8%; 36/125) - Adoption NOS (16%; 20/125) - Retention/ drop-out rates (16%; 20/125) - Frequency of engagement with VC (10.4%; 13/125) - Enrollment (7.2%; 9/125) - Factors associated with adoption (7.2%; 9/125) - Satisfaction (6.4%; 8/125) - Engagement over time (6.4%; 8/125) - No show/ cancellation rates (5.6%; 7/125) - Duration of visit (4.8%; 6/125) - Proportion of patients receiving VC out of those eligible (4%; 5/125) - Number of Logins (4%; 5/125) - Proportion of services transitioned from in-person to VC (3.2%; 4/125) - Willingness to use/ adopt VC (2.4%; 3/125) - Completion of training (1.6%; 2/125) - Perception of improved access to care (1.6%; 2/125) - Usage NOS (0.8%; 1/125) - Preference for specific VC modality (0.8%; 1/125) - Clinicians’ perceptions of patient adoption (0.8%; 1/125) - VC acceptance rate NOS (0.8%; 1/125) - Return users (0.8%; 1/125) - Context of use (0.8%; 1/125) - Location of use (0.8%; 1/125) - Technical difficulties (0.8%; 1/125) | A large proportion of articles did not provide a clear definition of adoption (1.6%; 2/125). For articles that did define adoption, there was high heterogeneity amongst measures; the most common definitions were objective measures of attendance or the number of VC visits completed (40%; 50/125), completion rate (28.8%; 36/125), and retention/ drop-out rates (16%; 20/125).  Methods for data collection were not described often. For studies utilizing qualitative data, the following collection methods were reported:   - Interviews (6.4%; 8/125) - Focus Groups (2.4%; 3/125) - Analysis of session recordings (0.8%; 1/125)   9 articles described the use of surveys (7.2%; 9/125), which included the following measures:   - Client Satisfaction Questionnaire (11.1%; 1/9) - Likert scale NOS (11.1%; 1/9) - Visual analog scale NOS (11.1%; 1/9) - Adherence checklist NOS (11.1%; 1/9) |
|  |  |  | Clinician Perspective (5.2%; 7/135) | - Competence with technology (42.9%; 3/7) - Willingness to use (42.9%; 3/7) - Proportion of visits completed using VC (42.9%; 3/7) - Workload associated with virtual visits (42.9%; 3/7) - Proportion of clinicians using VC (28.6%; 2/7) - Clinician-level factors associated with adoption (28.6%2/7) - Uptake of clinician training (28.6%; 2/7) - Practice-level factors associated with adoption (14.3%; 1/7) - Compliance with technology (14.3%; 1/7) - Clinician preference for VC (14.3%; 1/7) - Refusal rates (14.3%; 1/7) | The most commonly reported measures of clinician adoption of VC were competence with the technology (42.9%; 3/7) though specific measures were not defined, willingness to use VC (42.9%; 3/7), the proportion of visits completed using VC (42.9%; 3/7), and clinician workload associated with delivering VC (42.9%; 3/7).  Three different measurements were utilized to quantify physician workload quantification:   - EHR hours spent documenting/ ordering (33.3%; 1/3) - Utilized validated tool but tool was not specified (33.3%; 1/3) - Clinicians time conducting with virtual visits (33.3%; 1/3)   Most articles presented findings narratively (71.4%; 5/7); one used a validated measure (14.3%; 1/7) but did not specify which measure, two specified qualitative methods (28.6%; 2/7), though only one indicated that they used interviews and focus groups (14.3%; 1/7). |
|  |  |  | System Perspective (1.5%; 2/135) | - Uptake- NOS (50%; 1/2) - Proportion of practices using VC out of those where VC was available (50%; 1/2) - Regulatory Factors (50%; 1/2) | Few articles examined adoption from the system perspective, and focused on uptake at the level of the practice or institution (50%; 1/2), or the proportion of practices using virtual care out of those where virtual care was available (50%; 1/2). One article also described qualitative themes around the need for support from the government to overcome regulatory factors affecting system-level adoption (50%; 1/2), such as geographic restrictions to licensure preventing cross-jurisdictional practice. |
|  | Appropriateness (1.9%; 9/476) | | | - Perceived compatibility/ fit (33.3%; 3/9) - Cultural appropriateness (11.1%; 1/8) - Produced a guideline/ examined specific clinical scenarios (22.2%; 2/9) - Appropriateness- NOS (33.3%; 3/9) | Appropriateness was not always defined in articles (33.3%; 3/9), but where it was defined it most often captured end users perceptions of the compatibility of VC for the clinical scenario (33.3%; 3/9), or examined or produced a guideline for specific clinical scenarios under which VC could be used (22.2%; 2/9).  All articles utilized qualitative methods to assess appropriateness (100%; 9/9), though few specified the methods; those that did utilized interviews (22.2%; 2/9) and focus groups (11.1%; 1/9). Some articles reported on surveys (33.3%; 3/9) though no specific validated measures were reported. |
|  | Costs (46.4%; 221/476) | | Direct/ indirect costs (28.9%; 64/221) | - Intervention cost/ cost of delivering VC (50%; 32/64) - Comparative cost analysis relative to in-person care (25%; 16/64) - Out of pocket expenditure (15.6%; 10/64) - Implementation cost/ infrastructure cost (14.1%; 9/64) - Revenue generated (7.8%; 5/64) - Healthcare expenditure (3.1%; 2/64) - Cost NOS (3.1%; 2/64) - Opportunity Cost (1.5%; 1/64) - VC maintenance costs (1.5%; 1/63) | Many of the articles reporting on the costs associated with VC just quantified direct/indirect costs (28.9%; 64/221). These analyses were most frequently conducted from the system perspective (87.5%; 56/64), while few examined the patient (15.6%; 10/64) or societal perspectives (3.1%; 2/64). The most commonly reported measures were the direct/indirect costs of delivering VC (50%; 32/64), a comparison of the costs of delivering VC relative to care delivered in-person (25%; 16/64), or out-of-pocket expenditures from the patient perspective (15.6%; 10/64). Only one article reported on the long-term costs of sustaining VC delivery beyond the initial implementation (1.5%; 1/63). |
|  |  |  | Cost-Effectiveness (27.6%; 61/221) | - Incremental cost effectiveness ratio (55.7%; 34/61) - Cost effectiveness NOS (44.3%; 27/61) - Willingness to pay (4.9%; 3/61) - Cost effectiveness of implementation (3.3%; 2/61) | The cost-effectiveness of VC was most commonly reported as the incremental cost effectiveness ration (55.7%; 34/61). The perspective taken for the analysis was rarely reported but was most frequently the health system perspective (21.3%; 13/61), followed by the patient (13.1%; 8/61) and societal perspectives (8.2%; 5/61). |
|  |  |  | Cost utility (24.0%; 53/221) | - Cost per quality adjusted life years gained (90.5%; 48/53) - Cost per disability adjusted life year gained (11.3%; 6/53) - Cost utility analysis NOS (9.4%; 5/53) - Cost per health adjusted life year gained (5.6%; 3/53) - Willingness to pay threshold analysis (5.6%; 3/53) - Cost per life year gained (1.8%; 1/53) | For articles reporting on cost utility analyses (24.0%; 53/221), results were most frequently reported as the cost per quality adjusted life year (QALY [Quality-adjusted life years]; 90.5%; 48/52) or disability adjusted life year (DALY [Disability-adjusted life years]; 11.3%; 6/53) gained. Almost all of the reported analyses were conducted from the health system perspective (92.4%; 49/53), rather that the patient (5.6%; 3/53) or societal perspectives (1.9%; 1/53).  Measures utilized to calculate utility values were not always reported; however, the following validated measures were reported:   - EQ-5D (29.7%; 11/53) - Short form 36 item (SF-36; 7.5%; 4/53) - Short form 12 item (SF-12; 1.8%; 1/53) - Quality of Well Being Questionnaire (1.8%; 1/53) - Dartmouth Coop Chart Questionnaire (1.85; 1/53) - ICE-CAP-O (1.8%; 1/53) |
|  |  |  | Cost- other (37.5%; 83/221) | - Cost savings (74.6%; 62/83) - Cost-benefit analysis (9.6%; 8/83) - Cost minimization analysis (8.4%; 7/83) - Cost reduction (8.4%; 7/83) - Cost avoided (3.6%; 3/83) - Budget impact analysis (1.2%; 1/83) - Cost consequence analysis (1.2%; 1/83) | Outside of the above categories, the most frequent type of costing analysis was a cost savings analysis (74.6%; 62/83) from the health system perspective, most frequently reported relative to delivering in-person care. |
|  | Feasibility (22.6%; 108/476) | | | - Feasibility- NOS (25%; 27/108) - Number of sessions delivered/ frequency (8.3%; 9/108) - Utility (5.6%; 6/108) - Use over time/ engagement (5.6%; 6/108) - Clinical Capacity NOS (2.7%; 3/108) - Outpatient clinic slots freed up (1.85%; 2/108) - Missed visits (0.92%; 1/108) - Duration of visit (0.92%; 1/108) - Withdrawals (0.92%; 1/108) - Goodness of fit (0.92%; 1/108) | While many articles reported on feasibility of VC (22.6%; 108/476), it was often poorly defined or undefined (25%; 27/108). Most frequently, feasibility was defined as the number of sessions delivered/ frequency of VC sessions (8.3%; 9/108), perceptions surrounding the utility of VC in particular clinical scenarios (5.6%; 6/108), and usage/ engagement over time (5.6%; 6/108). Most articles did not specify methods. For those that did, the following methods were utilized:   - Quantitative NOS (4.6%; 5/108) - Surveys- no validated measures described (11.1%; 12/108) - Qualitative NOS (4.6%; 5/108) - Focus groups (1.85%;2/108) - Interviews (5.55%; 6/108) |
|  | Fidelity (9.5%; 45/476) | | Intervention Fidelity (95.6%; 43/45) | - Diagnostic accuracy relative to in-person (74.41%; 32/43) - Inter-assessor reliability of assessments (13.9%; 6/43) - Concordance with intervention manual/ protocol (23.2%; 10/43) - Concordance with in-person protocols (6.9%; 3/43) - Social validity/ perception that care is acceptable/ accurate (4.6%; 2/43) - Concordance with training (4.6%; 2/43) - Consistency of delivery (2.32%; 1/43) - Impact of technical challenges on intervention fidelity (2.32%; 1/43) - Skills following training on VC (2.32%; 1/43) - Clinician’s self-reported competence (2.32%; 1/43) | 43 studies reported on VC intervention fidelity (95/6%; 43/45). Intervention fidelity was most frequently reported as the diagnostic accuracy of virtual assessments relative to in-person visits (74.4%; 32/43), inter-assessor reliability of assessments (13.9%; 6/43), and concordance with an intervention manual/ protocol (23.2%; 10/43). Intervention fidelity was most frequently assessed in studies involving uploading of photos for dermatological assessment, and for diagnosing cognitive disorders such as autism.  Methods for assessing intervention fidelity were rarely defined, however, articles reported using the following methods:   - Random sampling of recordings of VC visits (2.32%; 1/43) - Real-time diary entries (2.32%; 1/43) - Surveys- no validated measures described (6.9%; 3/43) - Monthly case review sessions (2.32%; 1/43) - Checklist/ standardized coding scheme (9.3%; 4/43):   - Motivational Interviewing Treatment Integrity Tool (75%; 3/4)   - Motivational Interviewing Skill Code Tool (25%; 1/4) |
|  |  |  | Implementation fidelity (4.4%; 2/45) | - Ability to replicate the in-person experience in VC (50%; 1/2) - Ability to carry out implementation steps (50%; 1/2) | Few articles reported on assessing implementation fidelity (4.4%; 2/45); of these articles, measures and methods were poorly defined. |
|  | Penetration/ Access (5.0%; 24/476) | | | - Narrative results- NOS (37.5%; 9/24) - Availability of VC (20.8%; 5/24) - Satisfaction with access (16.6%; 4/24) - Sociodemographic factors associated with access (16.6%; 4/24) - Awareness of VC (12.5%; 3/24) - Visit rate (12.5%; 3/24) - Access to technology/ infrastructure (8.3%; 2/24) - Follow-up Rate (8.3%; 2/24) - Missed appointment/ attendance rate (12.5%; 3/24) - Ease of Use (4.2%; 1/24) - Convenience (4.2%; 1/24) - Digital Literacy (4.2%; 1/24) | Penetration/ access to VC were poorly defined with most studies (37/5%; 9/24) reporting narrative results only, or reporting on the availability of VC within a specific population or geography (20.8%; 5/24). None of the articles reported on validated measures. Populations that articles examining access focused on included:   - Indigenous people (4.2%; 1/24) - Trans people (4.2%; 1/24) - Access by Race/ Ethnicity (12.5%; 3/24) - People with communication impairments/ cognitive challenges (4.2%; 1/24) - People residing in rural/ remote (16.6%; 4/24) - Older people (4.2%; 1/24) |
|  | Sustainability (2.1%; 10/476) | | Of the Intervention (70%; 7/10) | - Whether the intervention has been maintained (57.1%; 4/7) - Embedding into practice (28.6%; 2/7) - Financial sustainability/ availability of funds to continue (28.6%; 2/7) - User preference to continue VC (14.3%; 1/7) - Sustainability NOS (14.3%; 1/7) | Sustainability of the intervention long-term was only evaluated in a handful of articles (2.1%; 10/476). It was most frequently reported as whether VC had been maintained (57.1%; 4/7) usually 6-12 months after the study period or VC implementation. Other articles examined embeddedness into practice (28.6%; 2/7) but did not reference utilization of any validated measures. |
|  |  |  | Environmental (30%; 3/10) | - Carbon footprint (100%; 3/3) - Travel distance to clinic (66.6%; 2/3) | Few articles examined the environmental impacts of VC (30%; 3/10), with all articles reporting on the impact on carbon footprint (100%; 3/3), most commonly calculated as Kg CO2/ consultation (66.6%; 2/3), though one article did not specify (33.3%; 1/3). Two articles also reported on the impact of VC on travel distance (66.6%; 2/3), though only one reported on units as Km/ consultation (33.3%; 1/3). |
| **Service Outcomes (41.1%; 507/1233)** | Efficiency- resource utilization (15.6%; 79/507) | | | - Number of visits (69.6%; 55/79) - Length of stay (17.7%; 14/79) - Visit duration (11.3%; 9/79) - Frequency of consultations per patient (10.1%; 8/79) - Conversion of visits from VC to in-person (7.5%; 6/79) - Number of unnecessary transfers (6.3%; 5/79) - Follow-up rate (6.3%; 5/79) - Resource utilization NOS (5.1%; 4/79) - Preventable visits avoided (5.1%; 4/79) - Doctor’s contact time (3.79%; 3/79) - Infrastructure needed to establish VC (3.79%; 3/79) - Discharge destination (2.53%; 2/79) - Types of consultations (2.53%; 2/79) - Follow-up rate (1.26%; 1/79) - Economic resources NOS (1.26%; 1/79) - Societal burden (1.26%; 1/79) - Change in clinical capacity (1.26%; 1/79) - No show rate (1.26%; 1/79) - Change in monthly visit rate over time (1.26%; 1/79) | 79 articles reporting on service outcomes included measures of efficiency (15.6%), focusing on the impact of VC on resource utilization. Of these articles few reported the overall findings, will 10.1% reported decreased resource utilization relative to in-person care (10.1%; 8/79), and 5.1% reported increased resource utilization (5.1%; 4/79) particularly around the amount of time required to deliver virtual care and the rate of conversion to in-person from VC.  There was heterogeneity in how efficiency/ resource utilization was defined; however, the most frequently utilized measures were the number of visits conducted using VC relative to in-person care (69.9%; 55/79), and the impact of VC on length of stay (17.7%; 14/79). |
|  | Safety (4.9%; 25/507) | | | - Adverse event rate relative to in-person care (36%; 9/25) - Need for ED/H visit (24%; 6/25) - Safety NOS (16%; 4/25) - Deaths (16%; 4/25) - Treatment complication rate (16%; 4/25) - Appropriateness of medical decision making (16%; 4/25) - Perceptions of safeness of VC (12%; 3/25) - Evaluation of clinical risk management (8%; 2/25) - Impact of VC on viral transmission rates (4%; 1/25) - Incidence of inadequate examination (4%; 1/25) - Incidence of malpractice (4%; 1/25) - Number of complaints (4%; 1/25) - Error rate (4%; 1/25) | Few articles assessed safety of VC (4.9%; 25/507). Of those articles assessing perceptions of the safety of VC (12%; 3/25), all were from the clinician perspective, and none were from the perspective of the patient. Most frequently assessed objective measures of safety included evaluating the VC-related adverse event rate relative to in-person care (36%; 9/25) and the need of an emergency department visit or hospitalization (24%; 6/25). |
|  | Effectiveness of Virtual Care (72.0%; 365/507) | | | - Lifestyle change/ Uptake of health behaviours (55.1%; 201/365) - Adherence to treatment/ medication (30.1%; 112/365) - Self-efficacy (30.4%; 111/365) - Self-management (10.1%; 37/365) - Health knowledge (7.9%; 29/365) - Self-care (5.7%; 21/365) - Patient engagement (2.7%; 10/365) - Health literacy (1.4%; 5/365) - Help seeking behaviours (0.82%; 3/365) - Patient empowerment (0.82%; 3/365) - Effectiveness NOS (0.54%; 2/365) - Self-esteem (0.54%; 2/365) - Self-awareness (0.27%; 1/365) | There was high heterogeneity of measures with most measures focused on how well virtual care modified health behaviours or the ability to self-manage. Most common measures were the uptake of lifestyle changes or health behaviours in response to VC such as improved diet or exercise behaviours (55.1%; 201/365), adherence to treatment or medications (30.1%; 112/365), and changes in self-efficacy (30.4%; 111/365). Very few studies reported on methods or cited validated measures. Of the minority of studies that utilized validated measures, the following measures were reported:   - Morisky Medication adherence score (4.3%; 16/365) - General self-efficacy scale (2.2%; 8/365) - Self-efficacy questionnaire (SEQ) 1.1%; 4/365) - Self-management for chronic disease (0.82%; 3/365) - Health education impact questionnaire (HEI-Q) (0.82%; 3/365) - Patient activation measure (0.54%; 2/365) - PROMIS self-efficacy for managing emotions (0.54%; 2/365) |
|  | Equity (5.5%; 28/507) | Marginalized/ vulnerable populations (89.3%; 25/28) | | - Narrative/ thematic (68%; 17/25) - Cultural appropriateness of care (20%; 5/25) - Factors associated with receipt of VC/ perceptions of VC based on regression analysis (12%; 3/25) | Of the articles examining equity issues related to VC, the majority focused on vulnerable or marginalized populations (89.3%; 25/28). However, there was a lack of objective measures of VC use/ delivery within the populations that were reported on. Most findings were qualitative thematic in nature or presented narratively (68%; 17/75), while few looked at cultural appropriateness of care (20%; 5/25), though no validated measure or standardized definition was used. Only three articles quantified associations between sociodemographic factors and VC perceptions/ receipt (12%; 3/25).  Most articles reporting on marginalized or vulnerable populations focused on a specific population:   - Indigenous population (32%; 8/25) - Migrants, refugees and asylum seeker (12%; 3/25) - Ethnicity/Race (28%; 7/25) - Incarcerated people (12%; 3/25) - Cultural identify NOS (16%; 4/25) - Socioeconomic status (8%; 2/25) |
|  |  | Language/ Literacy (3.6%; 1/28) | | Comprehension of text and medical terminology used (100%; 1/1) | Only one article reported specifically on issues of VC equity around availability/ integration of interpreters and literacy, and focused measuring whether or not the text or medical terminology used could be comprehended. No validated measure was utilized, issues of language and literacy were evaluated through qualitative themes of focus groups, interviews and open-text fields of surveys (100%; 1/1). |
|  |  | Gender (14.3%; 4/28) | | Gender based differences in uptake of virtual care (25%; 1/4)  Gender-based differences in effectiveness of virtual visits (25%; 1/4)  Call out gender-based inequities but have no measures of them (50%; 2/4) | Few studies examined gender-based disparities in VC (14.3%; 4/28). Of the studies that did report on gender, it was sometimes used interchangeably with sex; no formal or validated measures were reported. |
|  | Patient-Centeredness- Quality of the interaction/ communication (7.1%; 36/507) | Patient Perspective (94.4%; 34/36) | | - Therapeutic alliance (41.1%; 14/34) - Communication NOS (20.5%; 7/34) - Quality of patient/ clinician relationship/ bond (20.5%; 7/34) - Quality of communication by delivery modality (video vs telephone- 11.7%; 4/34) - Content of conversations (11.7%; 4/34) - Conversation duration (8.8%; 3/34) - Preference for VC vs in-person (2.9%; 1/34) - Factors associated with perceptions of quality of communication (2.9%; 1/34) - Trust/ perceived confidentiality (2.9%; 1/34) - Satisfaction with communication (2.9%; 1/34) - Patient expectations for care (2.9%; 1/34) - Depersonalization of care (2.9%; 1/34) - Availability of tailoring of care (2.9%; 1/34) - Attitudes towards VC (2.9%; 1/34) - Perceived clinician engagement/ attentiveness (2.9%; 1/34) - Relational continuity (2.9%; 1/34) - Conversation enjoyment (2.9%; 1/34) - Credibility (2.9%; 1/34) - Conversation dominance (2.9%; 1/34) | There was heterogeneity of measures for evaluating patient-centredness of care, though none of the included studies cited validated measures of patient-centredness. The most common measures were therapeutic alliance particularly in the treatment of mental health disorders (41.1%; 14/34), quality of the patient-clinician communication (20.5%; 7/34), and quality of the patient-clinician relationship or bond (20.5%; 7/34).  Methods that were utilized to evaluate patient centredness of care were:   - Interviews (11.7%; 4/34) - Focus groups (2.9%; 1/34) - Case analysis (2.9%; 1/34) - Qualitative NOS (5.8%; 2/34) - Surveys (44.1%; 15/34)   Validated measures that were reportedly used were:   - Working Alliance Inventory Short form (53.3%; 8/15) - Visit-specific Satisfaction Index VSQ-9 (6.6%; 1/15) - Helping Alliance Questionnaire (20%; 3/15) - Group Therapy Alliance Scale (6.6%; 1/15) - Therapeutic Alliance Scale (6.6%; 1/15) |
|  |  | Clinician Perspective (11.1%; 4/36) | | - Communication as a qualitative theme (50%; 2/4) - Knowledge/ Literacy (25%; 1/4) - Pre-interactional: attitudes, competencies, and cultural awareness (25%; 1/4) - Verbal communication: behavioral skills, timing, and types of clinician talk (25%; 1/4) - Nonverbal communication: eye contact, visual cues, and empathetic gestures (25%; 1/4) - Relational: rapport and relationship building (25%; 1/4) - Environmental: physical surroundings and privacy (25%; 1/4) - Educational: pre-professional or continuing development and evaluation of interpersonal skills with clinician–patient interaction (25%; 1/4) | Compared to the patient perspective, very few studies examine the quality of the VC interaction from the perspective of clinicians (11.1%; 4/36). The most common measure was quality of the communication, reported as a qualitative theme (50%; 2/4). None of the included studies reported utilizing a validated measure; where specified quality of the interaction was assessed using:   - Interviews (50%; 2/4) - Focus Groups (50%; 2/4) - Qualitative NOS (25%; 1/4) |
|  | Timeliness (8.6%; 44/507) | | | - Wait time for consultation (47.7%; 21/44) - Commuting time for patients/ clinicians (34.1%; 15/44) - Duration of consultation (22.7%; 10/44) - Time to initiate treatment (15.9%; 7/44) - Overall time savings (13.6%; 6/44) - Time off work/ school (11.4%; 5/44) - Physician’s time/ workload (9.1%; 4/44) - Delayed clinic visits (4.5%; 2/44) - Time to diagnosis (4.5%; 2/44) - Number of visits completed over time (4.5%; 2/44) - ER contact time (4.5%; 2/44) - Timeliness of care NOS (2.2%; 1/44) - Visit cancellations (2.2%; 1/44) - Delayed visits (2.2%; 1/44) - Timely availability of equipment to conduct VC visits (2.2%; 1/44) - Order processing time (2.2%; 1/44) - Conversion from in-person to VC visit (2.2%; 1/44) - Time to test result disclosure (2.2%; 1/44) | For studies reporting on timeliness (8.6%’ 44/507), all reported measures were quantifiable and objective though data sources varied by study. The most commonly reported measures were wait times for a VC consultation with a clinician relative to in-person care (47.7%; 21/44), changes in commuting times for patients or clinicians receiving/ delivering VC relative to in-person care (34.1%; 15/44), and the duration of the consultation (22.7%; 10/44). |
| **Client Outcomes (72.9%; 900/1233)** | Satisfaction (44.7%; 403/900) | | Satisfaction- Patient Perspective (93.3%; 281/301) | - Satisfaction NOS (94.6%; 266/281) - Acceptability NOS (5.33%; 15/281) - Perceived efficacy (3.2%; 9/281) - Preference for VC vs in-person (2.8%; 8/281) - Usability NOS (2.4%; 7/281) - Technical quality of visits (2.4%; 7/281) - Convenience (2.4%; 7/281) - Quality of the consultation (2.1%; 6/281) - Satisfaction with relationship with clinician (1.7%; 5/281) - Satisfaction with technology (1.4%; 4/281) - Ease of use (1.4%; 4/281) - Factors associated with satisfaction (1.4%; 4/281) - Perceived privacy and safety (1.4%; 4/281) - Willingness to recommend (1.4%; 4/281) - Perception of adequacy of care (1.4%; 4/281) - Satisfaction with VC experience (1.4%; 4/281) - Willingness to continue (1.4%; 4/281) - Perception of the overall quality of VC care (1.4%; 4/281) - Experience NOS (1.4%; 4/281) - Therapeutic alliance (1.1%; 3/281) - Accessibility (0.71%; 2/281) - Comfort/ confidence with use (0.71%; 2/281) - Perceived usefulness (0.71%; 2/281) - Feasibility NOS (0.35%; 1/281) - Satisfaction with VC scheduling (0.35%; 1/281) - Length of session (0.35%; 1/281) | Of the articles reporting on satisfaction with VC from the patient perspective (93.3%; 281/301), satisfaction was generally not clearly defined (94.6%; 266/281). Several articles evaluated acceptability, perceived efficacy or preference for VC over in-person visits from the patient perspective as measures of satisfaction with VC.  The majority of articles evaluated satisfaction with survey measures (56.2%; 158/281), though the following measures were also used:   - Interviews (4.6%; 13/281) - Focus groups (0.35%; 1/281) - Qualitative NOS (1.1%; 3/281)   Articles reported on using both validated measures and generic scales, including:   - Visual analog scale NOS (2.5%; 4/158) - Likert NOS (20.2%; 32/158) - Client Satisfaction Questionnaire (12.6%; 20/158) - Short Patient Satisfaction Survey-18 (6.3%; 10/158) - Visit Specific Satisfaction Questionnaire (3.2%; 5/158) - Patient Experience Questionnaire (1.9%; 3/158) - Healthcare Satisfaction Questionnaire (1.9%; 3/158) - Telemedicine Satisfaction and Usefulness Questionnaire (1.9%; 3/158) - Telemedicine Usability Questionnaire (1.9%; 3/158) - Working alliance inventory (1.3%; 2/158) - Charlson Psychiatric Outpatient Satisfaction Questionnaire (1.3%; 2/158) - Treatment Satisfaction Questionnaire (1.3%; 2/158) - Group Health Association of America Consumer Satisfaction Survey (1.3%; 2/158) - Patient Satisfaction with Nursing Care Quality Questionnaire (1.3%; 2/158) - Consumer Assessment of Healthcare Providers and Systems (1.3%; 2/158) - Telemedicine Satisfaction and Acceptance Questionnaire (0.63%; 1/158) - Short-form 12 (0.63%; 1/158) - Diabetes Treatment Satisfaction Questionnaire (0.63%; 1/158) - Treatment Acceptability Rating Scale (0.63%; 1/158) - System Usability Questionnaire (0.63%; 1/158) - Experience of Healthcare and Outcomes Questionnaire (0.63%; 1/158) - Canadian Healthcare Evaluation Project Patient Questionnaire (0.63%; 1/158) - Norwegian Outpatient Experience Questionnaire (0.63%; 1/158) - Oxford Maternity Diabetes Treatment Satisfaction Questionnaire (0.63%; 1/158) - Psychiatric Outpatient Satisfaction Scale (0.63%; 1/158) - Patients Assessment of Chronic Illness Care (0.63%; 1/158) - Quebec User Evaluation of Assistive Technology (0.63%; 1/158) - UK National Health Services Outpatient Questionnaire (0.63%; 1/158) - Outpatient Satisfaction Scale (0.63%; 1/158) - Q-LES-Q (0.63%; 1/158) - Therapeutic Alliance Scale (0.63%; 1/158) - Home Care Client Satisfaction Instrument (0.63%; 1/158) - Session Evaluation Questionnaire (0.63%; 1/158) |
|  |  |  | Satisfaction- Clinician Perspective (28.5%; 86/301) | - Satisfaction NOS (87.2%; 75/86) - Acceptability (11.6%; 10/86) - Technical difficulties (6.9%; 6/86) - Perceived usefulness (5.8%; 5/86) - Quality of the interaction (4.6%; 4/86) - Attitudes towards VC (3.4%; 3/86) - Suitability/ appropriateness of VC (3.4%; 3/86) - Willingness to recommend (3.4%; 3/86) - Satisfaction with technology (3.4%; 3/86) - Satisfaction relative to in-person (2.3%; 2/86) - Ease of use (2.3%; 2/86) - Privacy (2.3%; 2/86) - Preference for in-person vs VC (2.3%; 2/86) - Therapeutic alliance (2.3%; 2/86) - Clinician-patient rapport (2.3%; 2/86) - Intent to continue using VC (2.3%; 2/86) - Usability (1.2%; 1/86) - Perceived safety (1.2%; 1/86) - Quality of clinician-patient relationship (1.2%; 1/86) - Integration of VC (1.2%; 1/86) - Satisfaction with training (1.2%; 1/86) - Comfort with use (1.2%; 1/86) | Of the articles reporting satisfaction with VC from the clinician perspective (28.5%; 86/301), 17.4% reported positive impressions of VC (15/86). Many articles evaluated satisfaction without defining the concept (87.2%; 75/86), or used acceptability, experiencing technical difficulties while delivering VC and perceived usefulness of VC in a specific clinical scenario as proxy measures of satisfaction. Data on satisfaction was collected through a variety of methods:   - Survey (50%; 43/86) - Interviews (2.3%; 2/86) - Focus groups (2.3%; 2/86) - Qualitative NOS (2.3%; 2/86) |
|  |  |  | Experience- Patient Perspective (59.6%; 90/151) | - Patient experience NOS (54.4%; 49/90) - Satisfaction (22.2%; 20/90) - Acceptability (13.3%; 12/90) - Attitudes towards VC (13.3%; 12/90) - Perceived efficacy (11.1%; 10/90) - Convenience (10%; 9/90) - Factors associated with VC experience (10%; 9/90) - Ease of use (8.8%; 8/90) - Preference for VC vs in-person (6.6%; 6/90) - Privacy (5.5%; 5/90) - Comfort/ Confidence with VC (5.5%; 5/90) - Technological features (4.4%; 4/90) - Perceived usefulness (4.4%; 4/90) - Feasibility NOS (3.3%; 3/90) - Technical challenges (3.3%; 3/90) - Patient-clinician rapport (3.3%; 3/90) - Accessibility (2.2%; 2/90) - Trust (2.2%; 2/90) - Willingness to use (2.2%; 2/90) - Usability NOS (2.2%; 2/90) - Quality of experience with VC (1.1%; 1/90) - Logistical needs (1.1%; 1/90) - Impact of VC on everyday life (1.1%; 1/90) - Perceptions of clinician interpersonal skills (1.1%; 1/90) - Intention to use VC (1.1%; 1/90) - Perceived safety of VC (1.1%; 1/90) - Goodness of fit (1.1%; 1/90) | There was high heterogeneity in measures of patient experience with VC, whereby many articles did not define the concept (54.4%; 49/90). Of the articles reporting on experience, 33.3% reported a positive experience. While many articles indicated that they evaluated patient experience, they most commonly utilized satisfaction, acceptability and attitudes towards VC as proxy measures. A variety of methods were used to evaluate experience, including:   - Surveys (60%; 54/90) - Interviews (50%; 45/90) - Qualitative case studies 4.4%: (4/90) - Qualitative NOS (3.3%; 3/90) - Panel discussion (1.1%; 1/90) - Observations (1.1%; 1/90)   Validated measures of which were reported on included:   - Experience of care questionnaire (1.8%; 1/54) - Patient experience Questionnaire (3.7%; 2/54) - Diabetes Treatment Satisfaction Questionnaire (1.8%; 1/54) - Patient Assessment of Chronic Illness Care (11.8%; /54) - Patient Satisfaction Questionnaire Short Form (1.8%; 1/54) - Canadian Health Care Evaluation Project Questionnaire (1.8%; 1/54) - Functional Assessment of Chronic Illness Therapy- Treatment/ Patient Satisfaction (1.8%; 1/54) - Usefulness, Satisfaction and Ease of Use questionnaire (1.8%; 1/54) - System usability scale (1.8%; 1/54) - Application rating scale (1.8%; 1/54) - After scenario questionnaire (1.8%; 1/54) - Telehealth Acceptance measure (1.8%; 1/54) |
|  |  |  | Experience- Caregiver Perspective (26.4%; 40/151) | - Satisfaction NOS (35%; 14/40) - Caregiver mental health (30%; 12/40) - Wellbeing/ Quality of life (27.5%; 11/40) - Caregiver burden (25%; 10/40) - Caregiver stress/ distress (25%; 10/40) - Social support (17.5%; 7/40) - Attitude towards VC (10%; 4/40) - Caregiver strain (10%; 4/40) - Competence (10%; 4/40) - Caregiver experience NOS (7.5%; 3/40) - Quality of caregiver-clinician communication (7.5%; 3/40) - Convenience (5%; 2/40) - Perceived usefulness (5%; 2/40) - Coping (5%; 2/40) - Comfort with use (5%; 2/40) - Acceptability (5%; 2/40) - Caregiver-clinician relationship (2.5%; 1/40) - Preference for VC vs in-person (2.5%; 1/40) | Far fewer articles examined experience with VC from the caregiver perspective (48 vs 90). Experience was defined as satisfaction in many of the articles (35%; 14/40). Additionally, many articles used measures of caregiver mental health, wellbeing and quality of life, and burden as proxy measures of experience. Data on caregiver experience was collected via:   - Surveys (50%; 20/40) - Focus groups (2.5%; 1/40) - Interviews (5%; 2/40) - Qualitative NOS (10%; 4/40)   Validated survey measures that were reportedly used included:   - Zarit Burden Inventory (20%; 8/40) - Centre for Epidemiological Studies Depression scale (17.5%; 7/40) - Caregiver Quality of Life Index (12.%; 5/40) - Perceived Stress Scale (10%; 4/40) - Short-form 36 (SF-36; 10%; 4/40) - Caregiver Strain Index (10%; 4/40) - Short-form 12 (SF-12; 2.5%; 2/40) - Medical Outcomes Study Social Support Survey (2.5%; 2/40) - Caregiver Burden Inventory (2.5%; 2/40) |
|  |  |  | Experience- Clinician Perspective (31.8%; 48/151) | - Clinician experience NOS (25%; 12/48) - Technical components/ technical difficulties (20.8%; 10/48) - Satisfaction (20.85; 10/48) - Training needs (12.5%; 6/48) - Acceptability (12.5%; 6/48) - Patient-clinician communication (12.5%; 6/48) - Suitability of VC (10.4%; 5/48) - Ease of use (10.4%; 5/48) - Perception of impact on clinical outcomes (8.33%; 4/48) - Impact of VC on workflows (8.33%; 4/48) - Healthcare professional collaboration (8.33%; 4/48) - Privacy/ security (8.33%; 4/48) - Willingness to use/ continue (6.2%; 3/48) - Productivity/ efficiency of care delivery (6.2%; 3/48) - Clinical confidence (6.2%; 3/48) - Impact of VC on workload (6.2%; 3/48) - Therapeutic alliance (6.2%; 3/48) - Usability (6.2%; 3/48) - Clinician-patient relationship/ trust (6.2%; 3/48) - Attitudes towards VC (4.2%; 2/48) - Impact of VC on health care practice (4.2%; 2/28) - Flexibility of VC (4.2%; 2/48) - Perceived value (4.2%; 2/48) - Convenience (4.2%; 2/48) - Digital burn-out (2.1%; 1/48) - Consistency of care (2.1%; 1/48) - Continuity of care (2.1%; 1/48) - Change in clinician role (2.1%; 1/48) - Uptake of VC (2.1%; 1/48) | Relative to patients, fewer articles described clinician experience with VC (48 vs 90), and many did not define what clinician experience measured (25%; 12/48). 33.3% of articles reported that clinicians had a positive experience with delivering VC (16/48). Technical difficulties encountered while delivering VC and satisfaction with VC were commonly reported as proxy measures of experience. Data was collected through a variety of methods:   - Interviews (45.8%; 22/48) - Focus groups (14.5%; 7/48) - Surveys (47.9%; 23/48) - Action research approaches NOS (2.1%; 1/48) - Chart audits (2.1%; 1/48) - Observations (4.2%; 2/48) - Qualitative NOS (8.33%; 4/48) |
|  | Function (6.88%; 62/900) | | Privacy and Security (11.3%; 7/62) | - Qualitative Themes (71.4%; 5/7) - Use of encryption for transmission (28.5%; 2/7) - Compliance with relevant regulations (28.5%; 2/7) | Few articles examined issues of privacy and security (11.3%; 7/62). Articles that did utilizes qualitative methods (60%; 3/5), surveys (60%; 3/5), and interviews (20%; 1/5). |
|  |  |  | Usability- Patient Perspective (89.7%; 52/58) | - Usability NOS (40.3%; 21/52) - Satisfaction (30.7%; 16/52) - Ease of use (21.5%; 11/52) - Acceptance of technology (17.3%; 9/52) - Perceived usefulness (15.3%; 8/52) - Usage NOS (7.6%; 4/52) - Perceptions of technical features (5.7%; 3/52) - Frequency of use (5.7%; 3/52) - Factors associated with perceptions on usability (3.8%; 2/52) - Factors associated with usage (3.8%; 2/52) - Confidence/ comfort in using VC (3.8%; 2/52) - Duration of use (3.8%; 2/52) - Trust in VC (1.9%; 1/52) - Efficiency of use (1.9%; 1/52) - Accessibility (1.9%; 1/52) - Willingness to use (1.9%; 1/52) - Convenience (1.9%; 1/52) - Ease of learning to use VC (1.9%; 1/52) - User retention (1.9%; 1/52) - Likability (1.9%; 1/52) - Technical challenges (1.9%; 1/52) | Of the articles examining usability of VC from the patient perspective (89.7%; 52/58), usability was not clearly defined in many cases (40.3%; 21/52). Usability was most often measured as satisfaction with VC (30.7%; 16/52), ease of use (21.5%; 11/52), and acceptance of the technology (17.3%; 9/52). A variety of methods were utilized to assess usability, including:   - Qualitative NOS (9.6%; 5/52) - Interviews (17.3%; 9/52) - Focus groups (1.9%; 1/52) - Secondary data analysis (1/52) - Task analysis methods (5.7%; 3/52) - Log books/ usage logs (5.7%; 3/52) - Surveys (51.9%; 27/52)   Surveys included generic visual analog scales (7.4%; 2/27), as well as a number of validated measures:   - System Usability Scale (48.1%; 13/27) - Technology Usability Scale (7.4%; 2/27) - Post Study Usability Questionnaire (7.4%; 2/27) - Subjective Usability Scale (3.7%; 1/27) - Virtual System Instruction and Assessment Scale (3.7%; 1/27) - Technology Acceptance Questionnaire (3.7%; 1/27) - Mobile App Rating Scale (3.7%; 1/27) - Telehealth Acceptance Measure (3.7%; 1/27) - Unified Theory of Acceptance and Use of Technology scale (3.7%; 1/27) |
|  |  |  | Usability- Clinician Perspective (12.1%; 7/58) | - Usability NOS (14.2%; 1/7) - Acceptance of the technology (14.2%; 1/7) - Intention to Use (14.2%; 1/7) - Comfort with use/ ease of use (42.8%; 3/7) - Satisfaction with usability (14.2%; 1/7) - Utility- how useful was it (28.5%; 2/7) - Technical problems (14.2%; 1/7) | Relative to the patient perspective, few articles examined usability from the clinician perspective (52 vs 7). Usability most commonly evaluated comfort/ ease of VC use (42.8%; 3/7). Most articles utilized surveys to evaluate usability (85.7%; 6/7) though interviews were also reported (14.2%; 1/7). Most articles which utilized surveys did not specify that validated measures were used (71.4%; 5/7), though one study utilized the System Usability Scale. |
|  | Clinical Outcomes (81.5%; 734/900) | | Psychosocial Outcomes (59.7%; 438/734) | - Depression (56.6%; 248/438) - Anxiety (42.4%; 186/438) - Quality of life (23.1%; 101/438) - Mental health/ psychosocial outcomes NOS (19.6%; 86/438) - Distress (11.6%; 51/438) - Stress (9.8%; 43/438) - Fatigue (7.9%; 35/438) - Social functioning (7.9%; 35/438) - Mood (4.3%; 19/438) - PTSD (4.3%; 19/438) - Wellbeing (3.8%; 17/438) - Coping (2.7%; 12/438) - Sleep (2.7%; 12/438) - Grief (2.5%; 11/438) - Eating disorder severity/ symptoms (2.2%; 10/438) - Psychosis (1.8%; 8/438) - Suicidal ideation (1.8%; 8/438) - Emotional functioning (1.5%; 7/438) - Loneliness (1.5%; 7/438) - Fear of recurrence (0.91%; 4/438) - Panic/ panic attaches (0.91%; 4/438) - Burnout (0.68%; 3/438) - Agitation (0.68%; 3/438) - Addiction severity (0.68%; 3/438) - Intimate partner violence (0.68%; 3/438) - Dementia severity/ symptoms (0.68%; 3/438) - Self-harm/ Self-injurious thoughts (0.45%; 2/438) - Compassion (0.45%; 2/438) - Agoraphobia (0.45%; 2/438) - Hopelessness (0.45%; 2/438) - Empowerment (0.45%; 2/438) - Mania (0.22%; 1/438) - Apathy (0.45%; 2/438) - Resilience (2/438) - Hope (0.22%; 1/438) - Guilt (0.22%; 1/438) - Disconnectedness (0.22%; 1/438) - Self-esteem (0.22%; 1/438) - Suffering (0.22%; 1/438) - Suffering (0.22%; 1/438) - Attachment (0.22%; 1/438) - Empathy (0.22%; 1/438) - Relationship quality (0.22%; 1/438) - Family functioning (0.22%; 1/438) | As many of the included articles examined use of VC in treatment of mental health conditions and cognitive impairment, there were many articles that examined the effectiveness of virtual care on improving psychosocial outcomes (59.7%; 438/734). The most commonly evaluated measures were generally clinical condition agnostic, and were impact on severity of depression (56.6%; 248/438) or anxiety (42.4%; 186/438), or on quality of life (23.1%; 101/438). There was extremely high heterogeneity of survey measures used to assess severity of psychosocial symptoms and measures were specific to the clinical condition of study. |
|  |  |  | Physical Outcomes (76.3%; 560/734) | - Change in disease-specific laboratory values/ tests/ vital signs (35.5%; 199/560) - Symptom severity (21.2%; 119/560) - Physical fitness/ physical activity (20%; 112/560) - Adverse event incidence (17.1%; 96/560) - Acute care visits (15.5%; 87/560) - Complication rate (12.1%; 68/560) - General health/ physical functioning (11.8%; 66/560) - Pain (11.6%; 65/560) - Readmission rate (6.2%; 35/560) - Cognitive changes (5.5%; 31/560) - Rate of disability (4.4%; 25/560) - Clinical improvement/ efficacy NOS (3.7%; 21/560) - Length of hospital stay (3.7%; 21/560) - Symptom management activities (3.2%; 18/560) - Nutrition/ diet (2.8%; 16/560) - Visits to primary care clinician (2.5%; 14/560) - Rate of diagnosis (2.1%; 12/560) - Disease management decision making (1.6%; 9/560) - Healthcare resource utilization NOS (1.4%; 8/560) - Physical wellbeing (1.4%; 8/560) - Morbidity risk scores (1.4%; 8/560) - Motor function (1.3%; 7/560) - Sexual function (1.1%; 6/560) - Remission (1.1%; 6/560) - Symptom distress (1.1%; 6/560) - Recovery days/ time to recovery (1.1%; 6/560) - Disease progression (0.5%; 3/560) - Perceived change in health state (0.5%; 3/560) - Breastfeeding (0.4%; 2/560) - Discharge destination (0.4%; 2/560) - Rate of early detection (0.2%; 1/560) - Incidence of adverse events requiring follow-up (0.2%; 1/560) | There was high heterogeneity of measures evaluating physical clinical outcomes (76.3%; 560/734) as they were generally specific to the clinical condition under study. Most commonly, physical outcomes were evaluated as changes in disease-specific laboratory values or vital signs (35.5%; 199/560), as changes in symptom severity over time (21.2%; 119/560), or as changes in physical fitness or activity (20%; 112/560). |
|  |  |  | Mortality (12.94%; 95/734) | - All-cause Mortality (41.1%; 39/95) - Disease-related Mortality (21.21%; 23/95) - In-hospital Mortality (1.05%; 1/95) - Decision-making Related Mortality (1.05%; 1/95) - Mortality- not specified (43.15%; 41/95) | There was generally consensus on how mortality was evaluated with most articles reporting on either all-cause mortality (41.1%; 39/95) or disease-related mortality (21.2%; 23/95). Methods for reporting mortality included:   - Narratives (34.73%; 33/95) - Percentage/ proportion (26.31%; 25/95) - Relative Risk (21.21%; 23/95) - Odds Ratio (14.73%; 14/95) - Absolute number of events (1.05%; 1/95) - Hazard Ratio (1.05%; 1/95) |
